# Metal and Additive-Free Nondirected Meta-C–S Bond Formation on Anilines: Toward Biologically Relevant *S*‑Aryl Dithiocarbamates

**DOI:** 10.1021/acscentsci.5c01231

**Published:** 2025-09-15

**Authors:** Sushanta Kumar Parida, Srishti Sanghi, Ardhendu Mondal, Nameeta Choudhary, Prahallad Meher, Priyanka Singh, Sandip Murarka

**Affiliations:** † Department of Chemistry, 231955 Indian Institute of Technology Jodhpur, Karwar-342037, Rajasthan, India; ‡ Department of Bioscience and Bioengineering, 231955 Indian Institute of Technology Jodhpur, Karwar-342037, Rajasthan, India

## Abstract

The site-selective C–H functionalization to install meta-C–S bonds on aniline derivatives is highly desirable, due to the preponderance of resulting compounds in numerous medicinally relevant compounds. However, the execution of the same is far from being trivial, due to the intrinsic electronic bias of anilines and concerns associated with the ready availability of an appropriate and odorless sulfur source. Accordingly, we demonstrate a metal- and additive-free, one-pot, multicomponent reaction between *p*-anisidines/anilines, carbon disulfide, and aliphatic amines to install an otherwise difficult meta-C–S bond on anilines with exclusive regioselectivity, while furnishing an array of biologically relevant anisidine-derived *S*-aryl dithiocarbamates. The method exhibits broad scope with appreciable functional group tolerance, as demonstrated through late-stage modification of a variety of amino acids, pharmaceuticals, and natural products. Importantly, final *S*-aryl dithiocarbamates are amenable to further synthetic manipulations, furnishing highly valuable and medicinally relevant sulfur-containing functional moieties, such as thiols, thioethers, and sulfones. Furthermore, in vitro evaluations demonstrate that many of the synthesized dithiocarbamates exhibit promising drug-like properties, demonstrating antiproliferative activity on a nanomolar level for breast cancer cell lines by affecting microtubule dynamics.

## Graphical abstract

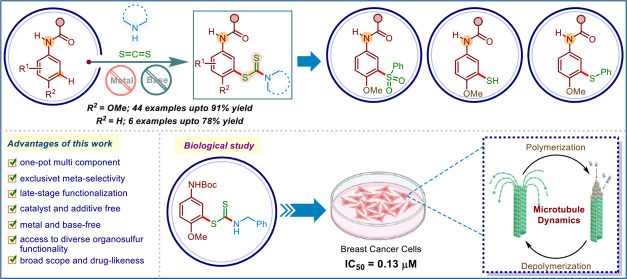

## Introduction

Sulfur-containing organic molecules are widely prevalent in natural products, pharmaceuticals, agrochemicals, and functional materials.^1–4^ Notably, organosulfur compounds account for 20% of all drugs approved by the FDA, and there are more than 200 drugs featuring C–S bonds that are prescribed to cure several major diseases, including HIV, insomnia, gastroesophageal reflux, diabetes, cancer, schizophrenia, and Parkinson’s disease among many others.^5,6^ Moreover, as many as six sulfur-containing functional groups, such as sulfonamide, disulfide, sulfone, sulfide, thiol, and sulfoxide, are recurring motifs in various medicinally relevant compounds.^7^ Accordingly, various synthetic approaches, including transition-metal-catalyzed cross-couplings, addition reactions, Stadler–Ziegler reaction, and radical-nucleophilic aromatic substitution reaction, among others, have been successfully deployed for the construction of C–S bonds.^8–10^ However, most of these methods involve toxic and expensive transition metals, air- or moisture-sensitive ligands, stoichiometric additives, and harsh conditions. Nonetheless, the recent emergence of visible light-mediated synthetic approaches has provided environmentally benign alternatives to forge C–S bonds.^11^ Interestingly, meta-C–S bond-bearing substituted anilines serve as marketed drugs and impart anticancer, antiangiogenic, anti-inflammatory, and antinociceptive activities (Scheme 1A).^12–15^ In this context, synthetic methods enabling direct meta-C–H functionalization to install C–S bonds on electron-rich aniline derivatives in a site-selective manner are highly desirable, albeit far from being trivial. The traditional electrophilic aromatic substitution exploiting inherent electronic reactivity provides access to ortho- and para-substituted aniline derivatives with a possibility of the formation of a mixture of isomers (Scheme 1B). The selective *ortho*-C–S bond formation on quinone imine ketals through an acid-promoted pathway employing thiols as nucleophiles has been reported as well.^16^ Although there are several methods for the transition-metal-catalyzed C–H thiolation of unactivated/activated arenes with/without the assistance of directing groups, these methods could not be extended to the synthesis of corresponding meta-thiolated congeners, presumably due to the inherent electronic bias.^17–22^ Over the past decades, various synthetic strategies relying on transition-metal-catalyzed/free transformations, Catellani norbornene relay procedure, scrupulously designed ligand-based reactivity, and template-based removable directing groups have been disclosed to accomplish C–C and C–X (B, Cl, N etc.) bond formation at the meta-position of anilines and their derivatives.^23–35^ Howbeit, the application of these strategies in realizing meta-thiolation of anilines remained underexplored.^36^ Other than the challenges associated with overriding the intrinsic electronic bias of anilines, the ready availability of an appropriate and odorless sulfur source is also a major bottleneck in executing meta-selective C–S bond construction.^37^ The conventional methods to forge meta-C–S bonds on anilines require a multistep approach involving functional group interconversion, prefunctionalized starting materials, transition metal catalysts, and high reaction temperatures (Scheme 1C).^15,38,39^ Accordingly, the development of a sustainable, inexpensive, mild, and environmentally benign technology for the installation of diverse sulfur functionalities at the meta-position of anilines in a site-selective manner is highly sought after.

**Scheme 1:**
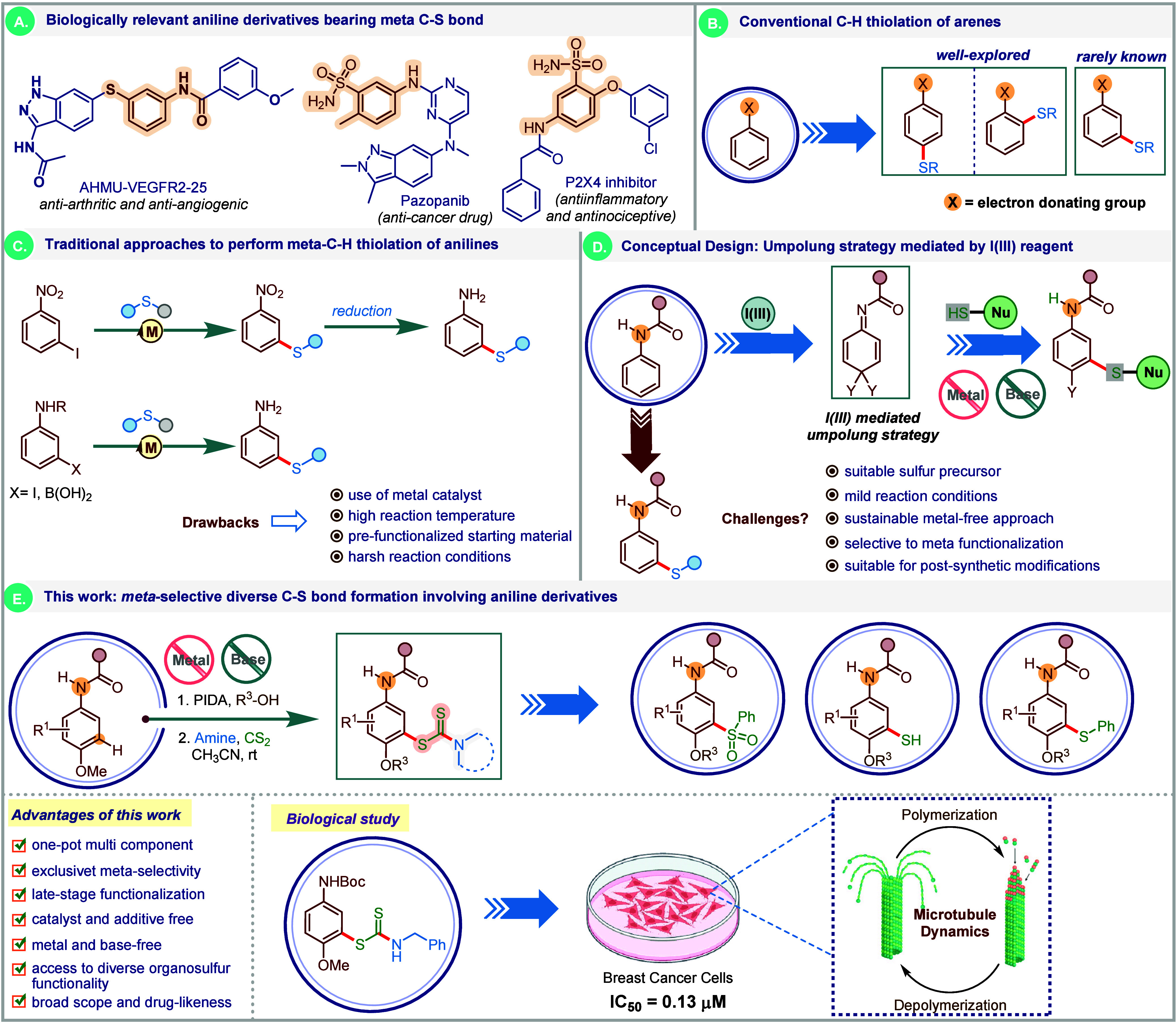
(A) Bioactive Aniline Derivatives Comprising meta-C–S Bond. (B) Conventional C–H Thiolation of Arenes. (C) Traditional Methods to Access meta-C–H Thiolation of Anilines. (D) Our Conceptual Design Based on Umpolung Strategy Mediated by Iodine­(III) Reagent. (E) This Work: meta-C–S Bond Formation on Anisidines/Anilines: Synthesis of *S*-aryl Dithiocarbamates

*S*-aryl dithiocarbamates belong to the privileged class of compounds with diverse applications spanning across diverse fields.^40–44^ Although there are a variety of methods available for the synthesis of this class of compounds, a suitable metal-free method enabling the synthesis of *S*-aryl dithiocarbamates with diverse multisubstitution patterns on the phenyl ring is still desirable.^45–51^ Inspired by the biological importance of the meta-thiolated anilines and anisidine-derived *S*-aryl dithiocarbamates, we investigated the development of atom-economic and sustainable technology based on easily accessible and inexpensive reacting partners to access these classes of compounds while forging meta-C–S linkage.

We envisaged the possibility of generating extremely reactive quinone imine ketals (QIK)^35,52–63^ from anilines or anisidines using iodine­(III) reagents^64,65^ as mild organic oxidants and reacting them with carbon disulfide, and amines to afford corresponding *S*-aryl dithiocarbamates, while installing the meta-C–S linkage in a site-selective manner (Scheme 1D). Despite the availability of competing electrophilic sites, we reasoned that after in-situ generation, QIKs will be appropriately aligned to serve as a Michael acceptor and trigger the conjugate addition of another in-situ-generated nucleophilic species, i.e., thiocarbamic acids, under mild reaction conditions. Such a one-pot metal-free multicomponent strategy would be novel, highly efficient, and fascinating from the perspective of step economy and green chemistry. As part of our program on hypervalent iodine­(III) reagents,^64,66–75^ and driven by our interest in dithiocarbamates,^67,68,76^ we disclose an additive- and metal-free multicomponent reaction between *N*-protected p-anisidines/anilines, carbon disulfide, and cyclic and acyclic amines to establish a site-selective meta-C–S linkage while furnishing an array of highly substituted anisidine-derived *S*-aryl dithiocarbamates (Scheme 1E). We successfully demonstrate that the final dithiocarbamates are amenable to further synthetic modifications to afford highly valuable and biologically relevant functional moieties, such as thiols, thioethers, and sulfones. Such a divergent approach, facilitating access to a variety of meta-thiolated anisidine derivatives, is noteworthy. Moreover, through in vitro studies we reveal that many of the synthesized *S*-aryl dithiocarbamates exhibit promising antiproliferative activity against breast cancer cell lines by affecting microtubule dynamics.

## Results and Discussion

We began our studies by selecting *N*-Boc *p*-anisidine **1a**, piperidine **3a**, and carbon disulfide (CS_2_) as reacting partners to explore the reaction conditions (Table 1). Initially, *p*-anisidine **1a** (0.1 mmol, 1 equiv) was dearomatized, and the reactive QIK **2** was generated in situ by stirring the reaction mixture in methanol using (diacetoxy)­iodobenzene (PIDA) (1.2 equiv) as a mild organic oxidant. Afterward, methanol was evaporated, the resulting residue was dissolved in dichloromethane, and carbon disulfide (2.5 equiv) and piperidine **3a** (1.2 equiv) were added under a nitrogen atmosphere. Delightfully, the planned Michael addition took place to afford the desired *S*-aryl dithiocarbamate **4** in 91% yield (entry 1, Table 1). The structure of compound **4** was confirmed by single-crystal X-ray analysis (see the SI for details). The yield remained at similar levels in dichloroethane (DCE) and toluene, and a slight reduction in yield was observed in the case of THF (Table 1, entries 2–4). However, upon switching the solvent from DCM to acetonitrile (CH_3_CN), the yield escalated to 92% (Table 1, entry 5). Among the other solvents tested (1,1,1,3,3,3-hexafluoro-2-propanol (HFIP), acetone, H_2_O), CH_3_CN remained the optimal one (Table 1, entries 6–8). Notably, no beneficial effect was observed either by prolonging or reducing the reaction time (Table 1, entries 9 and 10). It is important to mention that an inert atmosphere was crucial to the observed efficacy of the process, as a reduction in yield alongside the formation of decomposition products was observed when the reaction was carried out under open air (76%; see Table 1, entry 11). While reducing the loading of either CS_2_ or PIDA had a detrimental effect on the reaction outcome, the yield did not improve when the stoichiometry was increased (Table 1, entries 12–15).

**Table 1:**
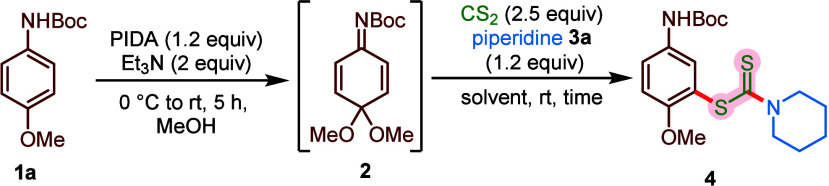
Optimization of the Reaction Conditions

entry	solvent	time (h)	yield **4** ^b^ (%)
1​	DCM	1	91
2​	DCE	1	89
3​	toluene	1	90
4​	THF	1	84
**5**	**ACN**	**1**	**92**
6​	HFIP	1	74
7​	acetone	1	78
8​	H_2_O	4	35
9​	CH_3_CN	24	86
10​	CH_3_CN	0.5	78
11​^c^	CH_3_CN	1	76
12​^d^	CH_3_CN	1	78
13​^e^	CH_3_CN	1	90
14​^f^	CH_3_CN	1	82
15​^g^	CH_3_CN	1	91

a Reaction conditions: **1a** (0.1 mmol), **3a** (0.12 mmol), PIDA (0.12 mmol), and CS_2_ (0.25 mmol), in solvent (1 mL) at 0 °C to room temperature.

b Isolated yield.

c Without N_2_.

d 1.5 equiv CS_2_.

e 3 equiv CS_2_.

f 1 equiv PIDA.

g 2 equiv PIDA.

Having optimized reaction conditions, we investigated the scope of the metal-free multicomponent reaction manifold by reacting *p*-anisidine **1** with electronically and structurally diverse amines **3** (Scheme 2). A variety of medicinally relevant cyclic aliphatic amines, including piperidine, pyrrolidine, morpholine, *N*-Boc-piperazine, tetrahydroisoquinoline, and azepane, participated in the transformation to deliver the corresponding products (**4**–**9**) in good to excellent yields (78%–91%). Besides, aliphatic cyclic amines, the one-pot methodology was potent for acyclic secondary aliphatic amines, affording respective *S*-aryl dithiocarbamates (**10**–**12**) in good yields. Delightfully, a series of benzyl and heterobenzyl primary amines embedded with heterocycles, such as pyridine, furan, and thiophene, underwent smooth transformation, furnishing products (**13**–**16**) in good yields (71%–78%). Similarly, the reaction was compatible with aliphatic primary amines, such as methyl, cyclohexyl, and allyl amines, to provide **17**–**19** in good yields (68%–79%). Notably, enantioenriched (*S*)-α-methylbenzylamine demonstrated facile reactivity to provide the corresponding chiral dithiocarbamate **20** in 81% yield. Unfortunately, the reaction between **1a** with aqueous ammonia and CS_2_, leading to aryl carbamodithioate, was unsuccessful.

**Scheme 2:**
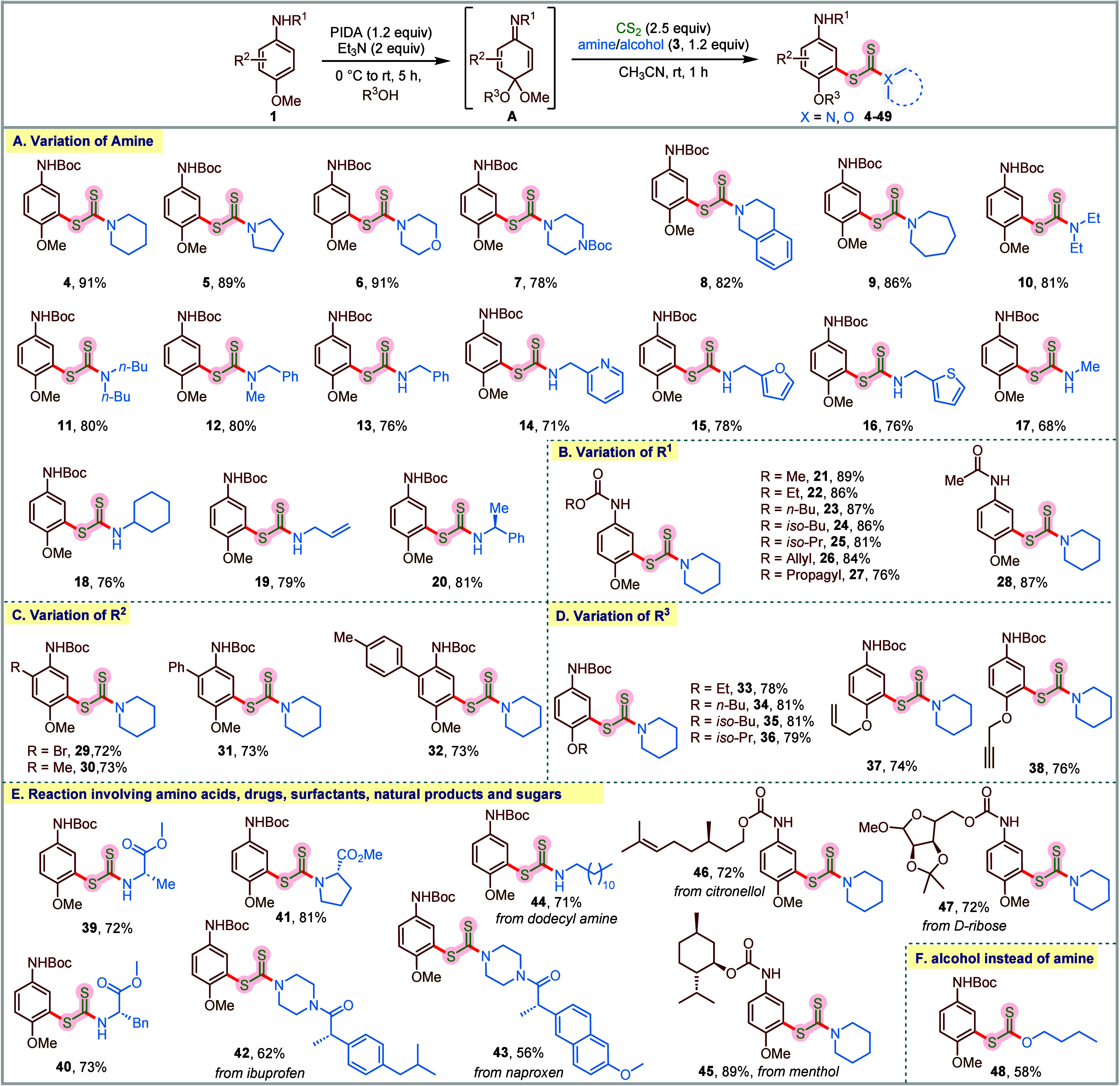
Scope of the Reaction

a Reaction conditions: **1** (0.2 mmol), **3** (0.24 mmol), PIDA (0.24 mmol), and CS_2_ (0.5 mmol), in CH_3_CN (2 mL) at 0 °C to room temperature.
b Isolated yield.

Afterward, we explored the scope of *N*-protected para-anisidine derivatives by reacting with piperidine under standard conditions (Scheme 2). Various carbamates, including those with allyl and propargyl functionality as nitrogen protecting groups (variation of R^1^), were tolerated to furnish the expected dithiocarbamates (**21**–**27**) in good to excellent yields (76%–89%). Moreover, anisidine with a less sterically demanding and readily removable acetyl protecting group underwent meta-selective dithiocarbamate formation in excellent yield (**28**, 87%). Next, we extended the scope to *N*-Boc-protected *p*-anisidines with diverse arene-bearing substituents, such as 2-Br, 2-Me, 2-Ph, and 2-tolyl (variation of R^2^), affording anticipated meta-dithiocarbamate-substituted anisidines (**29**–**32**) in good yields. It is interesting to note that, despite being less electrophilic due to the presence of relatively electron-rich substituents, these anisidines demonstrated appreciable reactivity under the reaction conditions. Notably, the dearomatization step and subsequent reaction could also be carried out in the presence of a palette of structurally diverse alcoholic solvents (ethanol, propanol, butanol, isopropanol, and isobutanol, variation of R^3^), to yield the desired products (**33**–**36**) in good yields (78%–81%). The reaction could also be carried out with allylic and propargylic alcohols, providing respective products (**37**, 74% and **38**, 76%), suitable for further synthetic manipulations. It is important to note that, in these cases, a selective formation of corresponding alcohol-substituted products (**33**–**38**) without the formation of compound **4** was observed, presumably due to the better leaving group aptitude of the methoxy group as compared to other employed alkoxy groups.^35^ However, when the reaction was performed on a 1 mmol scale for the synthesis of compound **35**, along with the desired product (70% yield), formation of compound **4** in 10% yield was also detected (see the Supporting Information for details).

Gladly, the methodology was successfully applied to the late-stage modification of amino acids, such as alanine (**39**, 72%), phenylalanine (**40**, 73%), and l-proline (**41**, 81%), pharmaceuticals (such as ibuprofen (**42**, 62%) and naproxen (**43**, 56%)), surfactants (such as dodecyl amine (**44**, 71%)), and natural products (such as menthol (**45**, 89%) and citronellol (**46**, 72%)), resulting into densely functionalized and diversely substituted anisidines with exclusive meta-dithiocarbamate linkage (Scheme 2E). Moreover, anisidine linked to d-ribose participated in the planned reaction to provide **47** in 72% yield. Notably, replacing the amine with alcohol, such as 1-butanol, under the optimized conditions led to the formation of the corresponding meta-*S*-aryl xanthate **48** in 67% yield (see Scheme 2F and the Supporting Information for details). However, the desired product was not obtained in the case of sterically encumbered isopropanol and *tert*-butyl alcohol. Needless to mention that, these transformations in a functionally orchestrated and relatively complex environment further reaffirm the versatility and robustness of this meta-selective C–S bond formation methodology.

Pleasingly, this multicomponent meta-C–S bond installation strategy was successfully extended to *N*-protected unsubstituted anilines **49** (Scheme 3). In the case of unsubstituted anilines, 2.2 equiv of PIDA was used to generate the corresponding quinone iminyl ketone intermediate, which, upon reaction with a variety of amines and CS_2_ under standard optimized conditions, provided the corresponding *S*-aryl dithiocarbamates (**4**, **14**, **19**, **25**-**26**, **29**) in moderate to good yields (61%–78%).

**Scheme 3:**
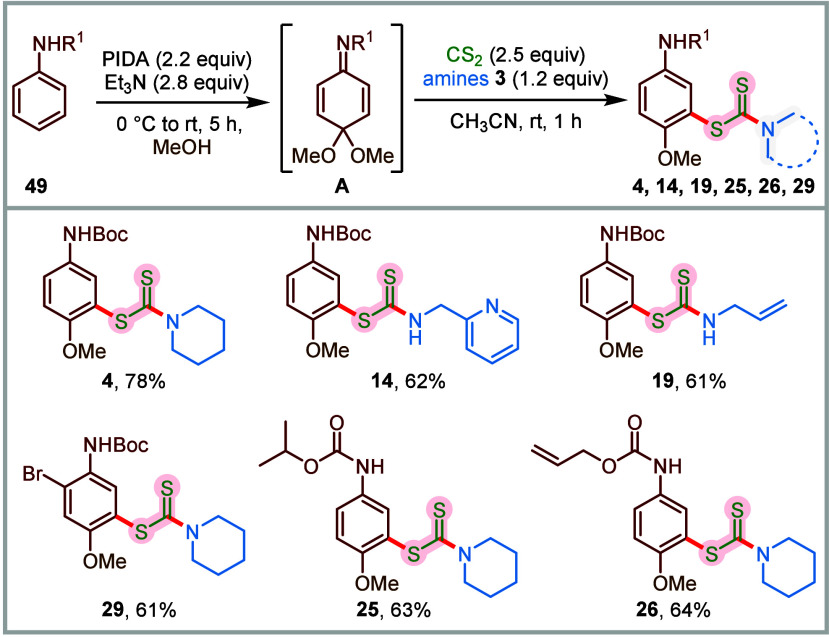
Scope of the Reaction from Anilines

a Reaction conditions: **49** (0.2 mmol), **3** (0.24 mmol), PIDA (0.44 mmol), CS_2_ (0.5 mmol), and CH_3_CN (2 mL) at 0 °C to room temperature for 1 h.

The scalability and industrial applicability of the method were demonstrated by carrying out the model reaction (entry 5, Table 1) with 1.1 g of *N*-Boc *p*-anisidine **1a**, furnishing desired compound **4** in 78% yield (Scheme 4a). Considering the densely functionalized nature of the final meta-dithiocarbamate-substituted anisidines, and the possibility of converting this hitherto unknown class of compounds into further value-added chemicals, we performed a series of postsynthetic modifications. We successfully transformed compounds **4**, **7**, and **10** into corresponding meta-substituted anilines (**50**–**52**) in excellent yields through selective *N*-Boc deprotection using trifluoroacetic acid at room temperature (Scheme 4b–d). Thiol-containing molecules hold significant relevance in biological systems; however, to the best of our knowledge, there are practically no methods for the synthesis of *p*-anisidine-derived thiols. To this end, we achieved the conversion of dithiocarbamate derivative **10** into the corresponding free thiol derivative **53** (86% yield), using an ethanolic solution of sodium hydroxide under heating conditions (Scheme 4e). Inspired by the biological importance of thioethers and sulfones,^3,77^ we dedicated our efforts to convert the final dithiocarbamates into these medicinally relevant functionalities. Accordingly, a copper-catalyzed cross-coupling between **10** and phenyl boronic acid enabled the synthesis of the corresponding diarylsulfide **54** in 71% yield (Scheme 4f). Furthermore, **10** was efficiently converted to the multisubstituted sulfone **55** (68%) by combining the Cu-catalyzed sulfide formation step with a peroxide-mediated oxidation of sulfides to sulfones (Scheme 4g). It is noteworthy that, besides being biologically relevant, the dithiocarbamate moiety also serves as an important building block toward the synthesis of other value-added functional entities, such as thiols, sulfides, and sulfones. While the one-pot multicomponent approach ensures installation of the dithiocarbamate unit at the meta-position in a regioselective manner, while forging the C–S bond, the series of post-synthetic transformations enables access to highly valuable sulfur-containing anisidine derivatives.

**Scheme 4:**
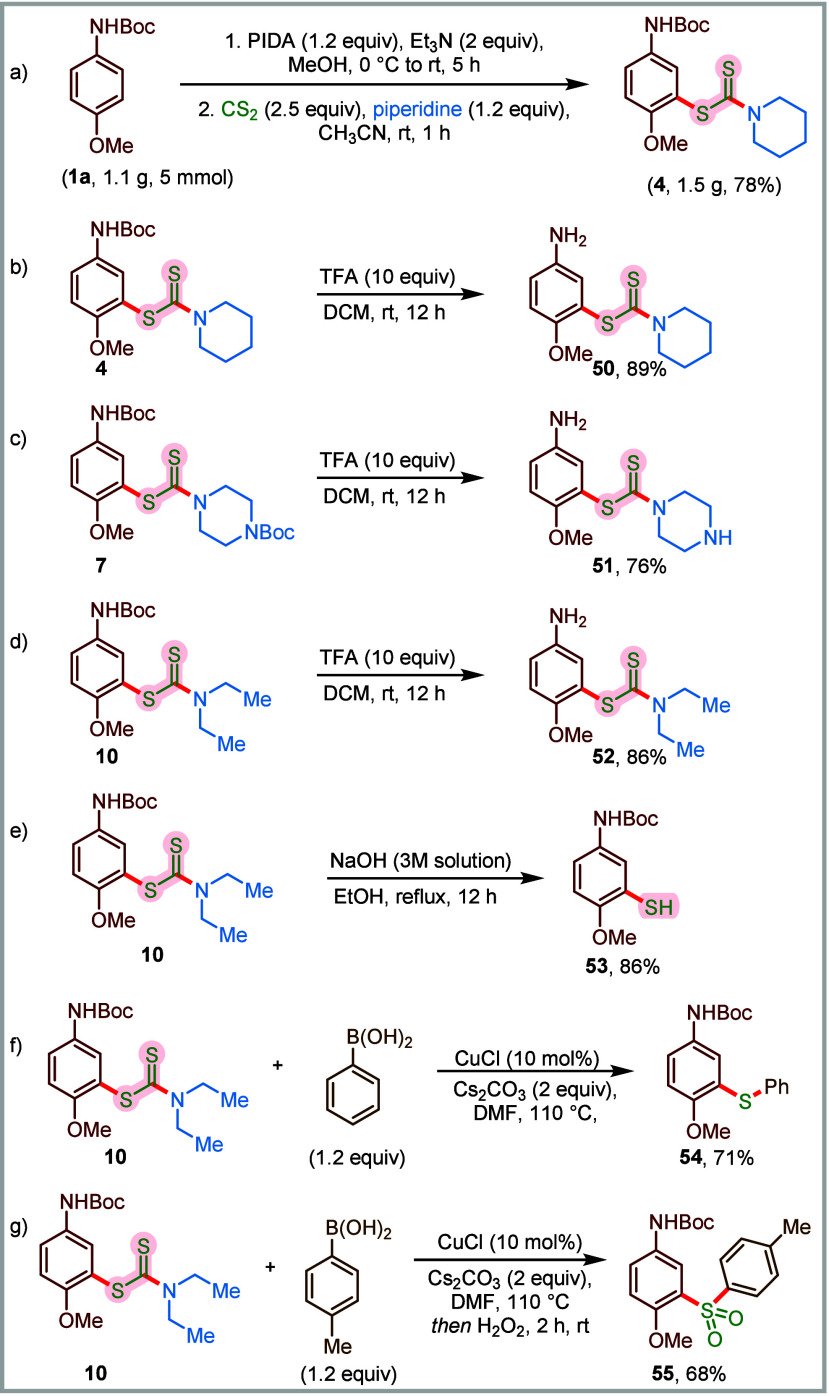
Scale-Up Synthesis and Post-Synthetic Modifications

To gain insight into the reaction mechanism, we performed several control experiments. The intermediacy of QIKs was established by reacting isolated and purified **2** with piperidine and CS_2_ under the optimized conditions to obtain the desired compound **4** in 96% yield (Scheme 5a). We also performed the model reaction in the presence of radical scavengers to evaluate the possibility of a radical mechanism. In the presence of scavengers, such as TEMPO and BHT, the reaction remained unaffected, indicating that a single electron transfer (SET) pathway was not followed in these transformations (Scheme 5b). We propose that the reaction begins with the formation of QIK intermediate **2** through the reaction of **1a** with PIDA (Scheme 6).^78^ On the other hand, the reaction between carbon disulfide and piperidine generates dithiocarbamic acid **56a**. Next, a proton transfer between **2** and **56a**, and subsequent conjugate addition of the resulting dithiocarbamate **I** to protonated **2** leads to the formation of intermediate **III**. Finally, dithiocarbamic acid-assisted rearomatization of intermediate **III** delivers the desired anisidine-based dithiocarbamate **4**.

**Scheme 5:**
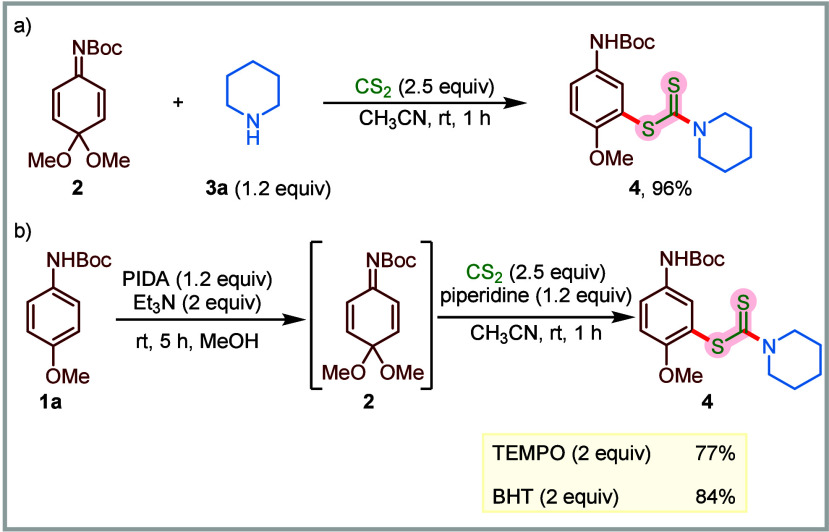
Control Experiments

**Scheme 6:**
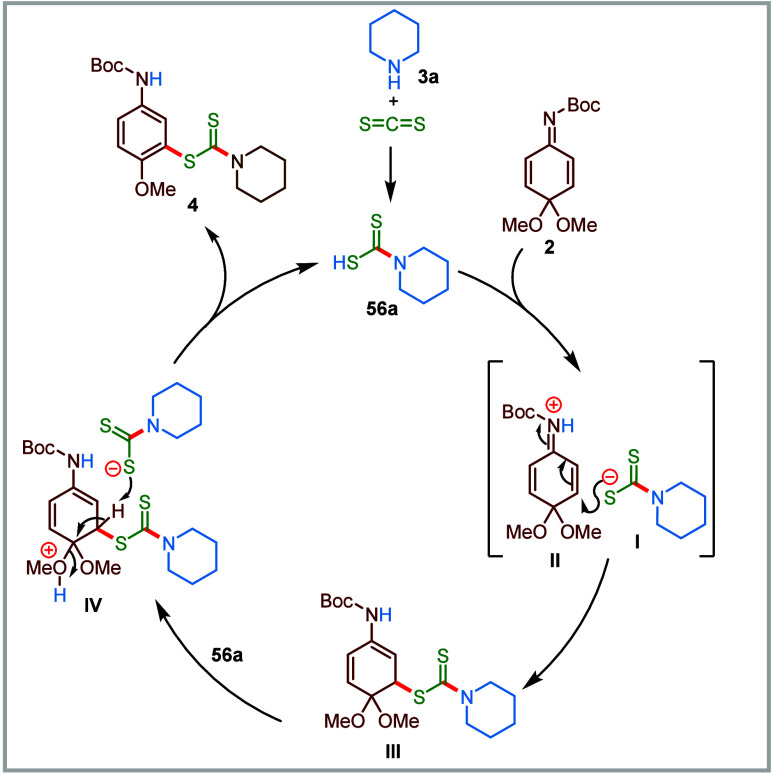
Proposed Mechanism

Breast cancer is a heterogeneous group of neoplasms that can lead to diverse responses to therapies.^79^ Taxanes, including paclitaxel, cabazitaxel, and docetaxel, together with anthracyclines, constitute the first-line therapy for patients with both metastatic and early-stage breast cancer.^80–82^ Paclitaxel functions as a microtubule-stabilizing agent by binding to the β-subunit of tubulin, preventing depolymerization and promoting the formation of stable microtubule structures.^83,84^ Microtubule stabilization leads to the development of abnormal microtubule bundles in dividing cells, which causes cell cycle arrest at the G_2_/M phase and triggers apoptosis in cancer cells.^85^ However, breast cancers are known to develop resistance against paclitaxel,^86,87^ thus suggesting the need to identify novel chemical scaffolds. Our previous studies on the potential of dithiocarbamates as promising anticancer drugs^67,68,76^ led us to identify a *S*-aryl dithiocarbamate-based compound **57** with IC_50_ 2.60 ± 0.30 μM in the breast cancer cell line, MCF-7. Interestingly, compound **57** was found to also affect microtubule dynamics by stabilizing them in the dividing cells. These studies motivated us to explore the antiproliferative activity of the newly synthesized and hitherto unknown anisidine-derived *S*-aryl dithiocarbamates in search of novel chemotypes with improved potency.

To evaluate in vitro cytotoxicity, the MCF-7 breast cancer cells were treated with varying concentrations of respective compounds for 72 h, which was followed by a colorimetric cell viability-based MTT assay. The drug-response curve of the assay was used to calculate the IC_50_ value of these compounds. For the structure–activity relationship analysis, we screened selected compounds from the synthesized library with variations regarding *N*-protecting group, substituents on the arene moiety, O-alkyl functionality, and structurally and electronically diverse amines. Pleasingly, *N*-Boc protected dithiocarbamate **4** emerged as a potent compound with 26-fold higher potency (IC_50_ = 0.10 ± 0.02 μM) as compared to **57** (entry 2, Table 2). Our findings demonstrate that Boc as a nitrogen protecting group was crucial, as the efficacy plummeted to IC_50_ = 8.9 ± 0.19 μM in the case of the corresponding free aniline derivative **50** (entry 3, Table 2). Replacing Boc with less sterically bulky nitrogen protecting groups did not impart any beneficial effect (entries 4–6, Table 2). Next, we studied the role of phenyl substitution pattern on the inhibitory activity and realized that incorporation of either alkyl or aryl substituents on the phenyl ring had an adverse effect, with a 10-fold decrease in the potency (entries 7–9, Table 2). Replacing the −OMe group with longer −OEt (**33**, IC_50_ = 1.09 ± 0.26 μM), −O^*n*^Bu (**34**, IC_50_ = 1.29 ± 0.39 μM), −O^*i*^Bu (**35**, IC_50_ = 3.31 ± 0.09 μM), −O allyl (**37**, IC_50_ = 7.01 ± 0.21 μM), and −O propargyl (**38**, IC_50_ = 3.77 ± 0.23 μM) or with sterically encumbered −O^*i*^Pr (**36**, IC_50_ = 0.62 ± 0.04 μM) provided inferior results (entries 10–15, Table 2). Our subsequent SAR studies were focused on the variation of a diverse set of amines. While a similar level of antiproliferative activity was observed with *N*-Boc-protected piperazine (**7**, IC_50_ = 0.12 ± 0.02 μM) instead of piperidine, a sharp reduction in potency happened with the corresponding unprotected piperazine derivative **51** (IC_50_ = 2.57 ± 0.18 μM) (entries 16 and 17, Table 2). Accordingly, we varied the protecting group of piperazine and integrated pharmaceuticals, such as ibuprofen and naproxen, by removing the Boc group. Unfortunately, the efficacy was much lower in both cases, compared to the Boc-protected piperazine derivative (entries 18 and 19, Table 2). Moreover, no improvement in the potency was noted with other cyclic tertiary amines, such as tetrahydroisoquinoline (**8**, IC_50_ = 0.19 ± 0.06 μM) and proline (**41**, IC_50_ = 0.15 ± 0.05 μM), and the corresponding acyclic variant, such as *N*-methyl benzylamine (**12**, IC_50_ = 0.15 ± 0.02 μM). However, with acyclic secondary amines, such as benzylamine (**13**, IC_50_ = 0.13 ± 0.01 μM) and 2-thiophenemethylamine (**16**, IC_50_ = 0.12 ± 0.02 μM), a potency comparable to piperidine (compound **4**) and *N*-Boc protected piperazine (compound **7**) was observed.

**Table 2:**
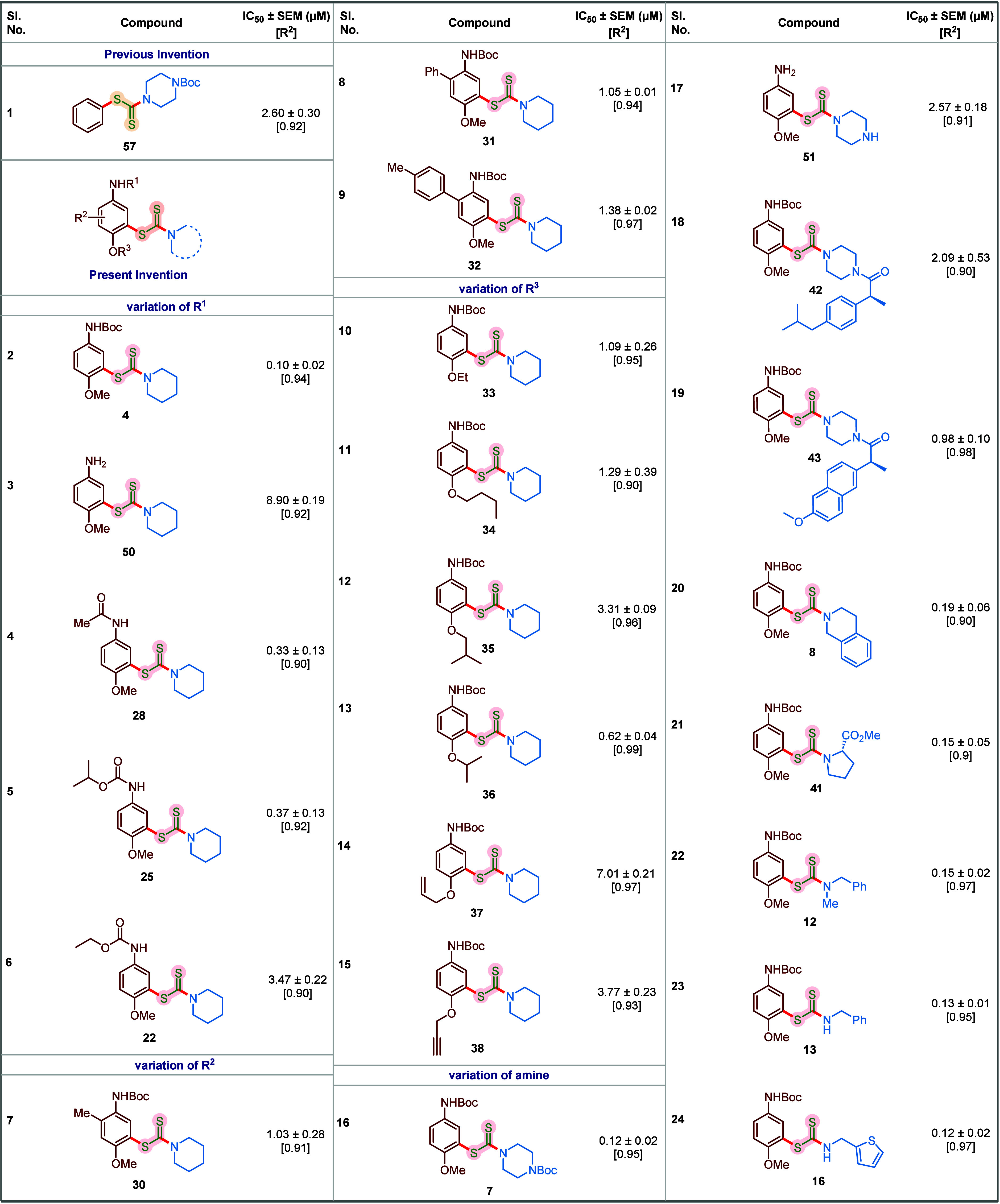
SAR Studies of Selected *S*-aryl Dithiocarbamates Based on Their Antiproliferative Activities in the MTT Assay

a IC_50_ values were calculated from two independent experimental measurements. The values are reported as the average IC_50_ ± standard error of mean.

Since the hit compound **57** from previous work docked in the Taxol-binding pocket of the β-tubulin subunit of α/β tubulin heterodimer, we also analyzed the docking efficiency of the selected best compounds (**4**, **7**, **12**, **13**, and **16**) from our SAR studies using the receptor tubulin (PDB ID: 6I2I). Among these compounds, only **7** and **13** were found to dock in the Taxol-binding pocket of β-tubulin with the atomic contact energies of −6.5 and −7.3 kcal/mol, respectively. The contact map analysis (Figure 1A) revealed that paclitaxel interacts with Histidine 229, Arginine 278, Glutamine 281, and Threonine 276 in the binding pocket of β-tubulin,^88^ and in the case of **7**, the NBoc group on the piperazine was found to be involved in hydrogen bonding (2.9–3 Å) with Threonine 276. However, in the case of compound **13**, besides hydrogen bonding with Threonine 276 (2.9 Å), the NHBoc group on the arene ring was also found to interact with Proline 274 (2.7 Å). Besides more polar interactions compared to compound **7**, compound **13** also possessed better hydrophobic interactions with the Taxol-binding pocket, as inferred from the corresponding binding energies.

**Figure 1:**
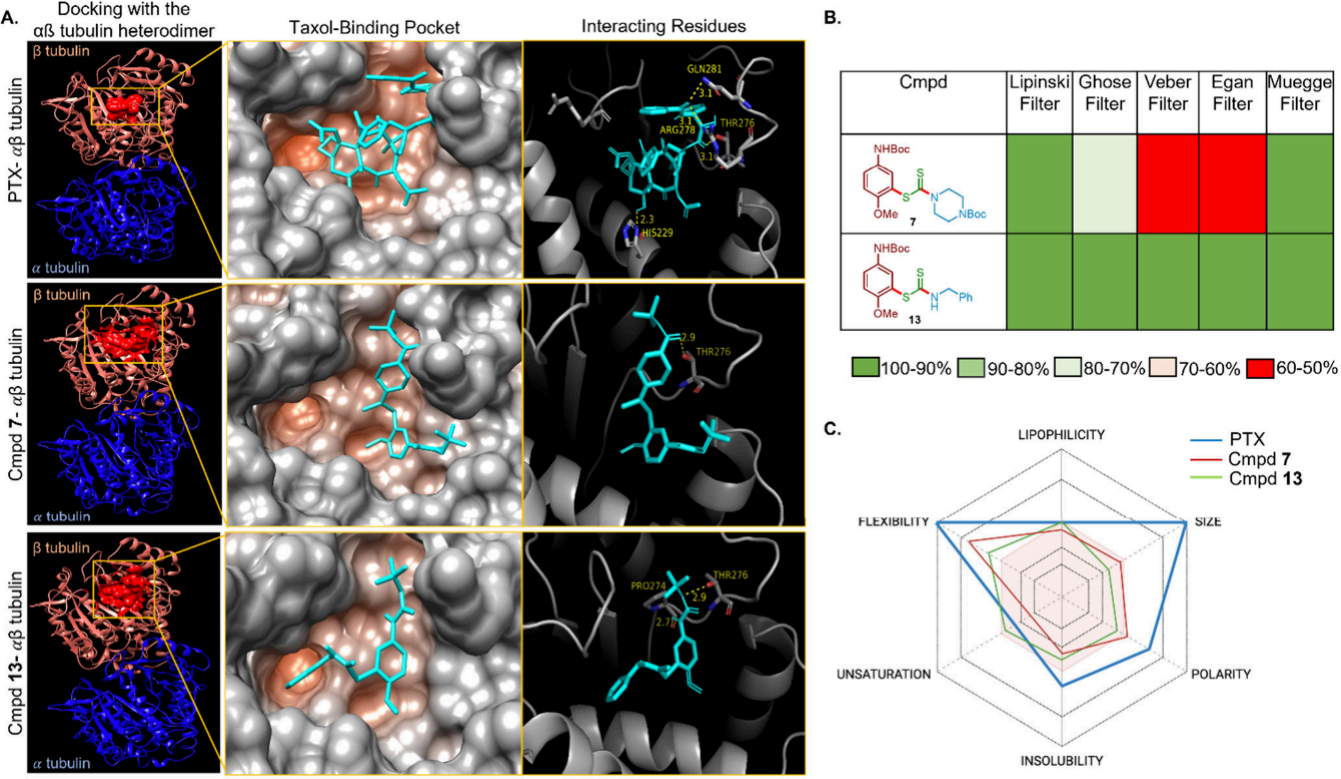
(A) Docking of paclitaxel (PTX), compounds **7** and **13** with the receptor α,β-tubulin (PDB ID: 6I2I) using a SwissDock server. Representative images of the docking site, binding pocket, and hydrogen bonds formed with interacting residues, along with bond lengths depicted for all compounds. The distances between interacting residues have been filtered to highlight only those under 3.5Å, indicating potential hydrogen bonding. (B) Color-coded heat map showing a drug-like filter compliance for compounds **7** and **13**. (C) Bioavailability radar plot generated using SwissADME, showing the physicochemical properties for paclitaxel (PTX, positive control), as well as compounds **7** and **13**. The red-colored region depicts the suitable physicochemical space for oral bioavailability. Lipophilicity: −0.7 < XLOGp3 < +5.0; size: 150 g/mol < MV < 500 g/mol; polarity: 20 A^2^ < TPSA < 130 A^2^; insolubility: −6 < log *S* (ESOL) < 0; unsaturation: 0.25 < fraction C sp^3^ < 1; flexibility: 0 < number rotatable bonds < 9.

Next, we performed in silico studies to evaluate the drug-like properties and lipophilicity of compounds **7** and **13** (Figure 1B). The analysis exhibited that compound **13** offers better drug-like properties, satisfying all drug-like filters with 100% efficiency, while showing improved oral bioavailability (Figure 1C). The in-silico ADMET analysis of compound **13** also indicated that it exhibits comparable or superior absorption and less toxicity profiles, relative to the commercial drug paclitaxel (Figure S1, Table S1). These findings collectively established compound **13** as a promising candidate for further cellular studies to evaluate its effect on microtubule dynamics. Notably, compounds **7** and **13** showed IC_50_ of 3.47 ± 0.3 μM and 1.65 ± 0.2 μM, respectively, in the noncancer cell line, 3T3L-1 (see Table S2). Hence, both compounds exhibited significantly higher cytotoxicity toward breast cancer cells than noncancerous cells, indicating their selectivity for cancerous cells. Moreover, comparison of the IC_50_ value of lead compound **13** in different cancer types like MCF7 (0.13 ± 0.01 μM), HeLa (1.00 ± 0.24 μM), and A549 (3.60 ± 0.30 μM), as indicated in Table S3, demonstrates better potency for the lead compound in the breast cancer cells.

To assess microtubule arrangement in cells, we employed fluorescence. Both compounds **7** and **13**, which dock in the Taxol-binding pocket of tubulin, induced microtubule bundling, much like paclitaxel-treated cells (Figures 2A and 2B). In contrast, compound **12**, used as a negative control due to its inability to dock in the Taxol-binding pocket, showed no effect on microtubule dynamics, further validating the docking analysis. The tubulin fluorescence signal in Figure 2A was quantified to reveal an average i-skewness in Figure 2B, which is an indicator of the tubulin fluorescence signal distribution in the cell. To exclude the indirect effect of compound treatment on microtubule dynamics, we utilized goat-brain purified microtubules, which were treated with either vehicle (control), paclitaxel (positive control), or compound **7**, **13**, or **57**. The formation of filamentous microtubule structures indicative of stabilization was observed in both positive control and compound **7**, **13**, or **57**-treated samples (Figure 2C), confirming a direct effect of these compounds on microtubule dynamics.

**Figure 2:**
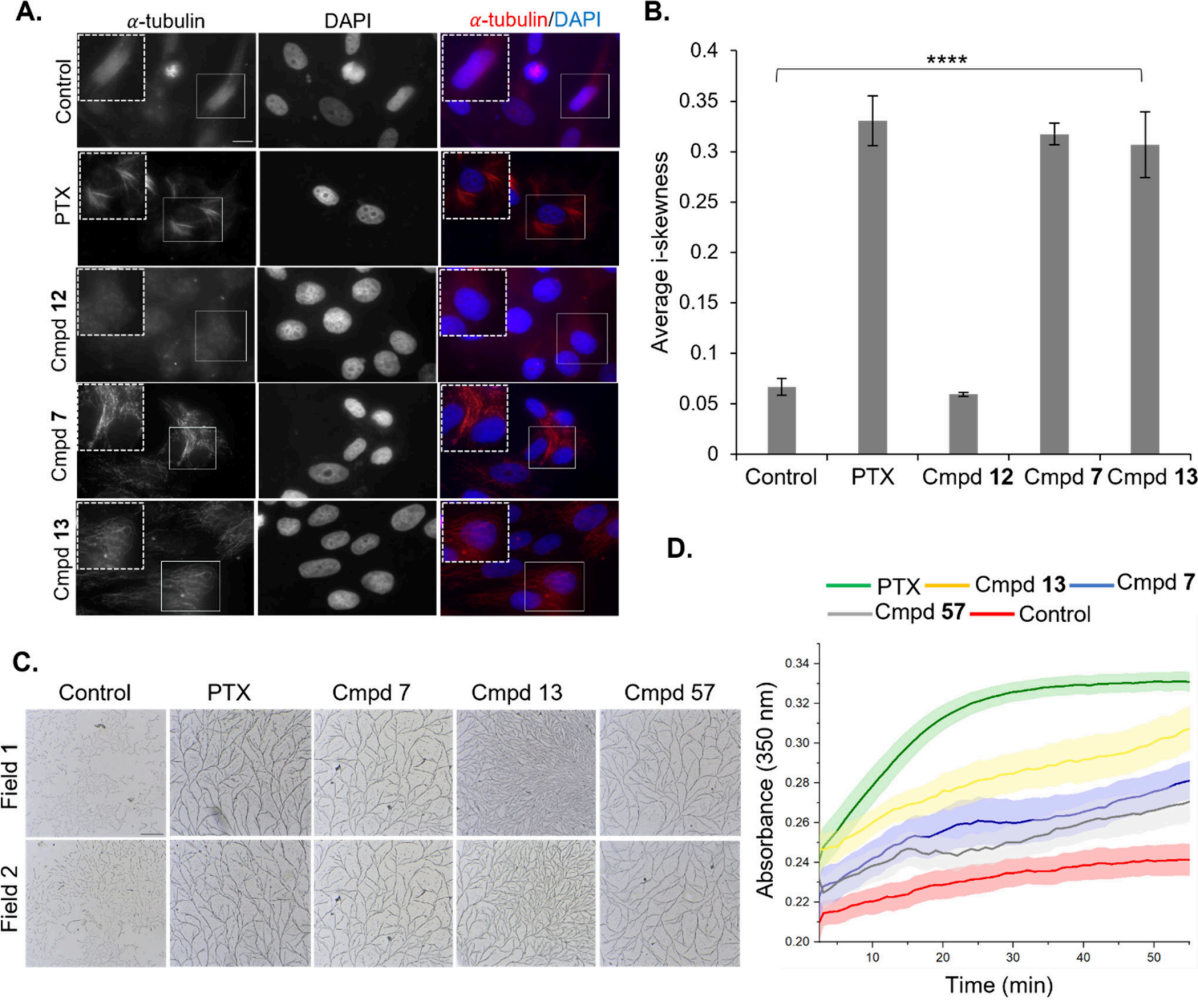
(A) Representative fluorescence images of MCF7 cells treated with 0.5 μm concentration of either paclitaxel (PTX, positive control), compound **12** (negative control), **7**, or **13** for 24 h. Cells immunostained for α-tubulin and DAPI (nuclear staining). Last panel shows both channels merge. Scale bar = 5 μm. (B) Bar graph representing average i-skewness ± SEM of tubulin signal in the experiment described in panel (A) for the stated conditions. [Legend: (****) *p* < 0.0001 (two-tailed unpaired Student’s *t*-test).] (C) Bright-field images for two fields of view (fields 1 and 2) of purified goat-brain tubulin treated with 10 μM of PTX (positive control), compound **7**, **13**, or **57** for 30 min. DMSO (vehicle)-treatment was used as a negative control. Scale bar = 10 μm. (D) Line plot indication microtubule polymerization rate for control (red), compound **13** (yellow), compound **7** (blue), compound **57** (gray), and PTX (green), using the turbidity assay at 350 nm. Error bars as shaded regions represent the standard error of mean.

The rate of microtubule polymerization monitored by an increase in turbidity of purified bovine tubulin solution at 350 nm with time suggested a slow and steady polymerization rate for conditions treated with compounds **7**,**13**, and **57**, when compared to paclitaxel. Interestingly, compound **13**, exhibiting better interaction compared to compounds **7** or **57** in the docking assay, also performed better in this assay (Figure 2D). Drugs targeting microtubule dynamics causes cell cycle arrest. Similarly, we also found that cells treated with compound **13** were arrested in the G_2_/M stage of the cell as shown by flow cytometry analysis (Figures S2A and S2B). Accordingly, we found enhanced levels of G_2_/M cell cycle-specific cyclin B1 protein and activated levels of cell cycle checkpoint p53 (phosphorylated) in cells treated with compound **13**, compared to vehicle control cells (see Figure S2C). These results establish compound **13** as the lead compound within this series, demonstrating superior functionality as a microtubule-polymerizing agent and effective potency in the MCF-7 breast cancer cell line.

## Conclusion

To summarize, we have developed a multicomponent reaction between *N*-protected *p*-anisidines/anilines, carbon disulfide, and aliphatic amines to install an otherwise difficult meta-C–S bond on anilines, while furnishing an array of highly substituted anisidine-derived *S*-aryl dithiocarbamates. Notably, the method does not require prefunctionalized starting materials, a metal catalyst, a direct group, or an additive, and operates under environmentally benign conditions. The domino process offers significant advantages, including scalability, cost efficiency, step and atom economy, making it both sustainable and highly practical. The site-selective meta-thiolation strategy was successfully applied to the late-stage modification of a variety of amino acids, pharmaceuticals, natural products, and sugar compounds, demonstrating its remarkable robustness and functional group tolerance. Importantly, final *S*-aryl dithiocarbamates could be converted to highly valuable and medicinally relevant sulfur functional moieties, such as thiols, thioethers, and sulfones. Furthermore, both in silico analyses and cellular studies confirm the drug-like properties of the synthesized *S*-aryl dithiocarbamates, underscoring their potential in pharmaceutical applications. The screening of newly synthesized dithiocarbamate molecules provided us with a promising lead compound with better efficacy in breast cancer cells, compared to our previously reported compound. We could show a direct effect of the lead compound on microtubule dynamics using the purified tubulin, thus providing a promising lead molecule for pharmaceutical applications.

## Materials and Methods

The Supporting Information includes all information about the materials and methods used in this study.

## Supplementary Material



## Data Availability

The data underlying this study is available in the published article and its Supporting Information.

## Notes

Experimental details, additional optimization studies, copies of ^1^H and ^13^C NMR spectra for all compounds; biology experimental details, ADMET analysis, and cell cycle profile by flow cytometry analysis (PDF)
