# Supplementary material for: Metal and Additive-Free Nondirected Meta-C–S Bond Formation on Anilines: Toward Biologically Relevant S‑Aryl Dithiocarbamates
Source: ACS Cent Sci. 2025 Sep 15;11(11):2121–32. doi: 10.1021/acscentsci.5c01231 (PMC12670321; doi:10.1021/acscentsci.5c01231)

# Supporting Information

## Metal and Additive-Free Nondirected Meta-C-S bond Formation on Anilines: Towards Biologically Relevant *S*-aryl dithiocarbamates

*Sushanta Kumar Parida,[a] Srishti Sanghi,[b] Ardhendu Mondal,[a] Nameeta Choudhary,[b] Prahallad Meher,[a] Priyanka Singh,*[b] Sandip Murarka*[a]*

[a]Department of Chemistry, Indian Institute of Technology Jodhpur, Karwar-342037, Rajasthan, India. Email: sandipmurarka@iitj.ac.in.

[b]Department of Bioscience and Bioengineering, Indian Institute of Technology Jodhpur, Karwar-342037, Rajasthan, India. Email: priyankasingh@iitj.ac.in.

## Table of Contents

| | | |
|---|---|---|
| 1 | General Information | S2 |
| 2 | Preparation of Starting Materials | S3 |
| 3 | General procedure (GP-1) for the synthesis of S-aryl dithiocarbamates **4-32** and **39-48** | S3 |
| 4 | General procedure (GP-2) for the synthesis of S-aryl dithiocarbamates **33-38** | S4 |
| 5 | General procedure (GP-3) for the synthesis of S-aryl dithiocarbamates **4, 14, 19, 25, 26, 29** | S4 |
| 6 | Characterization data | S5 |
| 7 | Gram scale synthesis of **4** and large-scale synthesis of **35** | S19 |
| 8 | Control Experiment | S20 |
| 9 | Post-synthetic Modification | S21 |
| 10 | Single Crystal X-ray data of the compound **4** | S24 |
| 11 | Unsuccessful Substrates | S26 |
| 12 | Biological material and methods | S26 |
| 13 | ADMET analysis | S30 |
| 14 | Comparison of $IC_{50}$ value in cancer and non-cancer cell | S34 |
| 15 | Cell cycle profile | S35 |
| 16 | References | S35 |
| 17 | NMR spectra | S36 |

## 1. General Information

All the reactions were performed using pre-dried glassware and screw-cap vials. All the solvents were obtained from Merck (Emparta grade) and used without further drying or fresh distillation. All the amines (primary, secondary, cyclic, and acyclic), Aniline, p-anisidine, diacetoxyiodobenzene (PIDA), triethylamine, and carbon disulphide were obtained from commercial sources and used without further purification. The corresponding p-anisidine/aniline derivatives were synthesized using the following procedure. The reported yields are of isolated compounds that are estimated to be >95% pure as determined by $^{1}$H NMR, $^{13}$C NMR, HRMS and single-crystal NMR. Thin layer chromatography (TLC) was performed on Merck pre-coated silica gel 60 $F_{254}$ aluminium sheets with detection under UV light at 254 nm. Chromatographic separations were carried out on Avra silica gel (100-200 mesh or 230−400 mesh). Nuclear magnetic resonance (NMR) spectroscopy was performed using Bruker 400 and 500 MHz spectrometers. If not otherwise specified, chemical shifts (δ) are provided in ppm. HRMS spectra were recorded by electron spray ionization (ESI) method on a Agilent Q-TOF Micro with lock spray source. Single crystal X-ray diffractions were recorded using Bruker AXS Smart Apex CCD diffractometer.

# 2. Preparation of Starting Materials:

The following cyclic amines were prepared as per the procedure in the literature.[1]

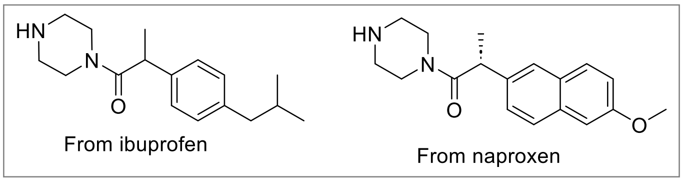


All the *N*-substituted *p*-anisidines were prepared using known literature procedures.[2,3]

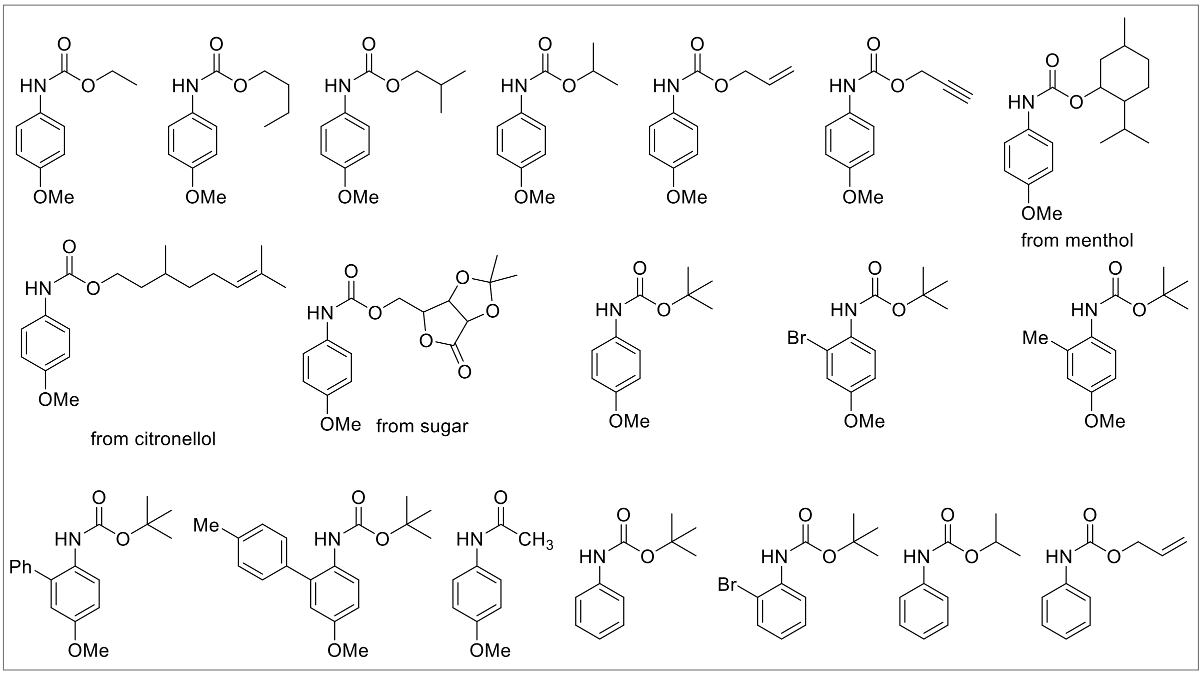


# 3. General procedure (GP-1) for the synthesis of *S*-aryl dithiocarbamates (4-32 and 39-48)

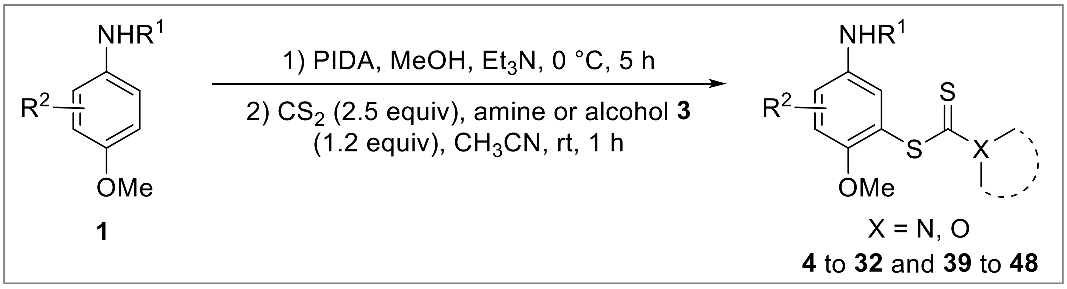


To a stirred solution of **1** (1.0 equiv, 0.2 mmol) in methanol (1.0 mL) was added (diacetoxyiodo)benzene (PIDA, 77.3 mg, 1.2 equiv) and $Et_3N$ (55.7 µL, 2.0 equiv) at 0 °C. The reaction mixture was stirred at 0 °C for 1 h and gradually warmed to rt. After complete consumption of **1** (monitored by TLC), MeOH was evaporated on a rotatory evaporator, and the residue was dissolved in acetonitrile (2.0 mL). To this solution, the carbon disulfide (2.5 equiv, 0.5 mmol, 37.1 mg) and corresponding amine or alcohol **3** (1.2 equiv, 0.24 mmol) were added, and the reaction mixture was stirred for 1 h at room temperature under an $N_2$

atmosphere. After completion of the reaction, acetonitrile was evaporated in vacuo, and the residue was purified by column chromatography using a gradient of hexane/ethyl acetate to afford the corresponding *S*-aryl dithiocarbamate products (**4-32**, **39-48**) in good to excellent yields.

## 4. General procedure (GP-2) for the synthesis of *S*-aryl dithiocarbamates (33-38)

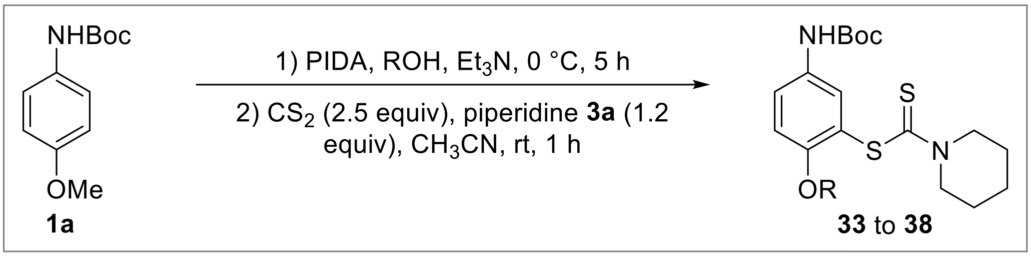


To a stirred solution of **1a** (1.0 equiv, 0.2 mmol) in corresponding alcohol (1.0 mL) was added (diacetoxyiodo)benzene (PIDA, 77.3 mg, 1.2 equiv) and $Et_3N$ (55.7 µL, 2.0 equiv) at 0 °C. The reaction mixture was stirred at 0 °C for 1 h and gradually warmed to rt. After complete consumption of **1a** (monitored by TLC), the corresponding alcohol was evaporated on a rotatory evaporator, and the residue was dissolved in acetonitrile (2.0 mL). To this solution, the carbon disulfide (2.5 equiv, 0.5 mmol, 37.1 mg) and piperidine **3a** (1.2 equiv, 0.24 mmol) were added, and the reaction mixture was stirred for 1 h at room temperature under an $N_2$ atmosphere. After completion of the reaction, acetonitrile was evaporated in vacuo, and the residue was purified by column chromatography using a gradient of hexane/ethyl acetate to afford the corresponding *S*-aryl dithiocarbamates products (**33-38**) in good to excellent yields.

## 5. General procedure (GP-3) for the synthesis of *S*-aryl dithiocarbamates (4, 14, 19, 25, 26, 29)

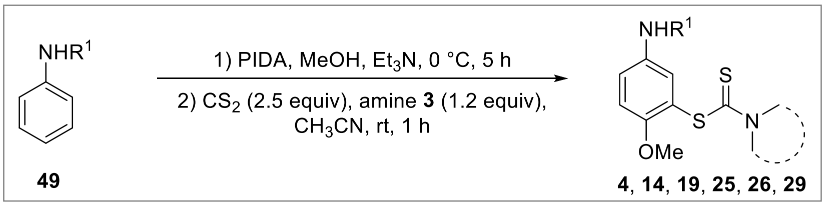


To a stirred solution of **49** (1.0 equiv, 0.2 mmol) in methanol (1.0 mL) was added (diacetoxyiodo)benzene (PIDA, 141.7 mg, 2.2 equiv) and $Et_3N$ (78 µL, 2.8 equiv) at 0 °C. The reaction mixture was stirred at 0 °C for 1 h and gradually warmed to rt. After complete consumption of **49** (monitored by TLC), methanol was evaporated on a rotatory evaporator, and the residue was dissolved in acetonitrile (2.0 mL). To this solution, carbon disulfide (2.5 equiv, 0.5 mmol, 37.1 mg) and corresponding amine **3** (1.2 equiv, 0.24 mmol) were added, and the reaction mixture was stirred for 1 h at rt under $N_2$ atmosphere. After completion of the reaction, acetonitrile was evaporated in *vacuo*, and the residue was purified by column

chromatography using a gradient of hexane/ethyl acetate to afford the corresponding *S*-aryl dithiocarbamates products (**4, 14, 19, 25, 26, 29**) in good to excellent yield.

## 6. Characterization data

### 5-((*tert*-Butoxycarbonyl)amino)-2-methoxyphenyl piperidine-1-carbodithioate (4)

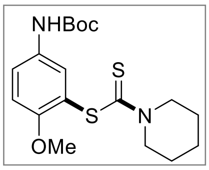


The compound was prepared according to **GP1/GP3** using piperidine (0.020 g, 0.24 mmol), $CS_2$ (0.037 g, 0.5 mmol) and *tert*-butyl (4-methoxyphenyl)carbamate (0.044 g, 0.2 mmol), Purification by column chromatography (15% ethyl acetate in hexane) gave **4** as a white solid (0.069 g, 91%). **$^1$H NMR (500 MHz, $CDCl_3$)** δ 7.55 – 7.39 (m, 2H), 6.92 (d, *J* = 8.9 Hz, 1H), 6.41 (s, 1H), 4.25 (s, 2H), 4.01 (s, 2H), 3.83 (s, 3H), 1.72 (s, 6H), 1.49 (s, 9H). **$^{13}$C NMR (126 MHz, $CDCl_3$)** δ 195.0, 156.7, 152.9, 131.7, 129.4, 123.0, 120.1, 112.2, 80.4, 56.6, 53.2, 52.1, 28.4, 26.2, 25.4, 24.2. **HRMS-ESI** (m/z): calcd for $C_{18}H_{27}N_2O_3S_2$ [M + H ]$^+$ 383.1458; found 383.1464.

### 5-((*tert*-Butoxycarbonyl)amino)-2-methoxyphenyl pyrrolidine-1-carbodithioate (5)

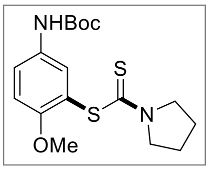


The compound was prepared according to **GP1** using pyrrolidine (0.017 g, 0.24 mmol), $CS_2$ (0.037 g, 0.5 mmol) and *tert*-butyl (4-methoxyphenyl)carbamate (0.044 g, 0.2 mmol). Purification by column chromatography (15% ethyl acetate in hexane) gave **5** as a yellow oil (0.065 g, 89%). **$^1$H NMR (500 MHz, $CDCl_3$)** δ 7.54 – 7.37 (m, 2H), 6.93 (d, *J* = 8.9 Hz, 1H), 6.43 (s, 1H), 3.90 (t, *J* = 7.0 Hz, 2H), 3.83 (s, 3H), 3.81 (t, *J* = 7.0 Hz, 2H), 2.11 (p, *J* = 6.9 Hz, 2H), 1.98 (p, *J* = 6.9 Hz, 2H), 1.49 (s, 9H). **$^{13}$C NMR (126 MHz, $CDCl_3$)** δ 192.1, 156.8, 153.0, 131.8, 129.7, 123.3, 119.6, 112.4, 80.5, 56.7, 55.4, 51.2, 28.5, 26.5, 24.5. **HRMS-ESI** (m/z): calcd for $C_{17}H_{25}N_2O_3S_2$ [M + H ]$^+$ 369.1301; found 369.1306.

### 5-((*tert*-Butoxycarbonyl)amino)-2-methoxyphenyl morpholine-4-carbodithioate (6)

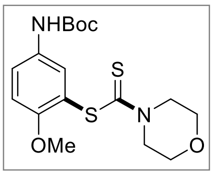


The compound was prepared according to **GP1** using morpholine (0.020 g, 0.24 mmol), $CS_2$ (0.037 g, 0.5 mmol) and *tert*-butyl (4-methoxyphenyl)carbamate (0.044 g, 0.2 mmol). Purification by column chromatography (15% ethyl acetate in hexane) gave **6** as a yellow oil (0.069 g, 91%). **$^1$H NMR (500 MHz, $CDCl_3$)** δ 7.52 – 7.43 (m, 2H), 6.92 (d, *J* = 8.8 Hz, 1H), 6.43 (s, 1H), 4.38 – 4.00 (m, 4H), 3.83 (s, 3H), 3.81 – 3.78 (m, 4H), 1.50 (s, 9H). **$^{13}$C NMR (126 MHz, $CDCl_3$)** δ 197.0, 156.7, 153.0, 131.9, 129.4, 123.3, 119.4, 112.3, 80.6, 66.4, 56.7, 51.4, 28.5. **HRMS-ESI** (m/z): calcd for $C_{17}H_{25}N_2O_4S_2$ [M + H]$^+$ 385.1250; found 385.1241.

**tert-Butyl 4-(((5-((tert-butoxycarbonyl)amino)-2-methoxyphenyl)thio)carbonothioyl)piperazine-1-carboxylate (7)**

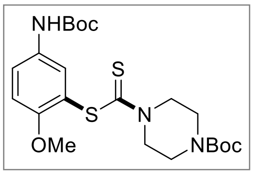


The compound was prepared according to **GP1** using *N*-boc-piperazine (0.044 g, 0.3 mmol), $CS_2$ (0.037 g, 0.625 mmol) and *tert*-butyl (4-methoxyphenyl)carbamate (0.044 g, 0.2 mmol). Purification by column chromatography (15-20% ethyl acetate in hexane) gave **7** as a yellow oil (0.075 g, 78%). **$^1$H NMR (500 MHz, $CDCl_3$)** δ 7.55 – 7.38 (m, 2H), 6.91 (d, *J* = 8.8 Hz, 1H), 6.48 (s, 1H), 4.34 – 3.98 (m, 4H), 3.81 (s, 3H), 3.58 (s, 4H), 1.48 (two s, 18H). **$^{13}$C NMR (126 MHz, $CDCl_3$)** δ 196.9, 156.6, 154.6, 153.0, 131.9, 129.2, 123.3, 119.4, 112.3, 80.7 (2C), 56.7, 51.0, 50.6, 43.3, 42.7, 28.5, 28.4. **HRMS-ESI** (m/z): calcd for $C_{22}H_{33}N_3O_5S_2$ $[M + H]^+$ 484.1934; found 484.1939.

**5-((tert-Butoxycarbonyl)amino)-2-methoxyphenyl 3,4-dihydroisoquinoline-2(1*H*)-carbodithioate (8)**

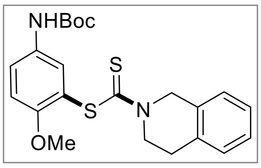


The compound was prepared according to **GP1** using tetrahydroisoquinoline (0.031 g, 0.24 mmol), $CS_2$ (0.037 g, 0.5 mmol) and *tert*-butyl (4-methoxyphenyl)carbamate (0.044 g, 0.2 mmol). Purification by column chromatography (15-20% ethyl acetate in hexane) gave **8** as a yellow oil (0.070 g, 82%). **$^1$H NMR (500 MHz, $CDCl_3$)** δ 7.57 – 7.40 (m, 2H), 7.26 – 7.16 (m, 4H), 6.94 (d, *J* = 8.9 Hz, 1H), 6.41 (s, 1H), 5.30 and 5.16 (two br s, 2H), 4.42 and 4.22 (two br s, 2H), 3.83 (s, 3H), 3.06 and 3.00 (two br s, 2H), 1.50 (s, 9H). **$^{13}$C NMR (126 MHz, $CDCl_3$)** δ 196.0, 156.8, 153.0, 134.4, 131.9, 129.4, 128.4, 127.8, 127.2, 127.0, 123.3, 119.5, 112.3 (2C), 80.6, 54.2, 52.1, 50.3, 48.7, 29.4, 28.5. **HRMS-ESI** (m/z): calcd for $C_{22}H_{27}N_2O_3S_2$ $[M + H]^+$ 431.1458; found 431.1452.

**5-((tert-Butoxycarbonyl)amino)-2-methoxyphenyl azepane-1-carbodithioate (9)**

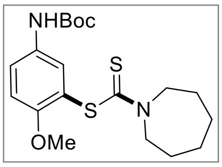


The compound was prepared according to **GP1** using azepine (0.022 g, 0.24 mmol), $CS_2$ (0.037 g, 0.5 mmol) and *tert*-butyl (4-methoxyphenyl)carbamate (0.044 g, 0.2 mmol). Purification by column chromatography (15-20% ethyl acetate in hexane) gave **9** as a yellow oil (0.68 g, 86%). **$^1$H NMR (500 MHz, $CDCl_3$)** δ 7.57 – 7.36 (m, 2H), 6.91 (d, *J* = 8.7 Hz, 1H), 6.42 (s, 1H), 4.21 – 4.13 (m, 2H), 4.07 – 3.98 (m, 2H), 3.82 (s, 3H), 1.95 (br s, 2H), 1.86 (br s, 2H), 1.63 (br s, 4H), 1.49 (s, 9H). **$^{13}$C NMR (126 MHz, $CDCl_3$)** δ 195.8, 156.7, 153.0, 131.8, 129.4, 123.1, 120.3, 112.3, 80.5, 56.7, 56.1, 53.6, 28.4, 27.7, 26.8, 26.7, 26.3. **HRMS-ESI** (m/z): calcd for $C_{19}H_{29}N_2O_3S_2$ $[M + H]^+$ 397.1620; found 397.1611.

***tert*-Butyl (3-((diethylcarbamothioyl)thio)-4-methoxyphenyl)carbamate (10)**

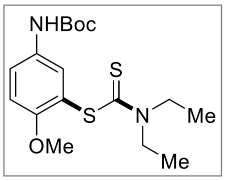


The compound was prepared according to **GP1** using diethylamine (0.018 g, 0.24 mmol), $CS_2$ (0.037 g, 0.5 mmol) and *tert*-butyl (4-methoxyphenyl)carbamate (0.044 g, 0.2 mmol). Purification by column chromatography (15-20% ethyl acetate in hexane) gave **10** as a yellow oil (0.060 g, 81%). **$^1$H NMR (500 MHz, $CDCl_3$)** δ 7.49 (d, *J* = 7.0 Hz, 1H), 7.42 (s, 1H), 6.91 (d, *J* = 8.9 Hz, 1H), 6.42 (s, 1H), 4.01 (dd, *J* = 13.2, 6.3 Hz, 2H), 3.87 (dd, *J* = 13.3, 6.5 Hz, 2H), 3.82 (s, 3H), 1.49 (s, 9H), 1.40 (t, *J* = 6.8 Hz, 3H), 1.27 (t, *J* = 6.8 Hz, 3H). **$^{13}$C NMR (126 MHz, $CDCl_3$)** δ 195.1, 156.7, 153.0, 131.7, 129.4, 123.1, 120.3, 112.4, 80.5, 56.7, 49.9, 47.5, 28.5, 12.9, 11.7. **HRMS-ESI** (m/z): calcd for $C_{17}H_{27}N_2O_3S_2$ $[M + H]^+$ 371.1463; found 371.1453.

***tert*-Butyl (3-((dibutylcarbamothioyl)thio)-4-methoxyphenyl)carbamate (11)**

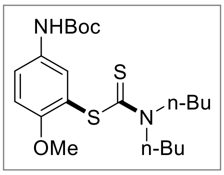


The compound was prepared according to **GP1** using dibutylamine (0.031 g, 0.24 mmol), $CS_2$ (0.037 g, 0.5 mmol) and *tert*-butyl (4-methoxyphenyl)carbamate (0.044 g, 0.2 mmol). Purification by column chromatography (15-20% ethyl acetate in hexane) gave **11** as a yellow oil (0.068 g, 80%). **$^1$H NMR (500 MHz, $CDCl_3$)** δ 7.50 – 7.38 (m, 2H), 6.88 (d, *J* = 8.8 Hz, 1H), 6.52 (s, 1H), 3.95 – 3.89 (m, 2H), 3.79 (s, 3H), 3.76 (s, 2H), 1.80 (s, 2H), 1.70 (s, 2H), 1.48 (s, 9H), 1.43 – 1.38 (m, 2H), 1.37 – 1.29 (m, 2H), 0.99 (t, *J* = 6.9 Hz, 3H), 0.92 (t, *J* = 6.8 Hz, 3H). **$^{13}$C NMR (126 MHz, $CDCl_3$)** δ 195.3, 156.5, 153.0, 131.7, 129.3, 122.9, 120.4, 112.3, 80.3, 56.6, 55.3, 53.2, 29.7, 28.5, 28.4, 20.1 (2C), 13.9, 13.8. **HRMS-ESI** (m/z): calcd for $C_{21}H_{35}N_2O_3S_2$ $[M + H]^+$ 427.2084; found 427.2089.

***tert*-Butyl (3-((dibutylcarbamothioyl)thio)-4-methoxyphenyl)carbamate (12)**

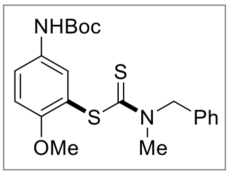


The compound was prepared according to **GP1** using *N*-methylbenzylamine (0.029 g, 0.24 mmol), $CS_2$ (0.037 g, 0.5 mmol) and *tert*-butyl (4-methoxyphenyl)carbamate (0.044 g, 0.2 mmol). Purification by column chromatography (15-20% ethyl acetate in hexane) gave **12** as a yellow oil (0.066 g, 80%). The product was characterized as a mixture of rotamers with 1.40:1 ratio at 298K. **$^1$H NMR (500 MHz, $CDCl_3$)** δ 7.57 – 7.43 (m, 2H), 7.40 – 7.28 (m, 5H), 6.94 (d, *J* = 8.3 Hz, 1H), 6.43 (s, 1H), 5.35 and 5.13 (two s, total 2H), 3.86 (s, 3H), 3.44 and 3.39 (two s, total 3H), 1.51 (s, 9H). **$^{13}$C NMR (126 MHz, $CDCl_3$)** δ 198.2, 197.1, 156.6, 153.0, 135.7, 134.9, 131.8, 129.0, 128.7, 128.1, 127.9, 127.8, 127.4, 123.6, 120.2, 112.4, 112.3, 80.4 59.8, 58.5, 56.6, 43.5, 39.4, 28.4. **HRMS-ESI** (m/z): calcd for $C_{21}H_{27}N_2O_3S_2$ $[M + H]^+$ 419.1458; found 419.1461.

**tert-Butyl (3-((benzylcarbamothioyl)thio)-4-methoxyphenyl)carbamate (13)**

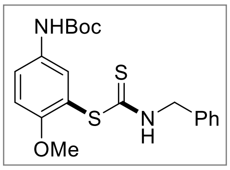


The compound was prepared according to **GP1** using benzyl amine (0.021 g, 0.24 mmol), $CS_2$ (0.037 g, 0.5 mmol) and *tert*-butyl (4-methoxyphenyl)carbamate (0.044 g, 0.2 mmol). Purification by column chromatography (15-20% ethyl acetate in hexane) gave **13** as a yellow oil (0.061 g, 76%). **$^1$H NMR (500 MHz, $CDCl_3$)** δ 9.29 (s, 1H), 7.45 – 7.38 (m, 2H), 7.34 (dd, *J* = 9.2, 7.1 Hz, 3H), 7.29 (d, *J* = 6.5 Hz, 2H), 6.71 (d, *J* = 8.9 Hz, 1H), 6.39 (s, 1H), 4.93 (d, *J* = 5.4 Hz, 2H), 3.43 (s, 3H), 1.51 (s, 9H). **$^{13}$C NMR (126 MHz, $CDCl_3$)** δ 191.8, 153.9, 152.9, 135.9, 132.7, 129.1, 128.3, 128.1, 124.9, 122.7, 122.1, 111.9, 80.9, 56.1, 50.5, 28.5. **HRMS-ESI** (m/z): calcd for $C_{20}H_{25}N_2O_3S_2$ $[M + H]^+$ 405.1301; found 405.1301.

**tert-Butyl (4-methoxy-3-(((pyridin-2-ylmethyl)carbamothioyl)thio)phenyl)carbamate (14)**

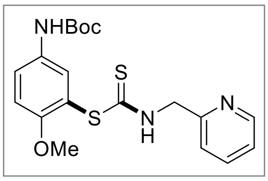


The compound was prepared according to **GP1/GP3** using 2-picolylamine (0.048 g, 0.24 mmol), $CS_2$ (0.037 g, 0.5 mmol) and *tert*-butyl (4-methoxyphenyl)carbamate (0.044 g, 0.2 mmol). Purification by column chromatography (15-20% ethyl acetate in hexane) gave **14** as a yellow oil (0.057 g, 71%). **$^1$H NMR (500 MHz, $CDCl_3$)** δ 8.55 (s, 1H), 8.28 (d, *J* = 4.6 Hz, 1H), 7.69 – 7.60 (m, 2H), 7.48 (s, 1H), 7.21 (d, *J* = 7.8 Hz, 1H), 7.17 – 7.12 (m, 1H), 6.98 (d, *J* = 8.9 Hz, 1H), 6.61 (s, 1H), 4.83 (d, *J* = 4.0 Hz, 2H), 3.76 (s, 3H), 1.48 (s, 9H). **$^{13}$C NMR (126 MHz, $CDCl_3$)** δ 193.9, 155.6, 154.1, 153.0, 148.6, 137.1, 132.5, 127.6, 124.0, 122.7, 122.1, 116.8, 112.6, 80.8, 56.3, 50.0, 28.4. **HRMS-ESI** (m/z): calcd for $C_{19}H_{24}N_3O_3S_2$ $[M + H]^+$ 406.1254; found 406.1223.

**tert-Butyl (3-(((furan-2-ylmethyl)carbamothioyl)thio)-4-methoxyphenyl)carbamate (15)**

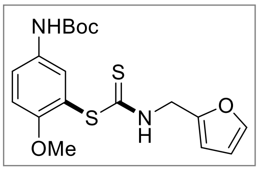


The compound was prepared according to **GP1** using furfuryl amine (0.023 g, 0.24 mmol), $CS_2$ (0.037 g, 0.5 mmol) and *tert*-butyl (4-methoxyphenyl)carbamate (0.044 g, 0.2 mmol). Purification by column chromatography (15-20% ethyl acetate in hexane) gave **15** as a yellow oil (0.062 g, 78%). **$^1$H NMR (500 MHz, $CDCl_3$)** δ 7.55 (d, *J* = 7.6 Hz, 1H), 7.46 (d, *J* = 2.6 Hz, 1H), 7.30 (d, *J* = 1.0 Hz, 1H), 7.04 (s, 1H), 6.92 (d, *J* = 9.0 Hz, 1H), 6.52 (s, 1H), 6.29 (dd, *J* = 3.0, 1.9 Hz, 1H), 6.23 (d, *J* = 2.9 Hz, 1H), 4.81 (d, *J* = 5.2 Hz, 2H), 3.75 (s, 3H), 1.50 (s, 9H). **$^{13}$C NMR (126 MHz, $CDCl_3$)** δ 194.4, 155.3, 153.0, 149.2, 142.5, 132.6, 127.5, 124.3, 116.8, 112.6, 110.7, 108.6, 81.0, 56.3, 43.1, 28.4. **HRMS-ESI** (m/z): calcd for $C_{18}H_{23}N_2O_4S_2$ $[M + H]^+$ 395.1094; found 395.1064.

**tert-Butyl (4-methoxy-3-(((thiophen-2-ylmethyl)carbamothioyl)thio)phenyl)carbamate (16)**

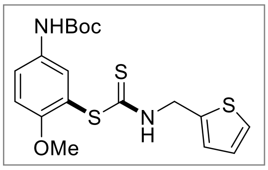


The compound was prepared according to **GP1** using 2-thiophenemethylamine (0.024 g, 0.24 mmol), $CS_2$ (0.037 g, 0.5 mmol) and *tert*-butyl (4-methoxyphenyl)carbamate (0.044 g, 0.2 mmol). Purification by column chromatography (15-20% ethyl acetate in hexane) gave **16** as a yellow oil (0.063 g, 76%). **$^1$H NMR (500 MHz, $CDCl_3$)** δ 7.70 – 6.44 (m, 2H), 7.33 (d, *J* = 1.0 Hz, 1H), 7.07 (s, 1H), 6.95 (d, *J* = 9.0 Hz, 1H), 6.55 (s, 1H), 6.33 – 6.24 (m, 2H), 4.84 (d, *J* = 5.2 Hz, 2H), 3.77 (s, 3H), 1.52 (s, 9H). **$^{13}$C NMR (126 MHz, $CDCl_3$)** δ 194.3, 155.2, 152.9, 149.2, 142.5, 132.5, 127.4, 124.2, 116.8, 112.6, 110.6, 108.5, 80.9, 56.3, 43.0, 28.3. **HRMS-ESI** (m/z): calcd for $C_{18}H_{23}N_2O_3S_3$ $[M + H]^+$ 411.0865; found 411.0833.

**tert-Butyl (4-methoxy-3-((methylcarbamothioyl)thio)phenyl)carbamate (17)**

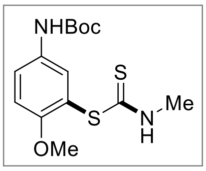


The compound was prepared according to **GP1** using methyl amine (0.008 g, 0.24 mmol), $CS_2$ (0.037 g, 0.5 mmol) and *tert*-butyl (4-methoxyphenyl)carbamate (0.044 g, 0.2 mmol). Purification by column chromatography (15-20% ethyl acetate in hexane) gave **17** as a yellow oil (0.045 g, 68%). **$^1$H NMR (500 MHz, $CDCl_3$)** δ 7.55 (d, *J* = 7.8 Hz, 1H), 7.48 (d, *J* = 2.2 Hz, 1H), 6.96 (d, *J* = 8.9 Hz, 1H), 6.85 (d, *J* = 3.4 Hz, 1H), 6.59 (s, 1H), 3.83 (s, 3H), 3.12 (d, *J* = 4.8 Hz, 3H), 1.49 (s, 9H). **$^{13}$C NMR (126 MHz, $CDCl_3$)** δ 195.1, 155.7, 153.1, 132.6, 128.1, 124.5, 116.8, 112.9, 81.0, 56.6, 33.5, 28.4. **HRMS-ESI** (m/z): calcd for $C_{14}H_{20}N_2NaO_3S_2$ $[M + Na]^+$ 351.0808; found 351.0806.

**tert-Butyl (3-((cyclohexylcarbamothioyl)thio)-4-methoxyphenyl)carbamate (18)**

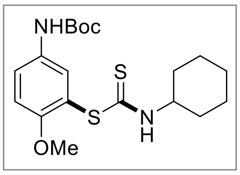


The compound was prepared according to **GP1** using cyclohexylamine (0.024 g, 0.24 mmol), $CS_2$ (0.037 g, 0.5 mmol) and *tert*-butyl (4-methoxyphenyl)carbamate (0.044 g, 0.2 mmol). Purification by column chromatography (15-20% ethyl acetate in hexane) gave **18** as a yellow oil (0.060 g, 76%). **$^1$H NMR (500 MHz, $CDCl_3$)** δ 7.59 (d, *J* = 7.6 Hz, 1H), 7.45 (d, *J* = 2.6 Hz, 1H), 6.96 (d, *J* = 8.9 Hz, 1H), 6.66 (d, *J* = 7.6 Hz, 1H), 6.53 (s, 1H), 4.40 – 4.28 (m, 1H), 3.83 (s, 3H), 1.95 – 1.85 (m, 2H), 1.56 – 1.51 (m, 3H), 1.50 (s, 9H), 1.40 – 1.29 (m, 2H), 1.15 – 1.04 (m, 3H). **$^{13}$C NMR (126 MHz, $CDCl_3$)** δ 192.0, 155.2, 152.9, 132.6, 127.3, 123.9, 117.1, 112.5, 80.9, 56.3, 54.5, 31.4, 28.3, 25.3, 24.3. **HRMS-ESI** (m/z): calcd for $C_{19}H_{29}N_2O_3S_2$ $[M + H]^+$ 397.1614; found 397.1594.

***tert*-Butyl (3-((allylcarbamothioyl)thio)-4-methoxyphenyl)carbamate (19)**

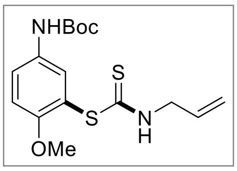


The compound was prepared according to **GP1/GP3** using allylamine (0.024 g, 0.24 mmol), $CS_2$ (0.037 g, 0.5 mmol) and *tert*-butyl (4-methoxyphenyl)carbamate (0.044 g, 0.2 mmol). Purification by column chromatography (15-20% ethyl acetate in hexane) gave **19** as a yellow oil (0.056 g, 79%). **$^1$H NMR (500 MHz, $CDCl_3$)** δ 7.55 – 6.45 (m, 2H), 6.96 (d, $J$ = 8.9 Hz, 1H), 6.82 (s, 1H), 6.56 (s, 1H), 5.85 – 5.71 (m, 1H), 5.15 – 4.95 (m, 2H), 4.26 (t, $J$ = 5.4 Hz, 2H), 3.83 (s, 3H), 1.49 (s, 9H). **$^{13}$C NMR (126 MHz, $CDCl_3$)** δ 194.4, 155.5, 153.1, 132.6, 131.9, 127.8, 124.4, 117.5, 116.8, 112.8, 81.0, 56.5, 48.3, 28.4. **HRMS-ESI** (m/z): calcd for $C_{16}H_{23}N_2O_3S_2$ $[M + H]^+$ 355.1145; found 355.1117.

***tert*-Butyl *(S)*-(4-methoxy-3-(((1-phenylethyl)carbamothioyl)thio)phenyl)carbamate (20)**

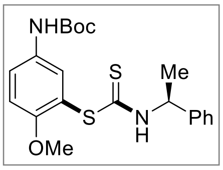


The compound was prepared according to **GP1** using *(S)*-1-phenylethan-1-amine (0.029 g, 0.24 mmol), $CS_2$ (0.037 g, 0.5 mmol) and *tert*-butyl (4-methoxyphenyl)carbamate (0.044 g, 0.2 mmol). Purification by column chromatography (15-20% ethyl acetate in hexane) gave **20** as a white solid (0.067 g, 81%). **$^1$H NMR (500 MHz, $CDCl_3$)** δ 7.34 (s, 1H), 7.26 (s, 1H), 7.08 (t, $J$ = 7.2 Hz, 2H), 7.05 –7.00 (m, 1H), 6.97 (d, $J$ = 7.4 Hz, 2H), 6.73 (d, $J$ = 7.7 Hz, 1H), 6.67 (d, $J$ = 8.9 Hz, 1H), 6.38 (s, 1H), 5.57 –5.48 (m, 1H), 3.46 (s, 3H), 1.29 (s, 9H), 1.25 (d, $J$ = 6.9 Hz, 3H). **$^{13}$C NMR (126 MHz, $CDCl_3$)** δ 192.7, 155.1, 153.0, 141.2, 132.6, 128.8, 127.8, 127.2, 126.3, 124.0, 116.8, 112.4, 80.9, 56.1, 55.0, 28.4, 20.4. **HRMS-ESI** (m/z): calcd for $C_{21}H_{27}N_2O_3S_2$ $[M + H]^+$ 419.1458; found 419.1457.

**2-Methoxy-5-((methoxycarbonyl)amino)phenyl piperidine-1-carbodithioate (21)**

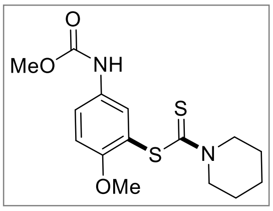


The compound was prepared according to **GP1** using piperidine (0.020 g, 0.24 mmol), $CS_2$ (0.037 g, 0.5 mmol) and methyl (4-methoxyphenyl)carbamate (0.036 g, 0.2 mmol). Purification by column chromatography (15-20% ethyl acetate in hexane) gave **21** as a yellow oil (0.060 g, 89%). **$^1$H NMR (500 MHz, DMSO)** δ 9.48 (s, 1H), 7.56 – 7.36 (m, 2H), 6.96 (d, $J$ = 9.0 Hz, 1H), 4.09 (s, 2H), 3.90 (s, 2H), 3.64 (s, 3H), 3.57 (s, 3H), 1.61 – 1.49 (m, 6H). **$^{13}$C NMR (126 MHz, DMSO)** δ 193.1, 155.7, 154.1, 132.3, 128.0, 122.1, 119.1, 112.4, 56.1, 56.1, 52.5, 51.6, 26.0, 25.1, 23.5. **HRMS-ESI** (m/z): calcd for $C_{15}H_{21}N_2O_3S_2$ $[M + H]^+$ 341.0988; found 341.0994.

**5-((Ethoxycarbonyl)amino)-2-methoxyphenyl piperidine-1-carbodithioate (22)**

The compound was prepared according to **GP1** using piperidine (0.020 g, 0.24 mmol), $CS_2$ (0.037 g, 0.5 mmol) and ethyl (4-methoxyphenyl)carbamate (0.039 g, 0.2 mmol). Purification by column chromatography (15-20% ethyl acetate in hexane) gave **22** as a white solid (0.061 g, 86%). **$^1$H NMR (500 MHz, $CDCl_3$)** δ 7.53 (s, 1H), 7.41 (s, 1H), 6.91 (d, $J$ = 8.9 Hz, 1H), 6.72 (s, 1H), 4.35 – 4.13 (m, 4H), 3.99 (br s, 2H), 3.81 (br s, 3H), 1.70 (s, 6H), 1.27 (t, $J$ = 7.1

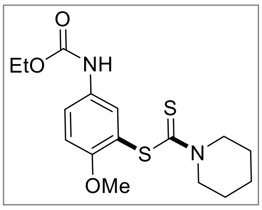


Hz, 3H).[13]**C NMR (126 MHz, CDCl₃)** δ 194.9, 156.8, 153.9, 131.4, 129.5, 123.2, 120.1, 112.2, 61.2, 56.6, 53.3, 52.2, 26.2, 25.4, 24.2, 14.6. **HRMS-ESI** (m/z): calcd for $C_{16}H_{23}N_2O_3S_2$ $[M + H]^+$ 355.1145; found 355.1153.

**5-((Butoxycarbonyl)amino)-2-methoxyphenyl piperidine-1-carbodithioate (23)**

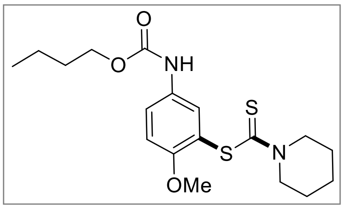


The compound was prepared according to **GP1** using piperidine (0.020 g, 0.24 mmol), $CS_2$ (0.037 g, 0.5 mmol) and butyl (4-methoxyphenyl)carbamate (0.044 g, 0.2 mmol). Purification by column chromatography (15-20% ethyl acetate in hexane) gave **23** as a yellow oil (0.066 g, 87%). **$^1$H NMR (400 MHz, $CDCl_3$)** δ 7.55 – 7.35 (m, 2H), 6.97 (s, 1H), 6.87 (d, *J* = 8.9 Hz, 1H), 4.20 (br s, 2H), 4.09 (t, *J* = 6.6 Hz, 2H), 3.95 (br s, 2H), 3.77 (s, 3H), 1.66 (s, 6H), 1.61 – 1.55 (m, 2H), 1.34 (dd, *J* = 15.0, 7.5 Hz, 2H), 0.89 (t, *J* = 7.4 Hz, 3H). **$^{13}$C NMR (101 MHz, $CDCl_3$)** δ 194.6, 156.5, 153.9, 131.4, 129.1, 122.9, 119.8, 112.0, 64.8, 56.4, 53.1, 52.0, 30.8, 26.0, 25.3, 24.0, 18.9, 13.6. **HRMS-ESI** (m/z): calcd for $C_{18}H_{27}N_2O_3S_2$ $[M + H]^+$ 383.1458; found 383.1477.

**5-((Isobutoxycarbonyl)amino)-2-methoxyphenyl piperidine-1-carbodithioate (24)**

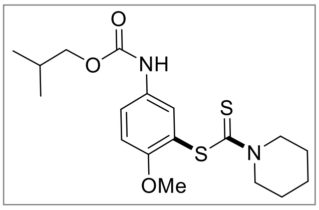


The compound was prepared according to **GP1** using piperidine (0.020 g, 0.24 mmol), $CS_2$ (0.037 g, 0.5 mmol) and isobutyl (4-methoxyphenyl)carbamate (0.044 g, 0.2 mmol). Purification by column chromatography (15-20% ethyl acetate in hexane) gave **24** as a yellow oil (0.065 g, 86%). **$^1$H NMR (500 MHz, $CDCl_3$)** δ 7.56 (s, 1H), 7.40 (s, 1H), 6.92 (d, *J* = 8.9 Hz, 1H), 6.69 (s, 1H), 4.24 (s, 2H), 3.99 (s, 2H), 3.91 (d, *J* = 6.5 Hz, 2H), 3.82 (s, 3H), 1.94 (dt, *J* = 13.3, 6.7 Hz, 1H), 1.71 (br s, 6H), 0.93 (d, *J* = 6.7 Hz, 6H).**$^{13}$C NMR (126 MHz, $CDCl_3$)** δ 194.9, 156.8, 154.0, 137.5, 131.4, 129.4, 123.1, 120.1, 112.2, 77.4, 71.4, 56.6, 53.3, 52.2, 28.0, 26.2, 25.4, 24.3, 19.1, 19.1. **HRMS-ESI** (m/z): calcd for $C_{18}H_{26}N_2O_3S_2$ $[M + K]^+$ 382.1853; found 382.1865.

**5-((Isopropoxycarbonyl)amino)-2-methoxyphenyl piperidine-1-carbodithioate (25)**

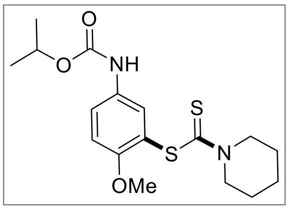


The compound was prepared according to **GP1/GP3** using piperidine (0.020 g, 0.24 mmol), $CS_2$ (0.037 g, 0.5 mmol) and isopropyl (4-methoxyphenyl)carbamate (0.040 g, 0.2 mmol). Purification by column chromatography (15-20% ethyl acetate in hexane) gave **25** as a yellow oil (0.59 g, 81%). **$^1$H NMR (500 MHz, $CDCl_3$)** δ 7.52 (d, *J* = 6.1 Hz, 1H), 7.41 (s, 1H), 6.90 (d, *J* = 8.9 Hz, 1H), 6.68 (s, 1H), 4.96 (dt, *J* = 12.4, 6.2 Hz, 1H), 4.22 (br s, 2H), 3.98 (br s, 2H), 3.80 (s, 3H), 1.69 (s, 6H), 1.25 (d, *J* = 6.3 Hz, 6H).**$^{13}$C NMR (126 MHz, $CDCl_3$)** δ 194.8, 156.7, 153.5, 131.5, 129.3, 123.0,

120.1, 112.2, 68.6, 56.6, 53.2, 52.1, 26.2, 25.4, 24.2, 22.1. **HRMS-ESI** (m/z): calcd for $C_{17}H_{25}N_2O_3S_2$ [M + H]$^+$ 369.1301; found 369.1314.

**5-(((Allyloxy)carbonyl)amino)-2-methoxyphenyl piperidine-1-carbodithioate (26)**

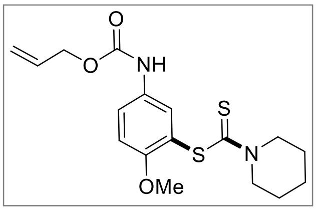


The compound was prepared according to **GP1/GP3** using piperidine (0.020 g, 0.24 mmol), $CS_2$ (0.037 g, 0.5 mmol) and allyl (4-methoxyphenyl)carbamate (0.074 g, 0.2 mmol). Purification by column chromatography (15-20% ethyl acetate in hexane) gave **26** as a yellow oil (0.061 g, 84%). **$^1$H NMR (500 MHz, CDCl$_3$)** δ 7.55 (s, 1H), 7.41 (s, 1H), 6.92 (d, *J* = 8.9 Hz, 1H), 6.78 (d, *J* = 9.3 Hz, 1H), 6.01 – 5.86 (m, 1H), 5.32 (dt, *J* = 11.4, 5.7 Hz, 1H), 5.26 – 5.19 (m, 1H), 4.63 (d, *J* = 5.5 Hz, 2H), 4.24 (s, 2H), 3.98 (d, *J* = 18.1 Hz, 2H), 3.82 (s, 3H), 1.71 (s, 6H).**$^{13}$C NMR (126 MHz, CDCl$_3$)** δ 194.9, 157.0, 153.6, 132.6, 131.2, 129.5, 123.3, 120.2, 118.2, 117.9, 115.8, 112.3, 65.8, 53.3, 52.2, 26.2, 25.4, 24.3. **HRMS-ESI** (m/z): calcd for $C_{17}H_{23}N_2O_3S_2$ [M + H]$^+$ 367.1145; found 367.1164.

**2-Methoxy-5-(((prop-2-yn-1-yloxy)carbonyl)amino)phenyl piperidine-1-carbodithioate (27)**

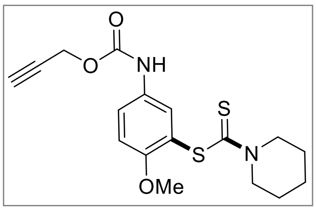


The compound was prepared according to **GP1** using piperidine (0.020 g, 0.24 mmol), $CS_2$ (0.037 g, 0.5 mmol) and prop-2-yn-1-yl (4-methoxyphenyl)carbamate (0.040 g, 0.2 mmol). Purification by column chromatography (15-20% ethyl acetate in hexane) gave **27** as a yellow oil (0.055 g, 76%). **$^1$H NMR (500 MHz, CDCl$_3$)** δ 7.53 (d, *J* = 6.8 Hz, 2H), 7.42 (s, 2H), 6.96 – 6.84 (m, 2H), 4.74 (d, *J* = 2.0 Hz, 2H), 4.24 (br s, 2H), 4.00 (br s, 2H), 3.81 (s, 3H), 2.50 (t, *J* = 2.3 Hz, 1H), 1.71 (br s, 6H).**$^{13}$C NMR (126 MHz, CDCl$_3$)** δ 194.7, 157.1, 152.8, 130.8, 129.6, 123.4, 120.2, 115.9, 112.3, 78.0, 75.1, 56.6, 53.3, 52.8, 52.2, 26.2, 25.4, 24.2. **HRMS-ESI** (m/z): calcd for $C_{17}H_{21}N_2O_3S_2$ [M + H]$^+$ 365.0988; found 365.1005.

**5-Acetamido-2-methoxyphenyl piperidine-1-carbodithioate (28)**

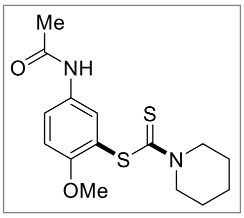


The compound was prepared according to **GP1** using piperidine (0.020 g, 0.24 mmol), $CS_2$ (0.037 g, 0.5 mmol) and *N*-(4-methoxyphenyl)acetamide (0.033 g, 0.2 mmol). Purification by column chromatography (15-20% ethyl acetate in hexane) gave **28** as a white solid (0.056 g, 87%). **$^1$H NMR (500 MHz, CDCl$_3$)** δ 7.78 (s, 1H), 7.62 (dd, *J* = 8.9, 2.6 Hz, 1H), 7.52 (d, *J* = 2.6 Hz, 1H), 6.89 (d, *J* = 8.9 Hz, 1H), 4.25 (s, 2H), 4.00 (s, 2H), 3.81 (s, 3H), 2.07 (s, 3H), 1.72 (s, 6H). **$^{13}$C NMR (151 MHz, CDCl$_3$)** δ 195.1, 168.6, 157.2, 131.7, 130.5, 124.6, 119.8, 112.1, 56.6, 53.4, 52.3, 26.3, 25.5, 24.3, 24.3. **HRMS-ESI** (m/z): calcd for $C_{15}H_{21}N_2O_2S_2$ [M + H]$^+$ 325.1039; found 325.1042.

**4-Bromo-5-((*tert*-butoxycarbonyl)amino)-2-methoxyphenyl piperidine-1-carbodithioate (29)**

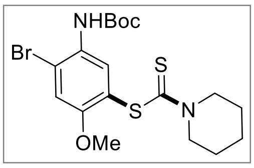


The compound was prepared according to **GP1/GP3** using piperidine (0.020 g, 0.24 mmol), $CS_2$ (0.037 g, 0.5 mmol) and *tert*-butyl (2-bromo-4-methoxyphenyl)carbamate (0.060 g, 0.2 mmol). Purification by column chromatography (15-20% ethyl acetate in hexane) gave **29** as a yellow oil (0.066 g, 72%). **$^1$H NMR (500 MHz, $CDCl_3$)** δ 8.21 (d, *J* = 8.0 Hz, 1H), 6.97 (d, *J* = 9.2 Hz, 1H), 6.93 (s, 1H), 4.24 (br s, 2H), 4.05 (br s, 2H), 3.84 (s, 3H), 1.74 (br s, 6H), 1.52 (s, 9H). **$^{13}$C NMR (126 MHz, $CDCl_3$)** δ 192.9, 157.7, 152.9, 131.1, 124.0, 122.0, 111.5 (2C), 81.0, 57.2, 53.4, 52.6, 28.4, 26.6, 25.6, 24.4. **HRMS-ESI** (m/z): calcd for $C_{18}H_{25}BrN_2O_3S_2$ $[M + K]^+$ 460.0490; found 460.0495.

**5-((*tert*-Butoxycarbonyl)amino)-2-methoxy-4-methylphenyl piperidine-1-carbodithioate (30)**

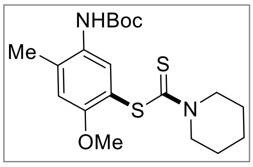


The compound was prepared according to **GP1** using piperidine (0.020 g, 0.24 mmol), $CS_2$ (0.037 g, 0.5 mmol) and *tert*-butyl (4-methoxy-2-methylphenyl)carbamate (0.047 g, 0.2 mmol). Purification by column chromatography (15-20% ethyl acetate in hexane) gave **30** as a yellow oil (0.058 g, 73%). **$^1$H NMR (500 MHz, $CDCl_3$)** δ 7.68 (br s, 1H), 6.84 – 6.75 (m, 1H), 6.14 (s, 1H), 4.23 (br s, 2H), 3.99 (br s, 2H), 3.80 (s, 3H), 2.28 (s, 3H), 1.70 (br s, 6H), 1.47 (s, 9H). **$^{13}$C NMR (126 MHz, $CDCl_3$)** δ 195.5, 157.4, 153.5, 129.4, 120.4, 117.3, 113.8, 109.4, 80.2, 56.5, 53.2, 52.0, 28.3, 26.1, 25.4, 24.2, 18.5. **HRMS-ESI** (m/z): calcd for $C_{19}H_{29}N_2O_3S_2$ $[M + H]^+$ 397.1614; found 397.1615.

**2-((*tert*-Butoxycarbonyl)amino)-5-methoxy-[1,1'-biphenyl]-4-yl piperidine-1-carbodithioate (31)**

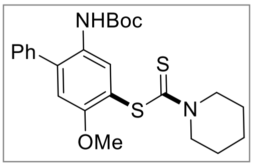


The compound was prepared according to **GP1** using piperidine (0.020 g, 0.24 mmol), $CS_2$ (0.037 g, 0.5 mmol) and *tert*-butyl (5-methoxy-[1,1'-biphenyl]-2-yl)carbamate (0.060 g, 0.2 mmol). Purification by column chromatography (15-20% ethyl acetate in hexane) gave **31** as a yellow oil (0.066 g, 73%). **$^1$H NMR (500 MHz, $CDCl_3$)** δ 8.25 – 8.10 (m, 1H), 7.49 (t, *J* = 7.2 Hz, 2H), 7.46 – 7.37 (m, 3H), 6.83 (s, 1H), 6.27 (s, 1H), 4.28 (br s, 2H), 4.03 (br s, 2H), 3.83 (s, 3H), 1.74 (br s, 6H), 1.43 (s, 9H). **$^{13}$C NMR (151 MHz, $CDCl_3$)** δ 195.1, 156.7, 153.2, 138.2, 129.7, 129.3 (2C), 129.1, 128.6, 128.5, 128.2, 128.1, 119.5, 113.8, 111.7, 80.4, 56.7, 53.3, 52.2, 28.4, 28.4, 26.3, 25.5, 24.4. **HRMS-ESI** (m/z): calcd for $C_{24}H_{30}N_2O_3S_2$ $[M + K]^+$ 458.1698; found 458.1702.

**2-((tert-butoxycarbonyl)amino)-5-methoxy-4'-methyl-[1,1'-biphenyl]-4-yl piperidine-1-carbodithioate (32)**

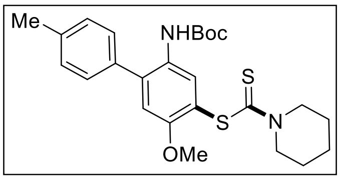


The compound was prepared according to **GP1** using piperidine (0.020 g, 0.24 mmol), $CS_2$ (0.037 g, 0.5 mmol) and *tert*-butyl (5-methoxy-4'-methyl-[1,1'-biphenyl]-2-yl)carbamate (0.062 g, 0.2 mmol). Purification by column chromatography (15-20% ethyl acetate in hexane) gave **32** as a white solid (0.068 g, 73%). **$^1$H NMR (400 MHz, $CDCl_3$)** δ 8.24 – 8.04 (m, 1H), 7.36 – 7.29 (m, 3H), 7.21 – 7.16 (1H, m), 6.80 (s, 1H), 6.30 (s, 1H), 4.25 (s, 2H), 4.02 (s, 2H), 3.81 (s, 3H), 2.41 (s, 3H), 1.69 (br s, 6H), 1.43 (s, 9H). **$^{13}$C NMR (101 MHz, $CDCl_3$)** δ 195.1, 156.6, 153.2, 138.0, 137.7, 135.1, 129.8, 129.1, 128.6, 119.2, 113.8, 111.5, 80.3, 56.6, 53.3, 52.2, 28.4, 26.3, 25.6, 24.3, 21.3 **.HRMS-ESI** (m/z): calcd for $C_{25}H_{32}N_2O_3S_2$ $[M + H]^+$ 473.1927; found 473.1932.

**5-((*tert*-Butoxycarbonyl)amino)-2-ethoxyphenyl piperidine-1-carbodithioate (33)**

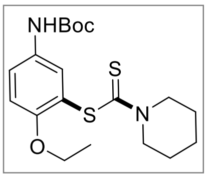


The compound was prepared according to **GP2** using piperidine (0.020 g, 0.24 mmol), $CS_2$ (0.037 g, 0.5 mmol) and *tert*-butyl (4-methoxyphenyl)carbamate (0.044 g, 0.2 mmol) in ethanol (1 ml). Purification by column chromatography (15-20% ethyl acetate in hexane) gave **33** as a yellow oil (0.062 g, 78%). **$^1$H NMR (500 MHz, $CDCl_3$)** δ 7.50 – 7.38 (m, 2H), 6.88 (d, $J$ = 8.9 Hz, 1H), 6.51 (s, 1H), 4.24 (s, 2H), 4.03 (q, $J$ = 7.0 Hz, 4H), 1.71 (br s, 6H), 1.48 (s, 9H), 1.35 (t, $J$ = 7.0 Hz, 3H). **$^{13}$C NMR (126 MHz, $CDCl_3$)** δ 195.2, 155.9, 153.0, 131.6, 128.9, 122.9, 120.8, 113.6, 80.3, 65.2, 53.2, 52.4, 28.4, 26.3, 25.5, 24.3, 14.9. **HRMS-ESI** (m/z): calcd for $C_{19}H_{29}N_2O_3S_2$ $[M + H]^+$ 397.1614; found 397.1620.

**2-Butoxy-5-((*tert*-butoxycarbonyl)amino)phenyl piperidine-1-carbodithioate (34)**

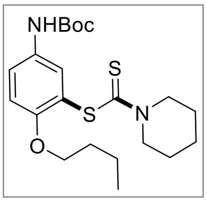


The compound was prepared according to **GP2** using piperidine (0.020 g, 0.24 mmol), $CS_2$ (0.037 g, 0.5 mmol) and *tert*-butyl (4-methoxyphenyl)carbamate (0.044 g, 0.2 mmol) in butanol (1 ml). Purification by column chromatography (15-20% ethyl acetate in hexane) gave **34** as a yellow oil (0.068 g, 81%). **$^1$H NMR (500 MHz, $CDCl_3$)** δ 7.47 (s, 1H), 7.39 (s, 1H), 6.89 (d, $J$ = 8.9 Hz, 1H), 6.40 (s, 1H), 4.24 (br s, 2H), 4.03 (br s, 2H), 3.97 (t, $J$ = 6.4 Hz, 2H), 1.76 – 1.70 (m, 8H), 1.49 (s, 9H), 1.49 – 1.43 (m, 2H), 0.93 (t, $J$ = 7.4 Hz, 3H). **$^{13}$C NMR (126 MHz, $CDCl_3$)** δ 195.3, 156.1, 153.0, 131.5, 129.0, 122.9, 120.8, 113.4, 80.4, 7.1, 53.2, 52.3, 31.4, 28.4, 26.3, 25.6, 24.4, 19.3, 14.0. **HRMS-ESI** (m/z): calcd for $C_{21}H_{33}N_2O_3S_2$ $[M + H]^+$ 425.1927; found 425.1925.

**5-((*tert*-Butoxycarbonyl)amino)-2-isobutoxyphenyl piperidine-1-carbodithioate (35)**

The compound was prepared according to **GP2** using piperidine (0.020 g, 0.24 mmol), $CS_2$ (0.037 g, 0.5 mmol) and *tert*-butyl (4-methoxyphenyl)carbamate (0.044 g, 0.2 mmol) in isobutanol (1 ml). Purification by column chromatography (15-20% ethyl acetate in hexane)

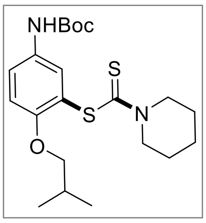


gave **35** as a yellow oil (0.068 g, 81%). **$^1$H NMR (500 MHz, CDCl$_3$)** δ 7.6 – 7.3 (m, 2H), 6.85 (d, *J* = 8.9 Hz, 1H), 6.52 (s, 1H), 4.22 (br s, 2H), 4.01 (br s, 2H), 3.71 (d, *J* = 6.4 Hz, 2H), 2.04 (dp, *J* = 13.2, 6.6 Hz, 1H), 1.70 (s, 6H), 1.48 (s, 9H), 0.97 (d, *J* = 6.8 Hz, 6H). **$^{13}$C NMR (126 MHz, CDCl$_3$)** δ 195.3, 155.9, 153.0, 131.4, 128.9, 122.9, 120.7, 113.1, 80.2, 75.4, 53.1, 52.1, 28.4, 28.4, 26.2, 25.5, 24.3, 19.3. **HRMS-ESI** (m/z): calcd for $C_{21}H_{23}N_2O_3S_2$ [M + H]$^+$ 425.1927; found 425.1938.

**5-((*tert*-Butoxycarbonyl)amino)-2-isopropoxyphenyl piperidine-1-carbodithioate (36)**

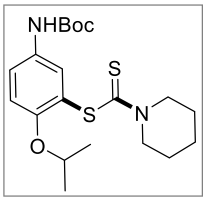


The compound was prepared according to **GP2** using piperidine (0.020 g, 0.24 mmol), $CS_2$ (0.037 g, 0.5 mmol) and *tert*-butyl (4-methoxyphenyl)carbamate (0.044 g, 0.2 mmol) in isopropyl alcohol (1 ml). Purification by column chromatography (15-20% ethyl acetate in hexane) gave **36** as a yellow oil (0.065 g, 79%). **$^1$H NMR (500 MHz, CDCl$_3$)** δ 7.50 – 7.35 (m, 2H), 6.90 (d, *J* = 8.9 Hz, 1H), 6.44 (s, 1H), 4.56 – 4.38 (m, 1H), 4.24 (br s, 2H), 4.03 (br s, 2H), 1.72 (s, 6H), 1.49 (s, 9H), 1.30 (d, *J* = 6.1 Hz, 6H). **$^{13}$C NMR (126 MHz, CDCl$_3$)** δ 195.4, 155.1, 131.7, 129.3, 122.8, 122.0, 115.6, 114.3, 80.4, 72.3, 53.3, 52.5, 28.4, 26.2, 25.6, 24.4, 22.3. **HRMS-ESI** (m/z): calcd for $C_{20}H_{31}N_2O_3S_2$ [M + H]$^+$ 411.1771; found 411.1781.

**2-(Allyloxy)-5-((*tert*-butoxycarbonyl)amino)phenyl piperidine-1-carbodithioate (37)**

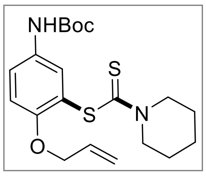


The compound was prepared according to **GP2** using piperidine (0.020 g, 0.24 mmol), $CS_2$ (0.037 g, 0.5 mmol) and *tert*-butyl (4-methoxyphenyl)carbamate (0.044 g, 0.2 mmol) in allylic alcohol (1 ml). Purification by column chromatography (15-20% ethyl acetate in hexane) gave **37** as a yellow oil (0.060 g, 74%). **$^1$H NMR (500 MHz, CDCl$_3$)** δ 7.53 – 7.37 (m, 2H), 6.90 (d, *J* = 8.9 Hz, 1H), 6.41 (s, 1H), 6.06 – 5.93 (m, 1H), 5.40 (dd, *J* = 17.3, 1.6 Hz, 1H), 5.22 (dd, *J* = 10.6, 1.4 Hz, 1H), 4.56 (d, *J* = 4.9 Hz, 2H), 4.25 (s, 2H), 4.02 (s, 2H), 1.73 (s, 6H), 1.49 (s, 9H). **$^{13}$C NMR (126 MHz, CDCl$_3$)** δ 195.2, 155.7, 153.0, 133.3, 132.0, 129.2, 122.8, 121.1, 117.2, 114.1, 80.5, 70.2, 53.3, 52.5, 28.5, 26.3, 25.6, 24.4. **HRMS-ESI** (m/z): calcd for $C_{20}H_{29}N_2O_3S_2$ [M + H]$^+$ 409.1612; found 409.1624.

**5-((*tert*-Butoxycarbonyl)amino)-2-(prop-2-yn-1-yloxy)phenyl piperidine-1-carbodithioate (38)**

The compound was prepared according to **GP2** using piperidine (0.020 g, 0.24 mmol), $CS_2$ (0.037 g, 0.5 mmol) and *tert*-butyl (4-methoxyphenyl)carbamate (0.044 g, 0.2 mmol) in propargylic alcohol (1 ml). Purification by column chromatography (15-20% ethyl acetate in hexane) gave **38** as a yellow oil (0.061 g, 76%). **$^1$H NMR (500 MHz, CDCl$_3$)** δ 7.50 – 7.39 (m, 2H), 6.89 (d, *J* = 8.9 Hz, 1H), 6.55 (s, 1H), 4.29 – 4.12 (m, 2H), 3.98 (br s, 1H), 3.80 (s, 3H), 3.38 – 3.32 (m, 1H), 1.70 (s, 6H), 1.47 (s, 9H). **$^{13}$C NMR (126 MHz, CDCl$_3$)** δ 194.9,

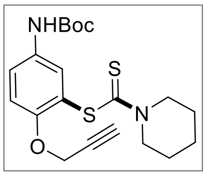


156.6, 153.0, 131.7, 129.3, 123.0, 120.0, 115.7, 112.2 (2C), 80.3, 56.6, 53.2, 52.1, 28.4, 26.2, 25.4, 24.2. **HRMS-ESI** (m/z): calcd for $C_{20}H_{27}N_2O_3S_2$ [M + H]$^+$ 407.1458; found 407.1448.

**Methyl (((5-((*tert*-butoxycarbonyl)amino)-2-methoxyphenyl)thio)carbonothioyl)alaninate (39)**

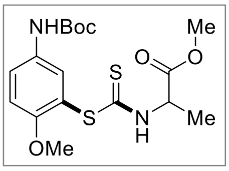


The compound was prepared according to **GP1** using methyl alaninate (0.024 g, 0.24 mmol), $CS_2$ (0.037 g, 0.5 mmol) and *tert*-butyl (4-methoxyphenyl)carbamate (0.044 g, 0.2 mmol). Purification by column chromatography (15-20% ethyl acetate in hexane) gave **39** as a yellow oil (0.057 g, 72%). **$^1$H NMR (500 MHz, CDCl$_3$)** δ 7.57 (d, *J* = 6.1 Hz, 1H), 7.51 (s, 1H), 7.42 (d, *J* = 6.9 Hz, 1H), 6.98 (d, *J* = 8.9 Hz, 1H), 6.66 (d, *J* = 4.7 Hz, 1H), 5.08 – 5.02 (m, 1H), 3.83 (s, 3H), 3.69 (s, 3H), 1.48 (s, 9H), 1.41 (d, *J* = 7.1 Hz, 3H). **$^{13}$C NMR (151 MHz, CDCl$_3$)** δ 193.9, 172.4, 155.2, 153.0, 132.6, 127.1, 124.1, 116.5, 112.6, 80.8, 56.3, 53.8, 52.7, 28.3, 17.5. **HRMS-ESI** (m/z): calcd for $C_{17}H_{25}N_2O_5S_2$ [M + H]$^+$ 401.1199; found 401.1203.

**Methyl (((5-((*tert*-butoxycarbonyl)amino)-2-methoxyphenyl)thio)carbonothioyl)phenylalaninate (40)**

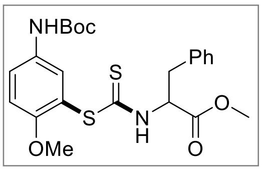


The compound was prepared according to **GP1** using methyl phenylalaninate (0.043 g, 0.24 mmol), $CS_2$ (0.037 g, 0.5 mmol) and *tert*-butyl (4-methoxyphenyl)carbamate (0.044 g, 0.2 mmol). Purification by column chromatography (15-20% ethyl acetate in hexane) gave **40** as a yellow oil (0.067 g, 73%). **$^1$H NMR (500 MHz, CDCl$_3$)** δ 7.54 (d, *J* = 6.5 Hz, 1H), 7.36 (d, *J* = 1.2 Hz, 1H), 7.23 (d, *J* = 7.3 Hz, 1H), 7.20 – 7.16 (m, 3H), 6.87 (t, *J* = 7.2 Hz, 3H), 6.51 (s, 1H), 5.35 – 5.25 (m, 1H), 3.70 (s, 3H), 3.66 (s, 3H), 3.30 (dd, *J* = 14.0, 5.7 Hz, 1H), 3.14 – 3.04 (m, 1H), 1.52 (s, 9H). **$^{13}$C NMR (126 MHz, CDCl$_3$)** δ 194.4, 170.7, 155.2, 152.8, 135.1, 132.5, 129.2, 128.6, 127.1 (2C), 124.0, 116.1, 112.6, 80.8, 58.5, 56.3, 52.5, 36.5, 28.4. **HRMS-ESI** (m/z): calcd for $C_{23}H_{29}N_2O_5S_2$ [M + H]$^+$ 477.1512; found 477.1510.

**Methyl (((5-((*tert*-butoxycarbonyl)amino)-2-methoxyphenyl)thio)carbonothioyl)-*L*-prolinate (41)**

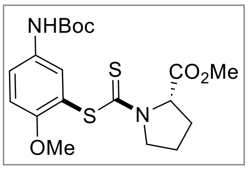


The compound was prepared according to **GP1** using *(S)*-1-phenylethan-1-amine (0.029 g, 0.24 mmol), $CS_2$ (0.037 g, 0.5 mmol) and *tert*-butyl (4-methoxyphenyl)carbamate (0.044 g, 0.2 mmol). Purification by column chromatography (15-20% ethyl acetate in hexane) gave **41** as a yellow oil(0.069 g, 81%). The product was characterized as a mixture of inseparable rotamers with a 4.8:1 ratio at 298K. **$^1$H NMR (500 MHz, CDCl$_3$)** δ 7.50 (s, 1H), 7.38 (d, *J* = 13.8 Hz, 1H), 6.89 (d, *J* = 8.9 Hz, 1H), 6.58 (s, 1H), 5.01 (dd, *J* = 8.3, 2.8 Hz, 1H), 4.08 – 3.95 (m, 1H), 3.88 – 3.82 (m, 1H), 3.79 (s, 3H), 3.69 (s, 3H), 2.31 – 2.18 (m, 2H), 2.16

– 2.04 (m, 2H), 1.47 (s, 9H). **$^{13}$C NMR (126 MHz, CDCl$_3$)** δ 194.3, 193.8, 171.1, 170.8, 156.4, 156.3, 153.0, 137.4, 131.8, 129.0, 123.1, 119.1, 116.5, 112.3, 80.2, 66.4, 63.2, 56.6, 55.4, 52.8, 52.3, 51.3, 31.7, 29.3, 28.3, 24.8, 22.5. **HRMS-ESI** (m/z): calcd for $C_{19}H_{27}N_2O_5S_2$ [M + H]$^+$ 427.1356; found 427.1362.

**5-*((tert*-Butoxycarbonyl)amino)-2-methoxyphenyl 4-(2-(4-isobutylphenyl)propanoyl)piperazine-1-carbodithioate (42)**

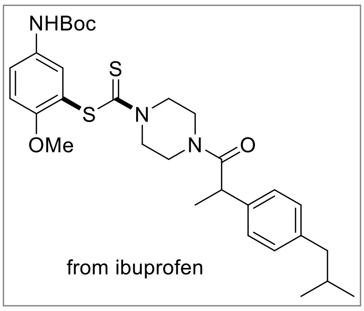


The compound was prepared according to **GP1** using *(S)*-1-phenylethan-1-amine (0.029 g, 0.24 mmol), CS$_2$ (0.037 g, 0.5 mmol) and *tert*-butyl (4-methoxyphenyl)carbamate (0.044 g, 0.2 mmol). Purification by column chromatography (15-20% ethyl acetate in hexane) gave **42** as a yellow oil (0.070 g, 62%). **$^1$H NMR (400 MHz, CDCl$_3$)** δ 7.49 – 7.39 (m, 2H), 7.12 (t, *J* = 6.6 Hz, 4H), 6.90 (d, *J* = 8.9 Hz, 1H), 6.43 (s, 1H), 4.14 – 3.94 (m, 2H), 3.88 – 3.81 (m, 2H), 3.79 (br s, 3H), 3.65 – 3.42 (m, 4H), 3.34 (br s, 1H), 2.44 (d, *J* = 7.1 Hz, 2H), 1.88 – 1.80 (m, 1H), 1.48 (s, 9H), 1.45 (d, *J* = 6.7 Hz, 3H), 0.89 (d, *J* = 6.6 Hz, 6H). **$^{13}$C NMR (126 MHz, CDCl$_3$)** $^{13}$C NMR (101 MHz, CDCl$_3$) δ 197.0, 172.7, 156.6, 153.0, 140.8, 138.8, 131.9, 130.0, 129.2, 126.9, 123.3, 119.2, 112.3, 80.6, 56.6, 45.1, 44.7, 43.5, 41.5, 34.0, 30.3, 28.5, 25.7, 25.0, 22.5, 20.7. **HRMS-ESI** (m/z): calcd for $C_{30}H_{42}N_3O_4S_2$ [M + H]$^+$ 572.2611; found 572.2611.

**5-((*tert*-Butoxycarbonyl)amino)-2-methoxyphenyl (*S*)-4-(2-(6-methoxynaphthalen-2-yl)propanoyl)piperazine-1-carbodithioate (43)**

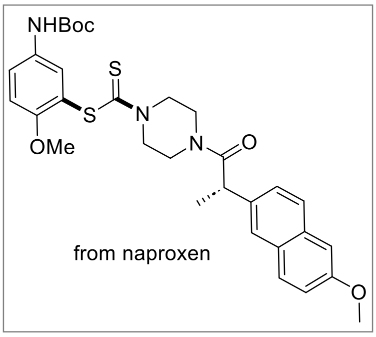


The compound was prepared according to **GP1** using (*S*)-2-(6-methoxynaphthalen-2-yl)-1-(piperazin-1-yl)propan-1-one (0.071 g, 0.24 mmol), CS$_2$ (0.037 g, 0.5 mmol) and *tert*-butyl (4-methoxyphenyl)carbamate (0.044 g, 0.2 mmol). Purification by column chromatography (15-20% ethyl acetate in hexane) gave **43** as a yellow oil (0.066 g, 56%). **$^1$H NMR (400 MHz, CDCl$_3$)** δ 7.70 (dd, *J* = 16.3, 8.7 Hz, 2H), 7.60 (s, 1H), 7.49 – 7.37 (m, 2H), 7.33 (dd, *J* = 8.4, 1.5 Hz, 1H), 7.16 (dd, *J* = 8.9, 2.3 Hz, 1H), 7.12 (s, 1H), 6.88 (d, *J* = 8.9 Hz, 1H), 6.49 (s, 1H), 4.30 – 3.95 (m, 4H), 3.91 (s, 3H), 3.76 (s, 3H), 3.70 – 3.34 (m, 4H), 3.31 (br s, 1H), 1.53 (d, *J* = 6.8 Hz, 3H), 1.47 (s, 9H). **$^{13}$C NMR (101 MHz, CDCl$_3$)** δ 196.9, 172.6, 157.9, 156.5, 153.0, 136.6, 133.6, 131.9, 129.2, 129.2 (2C), 128.0, 125.8, 125.6, 123.3, 119.5, 119.1, 112.2, 105.8, 80.5, 56.6, 55.5, 50.8, 50.3, 44.7, 43.7, 41.5, 28.4, 20.8. **HRMS-ESI** (m/z): calcd for $C_{31}H_{38}N_3O_5S_2$ [M + H]$^+$ 596.2247; found 596.2237.

***tert*-Butyl (3-((dodecylcarbamothioyl)thio)-4-methoxyphenyl)carbamate (44)**

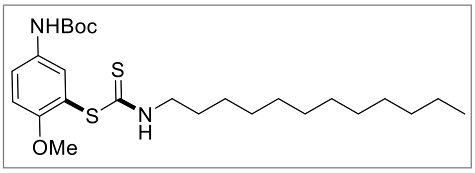


The compound was prepared according to **GP1** using dodecan-1-amine (0.044 g, 0.24 mmol), $CS_2$ (0.037 g, 0.5 mmol) and *tert*-butyl (4-methoxyphenyl)carbamate (0.044 g, 0.2 mmol). Purification by column chromatography (15-20% ethyl acetate in hexane) gave **44** as a yellow oil (0.068 g, 71%). **$^1$H NMR (500 MHz, $CDCl_3$)** δ 7.57 (d, *J* = 7.9 Hz, 1H), 7.47 (d, *J* = 2.4 Hz, 1H), 6.95 (d, *J* = 9.0 Hz, 1H), 6.77 (d, *J* = 4.8 Hz, 1H), 6.57 (s, 1H), 3.83 (s, 3H), 3.66 – 3.55 (m, 2H), 1.49 (s, 9H), 1.24 – 1.14 (m, 20H), 0.86 (t, *J* = 6.9 Hz, 3H). **$^{13}$C NMR (126 MHz, $CDCl_3$)** δ 193.6, 155.4, 153.0, 132.6, 127.8, 124.2, 117.0, 112.7, 81.0, 56.4, 46.5, 32.0, 29.7, 29.7 (2C), 29.6, 29.4, 29.2, 28.4, 28.2, 26.7, 22.8, 14.2. **HRMS-ESI** (m/z): calcd for $C_{25}H_{43}N_2O_3S_2$ $[M + H]^+$ 483.2710; found 483.2704.

**5-((((2-Isopropyl-5-methylcyclohexyl)oxy)carbonyl)amino)-2-methoxyphenyl piperidine-1-carbodithioate (45)**

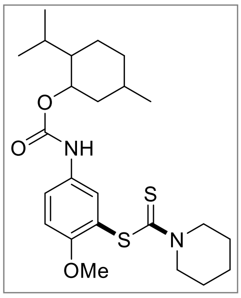


The compound was prepared according to **GP1** using piperidine (0.020 g, 0.24 mmol), $CS_2$ (0.037 g, 0.5 mmol) and 2-isopropyl-5-methylcyclohexyl (4-methoxyphenyl)carbamate (0.060 g, 0.2 mmol). Purification by column chromatography (15-20% ethyl acetate in hexane) gave **45** as a yellow oil (0.082 g, 89%). **$^1$H NMR (500 MHz, $CDCl_3$)** δ 7.57 (s, 1H), 7.41 (s, 1H), 6.92 (d, *J* = 8.9 Hz, 1H), 6.61 (s, 1H), 4.67 – 4.56 (m, 1H), 4.24 (s, 2H), 3.99 (s, 2H), 3.82 (s, 3H), 1.14 – 1.04 (m, 1H), 1.99 – 1.90 (m, 1H), 1.71 (br s, 6H), 1.69 – 1.64 (m, 3H), 1.54 – 1.44 (m, 1H), 1.35 – 1.30 (m, 1H), 1.10 – 1.02 (m, 1H), 1.01 – 0.94 (m, 1H), 0.90 (d, *J* = 3.1 Hz, 3H), 0.88 (d, *J* = 3.7 Hz, 3H), 0.78 (d, *J* = 6.9 Hz, 3H). **$^{13}$C NMR (126 MHz, $CDCl_3$)** δ 194.9, 156.7, 153.6, 131.6, 129.1, 122.9, 120.2, 112.3, 75.1, 56.6, 53.2, 52.2, 47.3, 41.4, 34.3, 31.4, 26.3, 26.1, 25.4, 24.3, 23.5, 22.1, 20.8, 16.5. **HRMS-ESI** (m/z): calcd for $C_{24}H_{37}N_2O_3S_2$ $[M + H]^+$ 465.2240; found 465.2249.

**5-((((3,7-Dimethyloct-6-en-1-yl)oxy)carbonyl)amino)-2-methoxyphenyl piperidine-1-carbodithioate (46)**

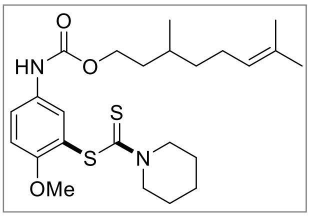


The compound was prepared according to **GP1** using piperidine (0.020 g, 0.24 mmol), $CS_2$ (0.037 g, 0.5 mmol) and 3,7-dimethyloct-6-en-1-yl (4-methoxyphenyl)carbamate (0.061 g, 0.2 mmol). Purification by column chromatography (15-20% ethyl acetate in hexane) gave **46** as a white solid (0.066 g, 72%). **$^1$H NMR (500 MHz, $CDCl_3$)** δ 7.56 (s, 1H), 7.39 (s, 1H), 6.92 (d, *J* = 8.9 Hz, 1H), 6.60 (s, 1H), 5.13 – 5.04 (m, 1H), 4.24 (s, 2H), 4.19 – 4.13 (m, 2H), 4.00 (s, 2H), 3.82 (s, 3H), 2.02 – 1.94 (m, 2H), 1.72 (br s, 6H), 1.67 (br s, 3H), 1.59 (br s, 3H), 1.49 – 1.42 (m, 1H), 1.39 – 1.33 (m, 1H), 1.32 – 1.27 (m, 1H), 1.21 – 1.14 (m, 1H), 0.92 (d, *J* = 6.6 Hz, 3H), 0.87 (t, *J* = 7.1 Hz, 1H). **$^{13}$C NMR (126 MHz, $CDCl_3$)** δ 194.9, 156.9, 154.0, 131.4, 129.5, 124.7 (2C),

123.2, 120.2, 112.3, 63.8, 56.6, 53.3, 52.2, 37.1, 35.9, 29.5, 26.2, 25.8, 25.5 (2C), 24.3, 19.5, 17.7. **HRMS-ESI** (m/z): calcd for $C_{24}H_{37}N_2O_3S_2$ [M + H]$^+$ 465.2240; found 465.2234.

**2-Methoxy-5-(((((3a*R*,6a*R*)-6-methoxy-2,2-dimethyltetrahydrofuro[3,4-*d*][1,3]dioxol-4-yl)methoxy)carbonyl)amino)phenyl piperidine-1-carbodithioate (47)**

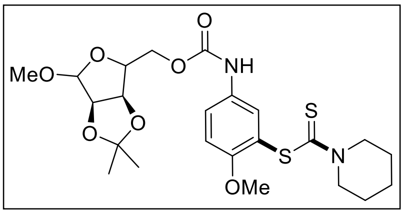


The compound was prepared according to **GP1** using piperidine (0.020 g, 0.24 mmol), $CS_2$ (0.037 g, 0.5 mmol) and ((*3aR,6aR*)-6-methoxy-2,2-dimethyltetrahydrofuro[3,4-d][1,3]dioxol-4-yl)methyl (4-methoxyphenyl)carbamate (0.07 g, 0.2 mmol). Purification by column chromatography (15-20% ethyl acetate in hexane) gave **47** as a yellow oil (0.073 g, 72%). **$^1$H NMR (500 MHz, CDCl$_3$)** δ 7.55 (s, 1H), 7.40 (s, 1H), 6.94 (d, *J* = 8.9 Hz, 1H), 6.66 (s, 1H), 4.99 (s, 1H), 4.70 (d, *J* = 5.5 Hz, 1H), 4.61 (d, *J* = 5.8 Hz, 1H), 4.39 (t, *J* = 6.8 Hz, 1H), 4.30 – 4.20 (m, 3H), 4.18 – 4.14 (m, 1H), 4.01 (br s, 2H), 3.84 (s, 3H), 3.33 (s, 3H), 1.73 (br s, 6H), 1.49 (s, 3H), 1.32 (s, 3H).**$^{13}$C NMR (126 MHz, CDCl$_3$)** 194.8, 157.1, 153.2, 131.0, 129.5, 123.2, 120.3, 112.7, 112.3, 109.4, 85.3, 84.5, 81.8, 65.4, 56.7, 55.1, 53.3, 52.2, 26.5, 26.3, 25.4, 25.1, 24.3. **HRMS-ESI** (m/z): calcd for $C_{23}H_{32}N_2O_7S_2$ [M + H]$^+$ 512.1651; found 512.1658.

***tert*-Butyl (3-((butoxycarbonothioyl)thio)-4-methoxyphenyl)carbamate (48)**

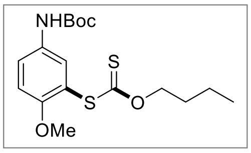


The compound was prepared according to **GP1** using 1-butanol (0.018 g, 0.24 mmol), $CS_2$ (0.037 g, 0.5 mmol) and *tert*-butyl (4-methoxyphenyl)carbamate (0.044 g, 0.2 mmol), Purification by column chromatography (10% ethyl acetate in hexane) gave **48** as a white solid (0.050 g, 67%). **$^1$H NMR (500 MHz, CDCl$_3$)** δ 7.47 – 7.44 (m, 2H), 6.88 (d, *J* = 8.7 Hz, 1H), 6.45 (s, 1H), 4.49 (t, *J* = 6.5 Hz, 2H), 3.81 (s, 3H), 1.66 – 1.59 (m, 2H), 1.50 (s, 9H), 1.30 – 1.22 (m, 2H), 0.86 (t, *J* = 7.4 Hz, 3H). **$^{13}$C NMR (126 MHz, CDCl$_3$)** δ 212.3, 155.5, 152.9, 131.7, 127.1, 122.8, 118.5, 111.8, 80.6, 74.0, 56.2, 30.1, 28.3, 18.9, 13.6. **HRMS-ESI** (m/z): calcd for $C_{17}H_{26}NO_4S_2$ [M + H ]$^+$ 372.1298; found 372.1295.

## 7. Gram-Scale synthesis of 4: -

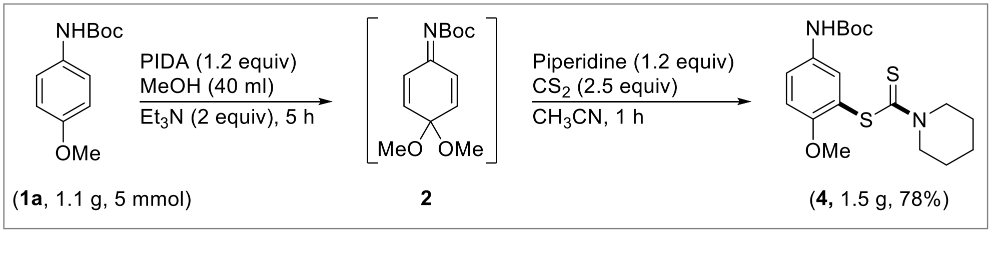


To a stirred solution of **1a** (1.1 g, 5 mmol) in methanol (40 mL) was added (diacetoxyiodo)benzene (PIDA, 1.93 g, 1.2 equiv) and $Et_3N$ (1 g, 2.0 equiv) at 0 °C. The reaction mixture was stirred at 0 °C for 1 h and gradually warmed to rt. After complete

consumption of **1a** (monitored by TLC), MeOH was evaporated on a rotatory evaporator, and the residue was dissolved in acetonitrile (40 mL). To this solution, the carbon disulfide (2.5 equiv, 0.5 mmol, 0.952 g) and piperidine **3a** (0.5 g, 1.2 equiv, 0.24 mmol) were added, and the reaction mixture was stirred for 1 h at room temperature under $N_2$ atmosphere. After completion of the reaction, acetonitrile was evaporated in vacuo, and the residue was purified by column chromatography using a gradient of 15% ethyl acetate in hexane to afford the corresponding **4** as a white solid (1.5 g, 78%).

**Large-Scale synthesis of Compound 35**:

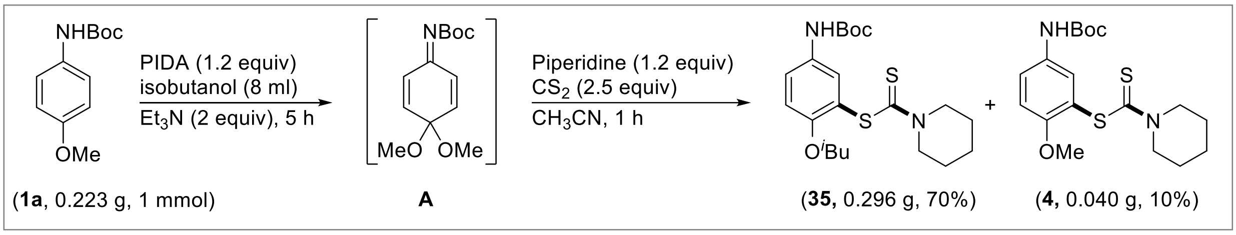


To a stirred solution of **1a** (1.2 mmol, 1.2 equiv) in isobutanol (8 mL) was added (diacetoxyiodo)benzene (PIDA, 1.2 mmol, 1.2 equiv) and $Et_3N$ (2 mmol, 2.0 equiv) at 0 °C. The reaction mixture was stirred at 0 °C for 1 h and gradually warmed to rt. After complete consumption of **1a** (monitored by TLC), isobutanol was evaporated on a rotatory evaporator, and the residue was dissolved in acetonitrile (8 mL). To this solution, the carbon disulfide (2.5 equiv, 2.5 mmol) and piperidine **3a** (1.2 equiv, 1.2 mmol) were added, and the reaction mixture was stirred for 1 h at room temperature under $N_2$ atmosphere. After completion of the reaction, acetonitrile was evaporated in vacuo, and the residue was purified by column chromatography using a gradient of 15% ethyl acetate in hexane to afford the corresponding products **35** (0.296 g, 70%) and **4** (0.040 g, 10%).

# 8. Control Experiment: -

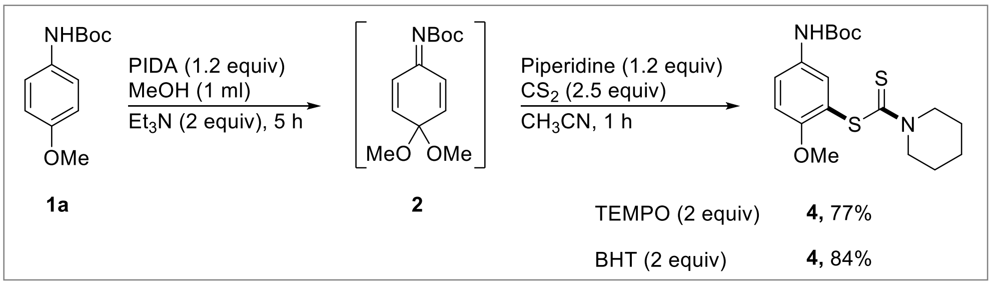


To a stirred solution of **1a** (1.0 equiv, 0.1 mmol) in methanol (1.0 mL) was added (diacetoxyiodo)benzene (PIDA, 39 mg, 1.2 equiv) and $Et_3N$ (23 µL, 2.0 equiv) at 0 °C. The reaction mixture was stirred at 0 °C for 1 h and gradually warmed to rt. After complete consumption of **1a** (monitored by TLC), MeOH was evaporated on a rotatory evaporator, and the residue was dissolved in acetonitrile (1.0 mL). To this solution, the carbon disulfide (2.5 equiv, 0.25 mmol, 18.55 mg), piperidine **3a** (1.2 equiv, 0.12 mmol), TEMPO (2 equiv, 0.2 mmol, 31 mg) were added, and the reaction mixture was stirred for 1 h at room temperature under an $N_2$ atmosphere. After completion of the reaction, acetonitrile was evaporated in vacuo,

and the residue was purified by column chromatography using a gradient of 15% ethyl acetate in hexane to afford the corresponding to afford the corresponding **4** as a white solid (0.029 g, 77%).

To a stirred solution of **1a** (1.0 equiv, 0.1 mmol) in methanol (1.0 mL) was added (diacetoxyiodo)benzene (PIDA, 39 mg, 1.2 equiv) and $Et_3N$ (23 µL, 2.0 equiv) at 0 °C. The reaction mixture was stirred at 0 °C for 1 h and gradually warmed to rt. After complete consumption of **1a** (monitored by TLC), MeOH was evaporated on a rotatory evaporator, and the residue was dissolved in acetonitrile (1.0 mL). To this solution, the carbon disulfide (2.5 equiv, 0.25 mmol, 18.55 mg), piperidine **3a** (1.2 equiv, 0.12 mmol), BHT (2 equiv, 0.2 mmol, 44 mg) were added, and the reaction mixture was stirred for 1 h at room temperature under an $N_2$ atmosphere. After completion of the reaction, acetonitrile was evaporated in vacuo, and the residue was purified by column chromatography using a gradient of 15% ethyl acetate in hexane to afford the corresponding to afford the corresponding **4** as a white solid (0.032 g, 84%).

## 9. Post-synthetic Modification: -

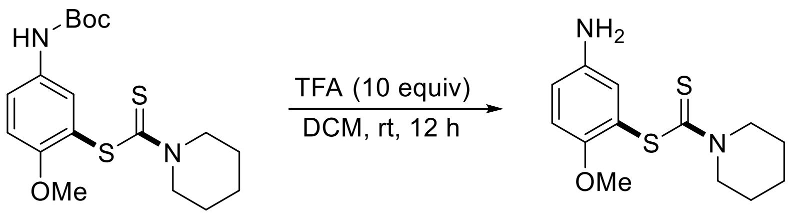


**4** **50**, 89%

Trifluoroacetic acid (2 mmol, 0.228 gm) was added to the solution of **4** (0.2 mmol, 76.5 mg) in DCM (2.0 mL), which was stirred at rt for 12 h. After the completion of the reaction indicated by TLC, the reaction mixture was diluted with aqueous $NaHCO_3$ (5.0 mL) and extracted with DCM (3 × 10.0 mL). The organic fraction was combined and washed with brine and dried over anhydrous $Na_2SO_4$ and concentrated under reduced pressure. The crude residue was purified by column chromatography (1-5% MeOH in DCM) to afford the desired product **50** as a white solid (0.048 g, 89% yield). **$^1$H NMR (500 MHz, CDCl$_3$)** δ 6.85 – 6.70 (m, 3H), 4.23 (br s, 2H), 3.98 (br s, 2H), 3.75 (s, 3H), 3.51 (s, 2H), 1.69 (s, 6H). **$^{13}$C NMR (126 MHz, CDCl$_3$)** δ 195.4, 153.7, 140.4, 125.4, 120.3, 119.1, 113.3, 56.9, 53.2, 52.1, 26.2, 25.4, 24.2. **HRMS-ESI** (m/z): calculated for $C_{13}H_{18}N_2OS_2$ $[M + H]^+$ 283.0933; found 283.0940.

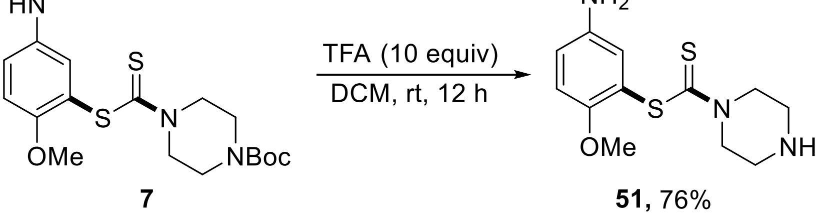


**7** **51**, 76%

Trifluoroacetic acid (2 mmol, 0.228 gm) was added to the solution of **7** (0.2 mmol, 76.5 mg) in DCM (2.0 mL), which was stirred at rt for 12 h. After the completion of the reaction indicated by TLC, the reaction mixture was diluted with aqueous $NaHCO_3$ (5.0 mL) and extracted with DCM (3 × 10.0 mL). The organic fraction was combined and washed with brine and dried over anhydrous $Na_2SO_4$ and concentrated under reduced pressure. The crude residue was purified by column chromatography (1-5% MeOH in DCM) to afford the desired product **51** as a white solid (0.043 g, 76% yield). **$^1$H NMR (500 MHz, DMSO)** δ 6.82 – 6.70 (s, 1H), 6.72 – 6.62 (m, 1H), 6.57 (s, 1H), 4.44 (br s, 1H), 4.09 (br s, 2H), 3.91 (br s, 2H), 3.60 (s, 3H), 2.75 (br s, 4H). **$^{13}$C NMR (126 MHz, DMSO)** δ 194.9, 152.0, 143.1, 124.0, 119.4, 118.0, 114.0, 56.9, 53.1, 52.7, 46.0, 45.8. **HRMS-ESI** (m/z): calcd for $C_{12}H_{17}N_3OS_2$ $[M + H]^+$ 284.0886; found 284.0893.

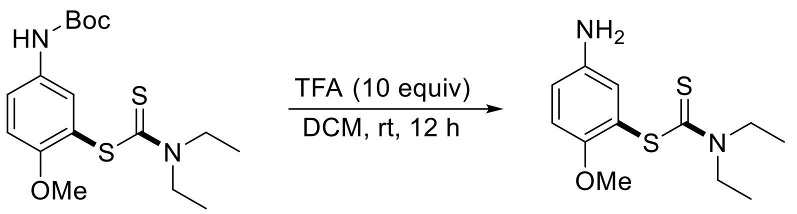


**10** **52,** 86%

Trifluoroacetic acid (2 mmol, 0.228 gm) was added to the solution of **10** (0.2 mmol, 76.5 mg) in DCM (2.0 mL), which was stirred at rt for 12 h. After the completion of the reaction indicated by TLC, the reaction mixture was diluted with aqueous $NaHCO_3$ (5.0 mL) and extracted with DCM (3 × 10.0 mL). The organic fraction was combined and washed with brine and dried over anhydrous $Na_2SO_4$ and concentrated under reduced pressure. The crude residue was purified by column chromatography (1-5% MeOH in DCM) to afford the desired product **52** as a white solid (0.046 g, 86% yield). **$^1$H NMR (400 MHz, $CDCl_3$)** δ 6.95 – 6.68 (m, 3H), 4.01 (dd, $J$ = 14.9, 7.2 Hz), 3.86 (dd, $J$ = 14.7, 7.1 Hz), 3.76 (s, 3H), 3.46 (s, 2H), 1.46 – 1.33 (m, 2H), 1.30 – 1.20 (m, 2H). **$^{13}$C NMR (101 MHz, $CDCl_3$)** δ 195.5, 153.8, 140.4, 125.5, 125.5, 120.6, 119.1, 113.5, 113.5, 113.5, 57.1, 49.8, 47.5, 12.9, 11.8.

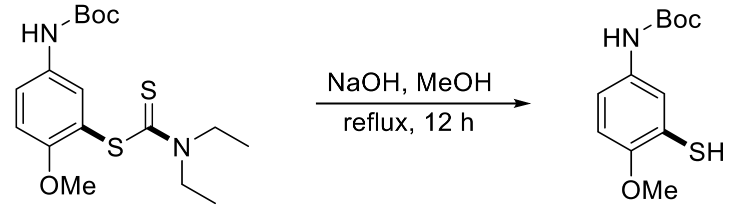


**10** **53,** 86%

To a stirred solution of **10** (92.63 mg, 0.25 mmol, 1 equiv) and methanol (5 mL). The vial was sealed with a septum cap, and a nitrogen balloon was attached. Nitrogen was bubbled through the reaction mixture for 15 minutes, and then NaOH (3M solution in methanol) was added. The reaction mixture was stirred at 60 °C for 12 h, cooled to room temperature and acidified to pH 5 with aqueous HCl solution (10%). The extracted EtOAc were separated, and the aqueous layer was extracted with (2 x 5 mL). All organic layers were combined, washed with saturated

NaCl solution (1 x 10 mL), and dried over anhydrous $Na_2SO_4$. The solvent was removed under reduced pressure to yield the desired product **53** as a yellow oil (0.046 g, 72%). **$^1$H NMR (500 MHz, $CDCl_3$)** δ 7.33 (s, 1H), 7.04 (d, *J* = 8.3 Hz, 1H), 6.75 (d, *J* = 8.8 Hz, 1H), 6.35 (s, 1H), 3.84 (s, 3H), 3.82 (s, 1H), 1.50 (s, 9H). **$^{13}$C NMR (126 MHz, $CDCl_3$)** δ 153.1, 151.0, 131.9, 121.4, 120.17, 117.0, 111.1, 80.6, 56.3, 28.5.

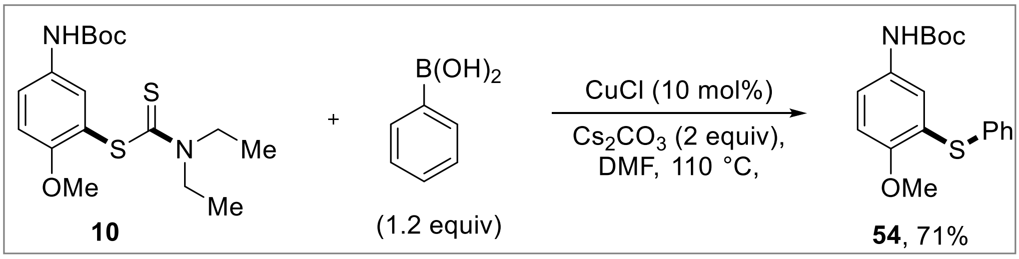


A pre-dried Schlenk-tube was charged with copper(I) chloride (2 mg, 10 mol%), *tert*-butyl (3-((diethylcarbamothioyl)thio)-4-methoxyphenyl)carbamate (74 mg, 0.2 mmol, 1 equiv), and phenyl boronic acid (29 mg, 0.24 mmol, 1.2 equiv). The tube was evacuated and backfilled with nitrogen 3 times. Then a solution of $Cs_2CO_3$ (130 mg, 0.4 mmol, 2 equiv) in DMF (2 ml) was added and the resulting reaction mixture was allowed to stir at 110 °C for 2 h. After completion, the reaction mixture was cooled to room temperature and quenched by the addition of sat. $NaHCO_3$ (5 ml). The resulting mixture was extracted with DCM (5 mL × 3), combined organic layers was dried over anhydrous $Na_2SO_4$, and concentrated under vacuum. The crude residue was purified by column chromatography (10% Ethyl acetate in hexane) to yield the corresponding thioether **54** as a yellow oil (0.047 g, 71%). **$^1$H NMR (500 MHz, $CDCl_3$)** δ 7.51 – 7.38 (m, 1H), 7.35 (d, *J* = 7.2 Hz, 2H), 7.31 (t, *J* = 7.3 Hz, 2H), 7.27 (d, *J* = 6.2 Hz, 1H), 6.89 – 6.79 (m, 2H), 6.27 (s, 1H), 3.83 (s, 3H), 1.46 (s, 9H). **$^{13}$C NMR (101 MHz, $CDCl_3$)** δ 153.5, 132.1, 131.7, 129.3, 129.3, 129.3, 127.3, 122.6, 119.4, 111.5, 80.4, 56.4, 28.4.

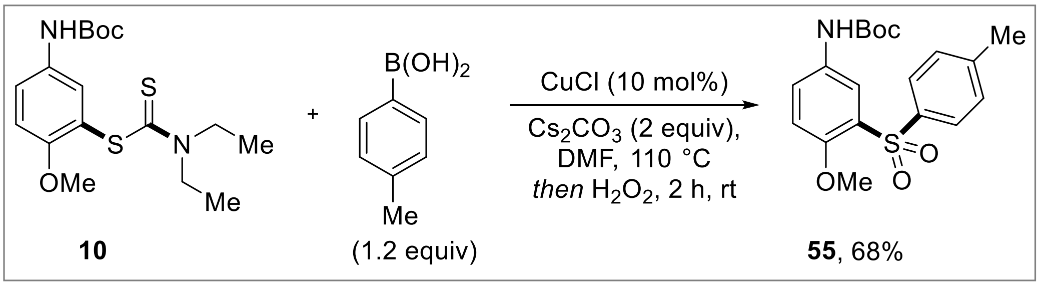


A pre-dried Schlenk-tube was charged with copper(I) chloride (2 mg, 10 mol%), *tert*-butyl (3-((diethylcarbamothioyl)thio)-4-methoxyphenyl)carbamate (74 mg, 0.2 mmol, 1 equiv), and phenyl boronic acid (29 mg, 0.24 mmol, 1.2 equiv). The tube was evacuated and backfilled with nitrogen 3 times. Then a solution of $Cs_2CO_3$ (130 mg, 0.4 mmol, 2 equiv) in DMF (2 ml) was added and the resulting reaction mixture was allowed to stir at 110 °C for 2 h. After completion, the reaction mixture was cooled to room temperature and quenched by the addition of sat. $NaHCO_3$ (5 ml). The resulting mixture was extracted with DCM (5 mL × 3), combined organic layers were dried over anhydrous $Na_2SO_4$, and concentrated under vacuum. The crude

residue was dissolved in water, and $H_2O_2$ (20 mg, 0.6 mmol, 3 equiv) was added to the reaction for 6 h. Then the reaction mixture was purified by column chromatography (25% Ethyl acetate in hexane) to yield the desired sulfones **55** as a pale yellow semi-solid (0.047 g, 71%). **$^1$H NMR (500 MHz, $CDCl_3$)** δ 7.97 (s, 1H), 7.83 (d, *J* = 7.8 Hz, 3H), 7.25 (d, *J* = 7.6 Hz, 2H), 6.94 (d, *J* = 5.7 Hz, 1H), 6.82 (d, *J* = 8.8 Hz, 1H), 3.70 (s, 3H), 2.39 (s, 3H), 1.48 (s, 9H). **$^{13}$C NMR (126 MHz, $CDCl_3$)** δ 153.2, 152.8, 144.0, 138.3, 132.0, 129.2, 128.5, 127.0, 126.1, 120.4, 113.4, 80.8, 56.3, 28.4, 21.7.

## 10. Crystallographic data of 5-((*tert*-butoxycarbonyl)amino)-2-methoxyphenyl piperidine-1-carbodithioate 4:-

The crystal was prepared by slow evaporation of the compound **4** in the solution of ethanol.

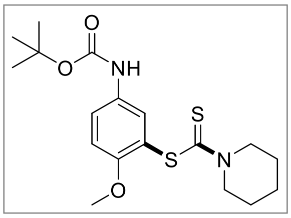


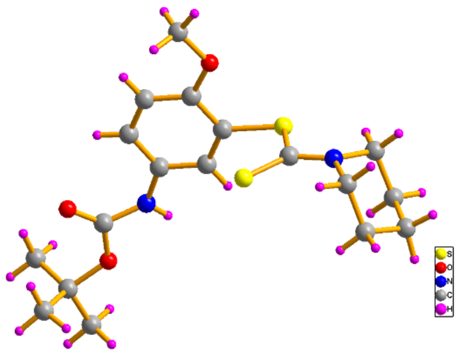

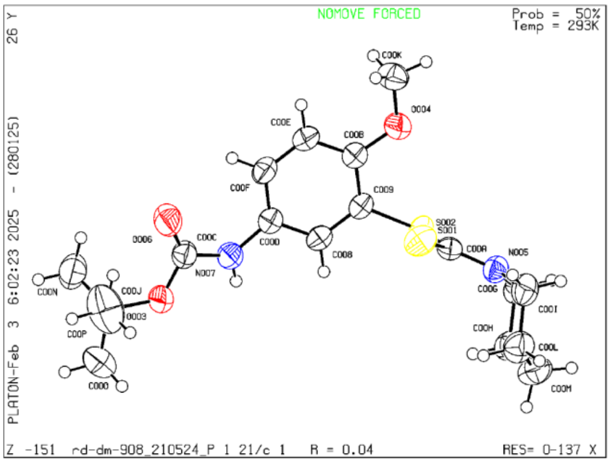


# Crystal structure data table of 4

| | |
|---|---|
| Empirical formula | $C_{18}H_{26}N_2O_3S_2$ |
| CCDC No | 2420899 |
| Formula weight | 382.53 |
| Temperature/K | 293(2) |
| Crystal system | monoclinic |
| Space group | $P2_1/c$ |
| a/Å | 17.0964(5) |
| b/Å | 6.6792(2) |

| | |
|---|---|
| c/Å | 18.6716(6) |
| α/° | 90 |
| β/° | 109.331(3) |
| γ/° | 90 |
| Volume/$Å^3$ | 2011.91(11) |
| Z | 4 |
| $\rho_{calc}$g/$cm^3$ | 1.263 |
| μ/$mm^{-1}$ | 0.283 |
| F(000) | 816.0 |
| Crystal size/$mm^3$ | 0.2 × 0.18 × 0.17 |
| Radiation | MoKα (λ = 0.71073) |
| 2Θ range for data collection/° | 4.474 to 54.16 |
| Index ranges | -17 ≤ h ≤ 21, -8 ≤ k ≤ 8, -23 ≤ l ≤ 22 |
| Reflections collected | 17705 |
| Independent reflections | 4209 [$R_{int}$ = 0.0243, $R_{sigma}$ = 0.0267] |
| Data/restraints/parameters | 4209/0/234 |
| Goodness-of-fit on $F^2$ | 1.096 |
| Final R indexes [I>=2σ (I)] | $R_1$ = 0.0377, $wR_2$ = 0.0933 |
| Final R indexes [all data] | $R_1$ = 0.0559, $wR_2$ = 0.0997 |
| Largest diff. peak/hole / e $Å^{-3}$ | 0.17/-0.23 |

## 11. Unsuccessful Substrates:

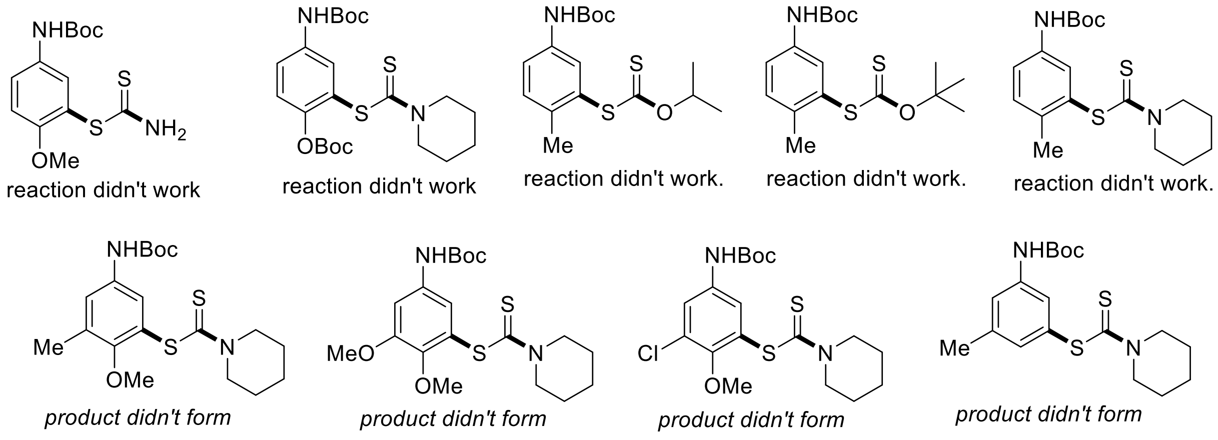


**Scheme S1. Unsuccessful Substrates**

## 12. Biological Materials and Methods

### Cell culture and reagents

The MCF7 were cultured in DMEM medium (Himedia, #AL111) supplemented with 10% FBS (Himedia #RM10434, #RM10432 respectively), 1% penicillin/streptomycin (Sigma, #P4333), and 1% L-glutamine (Sigma, #G7513) in a humidified incubator with 5% $CO_2$ at 37 °C.

### MTT Assay

Approximately 3000 cells were seeded per well in a 96-well tissue culture plate (Tarsons, #980040) and allowed to settle overnight. The next day, the compound was added at 0.01, 0.025, 0.05, 0.1, 0.25, 0.5, 1.0, 2.5, 5.0 and 10.0 μM concentrations. Each condition was performed in triplicates. DMSO was added to three wells as a control, and three wells were left untreated, which were used as background control. The cells were treated for 72 hours at 37 °C in a 5% $CO_2$ atmosphere. Then, 10 μl of MTT (3-(4,5- dimethylthiazol-2-yl)-2,5-diphenyl tetrazolium bromide; Invitrogen, #M6494) stock solution prepared in PBS at 5 mg/ml was added to each well in 100 μl serum-free medium. The plate was incubated at 37 °C in a humidified incubator chamber for 4 hours. Acidic isopropanol (100μl) was added to each well and mixed thoroughly in a shaker to dissolve the purple formazan crystals. The absorbance was measured using a

microplate reader (Biotek Synergy H1) at a wavelength of 570 nm. The results were analyzed in Origin using a dose-response curve on a Windows 10 PC and plotted in Microsoft excel.

**Molecular Docking**

SwissDock online docking server (Grosdidier et al., 2011) was employed to predict the atomic contact energy and interaction interface area of receptor tubulin (PDB ID: 6I2I) with ligands (Cmpd **7**, Cmpd **13** and paclitaxel). The top-scoring model was analyzed for binding of the ligands to the taxol binding pocket and visualized using UCSF Chimera version 1.14. Contact maps were generated using BIOVIA Discovery Studio molecular visualization software.

**Microtubule Bundling Assay**

Cells were plated at a confluency of 50-60 % on poly-D lysine-coated coverslips and treated with DMSO (control), Paclitaxel (Spectrochem, #011615), Compound **7**, Compound **12** and Compound **13** at 0.5 μM for 24 hours. The coverslips were washed thrice with 1x PBS and permeabilized using 0.1% Triton-X in 1xPBS for 2 minutes. This was followed by fixation with 4% (v/v) paraformaldehyde solution in 1x PBS for 10 minutes at room temperature. The fixing solution was removed, and the coverslip was washed using 1x PBS. 1% BSA in PBST was used for blocking the coverslips for 1 hour. Anti-α- tubulin antibody (Sigma, #T6074, 1:200) was added and left overnight at 4 $^{\circ}$C. The next day, the coverslips were washed with 1x PBS and incubated with anti-mouse IgG TRITC (Sigma, #SAB3701020, 1:500) in the blocking solution for 1 hour. The nuclei were stained by incubating with 0.5 μg/ml DAPI solution in 1x PBS. The coverslip was mounted on a glass slide using DABCO anti-fade solution. The images were acquired using a 100X objective lens of epifluorescence microscopy. The images were analyzed in ImageJ v 1.53 using plug-in LPX (Higaki, 2017) which provides i-skewness of the tubulin fluorescence intensity distribution.

**Microtubule Polymerization Assay using Goat-Tubulin**

Tubulin was purified from goat brain using the protocol by Souphron et al. (Souphron et al., 2019). 50 uL reactions consisting of purified goat-tubulin (0.5 mg/ml), 1xBRB80 buffer (Na-PIPES 80 mM, EGTA 1mM, $MgCl_2$ 1 mM, DTT 1mM, titrate to pH 6.8 using KOH), 0.75 mM

GTP and 10 uM compound was set up. The solution was prepared after thawing tubulin on ice for 30 mins. Drop cast method was used for this assay where the reactions were added as a drop on top of a slide and left undisturbed for 10-20 seconds. A drop of filtered autoclaved water was added to each reaction for 10 seconds and the solutions were discarded. Slides were transferred to 37 ºC till the slide was completely dry. Imaging was performed under DIC using the IX83 Inverted Fluorescence Microscope. Images were taken at 10X magnification.

**Tubulin Polymerization using turbidity assay**

Recombinant tubulin was isolated from goat brain using the protocol by Souphron et al. (Souphron et al., 2019). 100ul reactions were prepared that consisted of tubulin (9 mg/ml) in 1× BRB80 buffer (80 mM Na-PIPES, 1 mM EGTA, 1 mM $MgCl_2$, 1 mM DTT, pH 6.8) and 0.75 mM GTP and 20 uM compound (Paclitaxel/Cmpd **7**/Cmpd **57**/Cmpd **13**). Reaction without drug was taken as control. The reactions were set up in a 96-well UV-transparent plate (Corning, Cat. No. 3635) and placed in a preheated the plate reader to 37 °C. The absorbance at 350 nm was recorded every 30 seconds for 1 hour using Synergy H1 multi-Mode microplate reader. The graph was plotted using Origin.

**Flow cytometry analysis**

MCF7 cells seeded at 70% confluency were treated with parent compound and compound **13** at their respective $IC_{50}$ and 2X $IC_{50}$ values. After 72 hours of drug treatment the cells were harvested by centrifugation at 5000 rcf for 5 minutes followed by two washes with 1x PBS. Cells were then fixed using ice-cold 70% ethanol for 1 hour at −20 °C. Post-fixation, the cells were centrifuged at 800 rcf for 5 minutes at 4 °C and washed twice with 1x PBS. Pellets were then dissolved in 400 µl of staining solution (50 mg/ml propidium iodide and 100 mg/ml RNAse-A prepared in 1x PBS) and incubated at room temperature for 1 hour. Analysis of the DNA content was done using flow cytometry (BD FACS Discover S8), and the data was analysed using FlowJo software.

**Western blotting**

The MCF cells at a confluency of 40-50% were treated with compound **13** at an $IC_{50}$ concentration of 0.13 µM for 72 hours. The cells were lysed in 1x RIPA buffer ( 50 mM Tris pH8, 150 mM NaCl, 5 mM EDTA, 1% NP40, 0.5% Sodium Deoxycholate, 0.1 % SDS +

PMSF, protease and phosphatase inhibitors) and separated on 10 % SDS-PAGE. This was followed by transfer to a nitrocellulose membrane (Biorad) for western blotting. Briefly, the membrane was blocked in 5% BSA in 1x PBST (0.05% tween-20) for 2 h at room temperature with shaking. This was followed by overnight incubation of membrane at 4 °C with the relevant primary antibody against rabbit cyclinB1 (1:1000; CST#4138S), mouse phosphorylated-p53 (1:1000; CST#9286T), and mouse α-tubulin (1:1000; Merck#T6074). The membrane was washed thrice with 1x PBST for 10 minutes each wash at room temperature under shaking conditions. This was followed by a 1 h treatment with HRP-conjugated relevant secondary antibody at 1:10,000 [anti-mouse (CST#7076S) and anti-rabbit (CST#7074S)] dilution in the blocking solution at room temperature with shaking. The membrane was washed four times with 1x PBS, followed by the last wash with 1x PBS. The blot was developed using Clarity™ Western Enhanced Chemiluminescent Substrate (Bio-Rad Laboratories, Inc.#1705060;.) and visualized by a gel documentation system.

**Table S1**: ***in silico* ADMET analysis:** ADMET properties of drugs paclitaxel (PTX), compound **7** and **13** based on *in silico* prediction using ProTox 3.0, ADMETsar, ADMETlab 2.0. The prediction probability is given in the brackets for each property.

| **Property** | **Cmpd 7** | **Cmpd 13** | **PTX** |
|---|---|---|---|
| **Absorption** | | | |
| Blood-Brain Barrier | BBB+ (0.76) | BBB+ (0.74) | BBB- (0.97) |
| Human Intestinal Absorption | HIA- (0.51) | HIA+ (0.93) | HIA+ (0.91) |
| Caco-2 Permeability | Caco-2 (0.52) | Caco-2 (0.52) | Caco-2 (0.89) |
| P-glycoprotein Substrate | Substrate (0.71) | Non-substrate (0.68) | Substrate (0.83) |
| P-glycoprotein Inhibitor | Inhibitor (0.79) | Non-inhibitor (0.52) | Inhibitor (0.55) |
| Renal Organic Cation Transporter | Non-inhibitor (0.74) | Non-inhibitor (0.86) | Non-inhibitor (0.93) |
| **Distribution** | | | |
| Subcellular Localization | Lysosome (0.59) | Mitochondria (0.8) | Mitochondria (0.65) |
| Metabolism | | | |
| CYP450 2C9 Substrate | Non-substrate (0.8) | Non-substrate (0.70) | Non-substrate (0.83) |
| CYP450 3A4 Substrate | Substrate (0.66) | Substrate (0.57) | Substrate (0.72) |
| CYP Inhibitory Promiscuity | Low (0.57) | High (0.93) | Low (0.89) |
| **Excretion** | | | |

| | | | |
|---|---|---|---|
| Biodegradation | Not biodegradable (1) | Not biodegradable (1) | Not biodegradable (0.94) |
| **Toxicity** | | | |
| AMES Toxicity | Non-toxic (0.68) | Non-toxic (0.58) | Non-toxic (0.91) |
| Carcinogens | Non-carcinogen (0.84) | Non-carcinogen (0.68) | Non-carcinogen (0.91) |
| Fish Toxicity | High FHMT (0.98) | High FHMT (0.99) | High FHMT (0.98) |
| Tetrahymena Pyriformis Toxicity | High TPT (0.85) | High TPT (0.90) | High TPT (0.99) |
| Acute Oral Toxicity | III (0.59) | III (0.45) | III (0.59) |
| **Organ Toxicity** | | | |
| Hepatotoxicity | Inactive (0.68) | Inactive (0.53) | Inactive (0.63) |
| Neurotoxicity | Active (0.57) | Inactive (0.57) | Active (0.89) |
| Nephrotoxicity | Inactive (0.55) | Inactive (0.54) | Active (0.79) |
| Respiratory toxicity | Active (0.78) | Active (0.62) | Active (0.95) |
| Cardiotoxicity | Inactive (0.73) | Inactive (0.66) | Inactive (0.55) |
| **Toxicity End Points** | | | |
| Carcinogenicity | Inactive (0.61) | Inactive (0.63) | Inactive (0.61) |
| Immunotoxicity | Inactive (0.85) | Inactive (0.92) | Active (0.99) |
| Mutagenicity | Inactive (0.72) | Inactive (0.6) | Inactive (0.85) |
| BBB-barrier | Active (0.68) | Active (0.63) | Inactive (0.96) |
| Ecotoxicity | Active (0.59) | Active (0.75) | Inactive (0.65) |
| Clinical toxicity | Active (0.53) | Inactive (0.51) | Active (0.83) |

| | | | |
|---|---|---|---|
| Nutritional toxicity | Active (0.59) | Active (0.57) | Active (0.61) |
| **Tox21-Nuclear Receptor Pathways** | | | |
| Aryl hydrocarbon Receptor (AhR) | Inactive (0.92) | Inactive (0.62) | Inactive (0.98) |
| Androgen Receptor (AR) | Inactive (0.89) | Inactive (0.9) | Inactive (0.97) |
| Androgen Receptor Ligand Binding Domain (AR-LBD) | Inactive (0.97) | Inactive (0.92) | Inactive (0.88) |
| Aromatase | Inactive (0.92) | Inactive (0.88) | Inactive (0.95) |
| Estrogen Receptor Alpha (ER) | Inactive (0.85) | Inactive (0.81) | Inactive (0.87) |
| Estrogen Receptor Ligand Binding Domain (ER-LBD) | Inactive (0.84) | Inactive (0.95) | Inactive (0.98) |
| Peroxisome Proliferator Activated Receptor Gamma | Inactive (0.93) | Inactive (0.9) | Inactive (0.97) |
| **Tox21-Stress Response Pathways** | | | |
| Nuclear factor (nrf2/ARE) | Inactive (0.92) | Inactive (0.91) | Inactive (0.93) |
| Heat shock factor response element (HSE) | Inactive (0.92) | Inactive (0.91) | Inactive (0.93) |
| Mitochondrial Membrane Potential (MMP) | Inactive (0.82) | Inactive (0.64) | Active (0.9) |
| Phosphoprotein (Tumor Suppressor) p53 | Inactive (0.85) | Inactive (0.87) | Active (0.79) |
| ATPase family AAA domain-containing protein 5 | Inactive (0.96) | Inactive (0.93) | Inactive (0.97) |

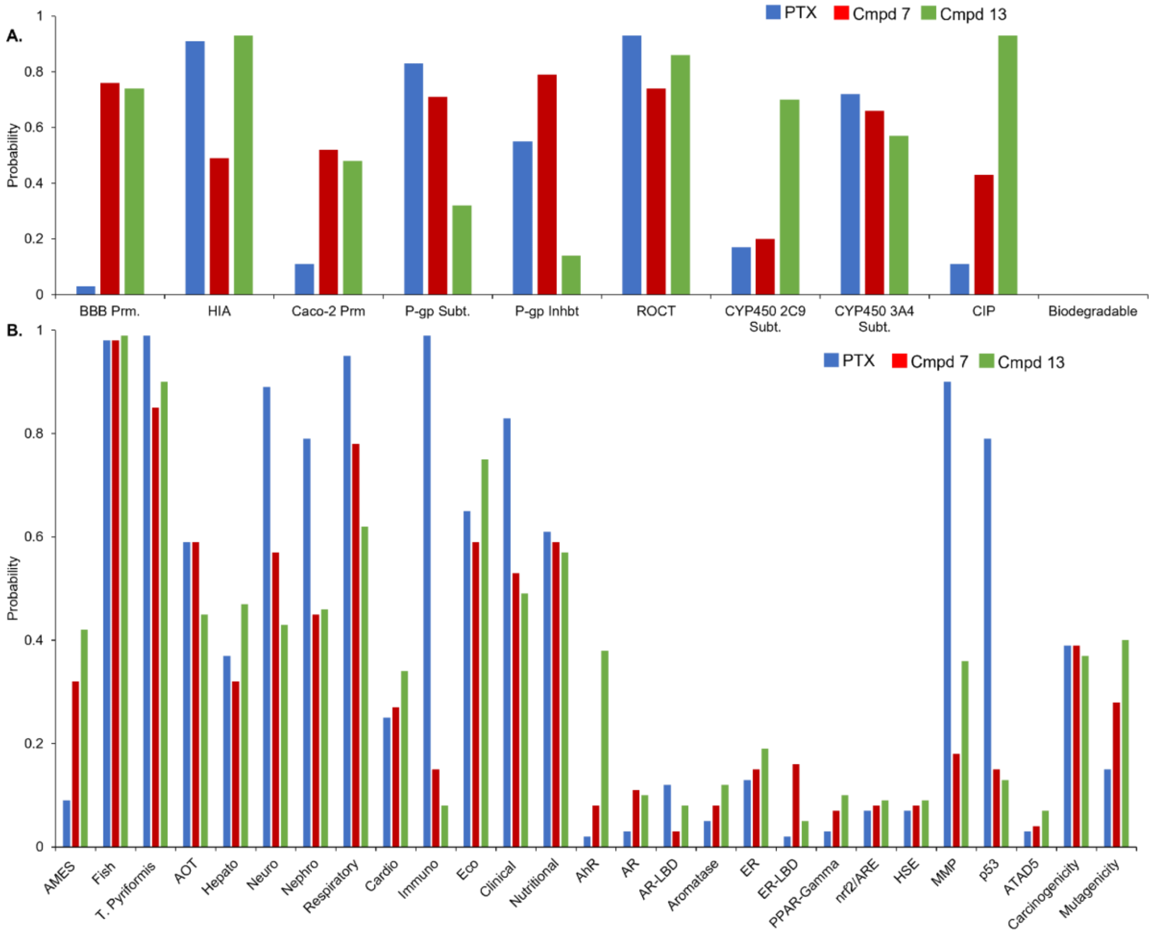


**Figure S1**: **A)** Bar-graph depicting ADME parameters based on predicted probabilities. BBB Prm: Blood brain permeability; HIA: human intestinal absorption; Caco-2 Prm: Caco-2 cell permeability; P-gp Subt: P-glycoprotein substrate; P-gp Inhbt: P-glycoprotein inhibitor; ROCT: Renal Organ cation transporter; CYP450 2C9 Subt: CYP450 2C9 Substrate; CYP450 3A4 Subt: CYP450 3A4 Substrate; CIP: CYP Inhibitory Promiscuity. Data generated using ProTox 3.0, ADMETsar, ADMETlab 2.0 **B)** Bar-graph depicting organ and fish toxicity based on predicted probabilities. T.Pyriformis: *Tetrahymena pyriformis*; AOT: Acute organ toxicity; AhR: Aryl hydrocarbon receptor; AR: Androgen receptor; AR-LBD: Androgen receptor ligand binding domain; ER: Estrogen receptor; ER-LBD: Estrogen receptor ligand binding domain; HSE: heat shock receptor element; MMP: matrix metalloproteinases; ATAD5: ATPase family AAA domain-containing protein 5. Blue (PTX, paclitaxel); Red (compound **7**) and Green (compound **13**).

**Table S2**: Comparison of $IC_{50}$ values in the breast cancer cell line MCF7 with the non-cancer cell line, 3T3L-1 for paclitaxel, compound **7** and **13**.

| Cell line | Compound | $IC_{50}$±SEM | $R^2$ |
|---|---|---|---|
| MCF7 (Breast Cancer cells) | Paclitaxel | 0.01±0.01 | 0.96 |
| | Cmpd **7** | 0.12±0.02 | 0.95 |
| | Cmpd **13** | 0.13±0.01 | 0.95 |
| 3T3L-1 (Non-cancerous preadipocyte cells) | Paclitaxel | 0.3±0.08 | 0.91 |
| | Cmpd **7** | 3.47±0.32 | 0.96 |
| | Cmpd **13** | 1.65±0.2 | 0.97 |

**Table S3**: Comparison of $IC_{50}$ value of lead compound **13** in different cancer cell line.

| Cancer cell line | $IC_{50}$±SEM | $R^2$ |
|---|---|---|
| MCF7 | 0.13±0.01 | 0.95 |
| HeLa | 1.00±0.24 | 0.94 |
| A549 | 3.60±0.30 | 0.90 |

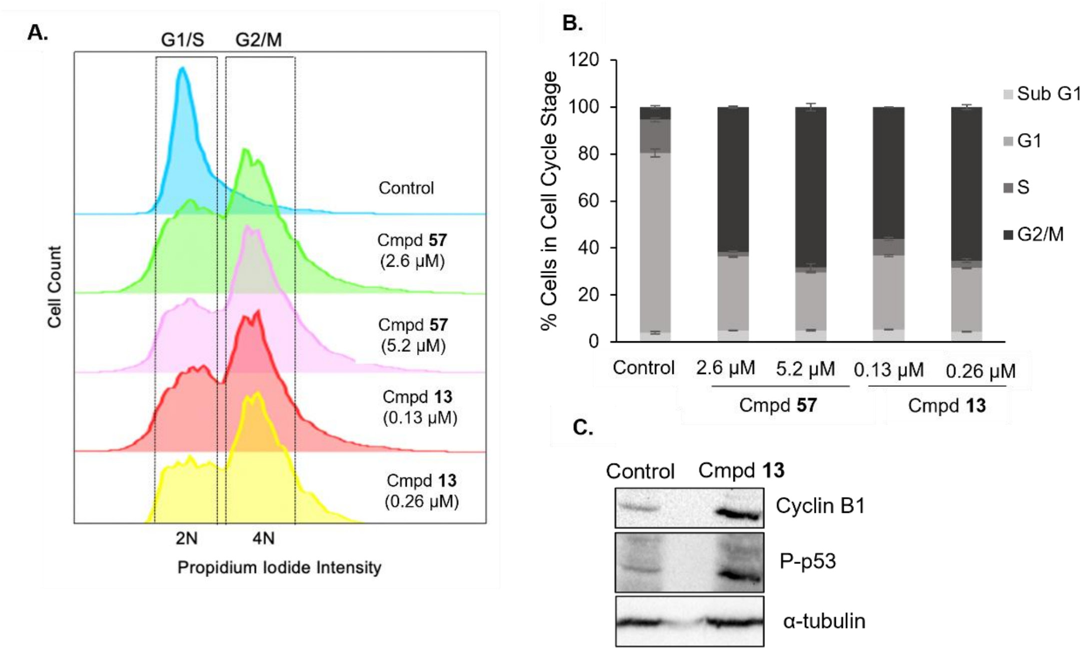


**Figure S2**: Showing flow cytometry analysis of MCF7 cells post-treatment with Compound 56 and 13. (**A**) Cell cycle profile of MCF7 cells treated with the DMSO (vehicle control), Compound 56 at $IC_{50}$ (2.6 µM) and 2*$IC_{50}$ (5.2 µM) and compound **13** at $IC_{50}$ (0.13 µM) and 2*$IC_{50}$ (0.26 µM) doses.( **B**) Bar graph representing the relative percentage of cell populations in the different cell cycle stages (Sub G1, G1, S and G2/M) depicted by analysing the flow cytometry data. (**C**) Western blot showing an increase in G2/M cell cycle marker, Cyclin B1 and activation of checkpoint protein, phosphorylated p53 in cells treated with the lead compound **13** (0.13 µM), compared to vehicle control (DMSO). α-tubulin acts as a loading control.

## 16. References


1. Das, K.; Sarkar, K.; Maji, B. *ACS Catal.* **2021**, *11*, 7060–7069
2. Zhou, Z.; Li, Y.; Han, B.; Gong, L.; Meggers, E. *Chem. Sci.* **2017**, *8*, 5757-5763.
3. Hashimoto, T.; Nakatsu, H.; Takiguchi, Y.; Maruoka, K. *J. Am. Chem. Soc.* **2013**, *135*, 16010.
4. Bodipati, N.; Peddinti, R. K. *Org. Biomol. Chem.* **2012**, *10*, 4549.
5. Souphron, J.; Bodakuntla, S.; Jijumon, S. A.; Lakisic, G.; Gautreau, A. M; Janke, C.; Magiera, M. M. *Nat. Protoc.* **2019**, *14*, 1634.
6. Grosdidier, A.; Zoete, V.; Michielin, O. *Nucleic Acids Research*, **2011**, *39*, W270-W277.

## 17. NMR Spectra

**$^{1}H$ and $^{13}C$ NMR of 4**

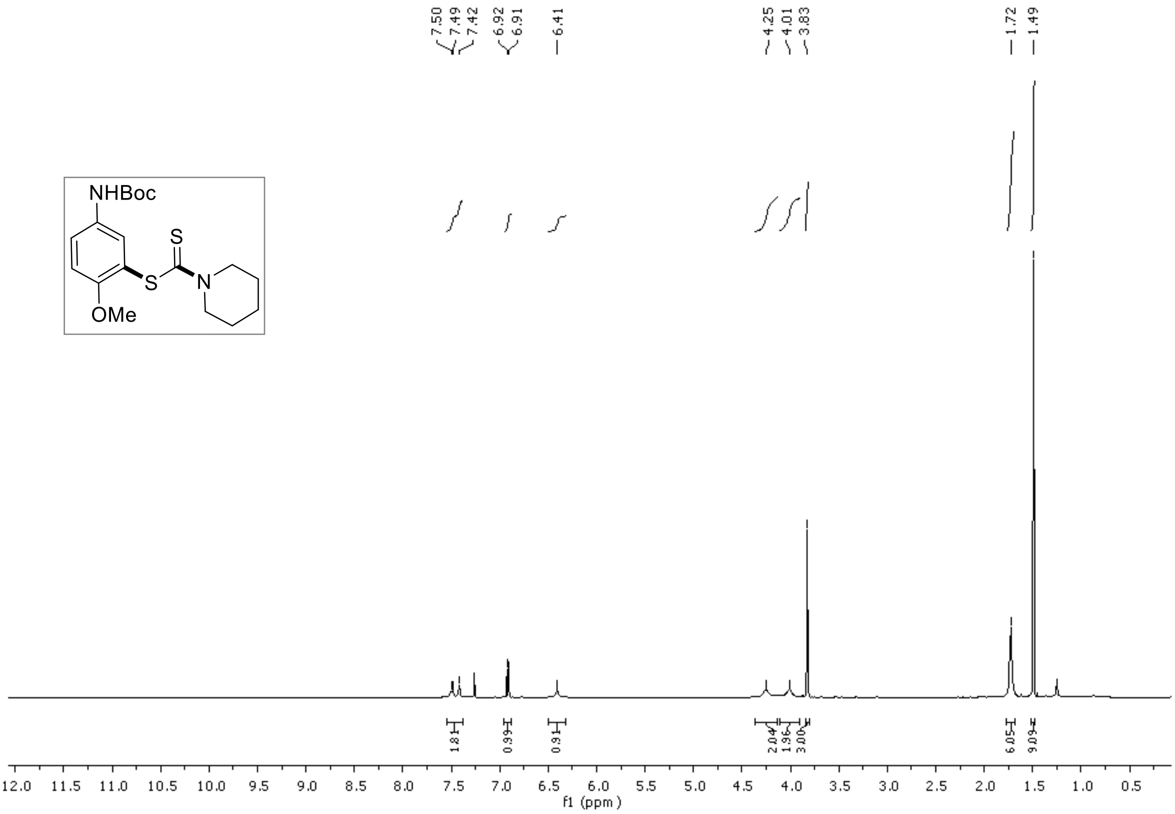


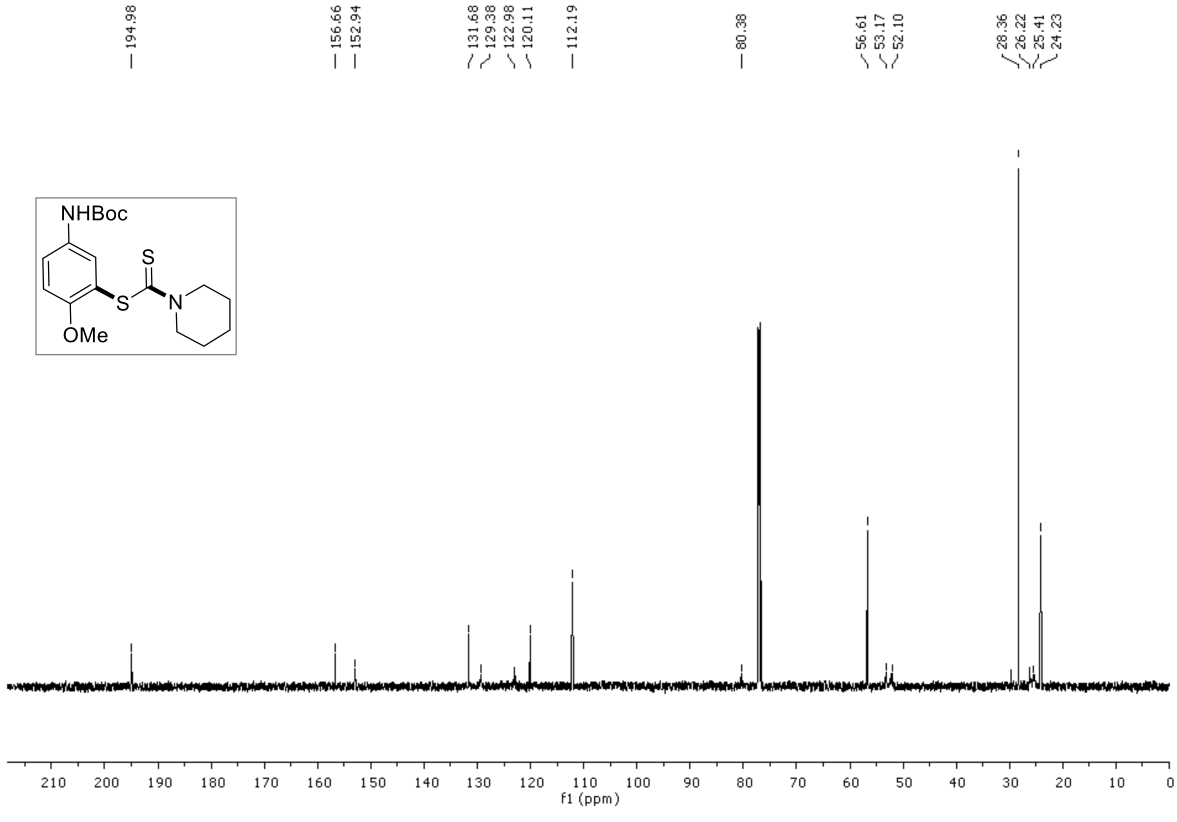

## $^{1}$H and $^{13}$C NMR of 5

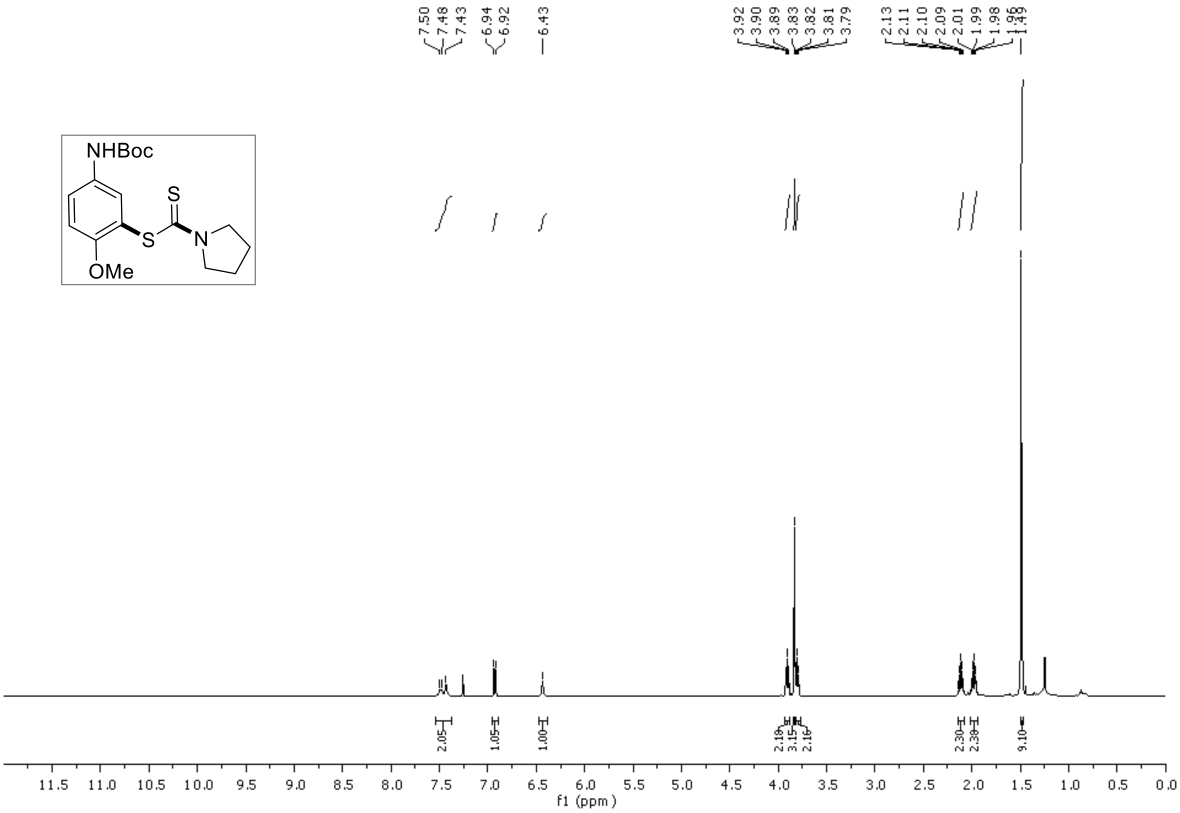


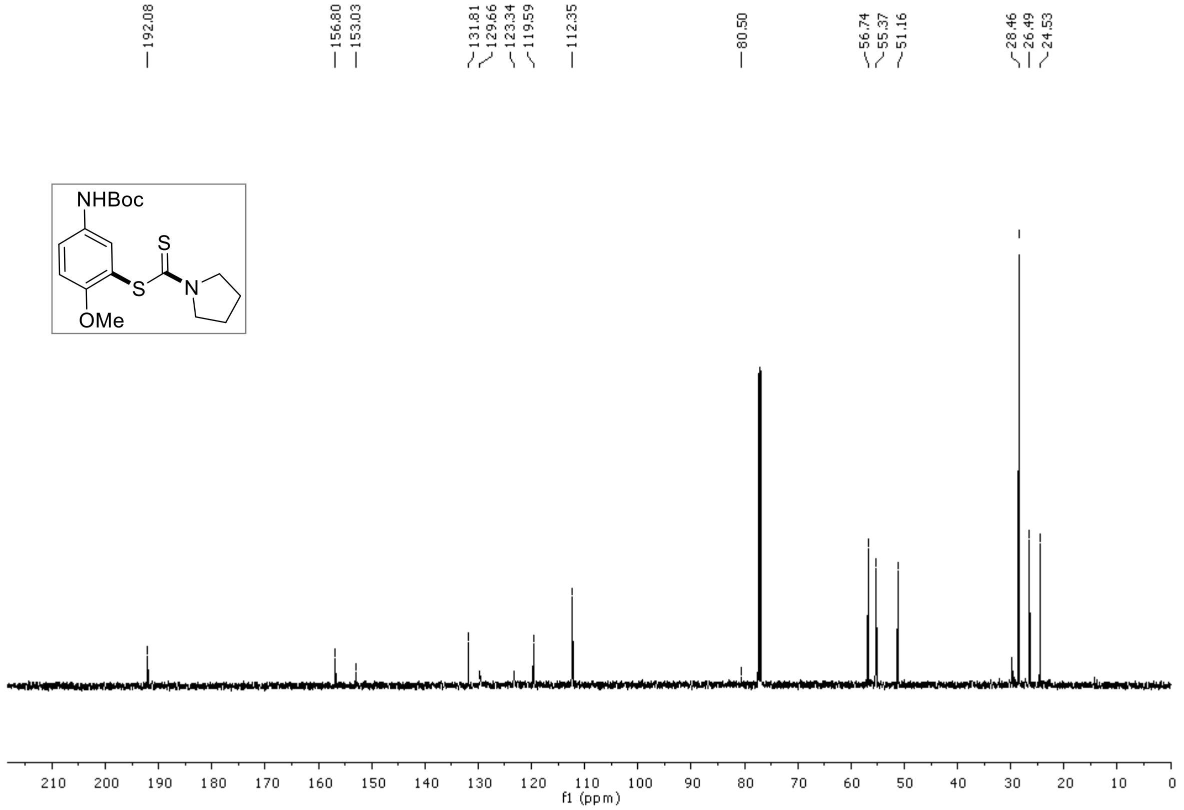

## $^{1}$H and $^{13}$C NMR of 6

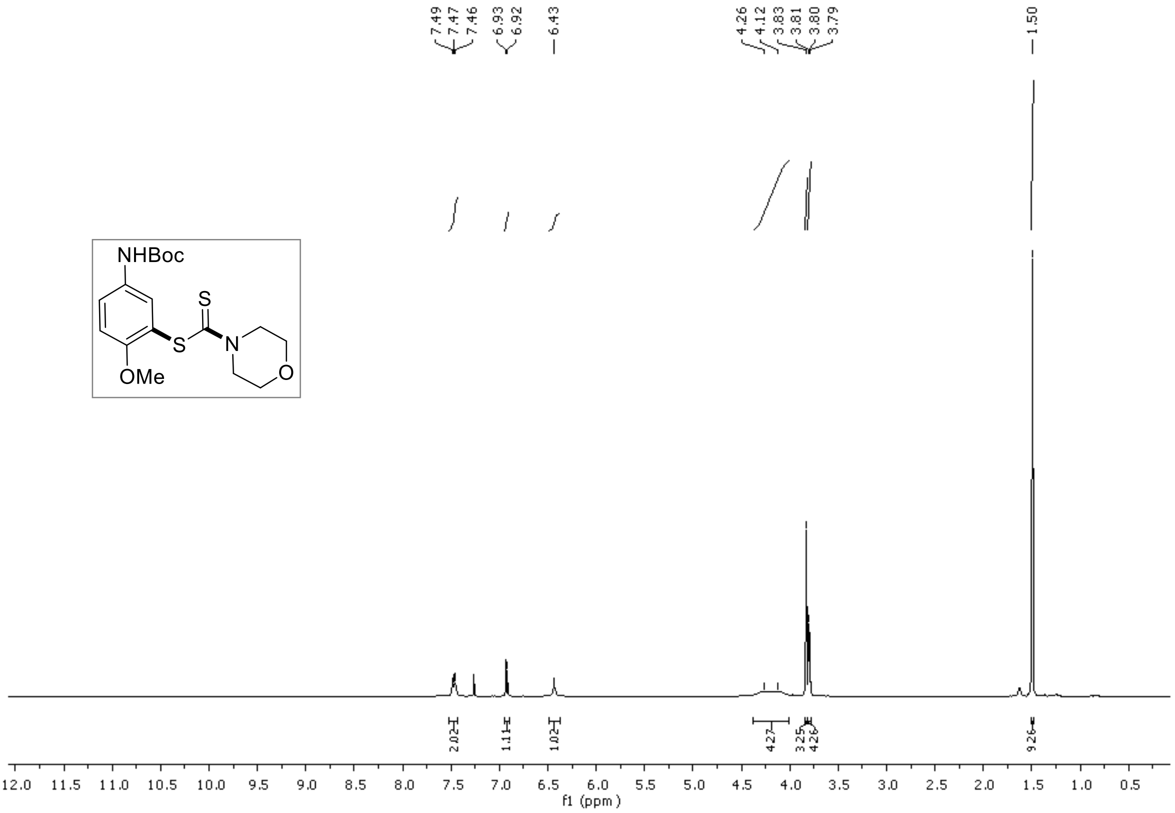


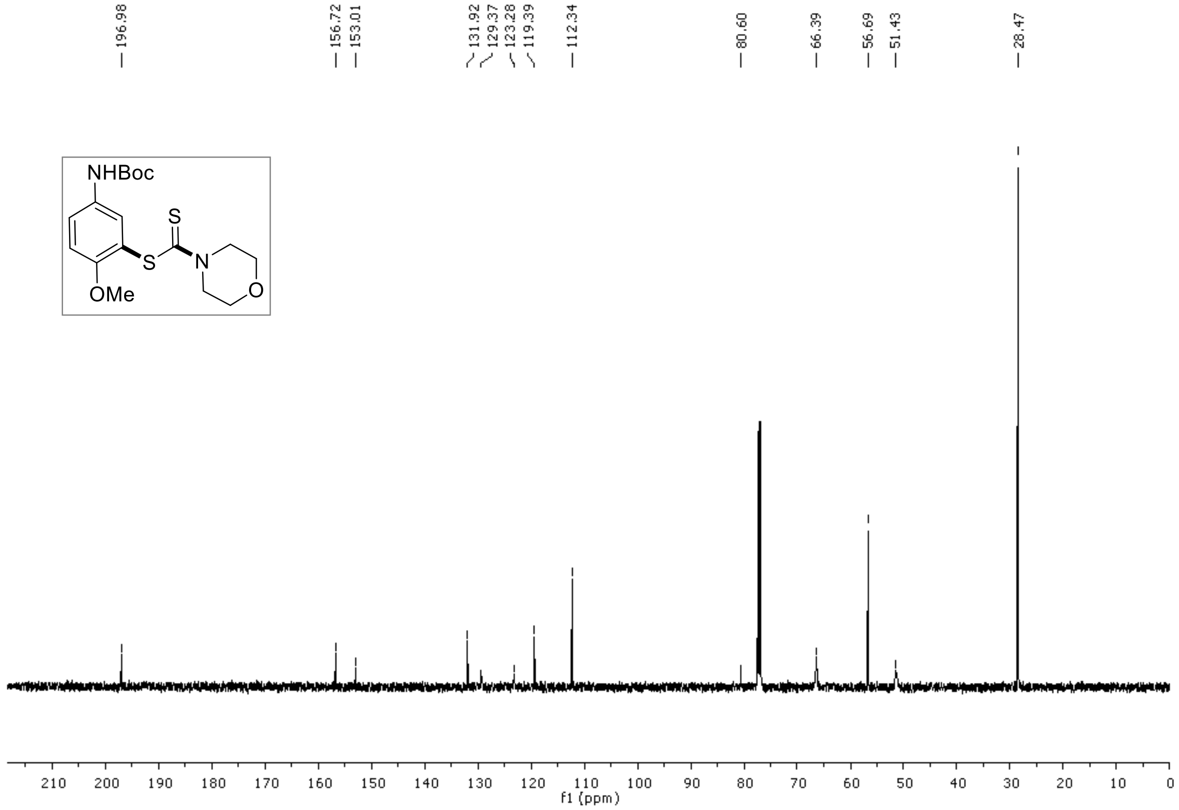

## $^{1}H$ and $^{13}C$ NMR of 7

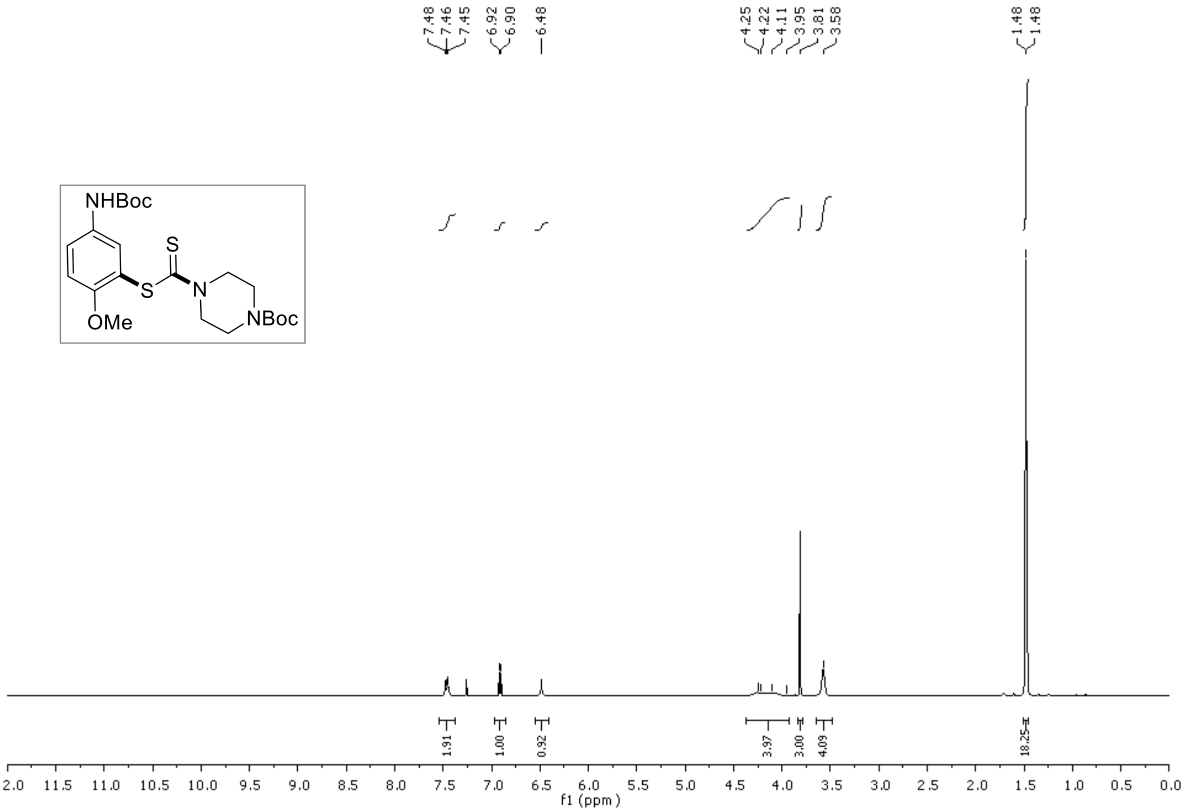

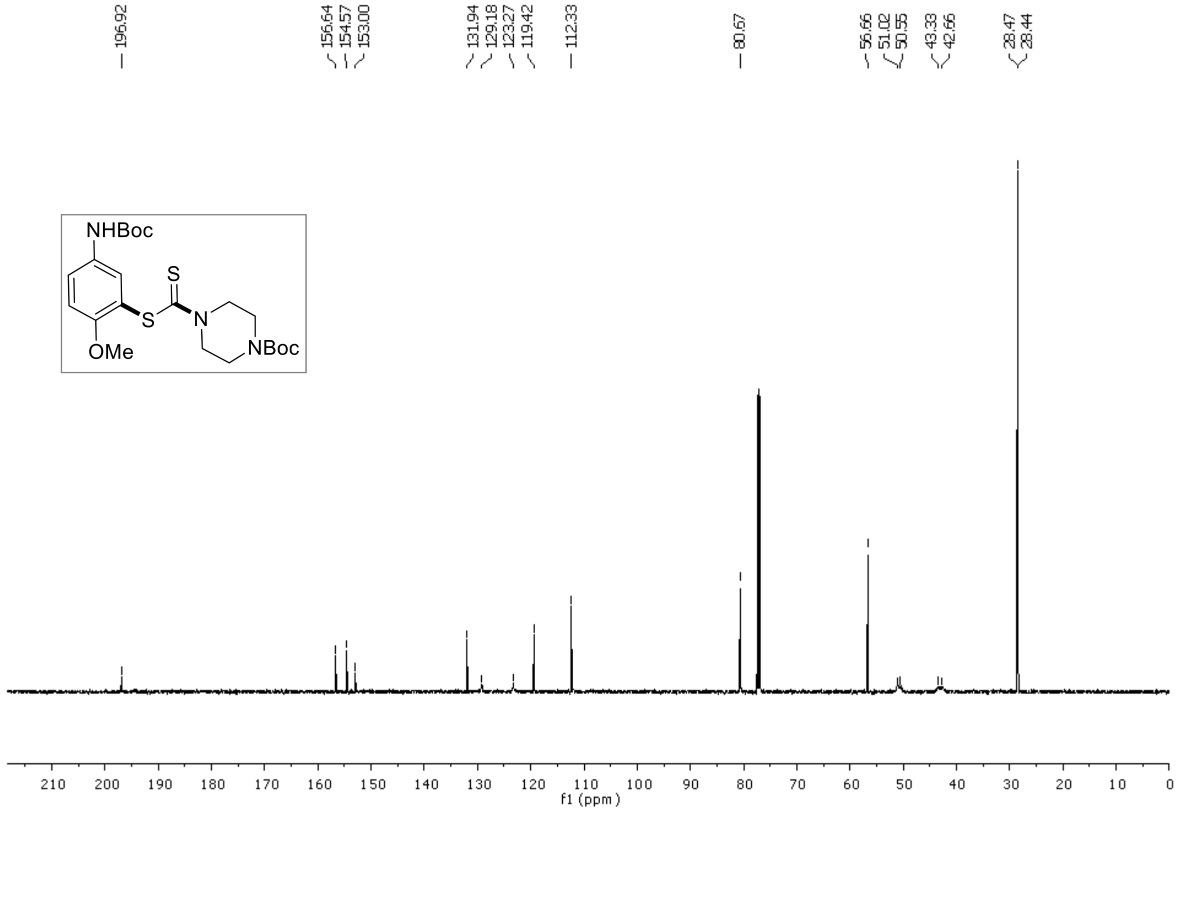

**$^1$H and $^{13}$C NMR of 8**

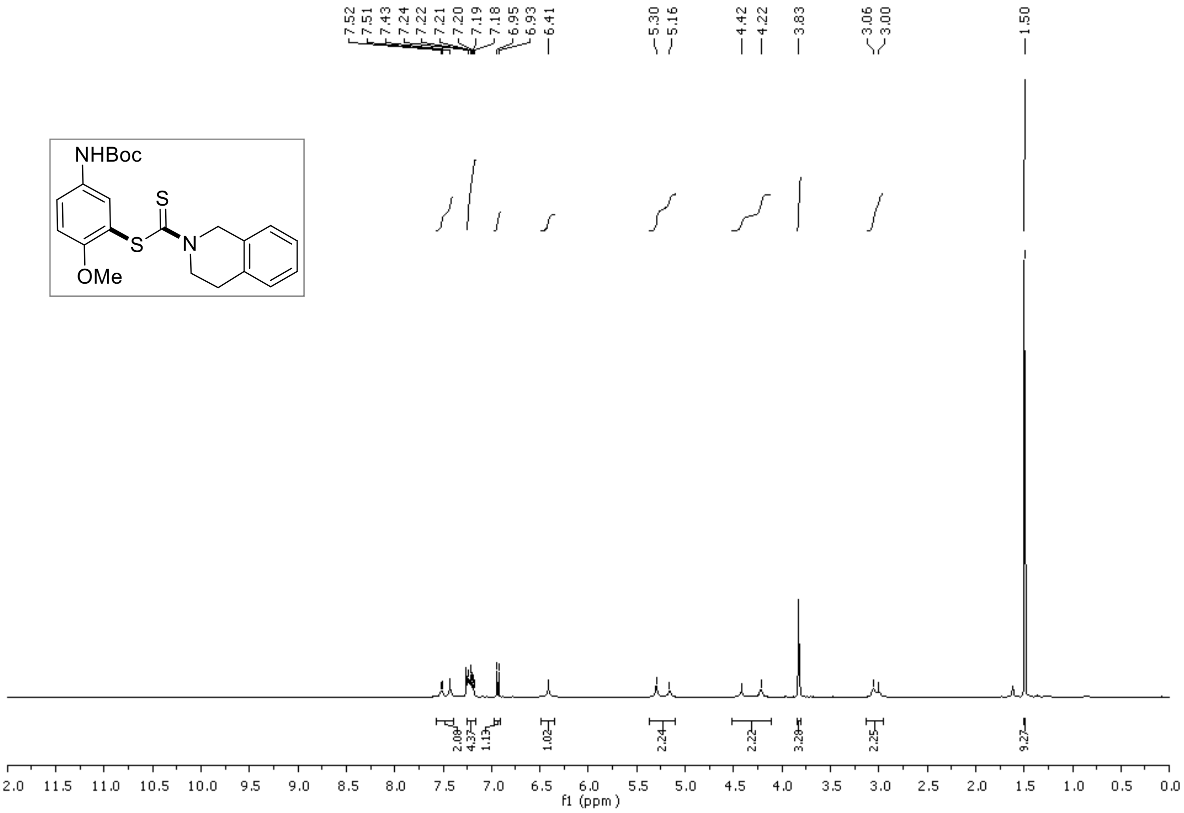


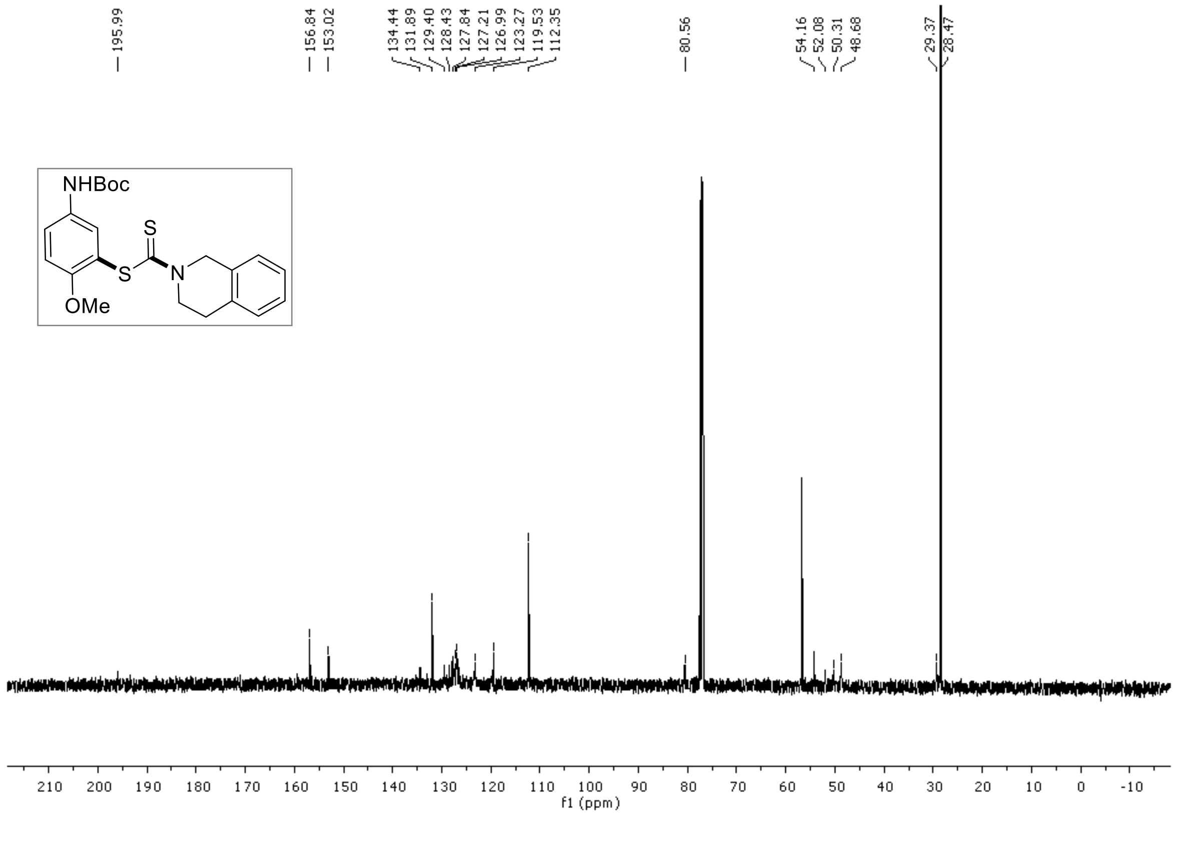

**$^{1}$H and $^{13}$C NMR of 9**

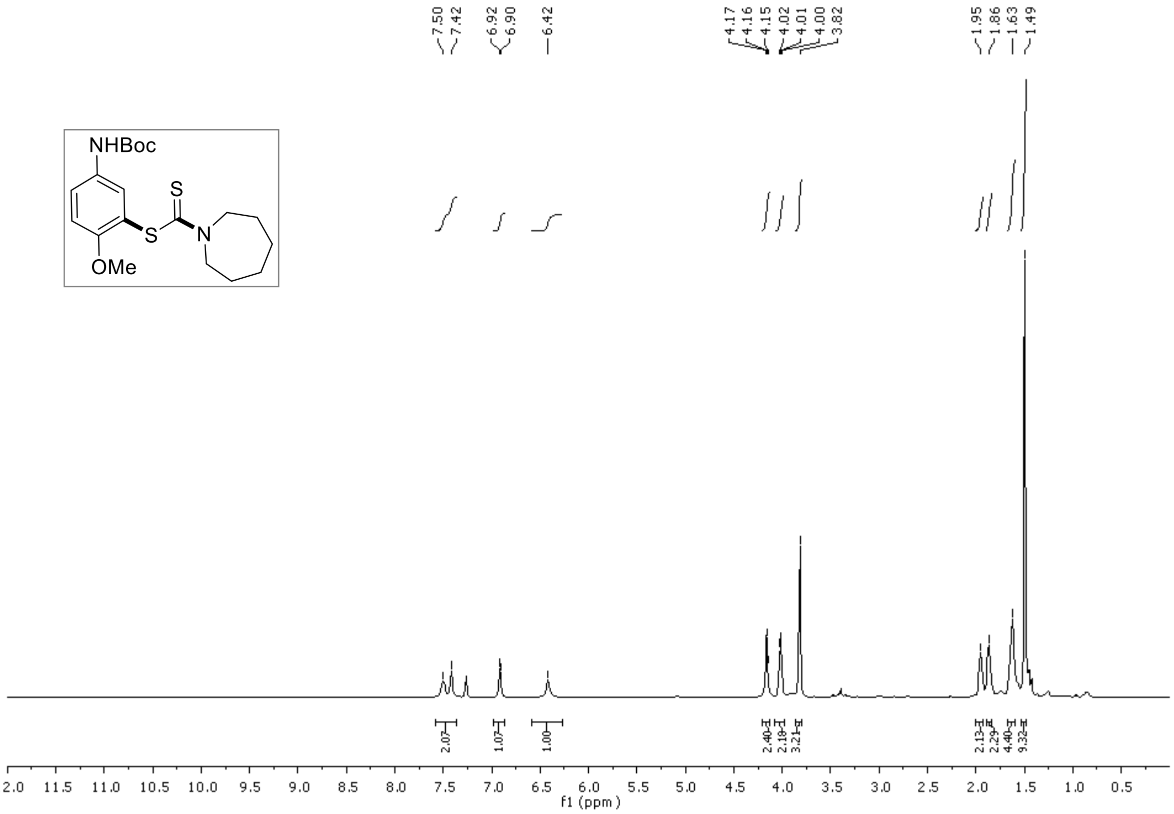


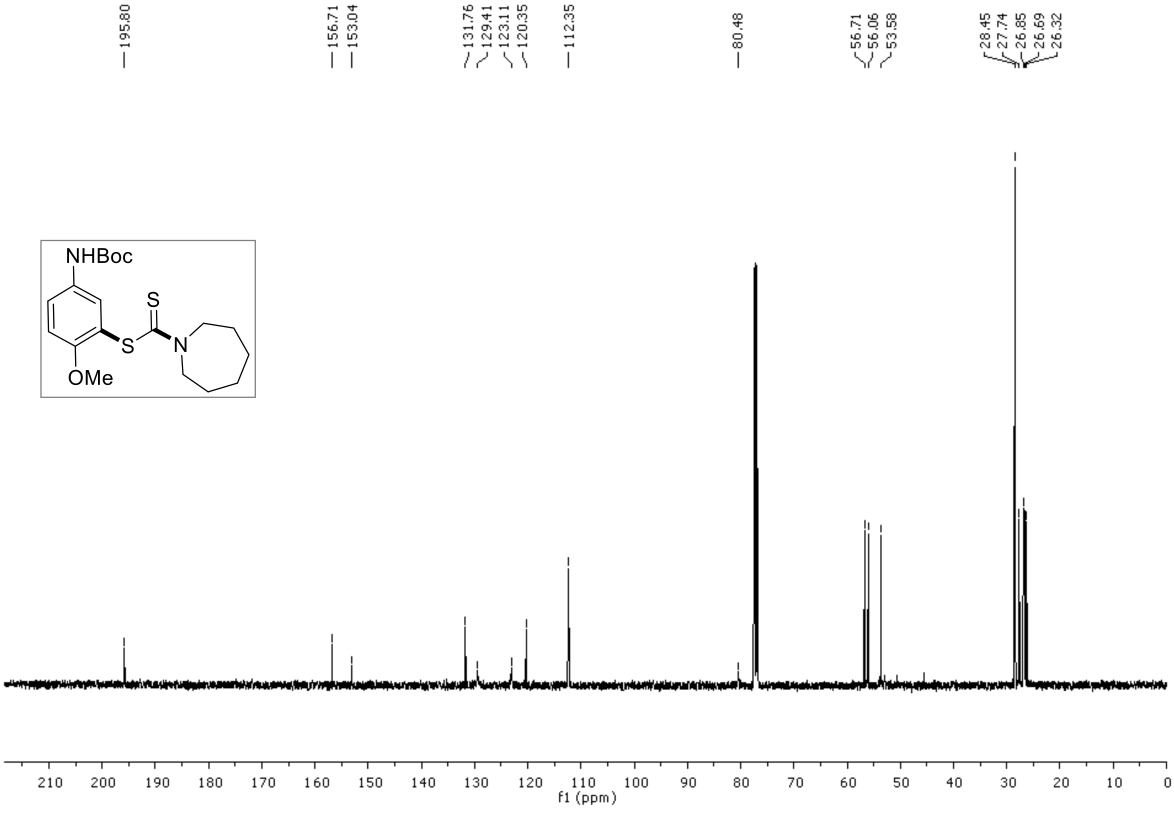

**$^1$H and $^{13}$C NMR of 10**

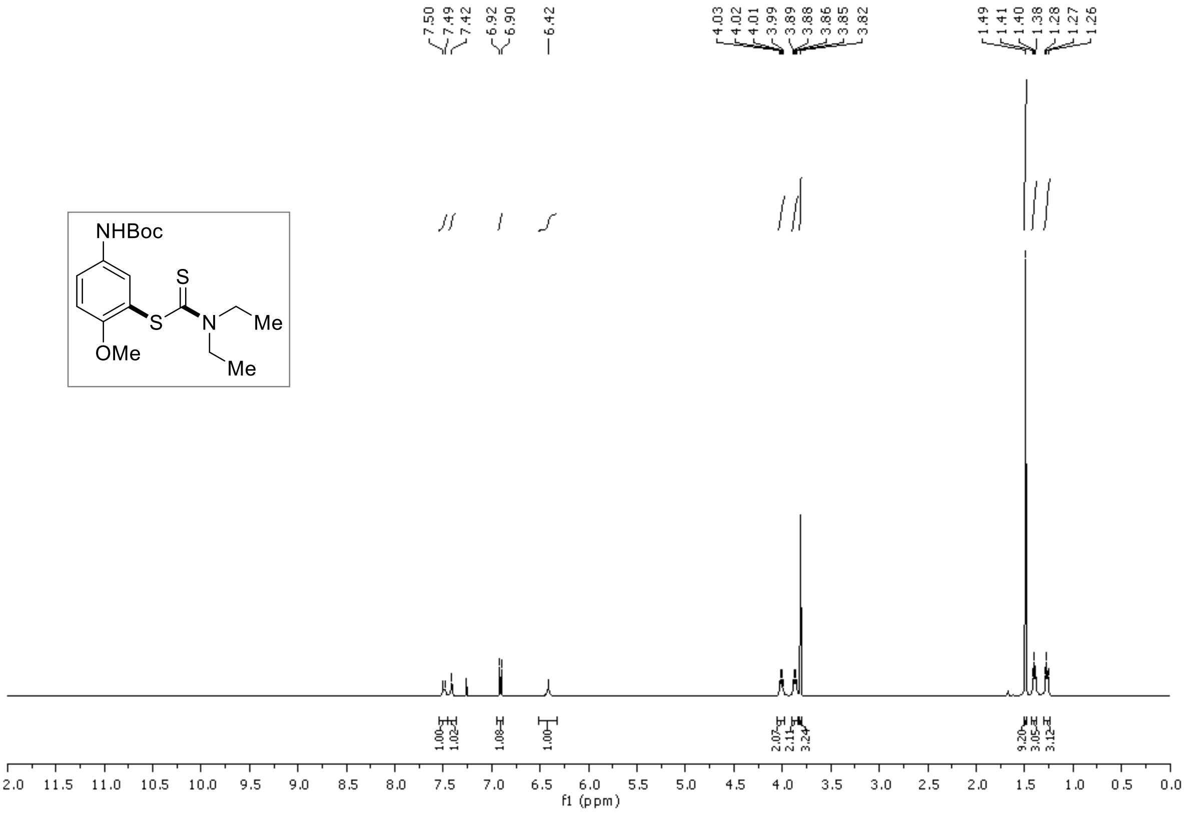


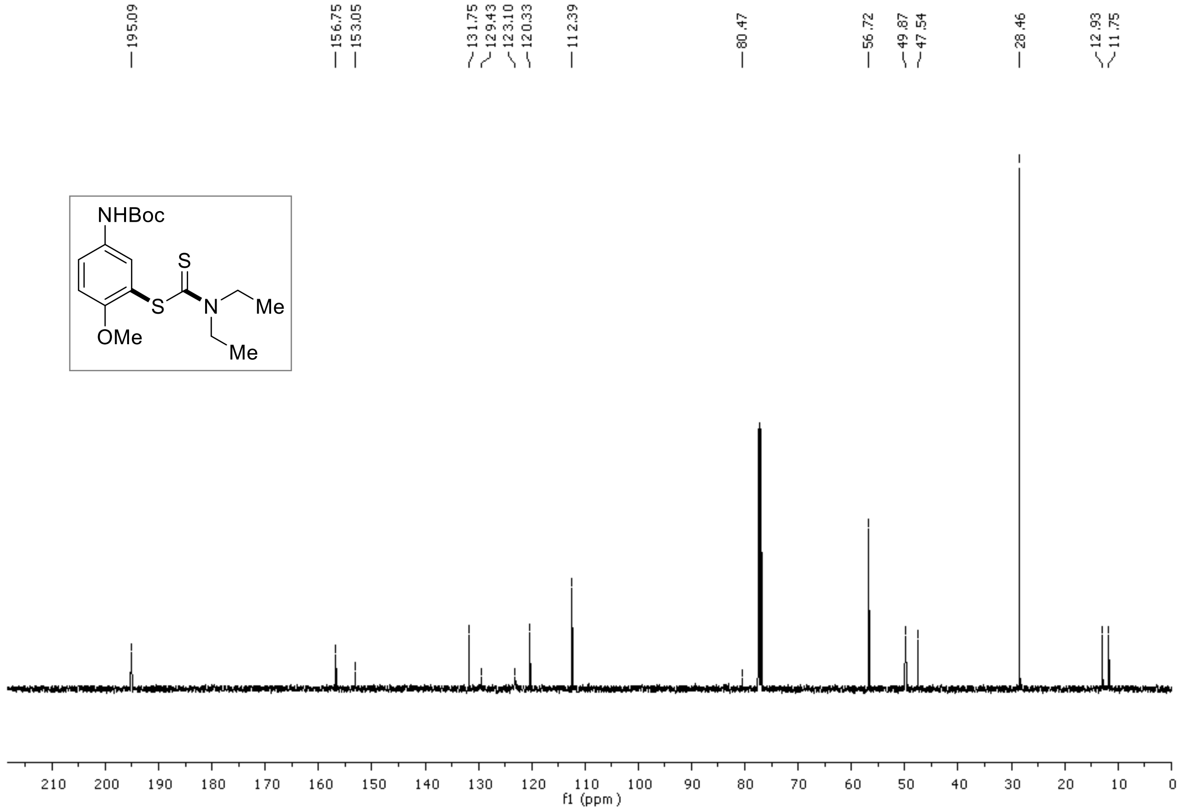

# 1H and 13C NMR of 11

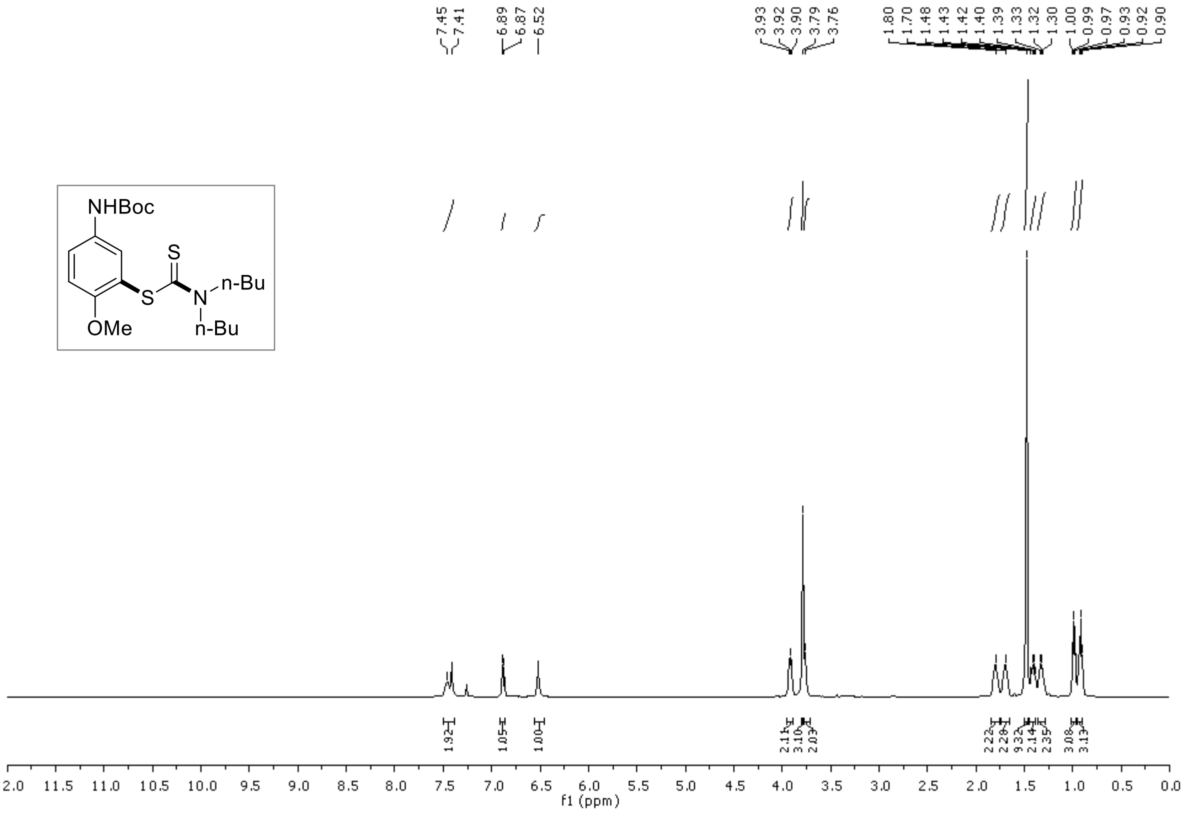


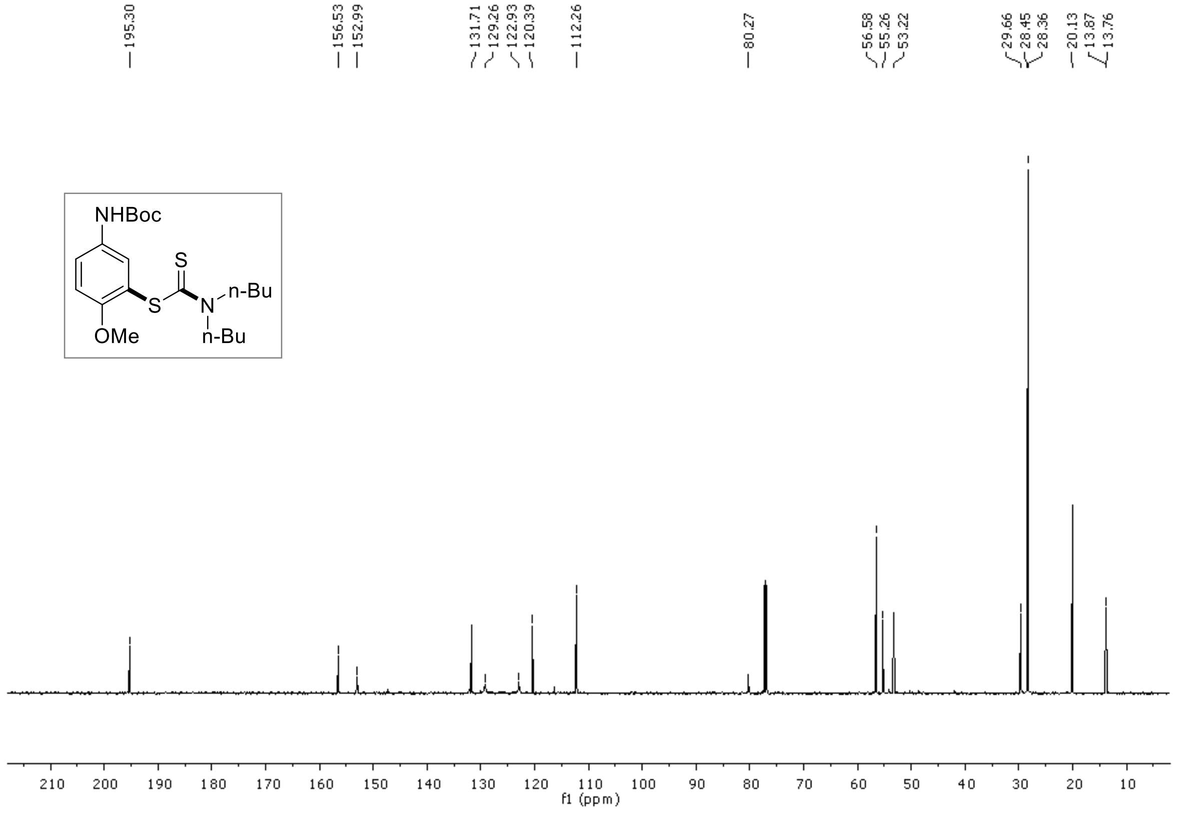

## $^{1}H$ and $^{13}C$ NMR of 12

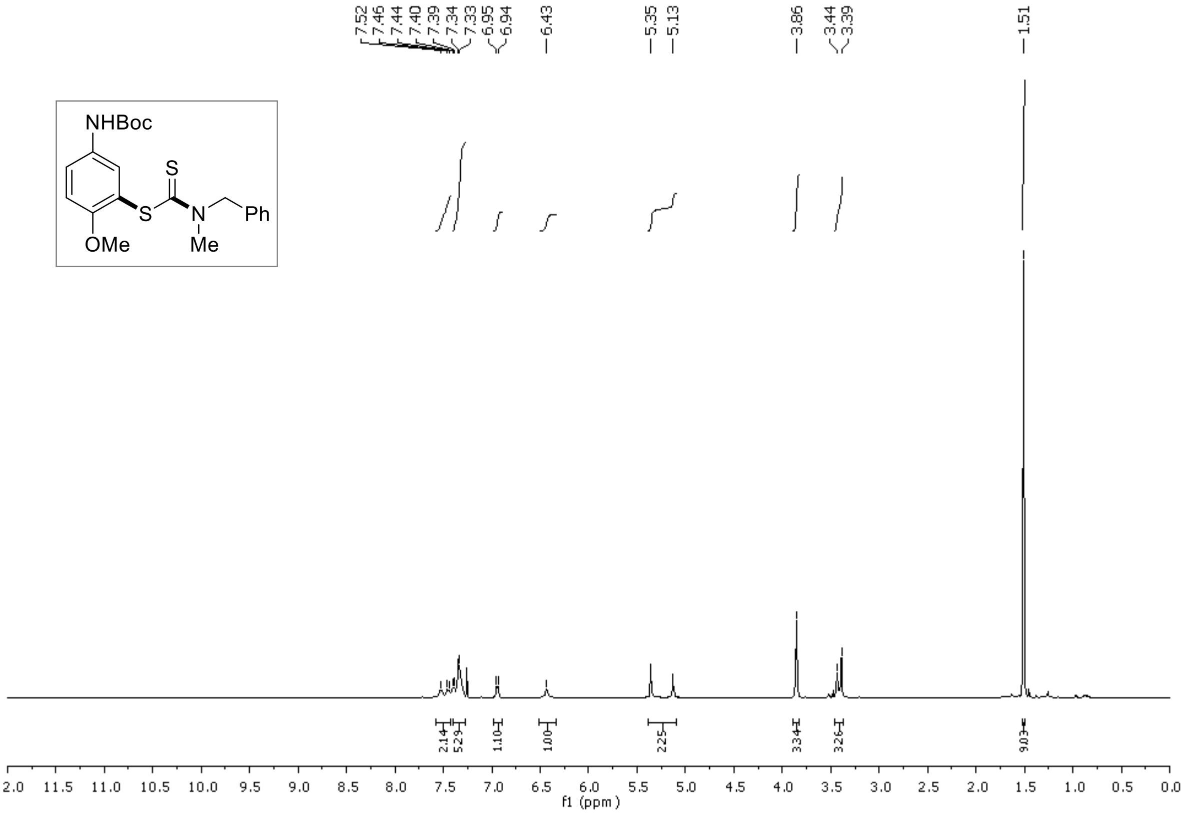


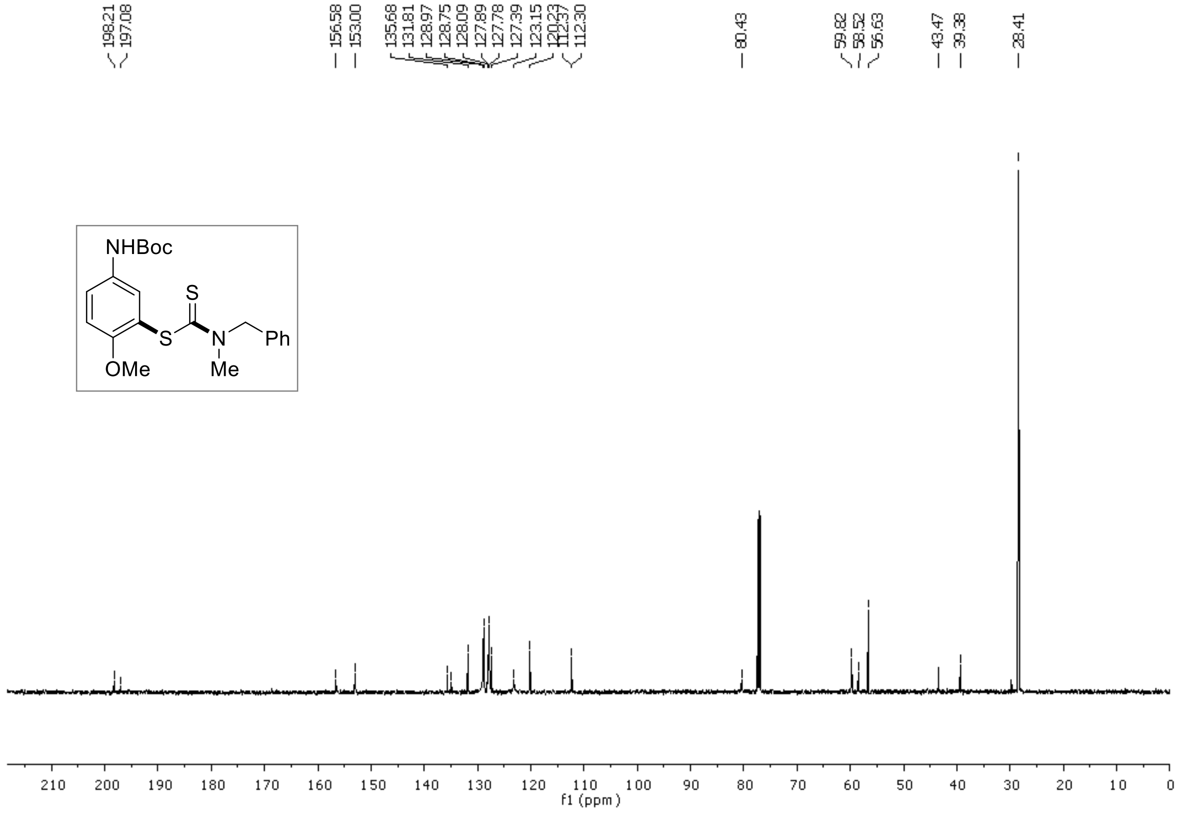

## $^{1}H$ and $^{13}C$ NMR of 13

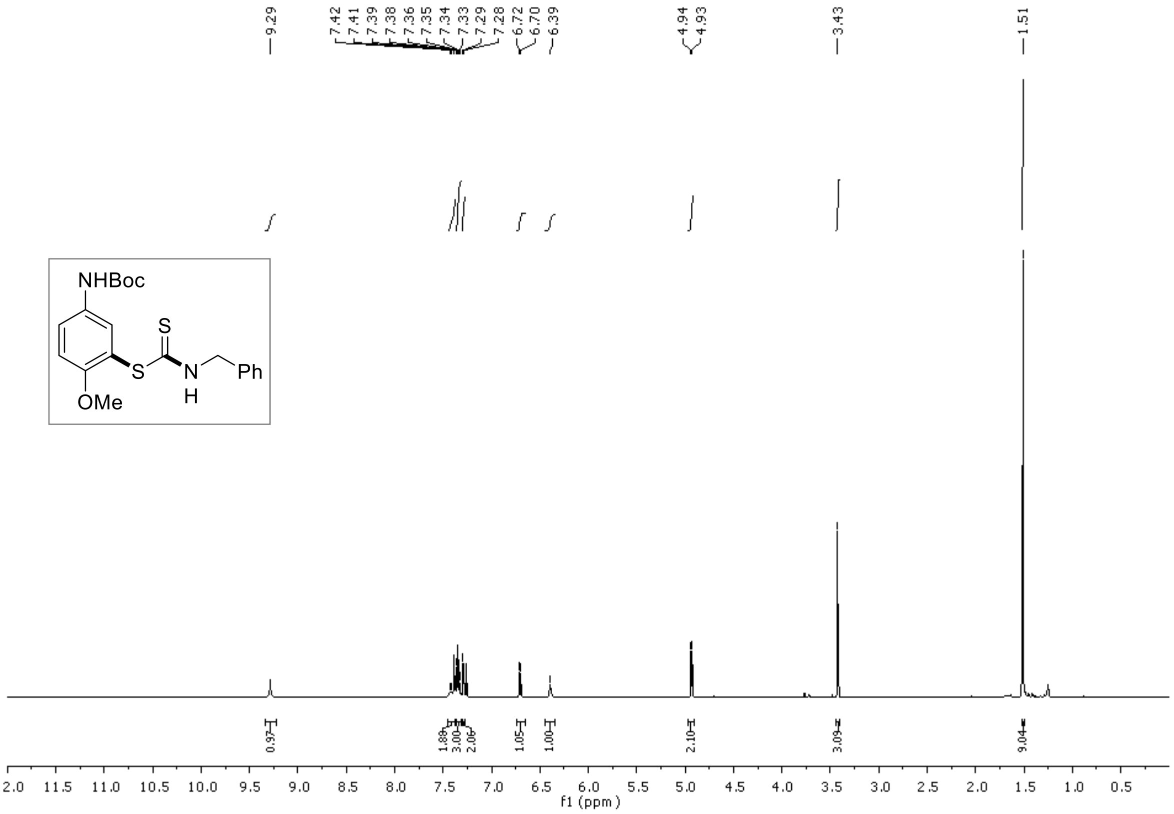


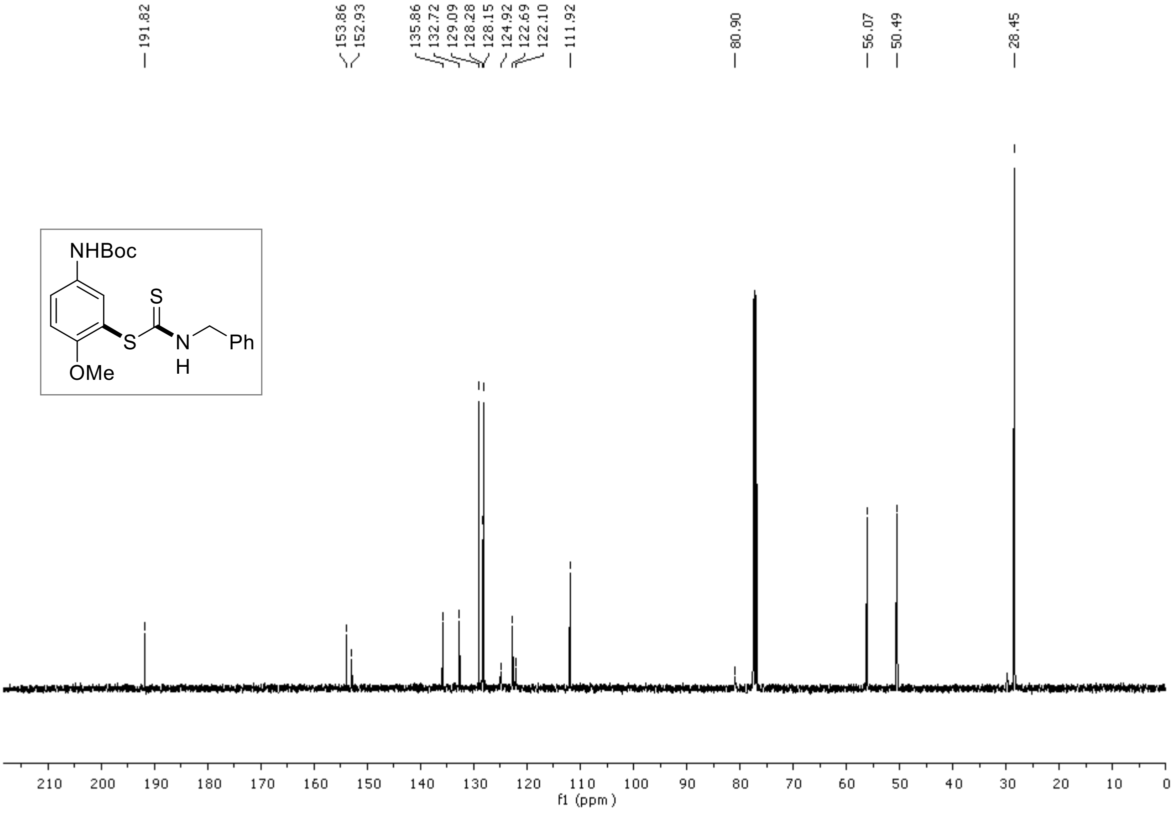

## $^{1}$H and $^{13}$C NMR of 14

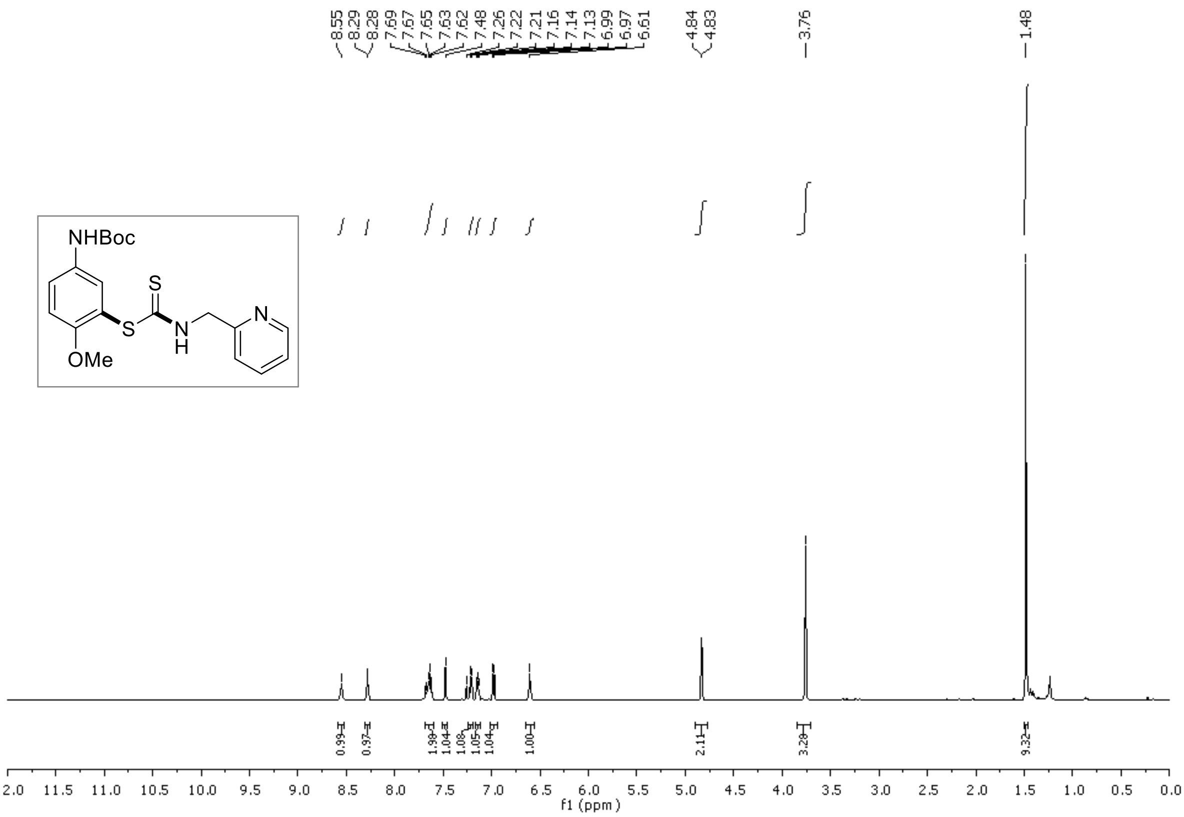


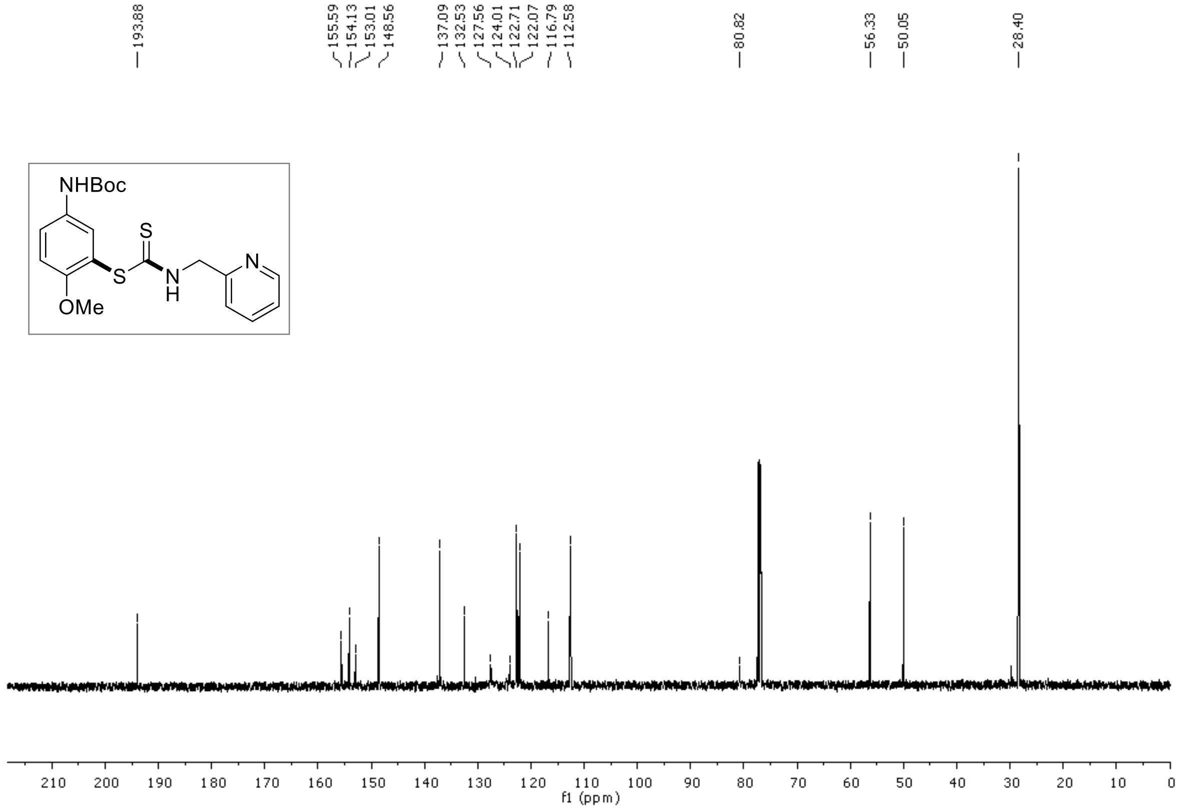

# $^{1}$H and $^{13}$C NMR of 15

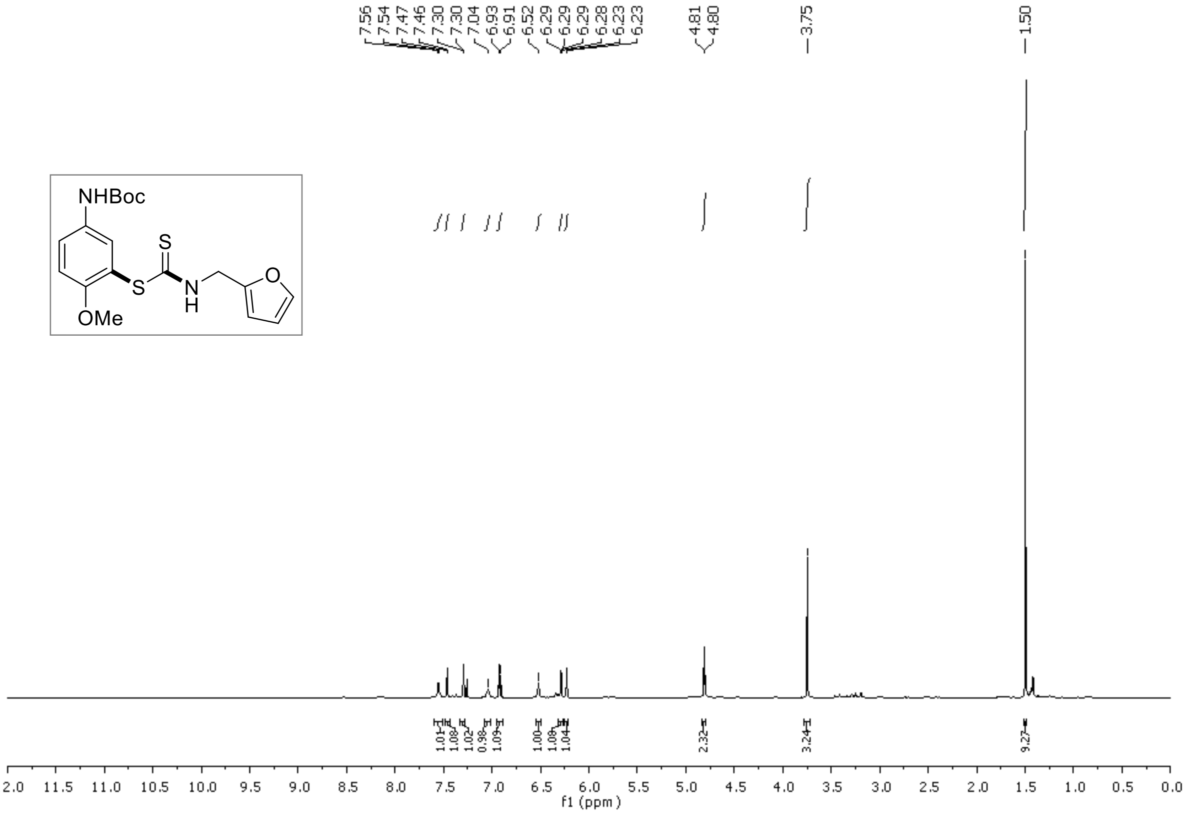


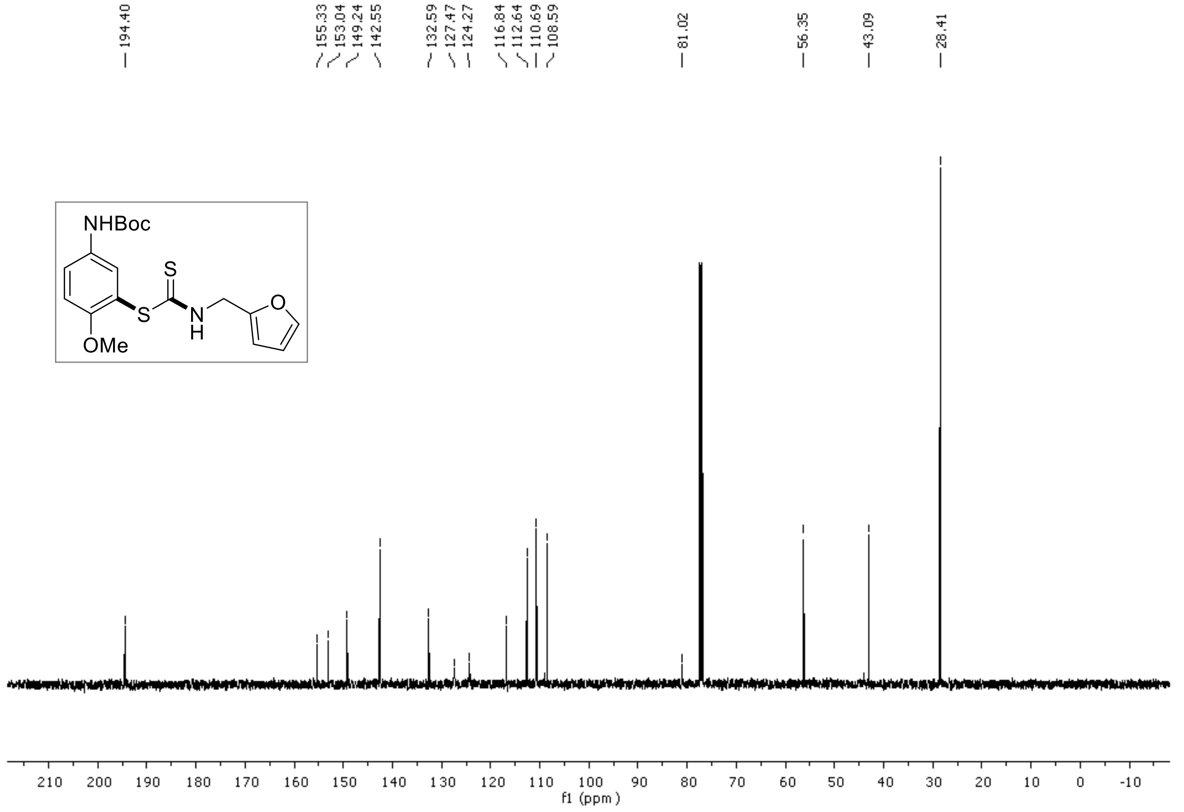

# $^{1}$H and $^{13}$C NMR of 16

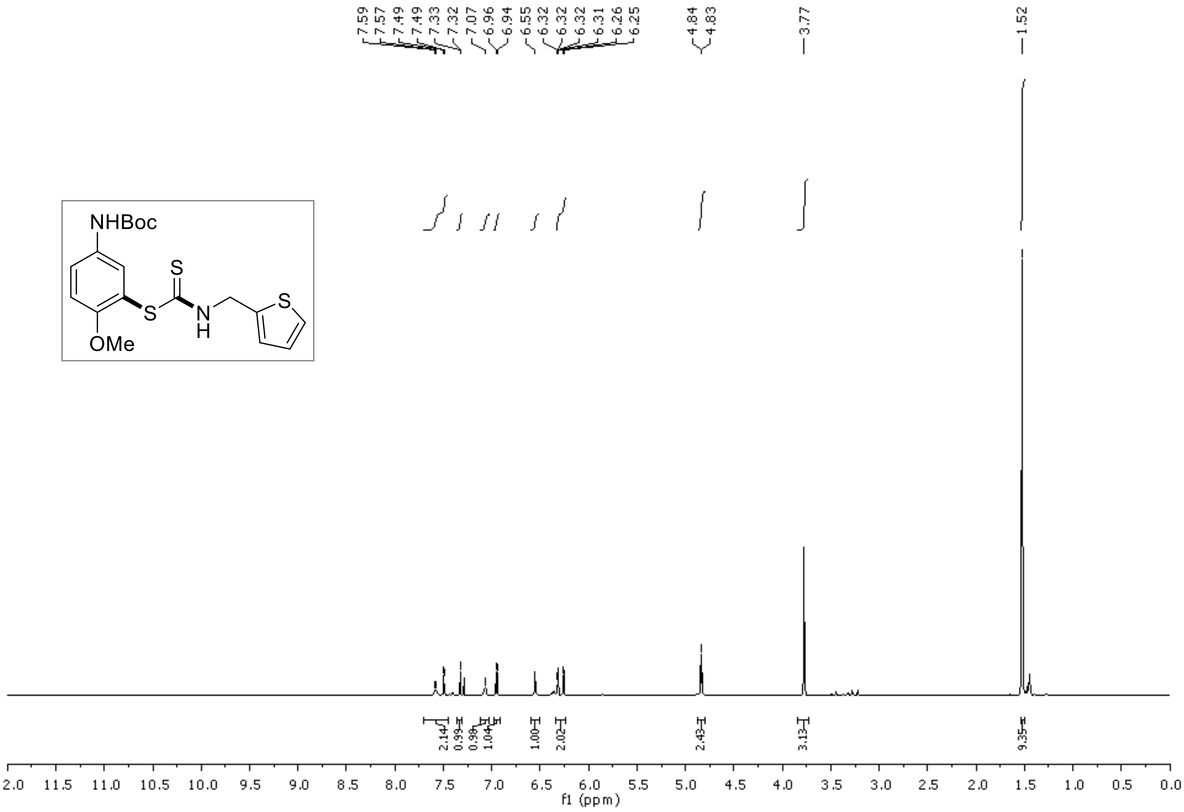


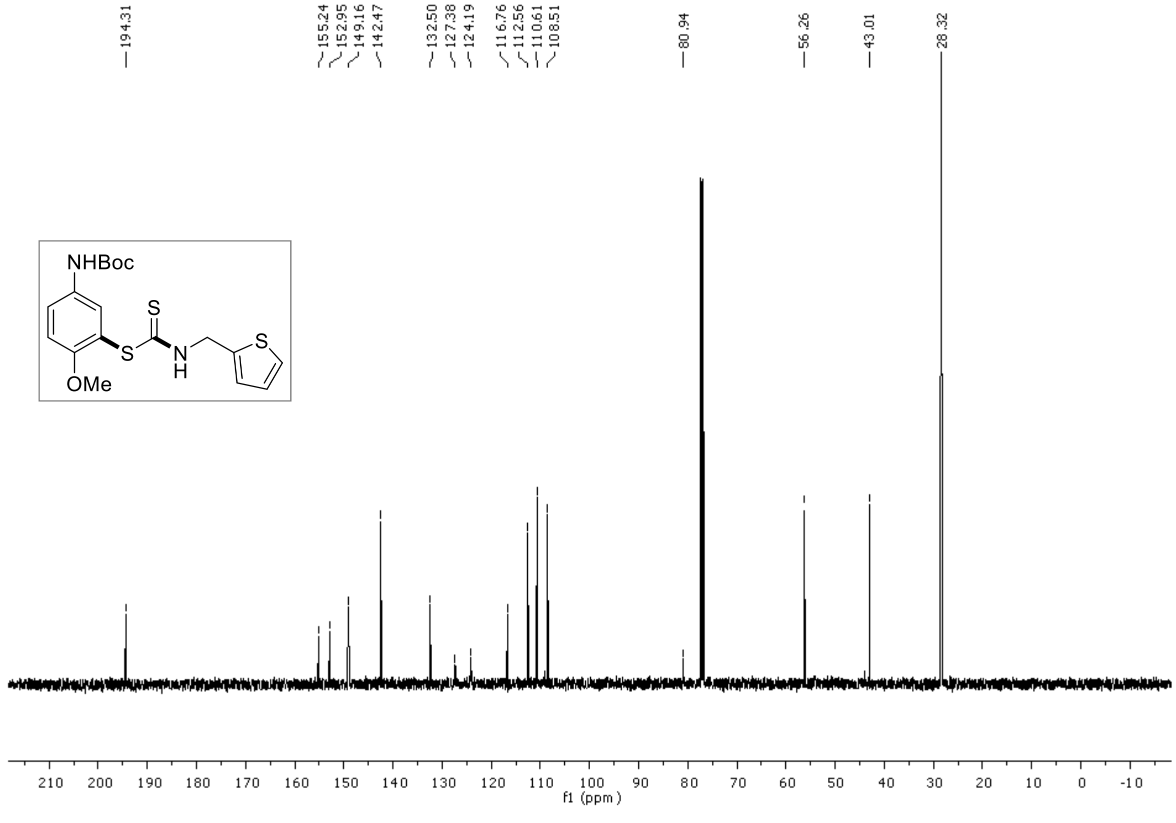

# $^{1}H$ and $^{13}C$ NMR of 17

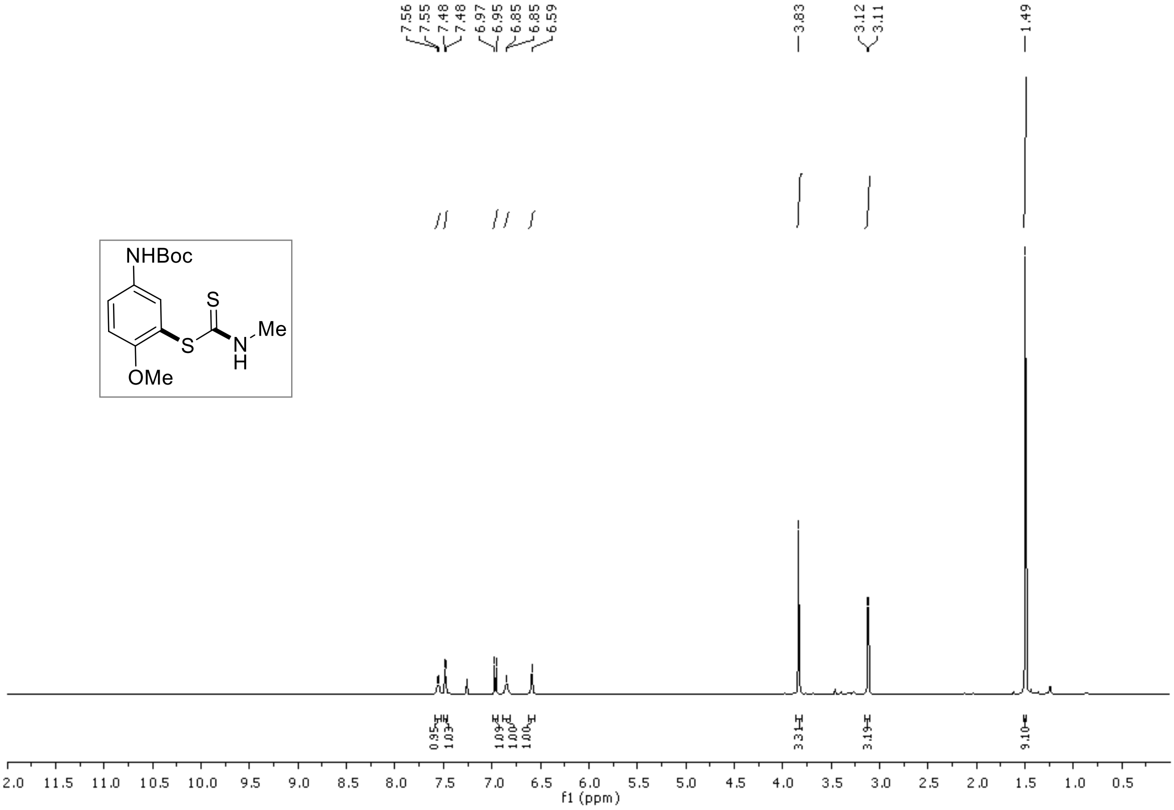


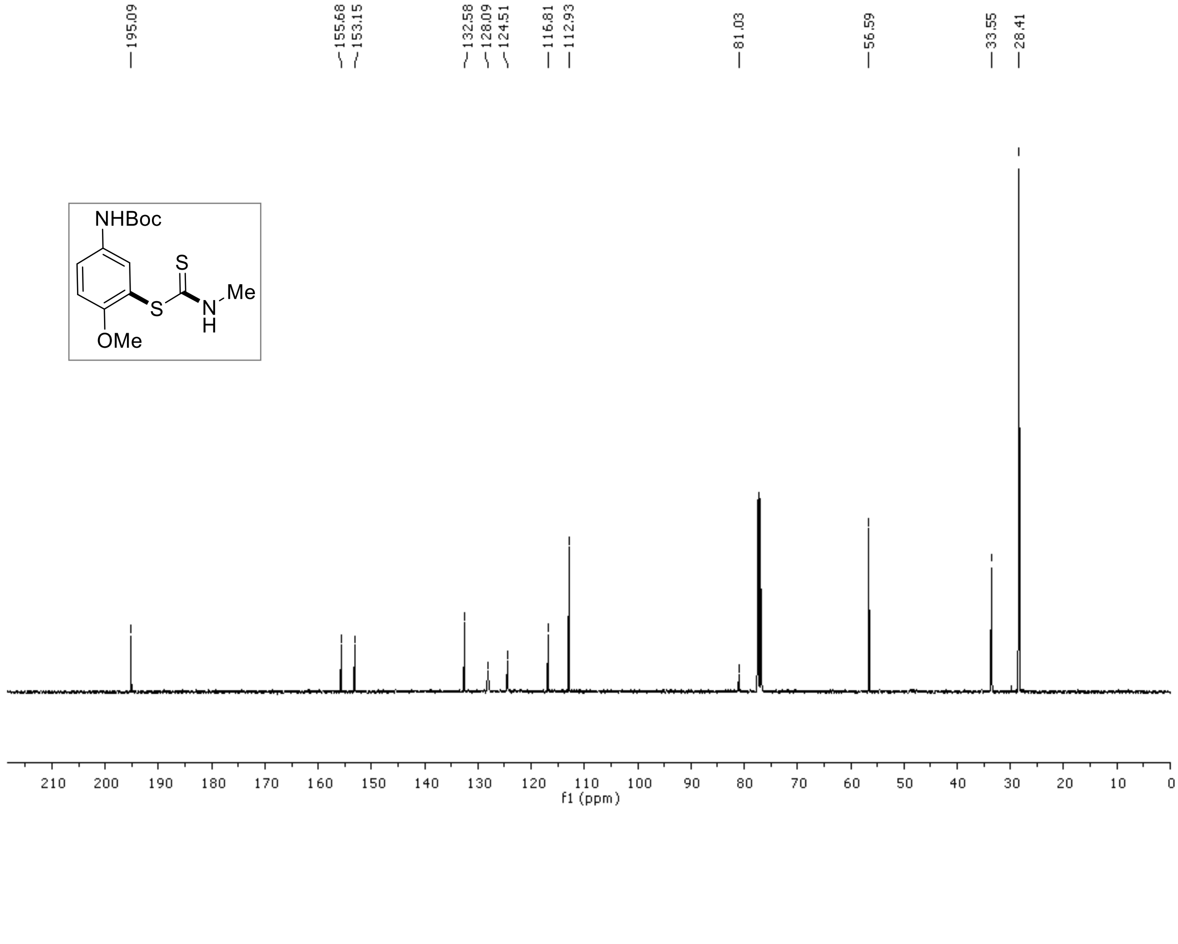

## $^{1}$H and $^{13}$C NMR of 18

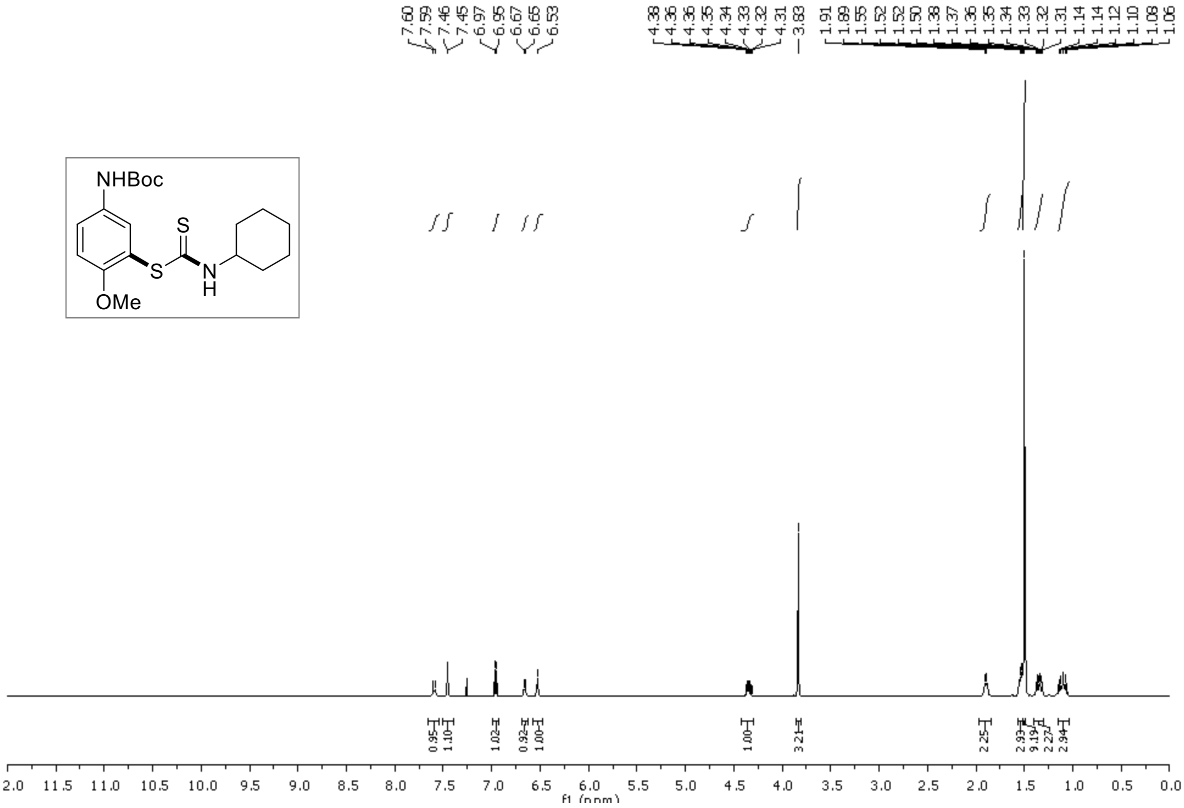


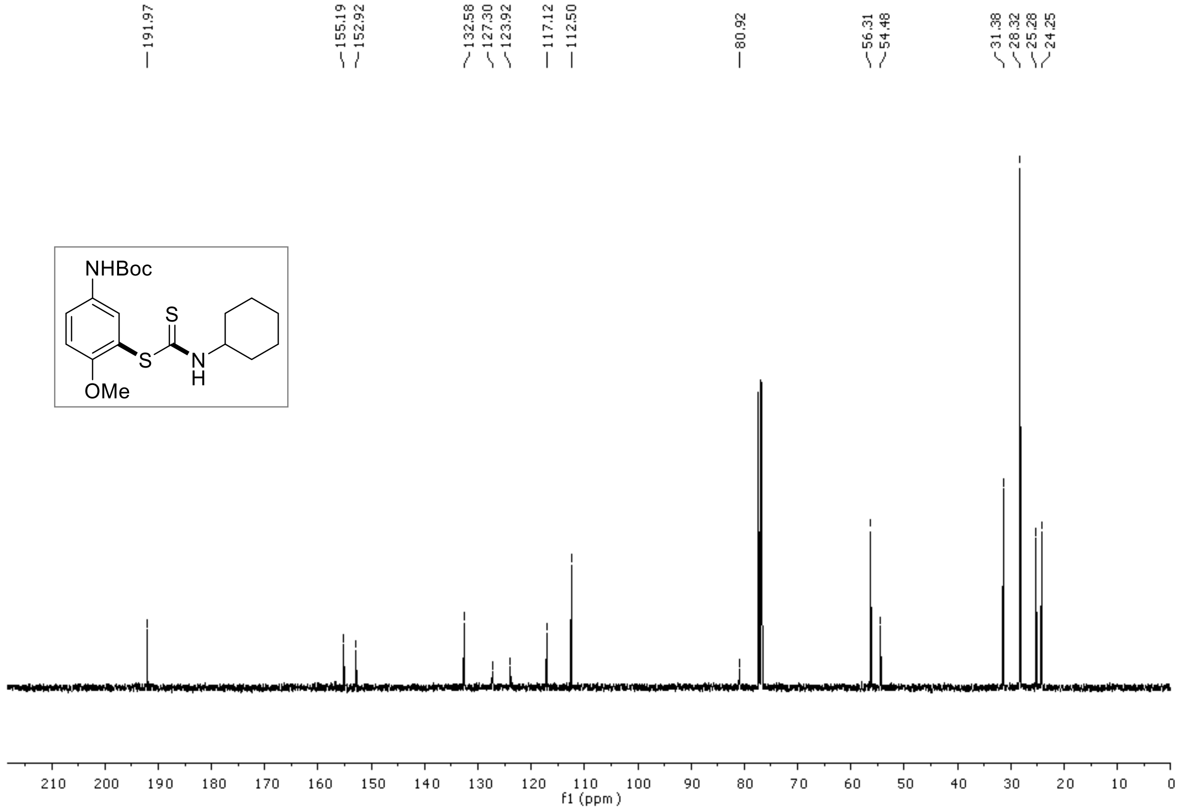

## $^{1}H$ and $^{13}C$ NMR of 19

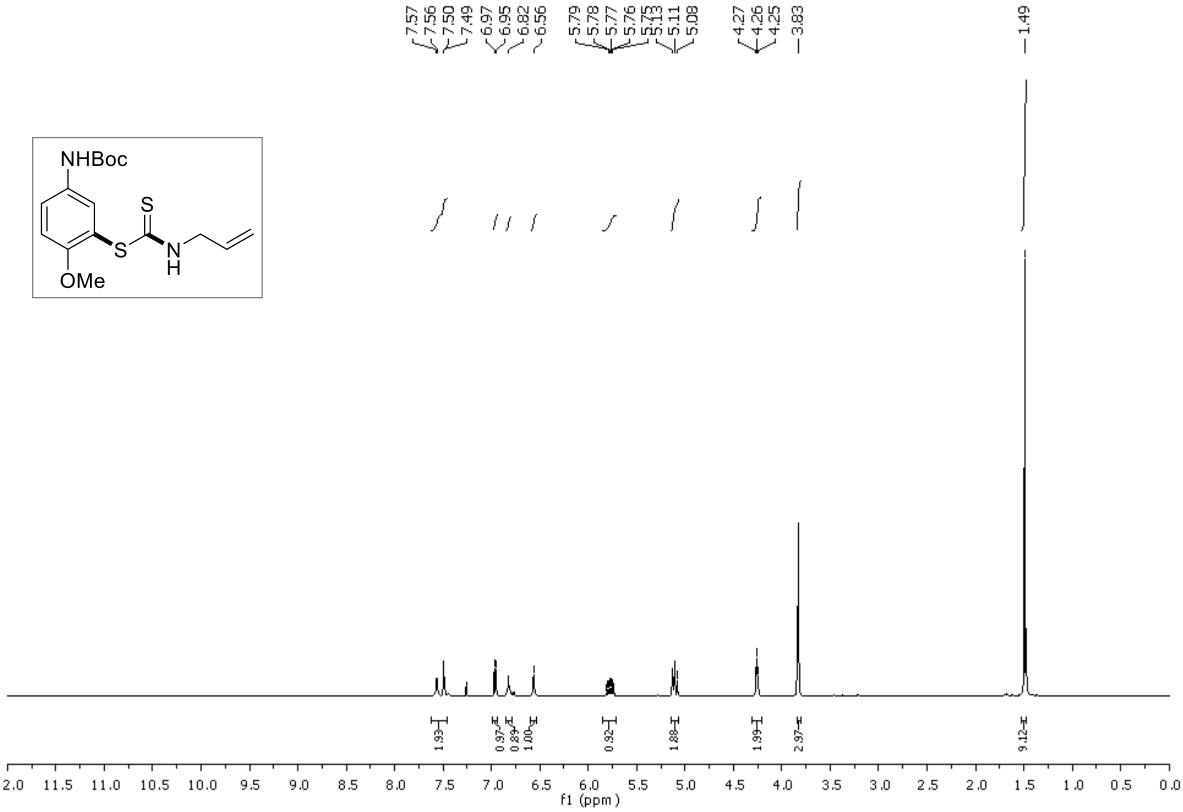


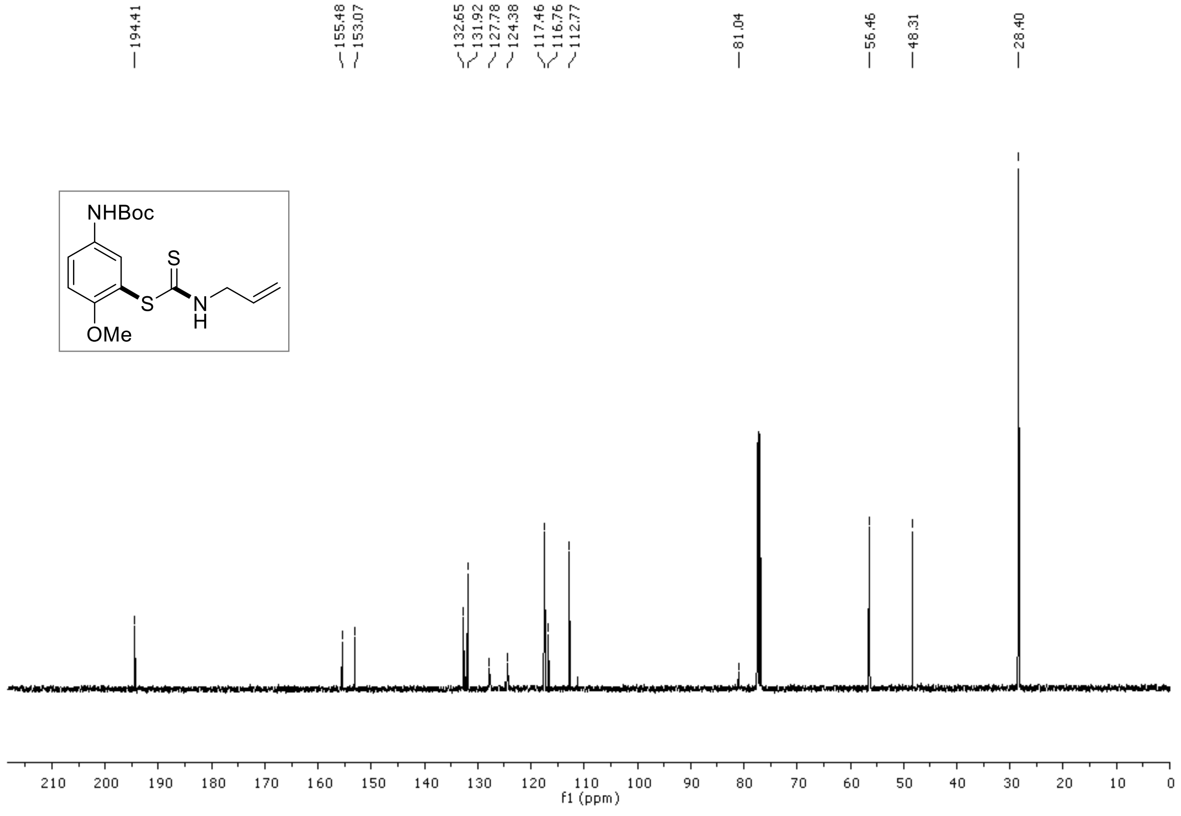

## $^{1}$H and $^{13}$C NMR of 20

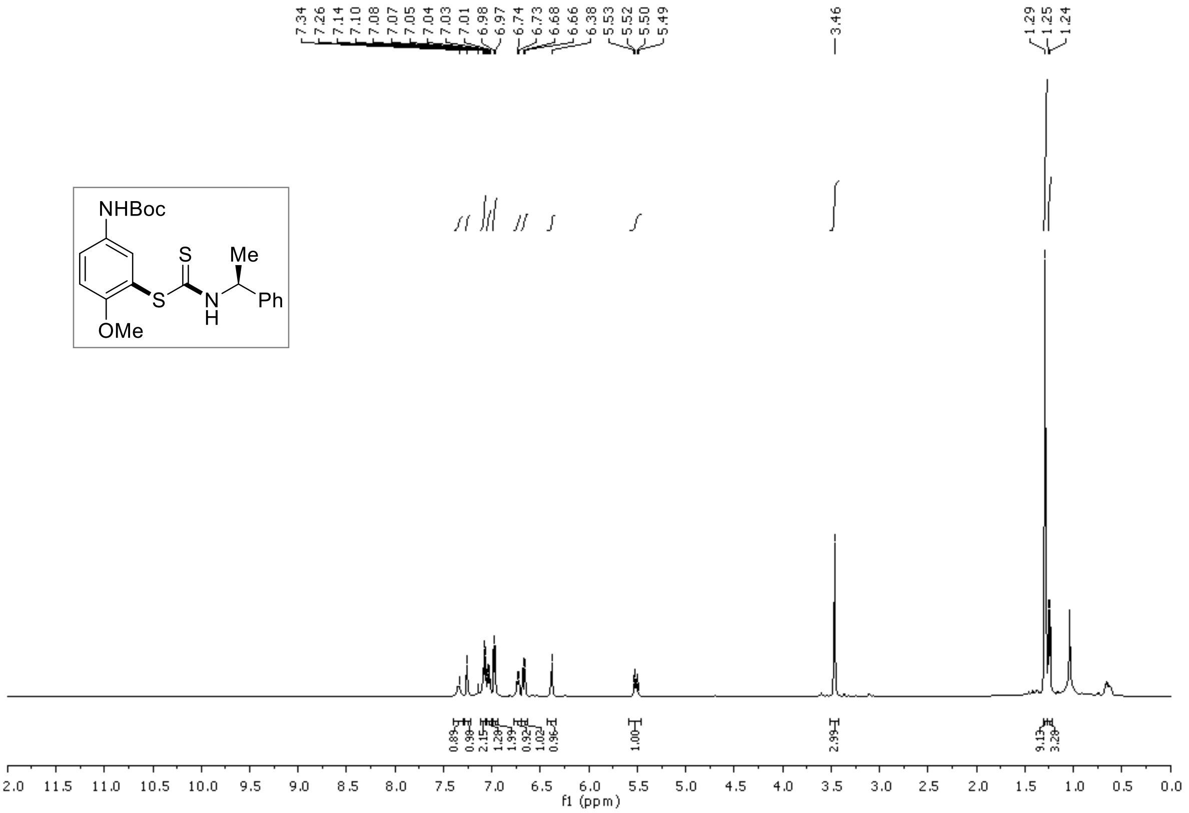


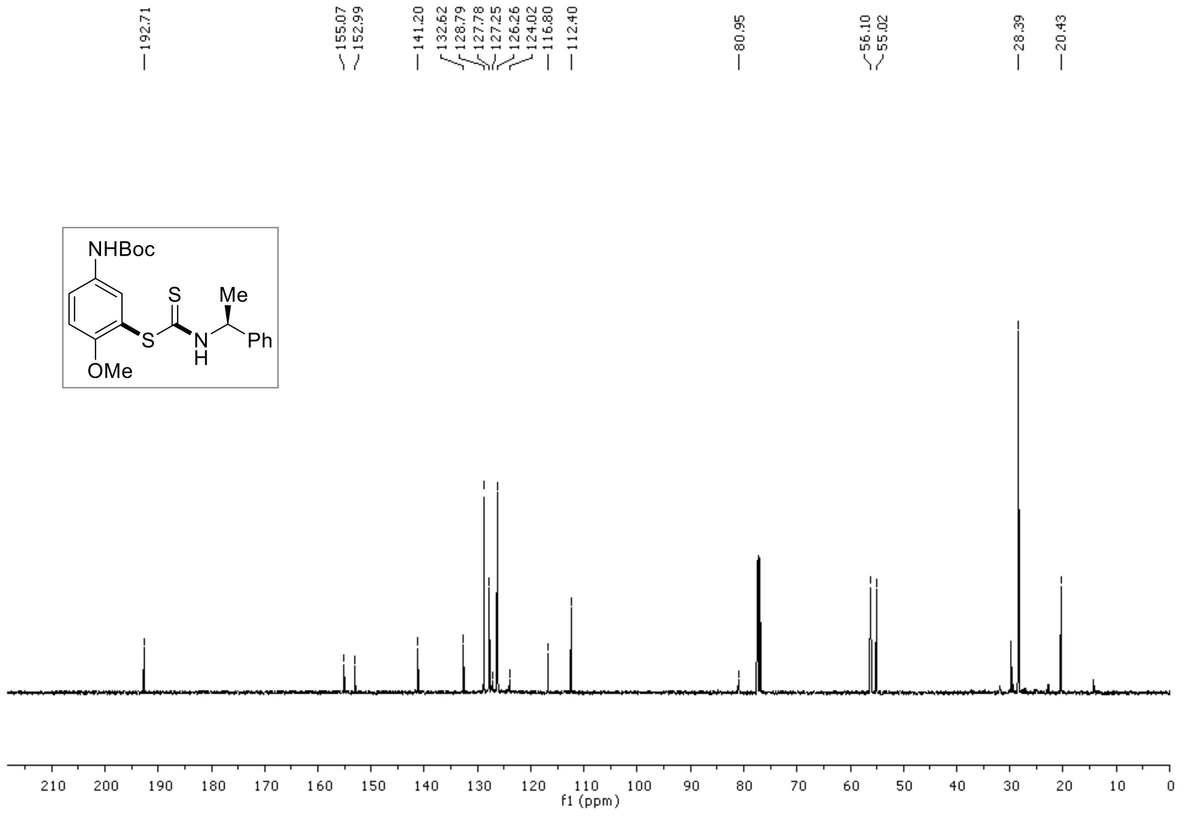

**$^{1}$H and $^{13}$C NMR of 21**

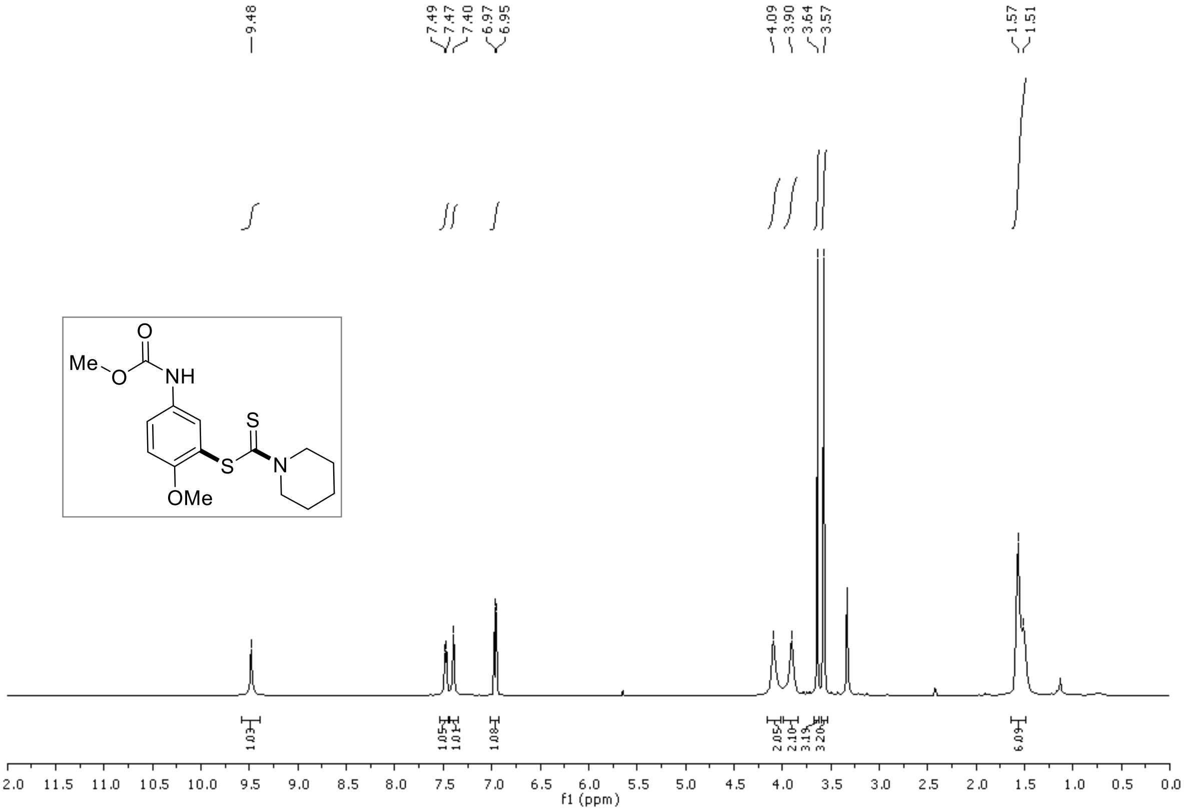


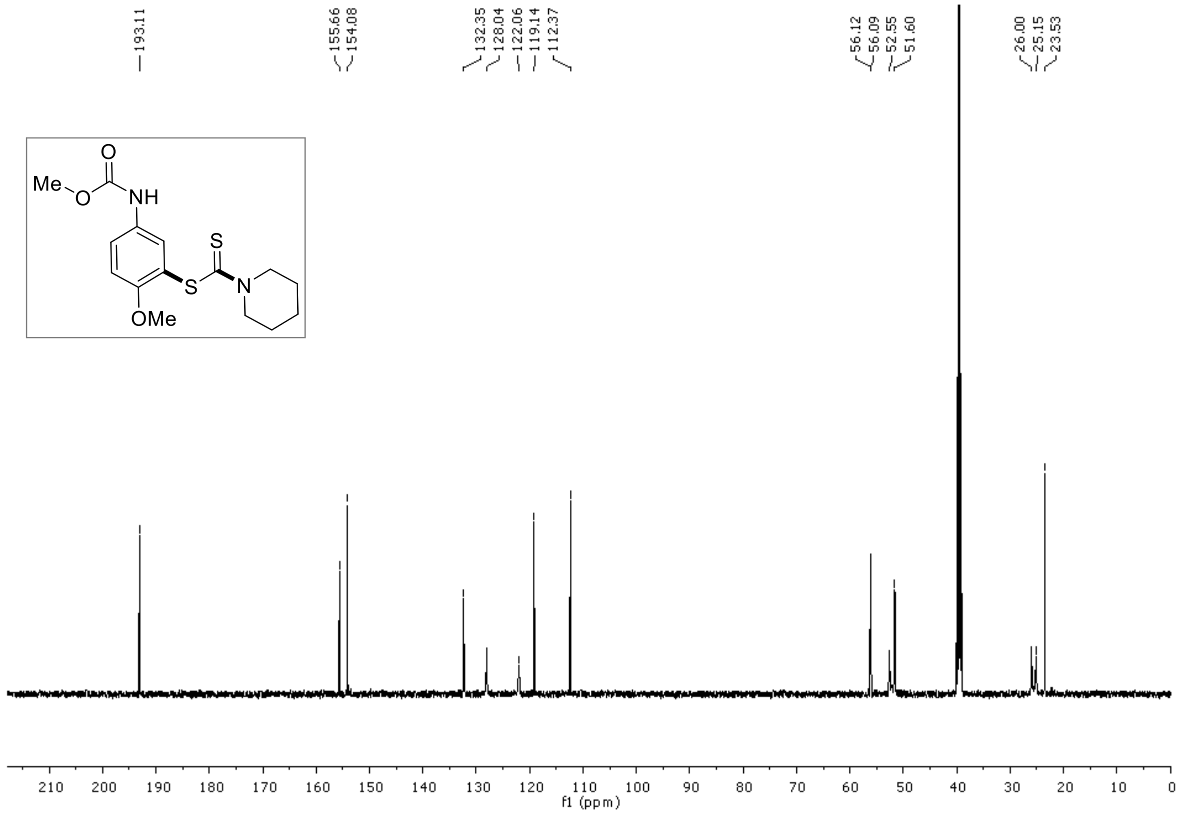

## $^{1}H$ and $^{13}C$ NMR of 22

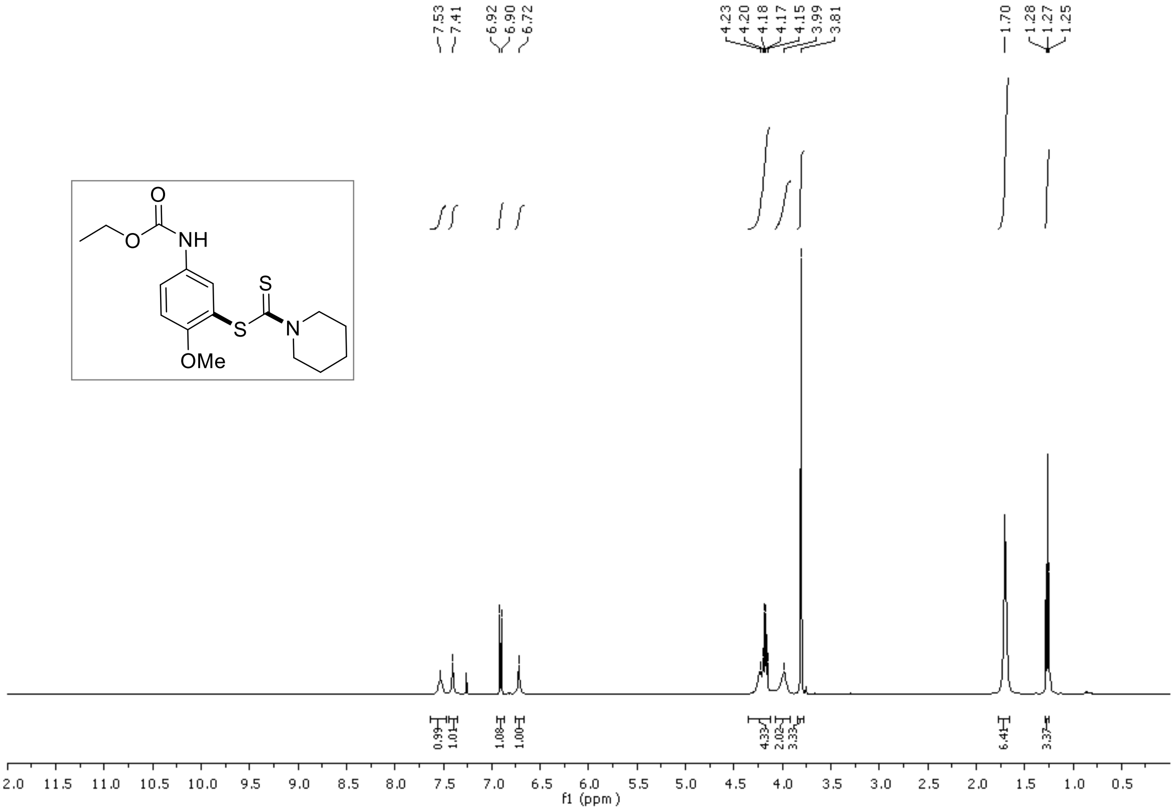


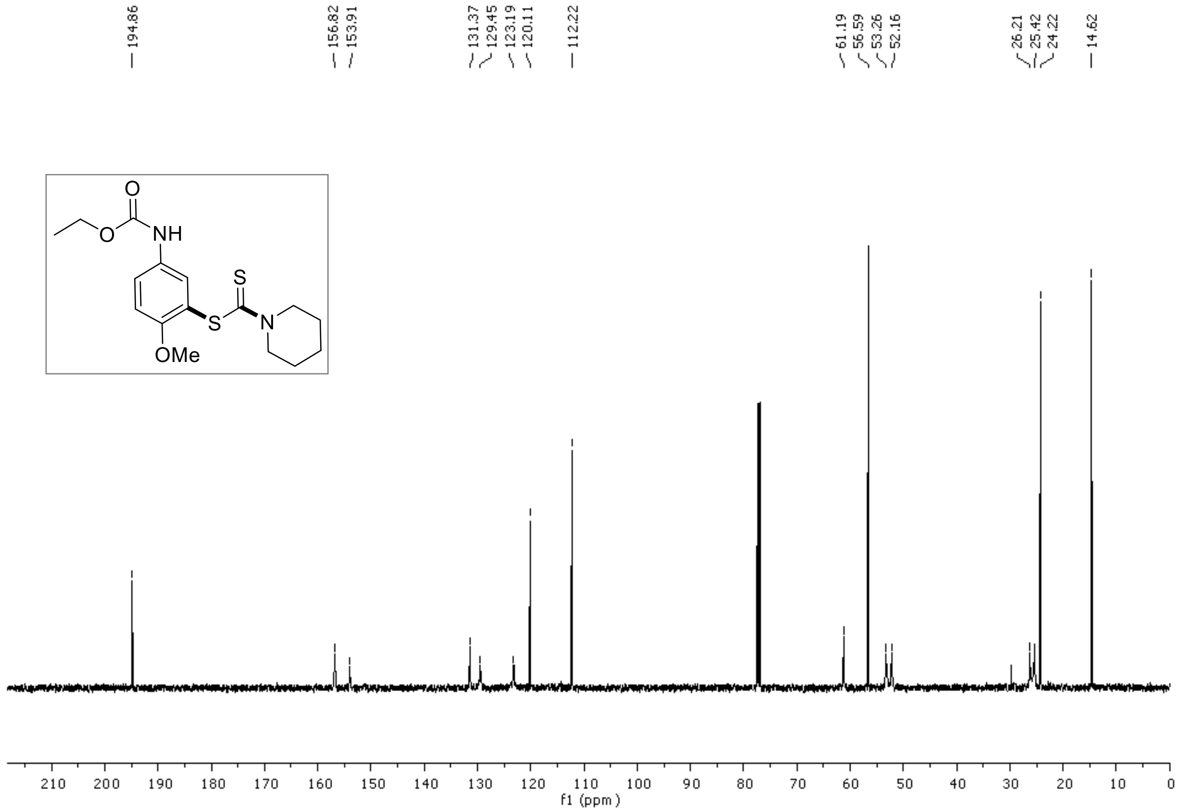

## $^{1}H$ and $^{13}C$ NMR of 23

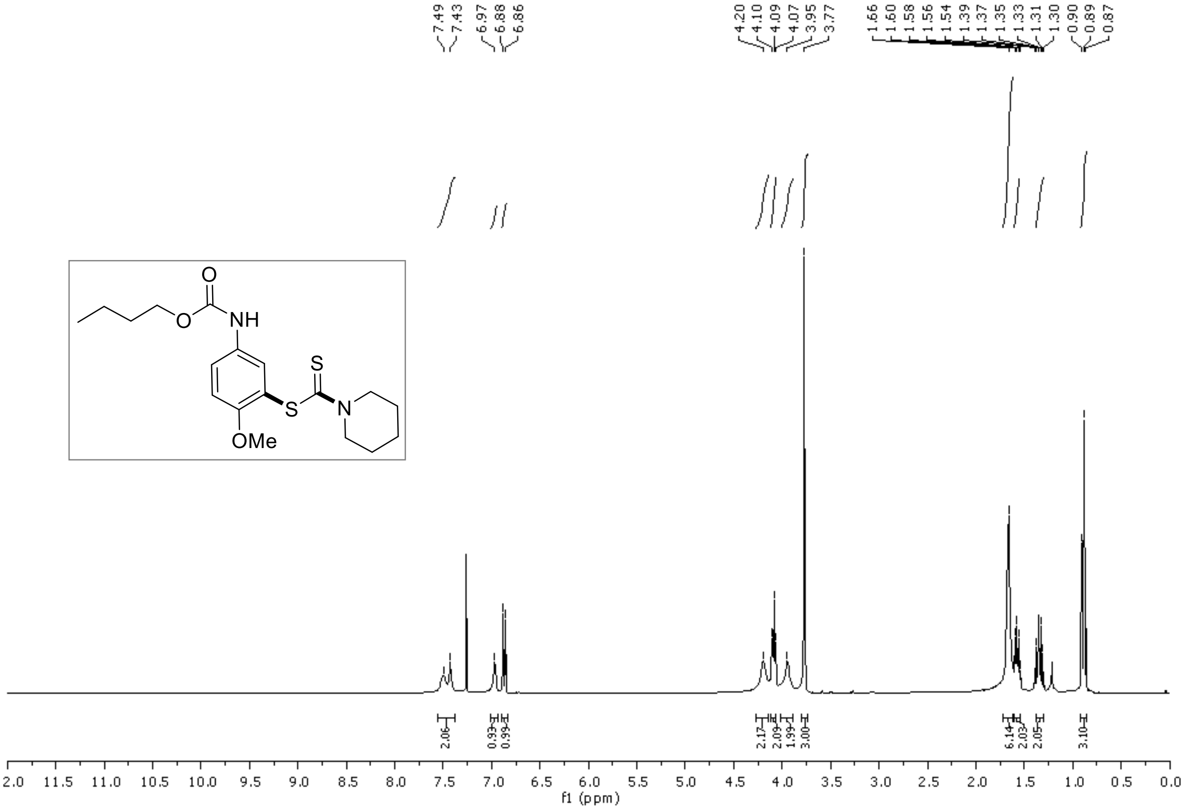


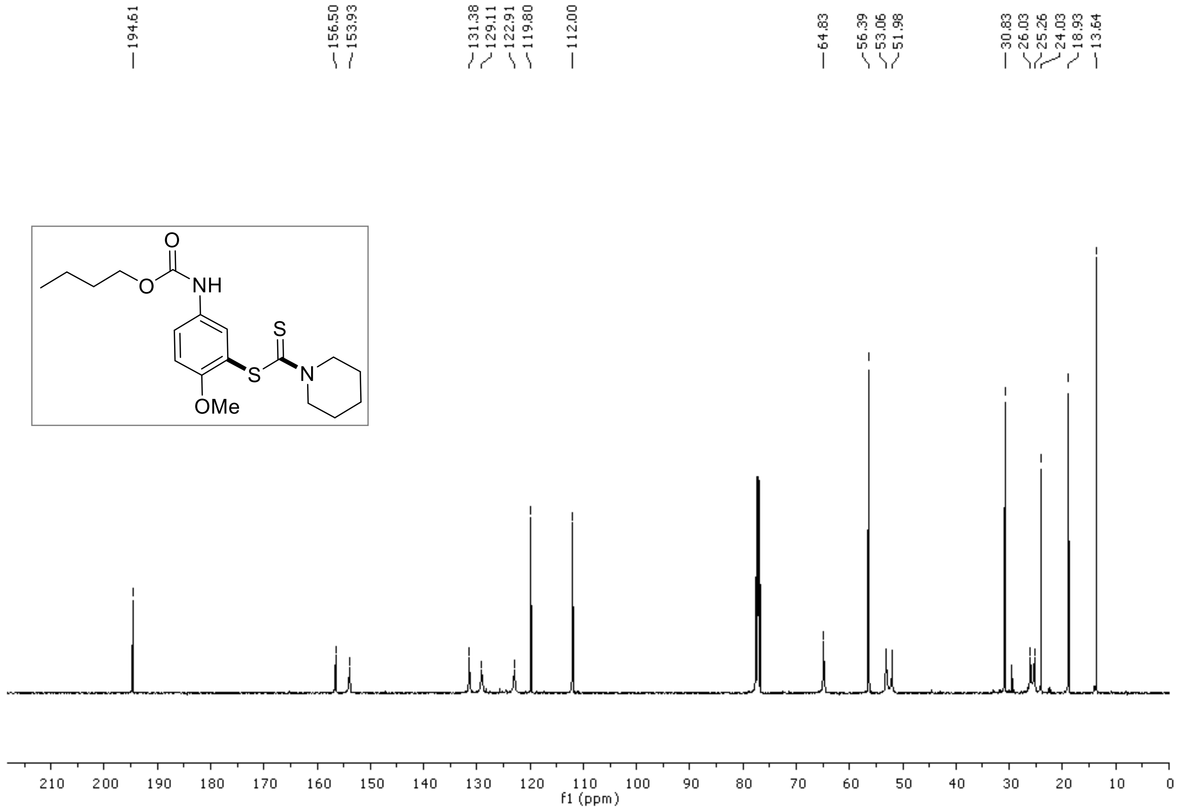

## $^{1}$H and $^{13}$C NMR of 24

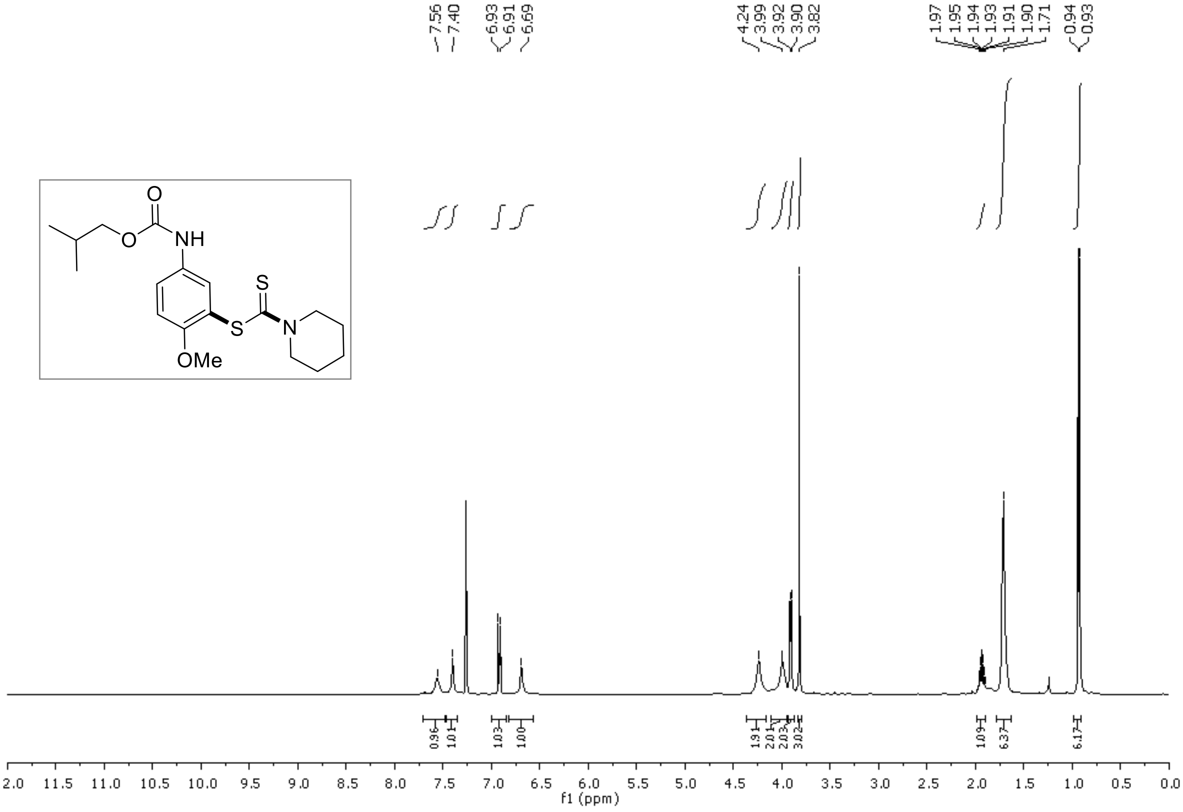


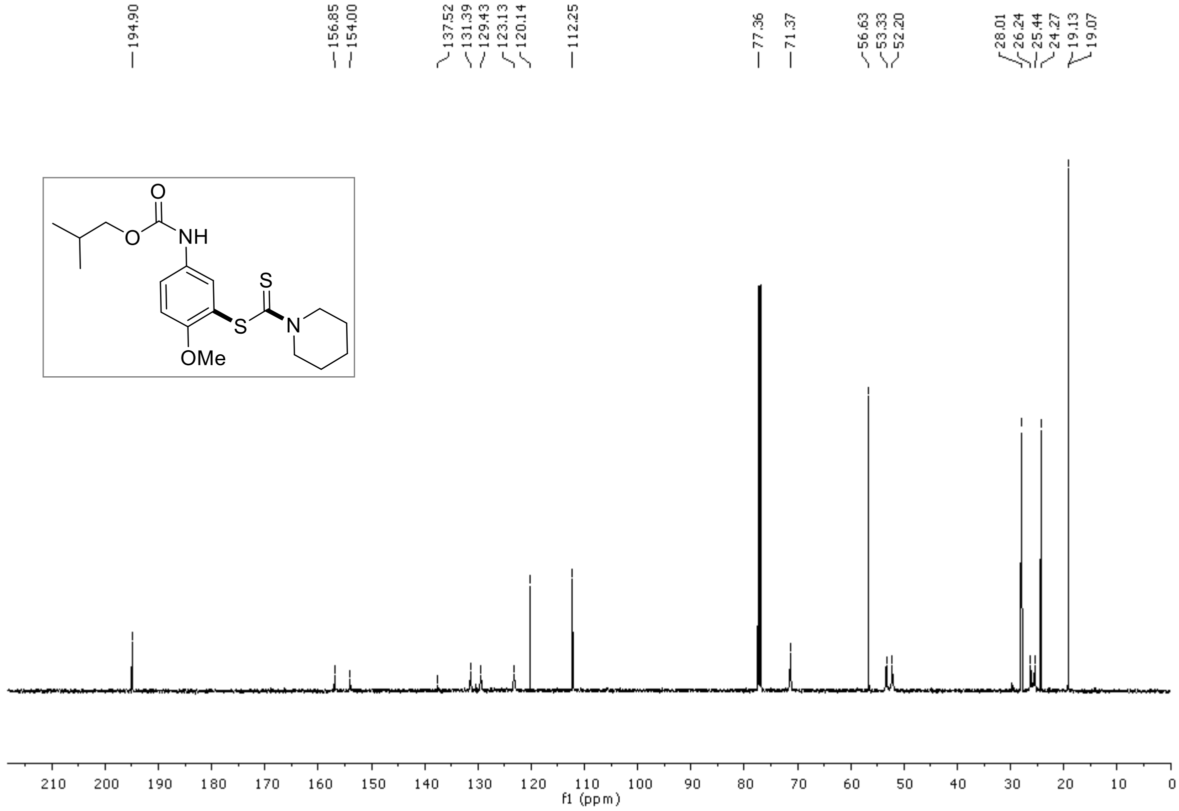

## $^{1}$H and $^{13}$C NMR of 25

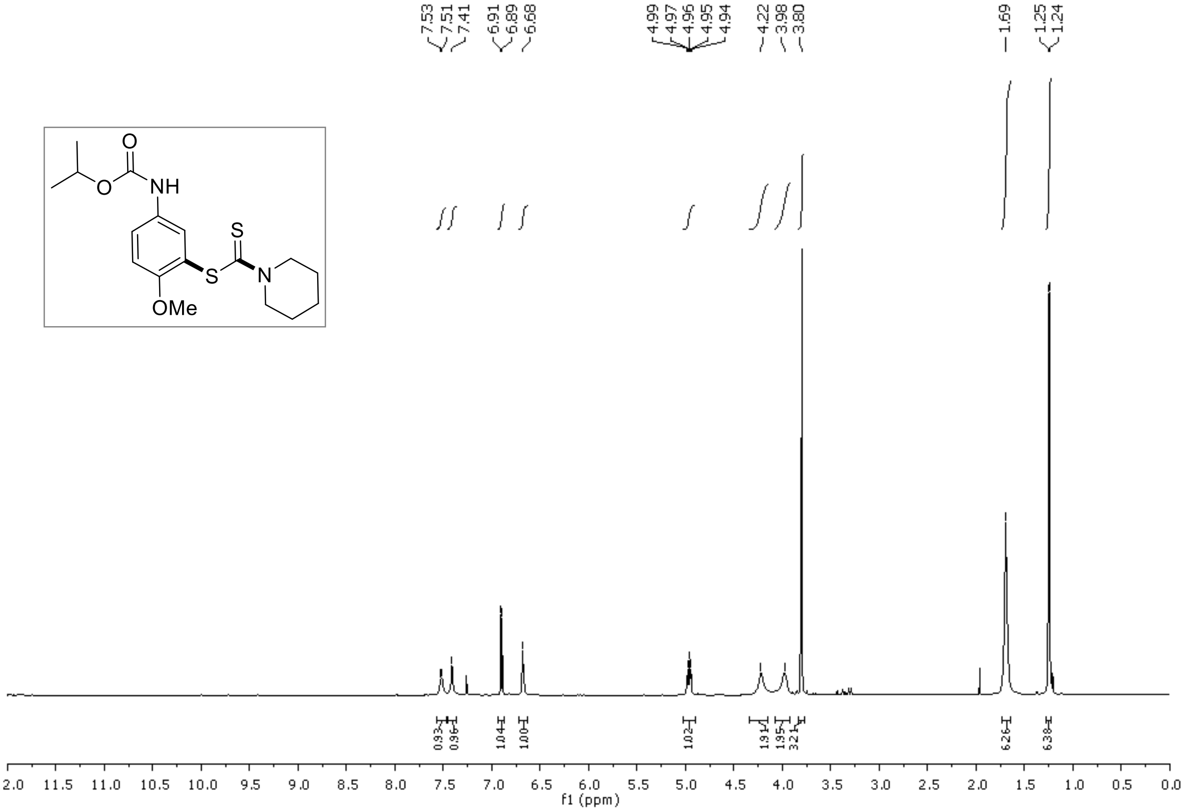


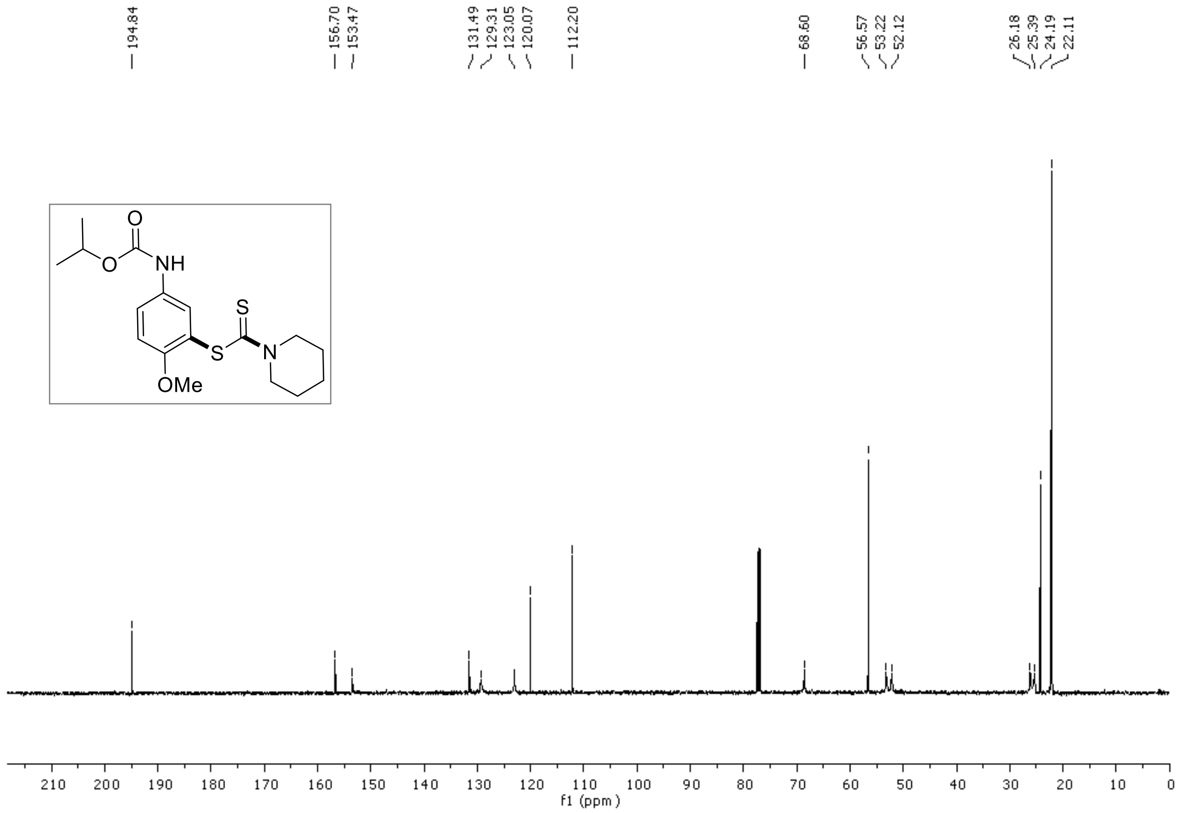

## $^{1}$H and $^{13}$C NMR of 26

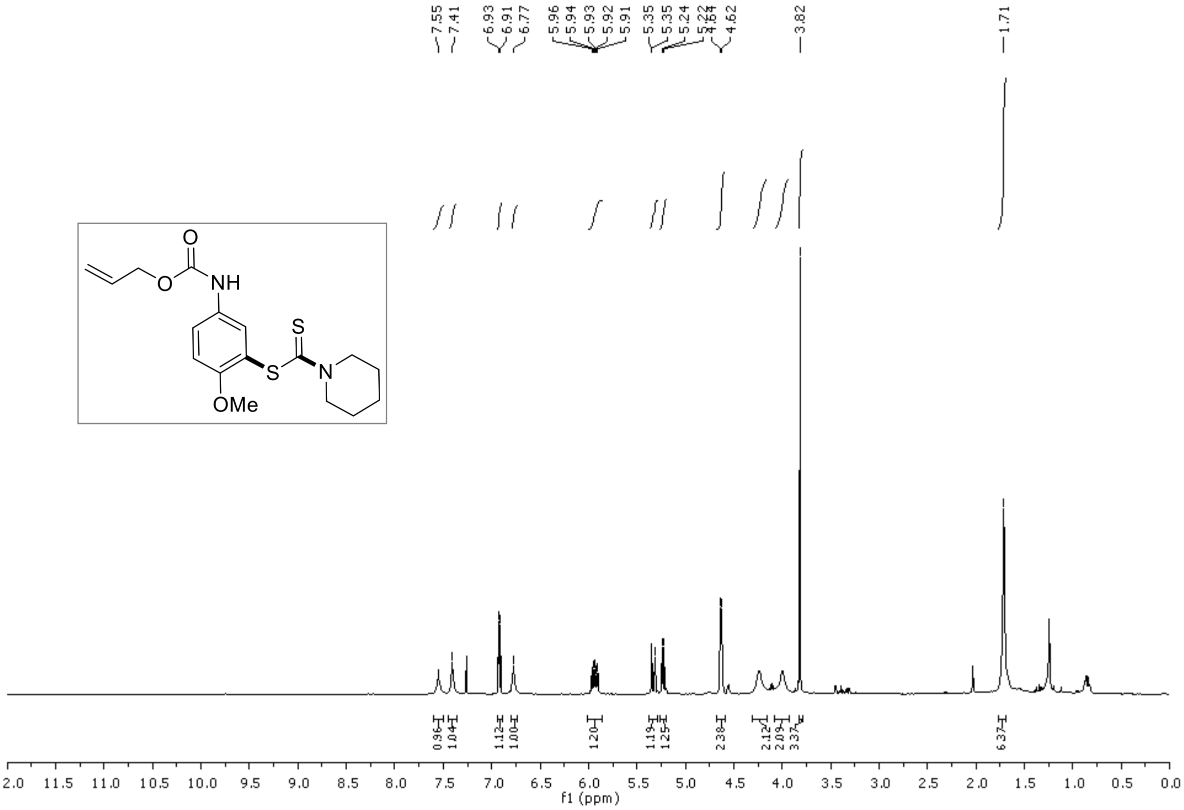


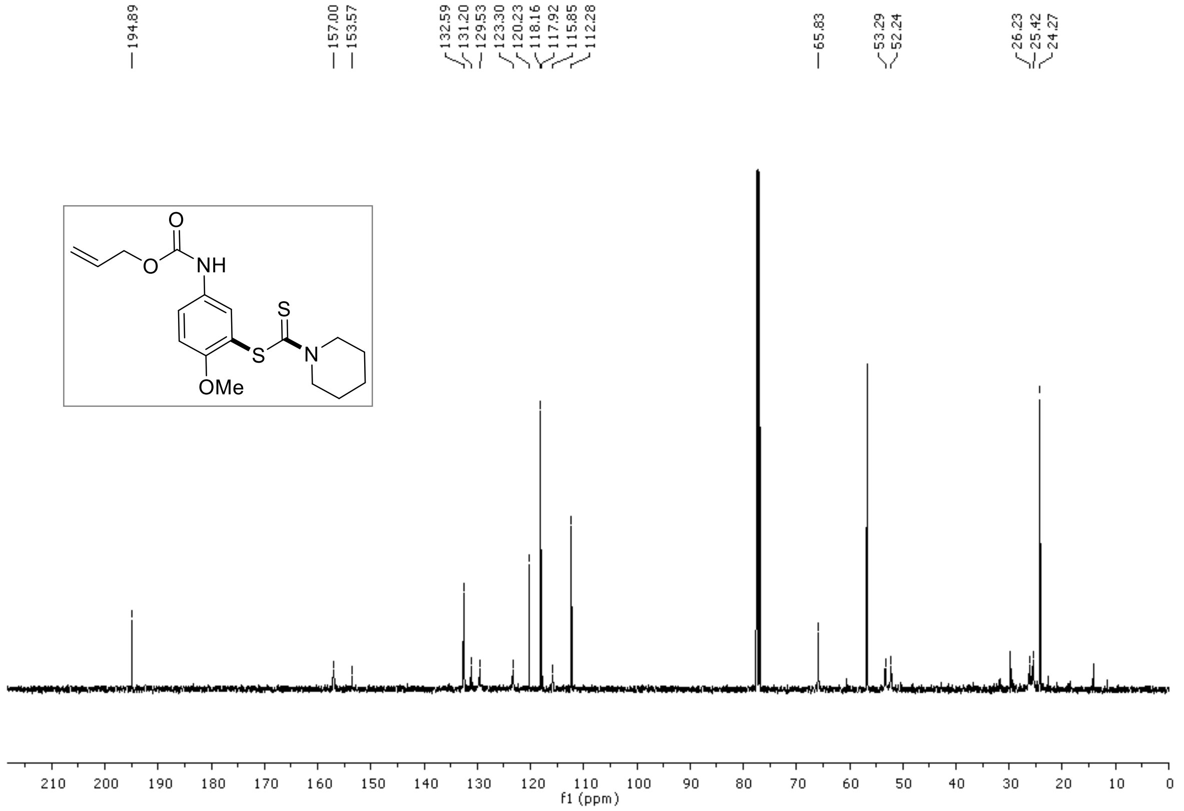

## $^{1}$H and $^{13}$C NMR of 27

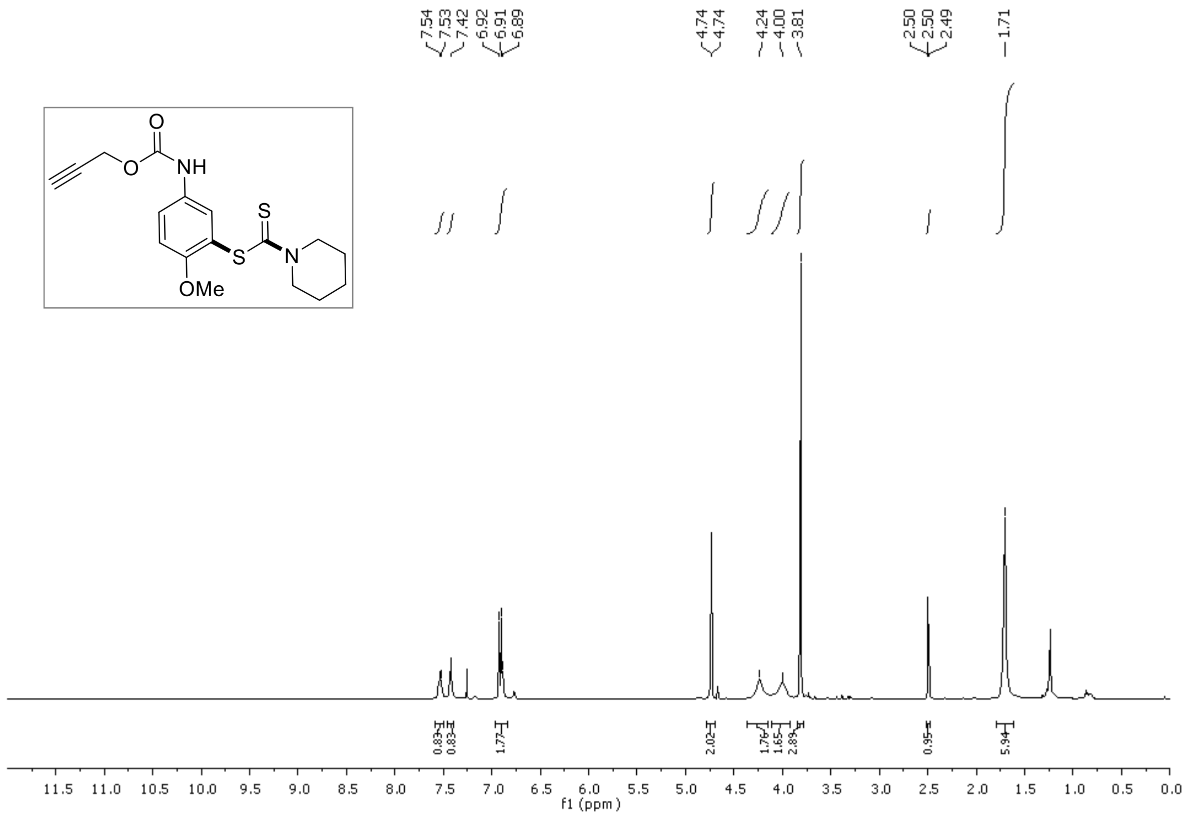


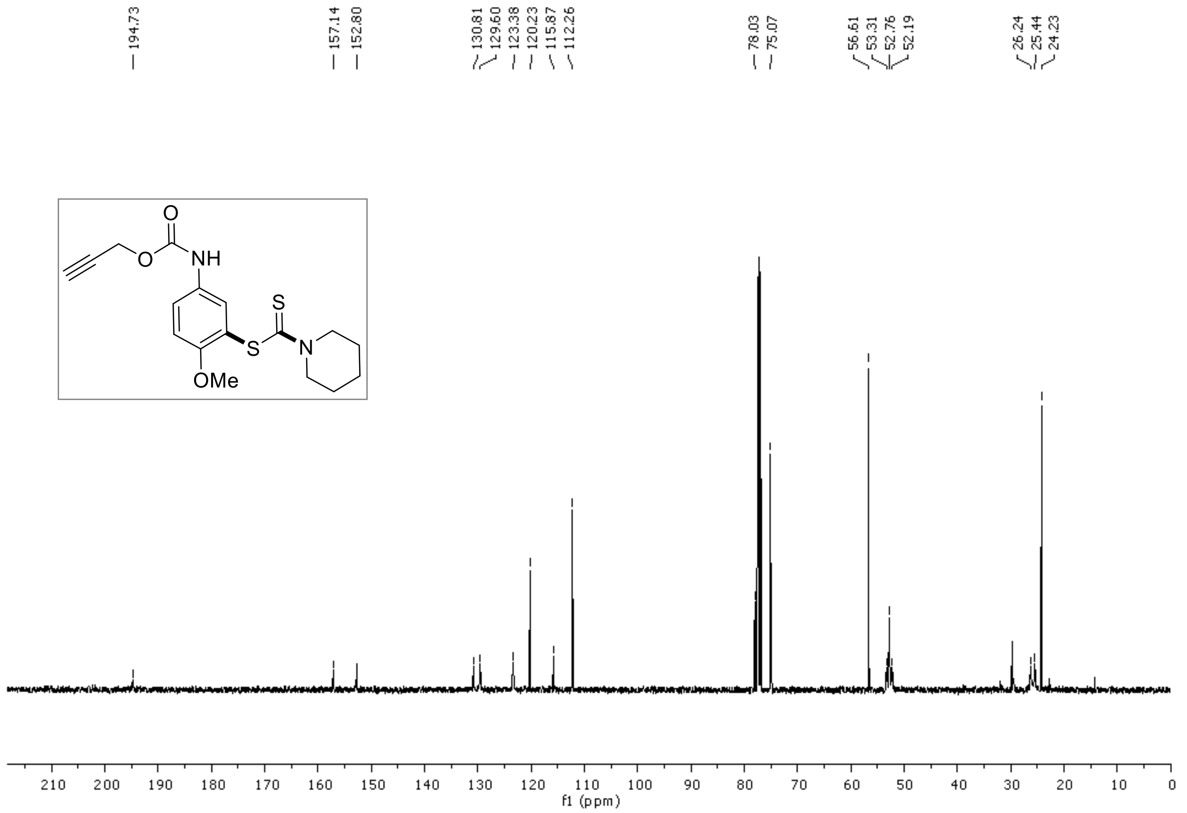

## $^{1}$H and $^{13}$C NMR of 28

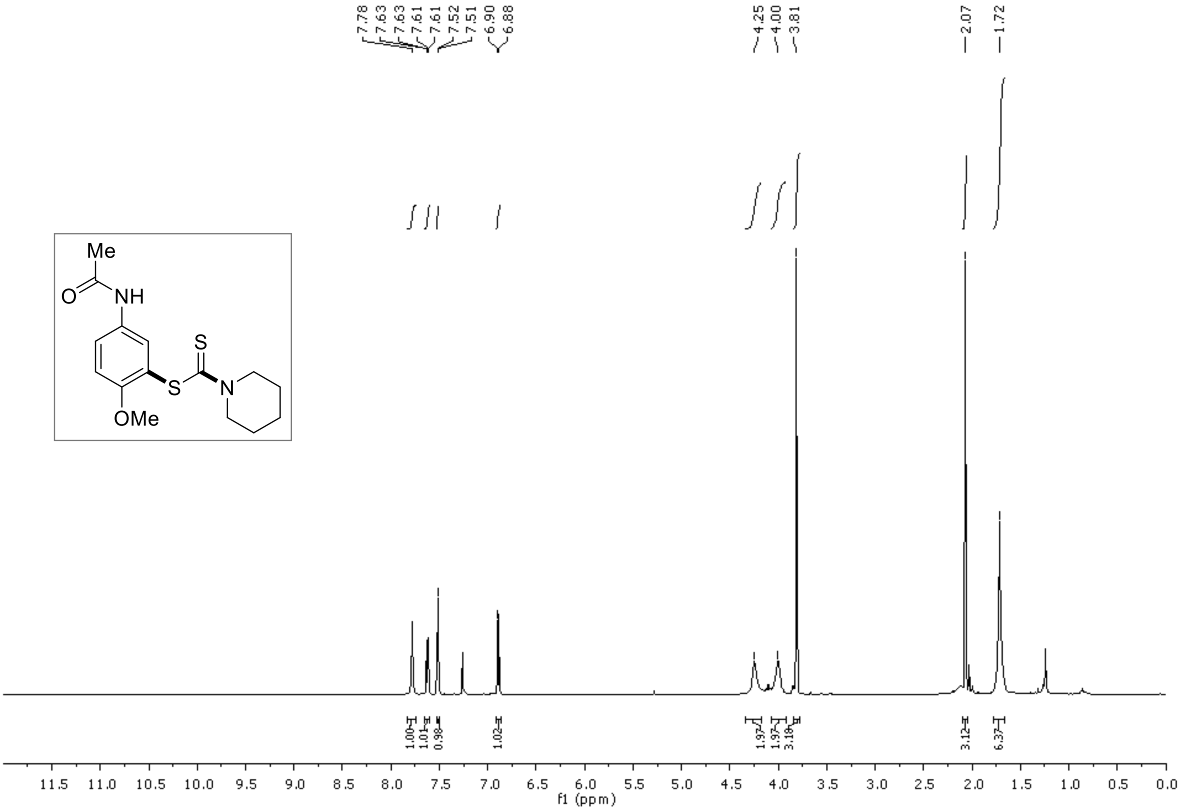


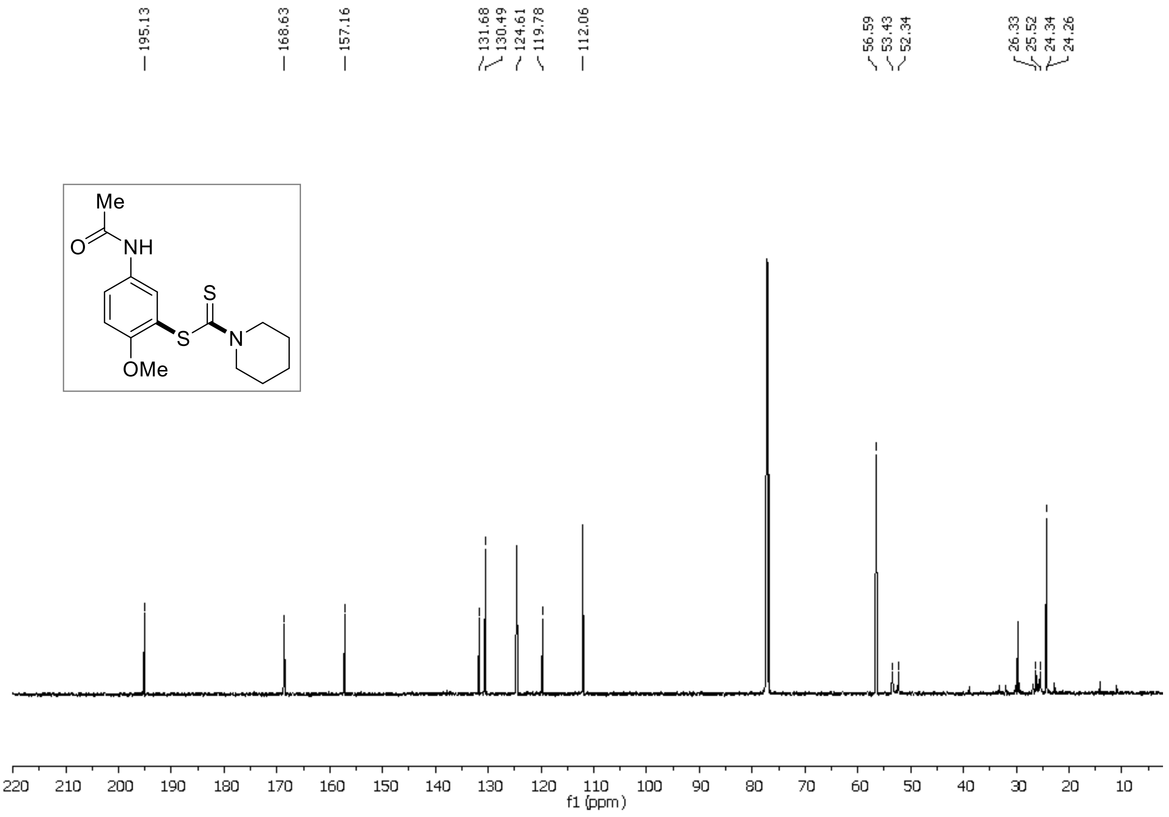

**$^{1}$H and $^{13}$C NMR of 29**

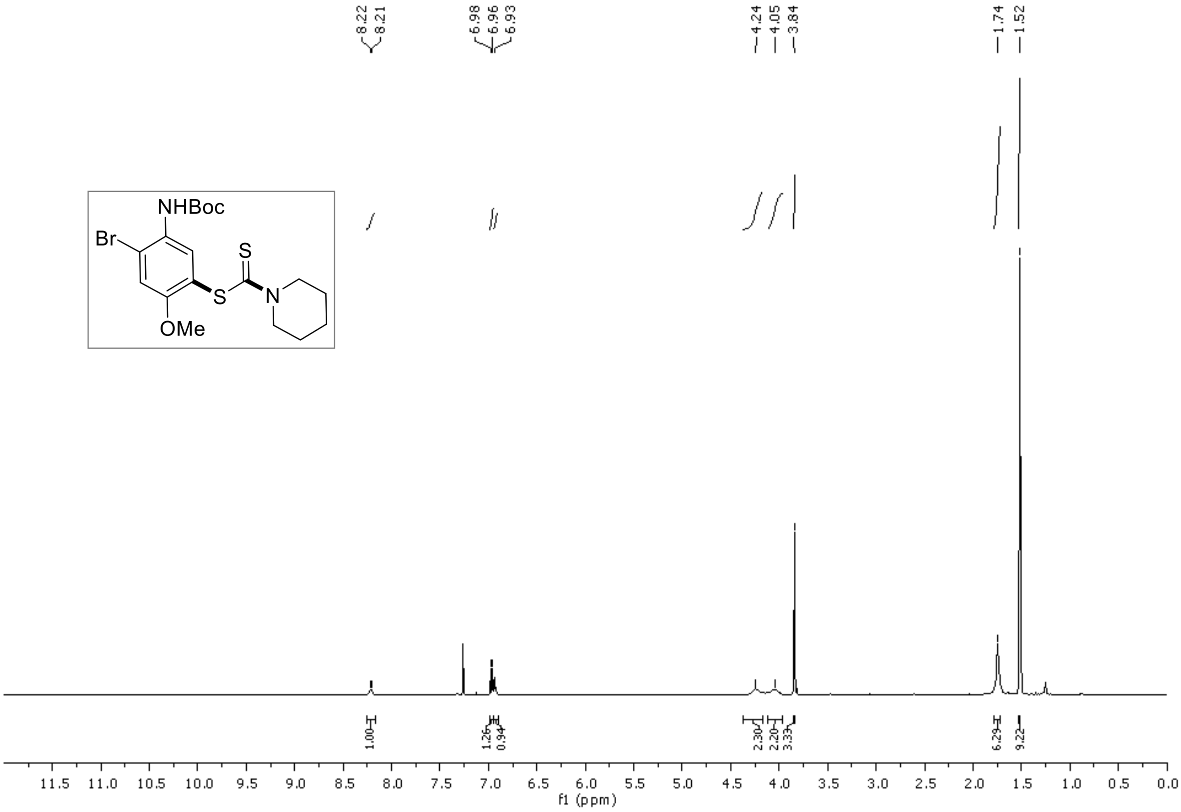


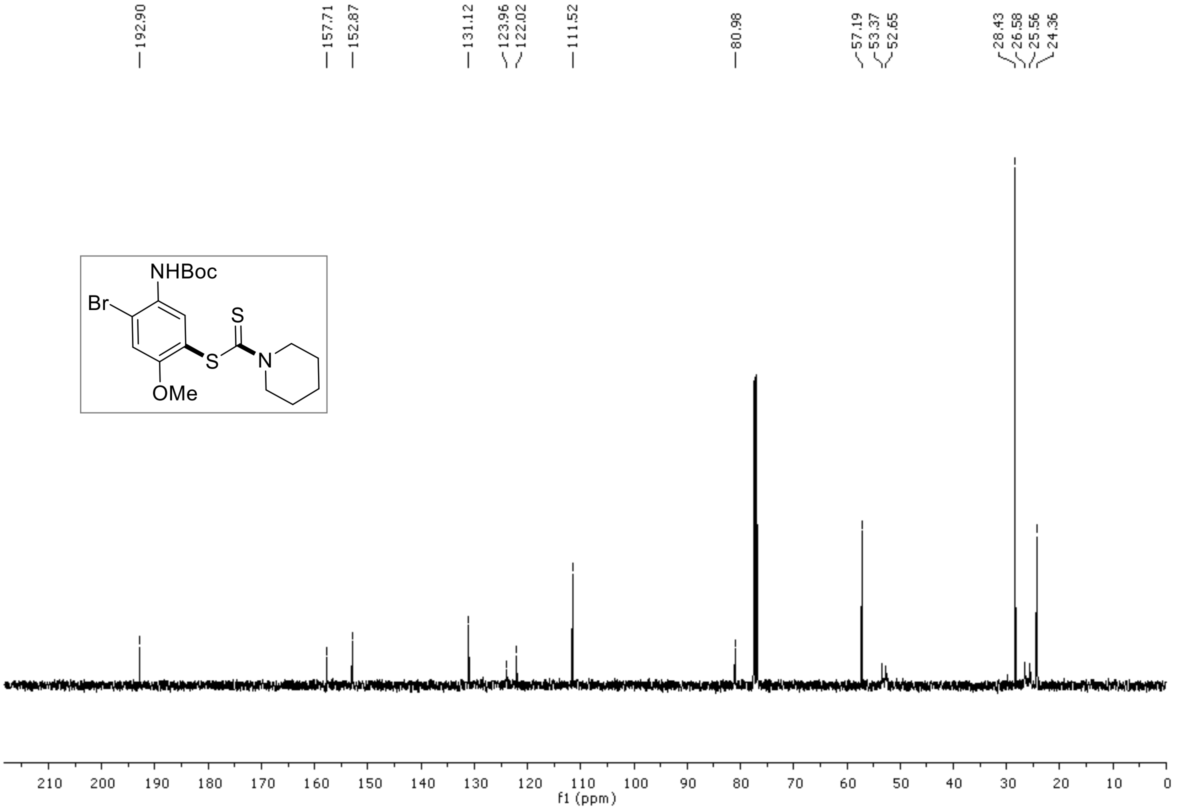

## $^{1}$H and $^{13}$C NMR of 30

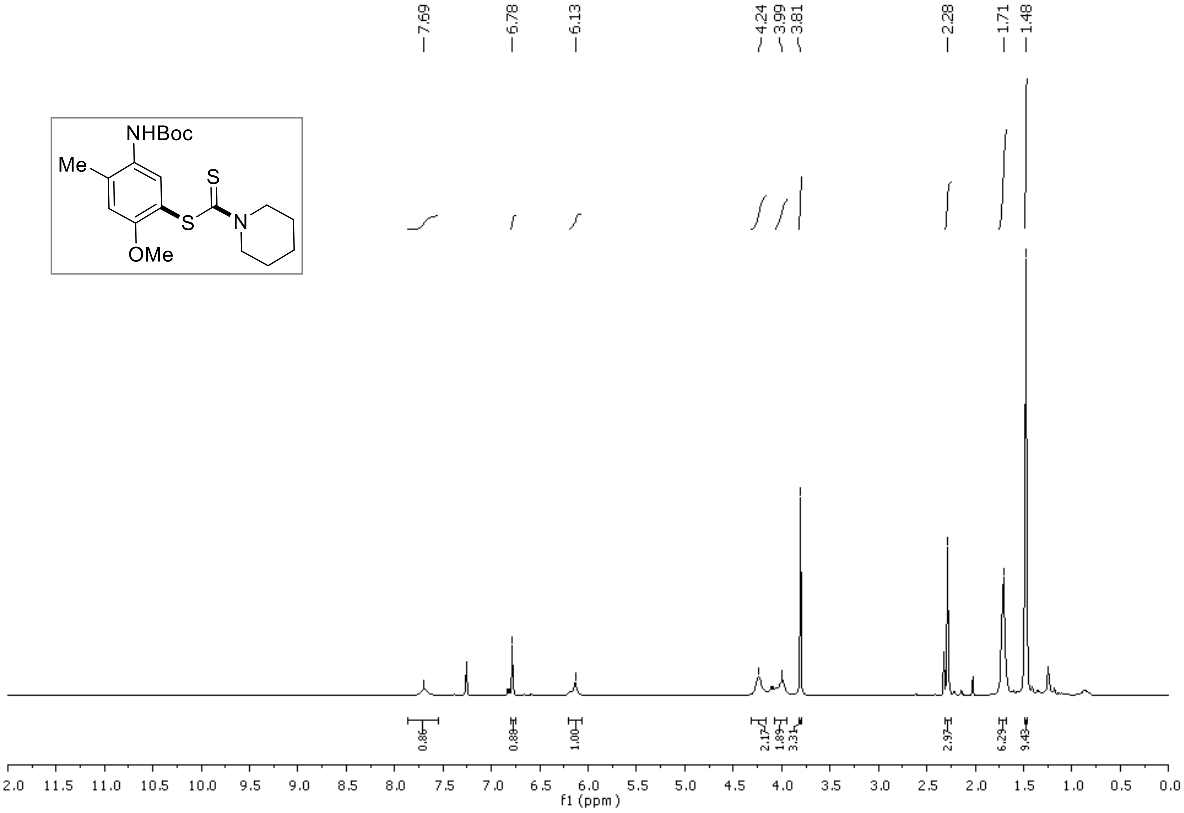


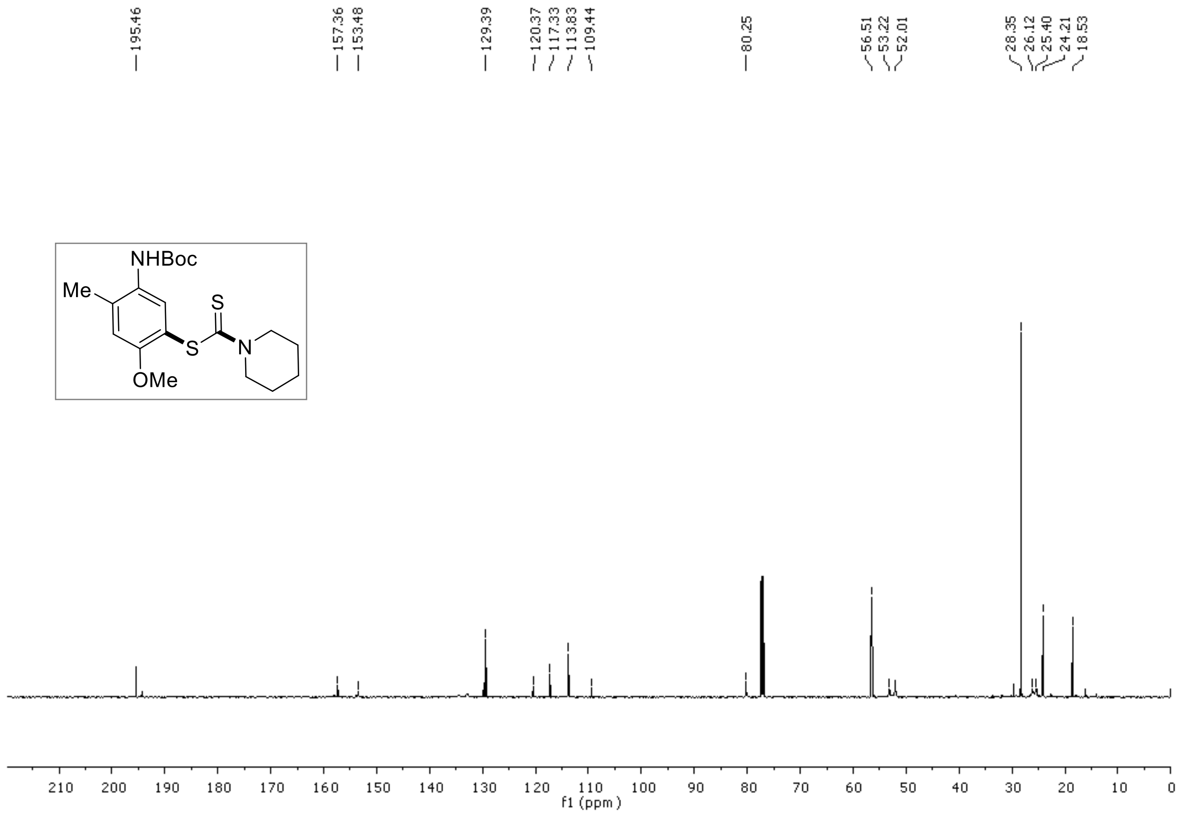

# $^{1}$H and $^{13}$C NMR of 31

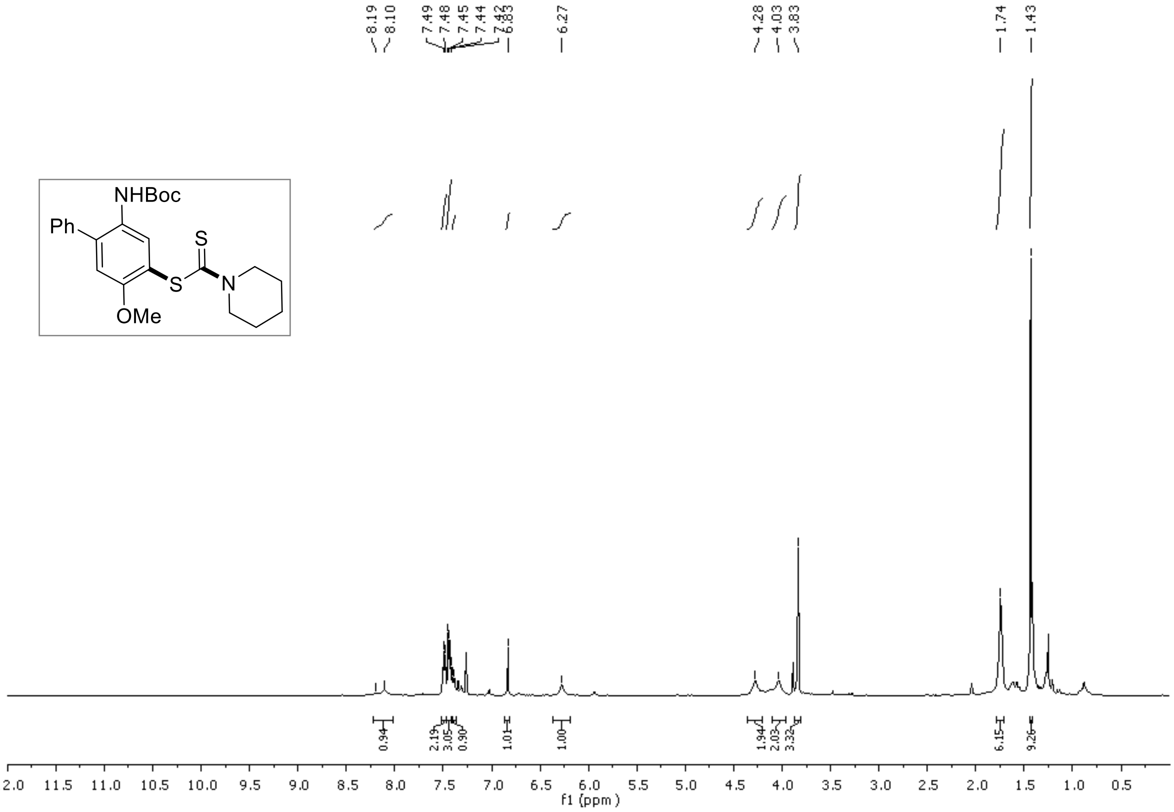


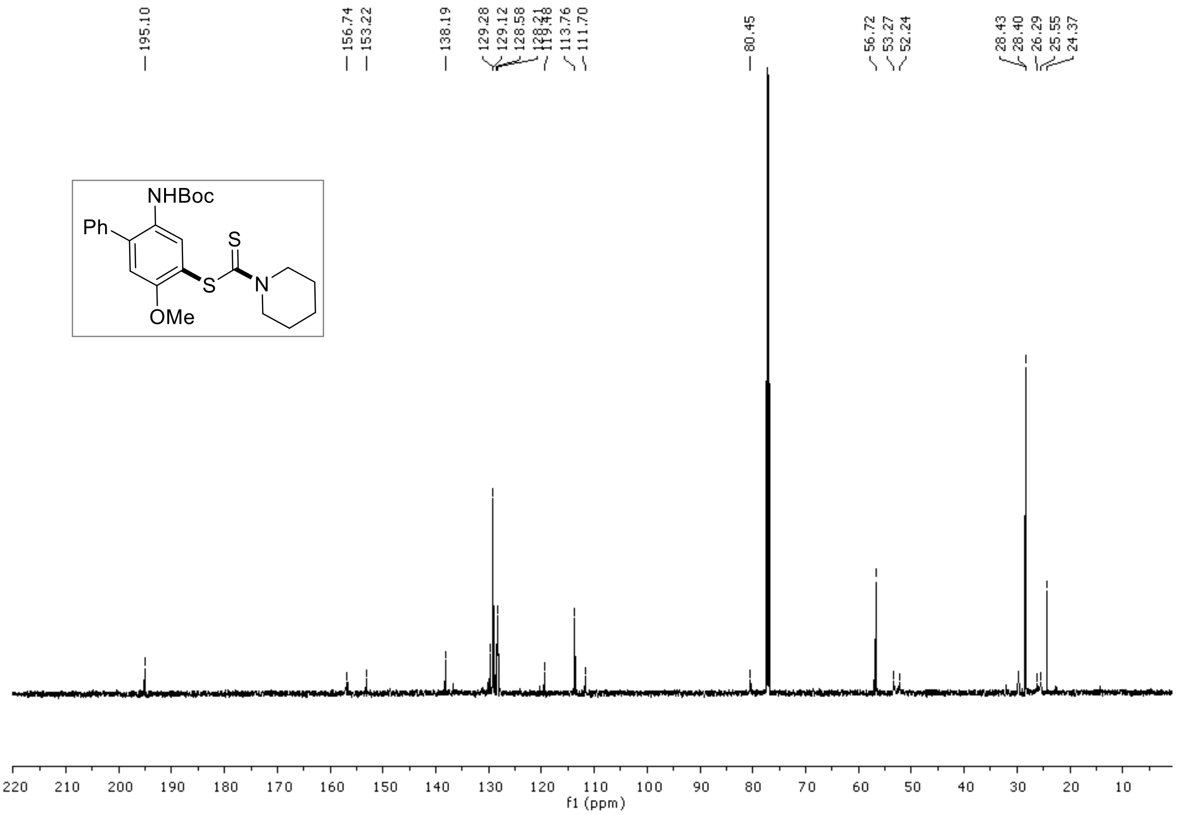

**$^{1}$H and $^{13}$C NMR of 32**

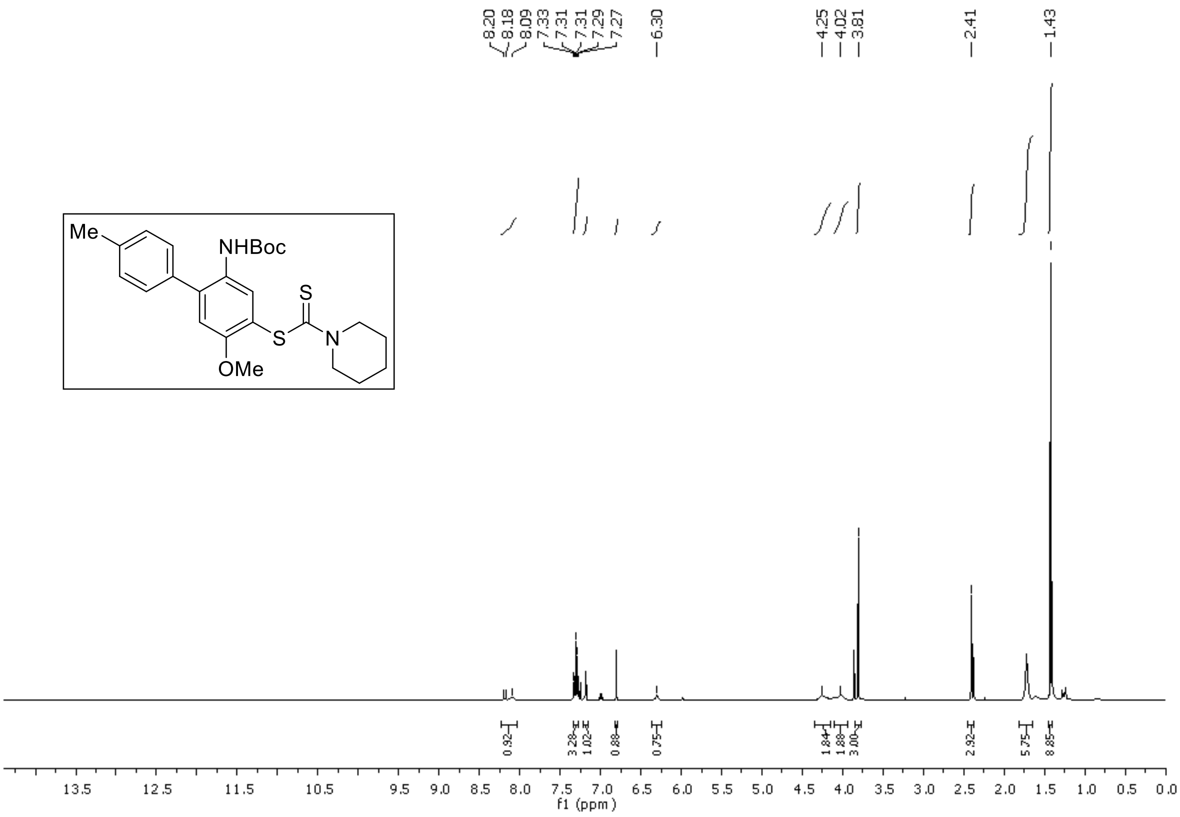

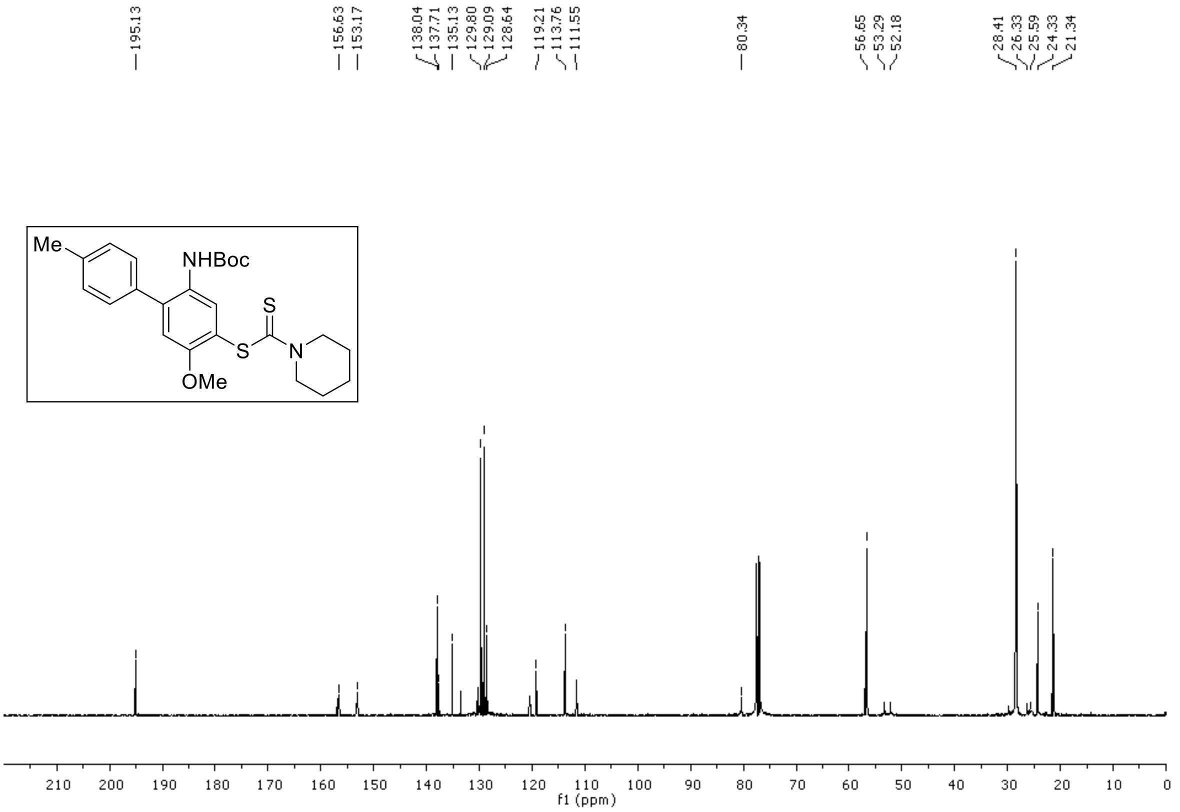

# $^{1}H$ and $^{13}C$ NMR of 33

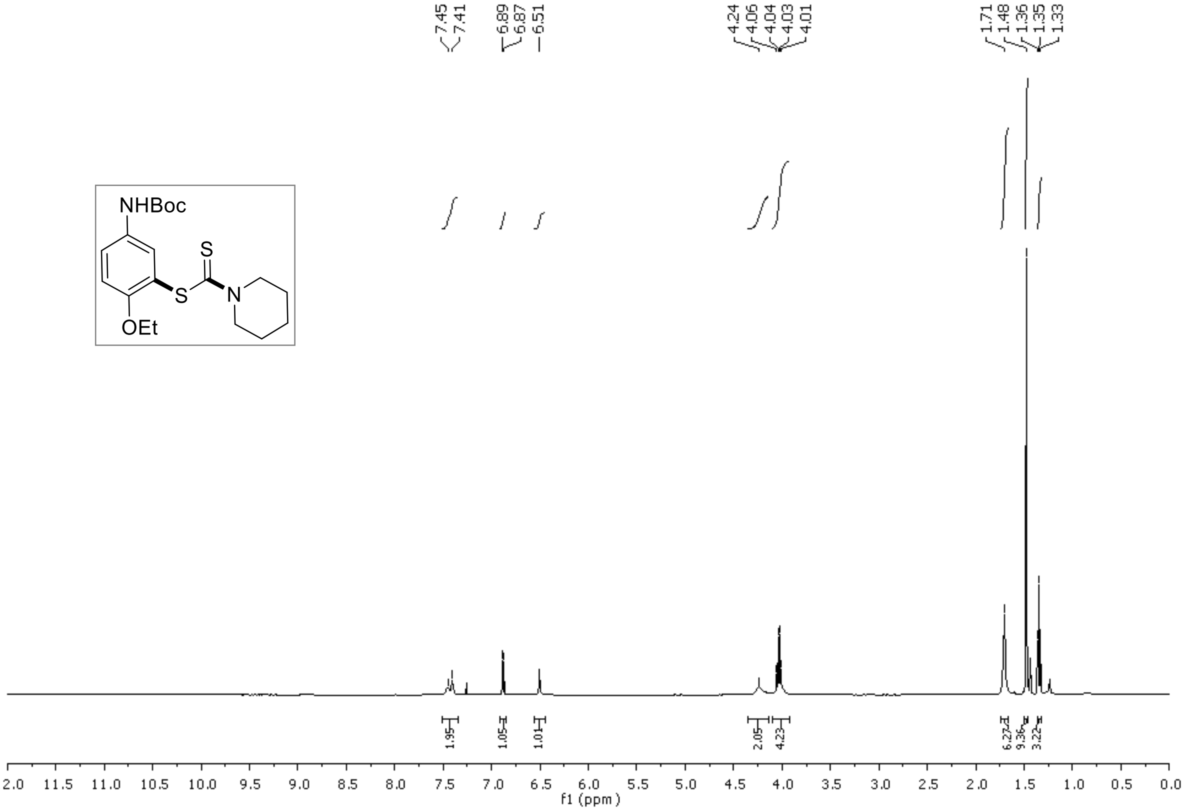


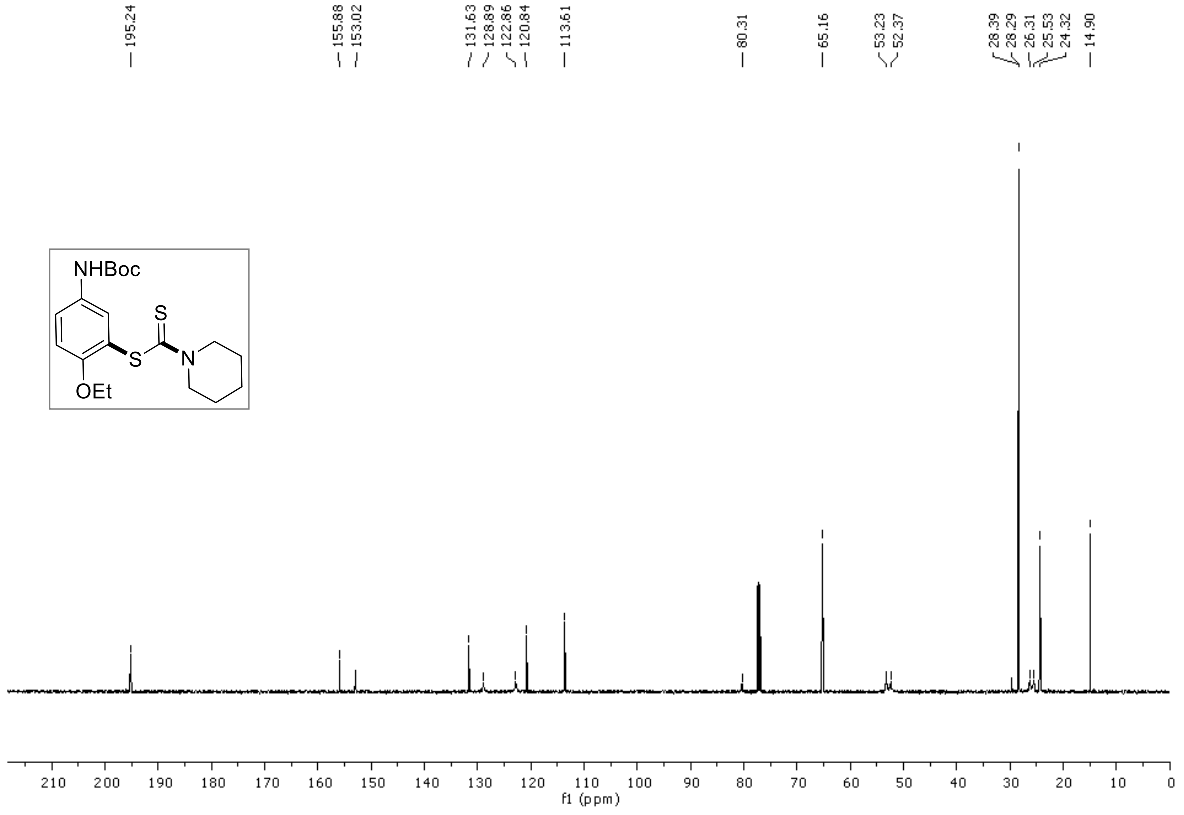

## $^{1}$H and $^{13}$C NMR of 34

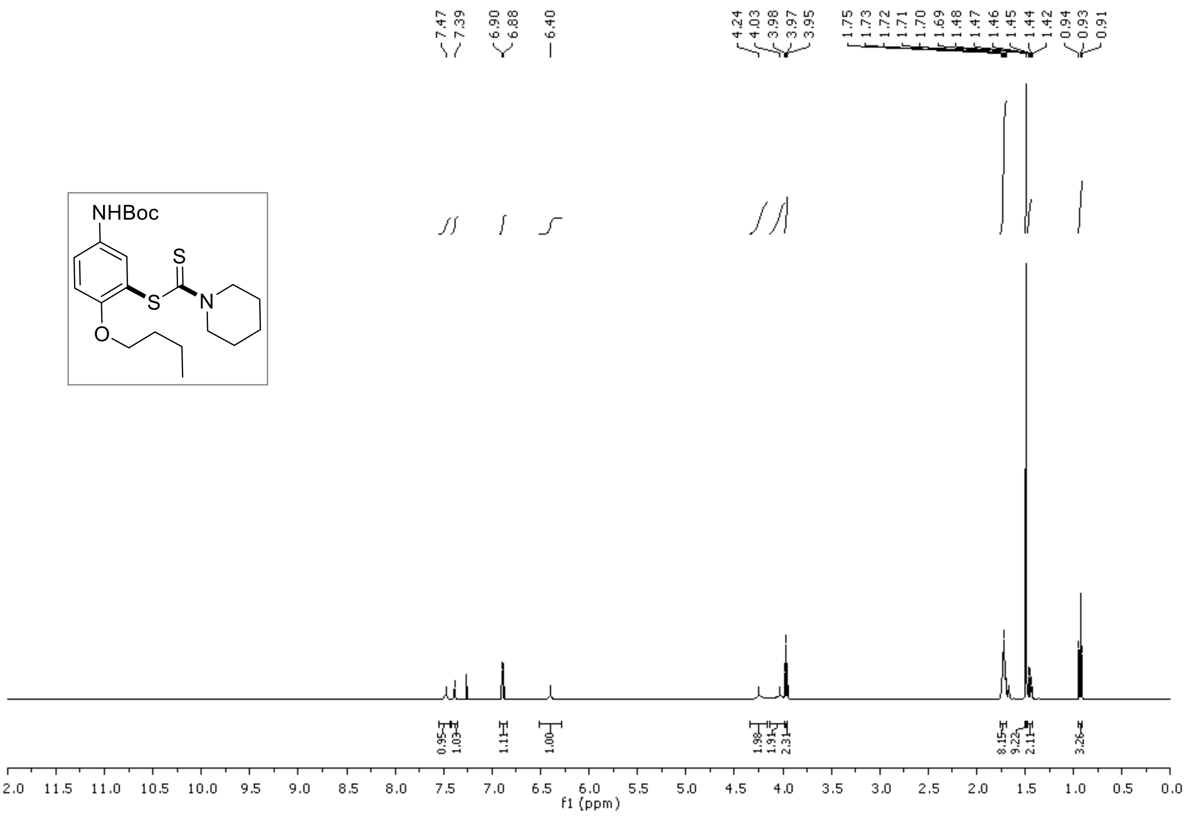


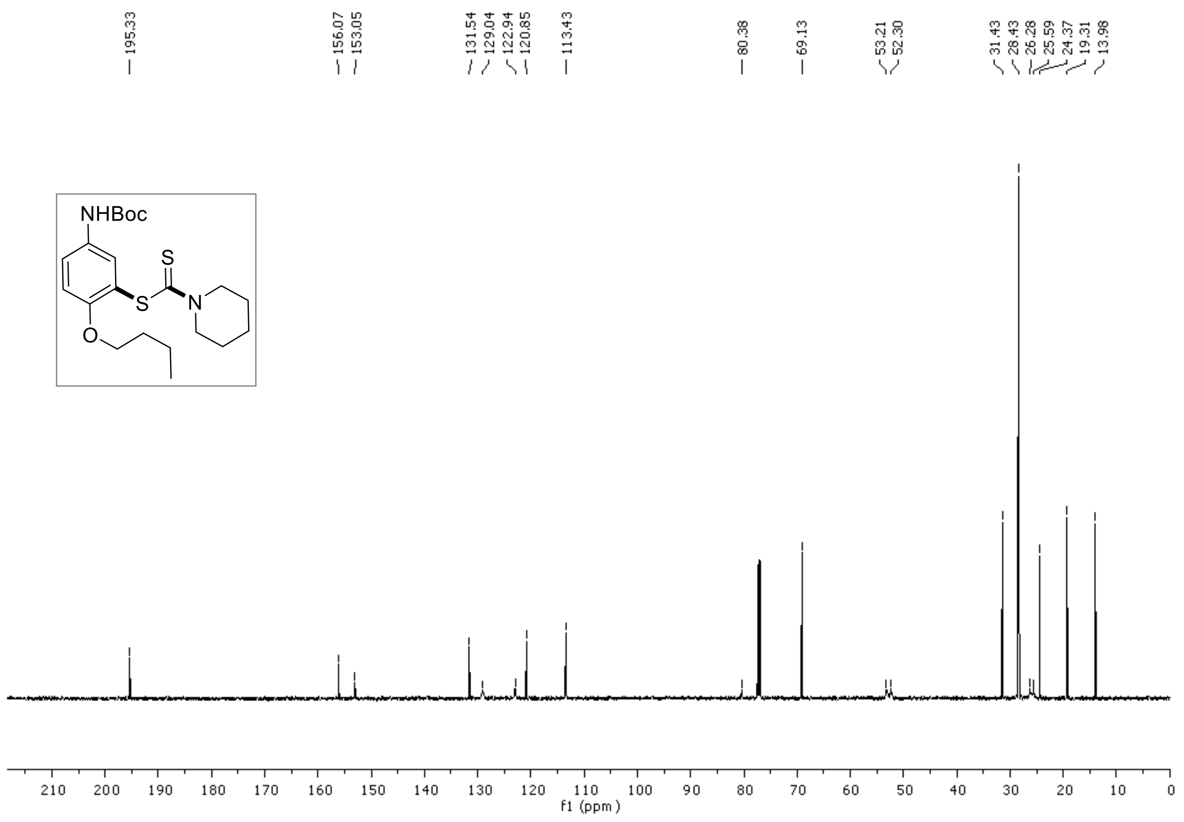

## $^{1}$H and $^{13}$C NMR of 35

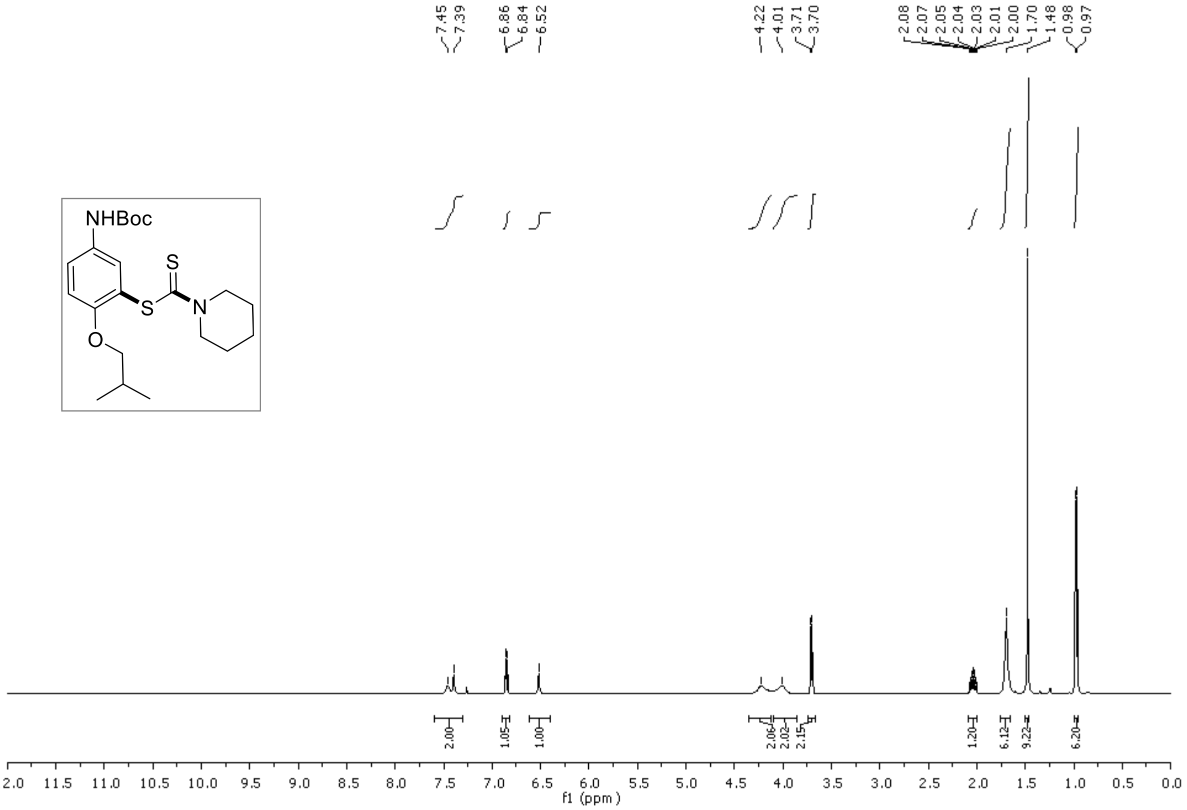


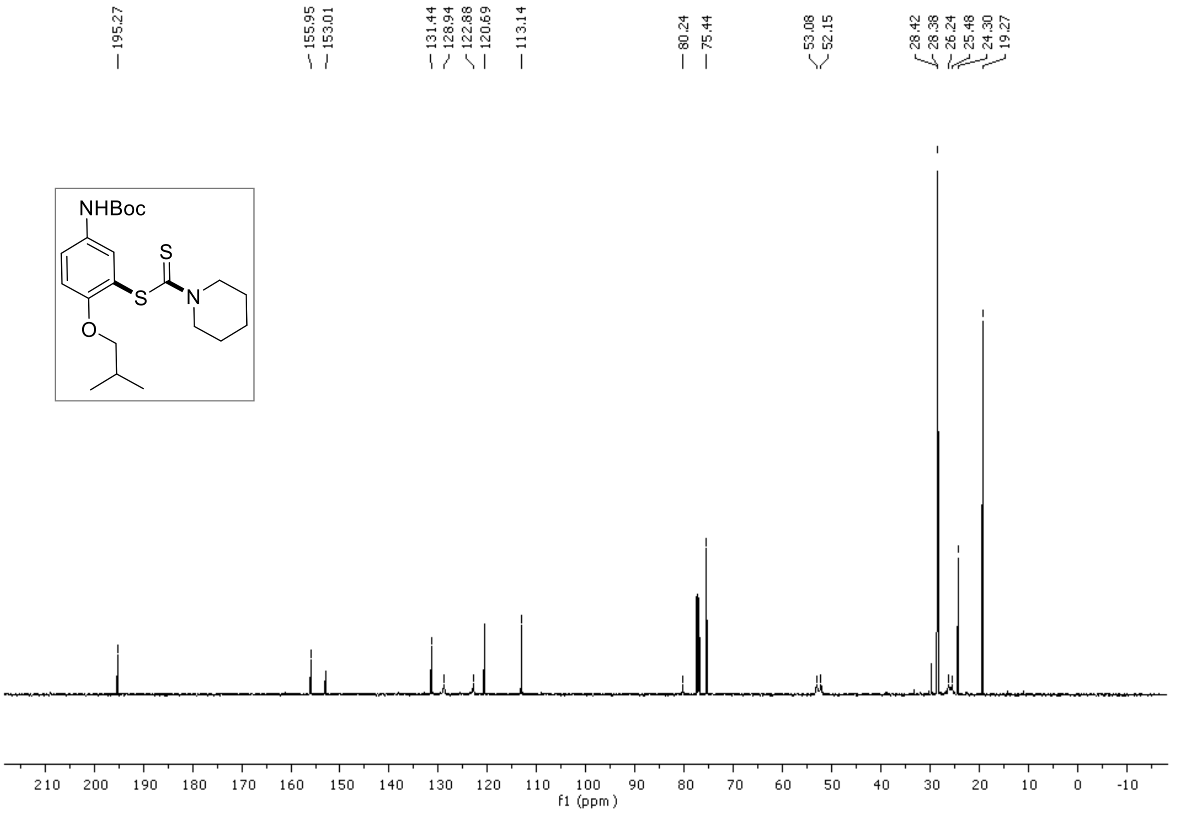

## $^1$H and $^{13}$C NMR of 36

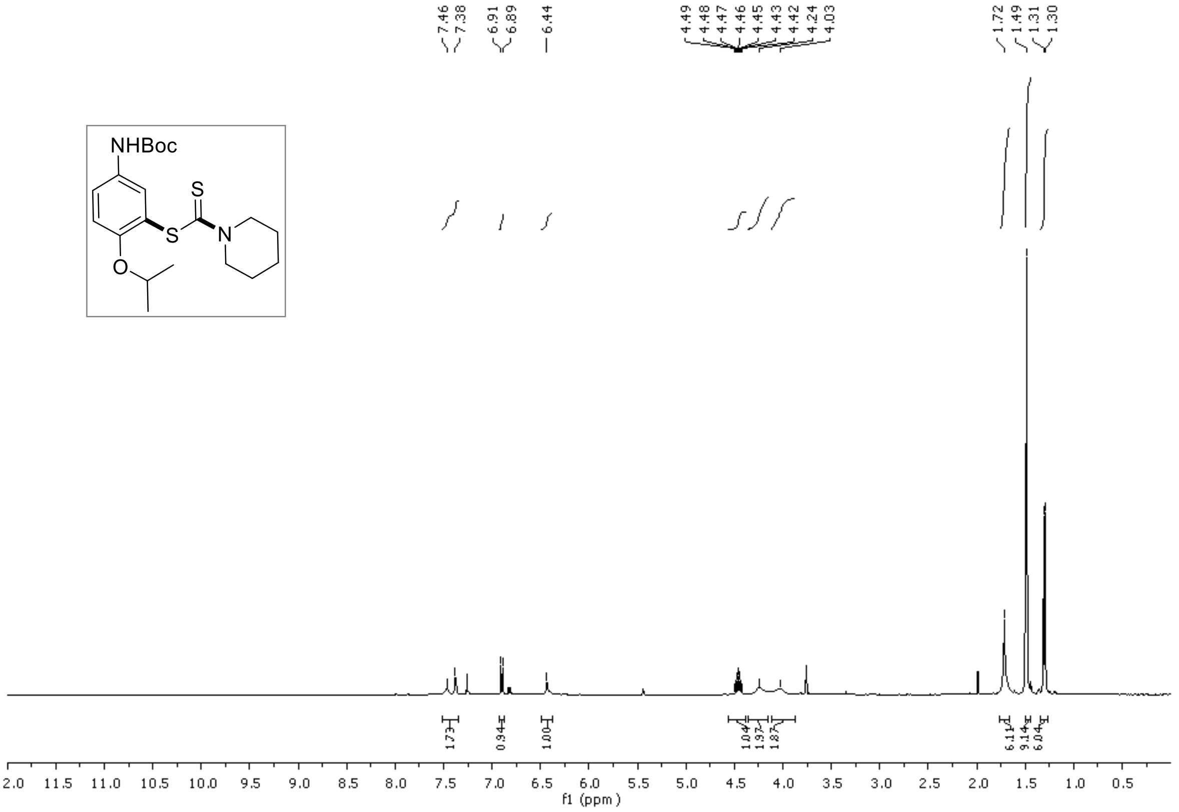


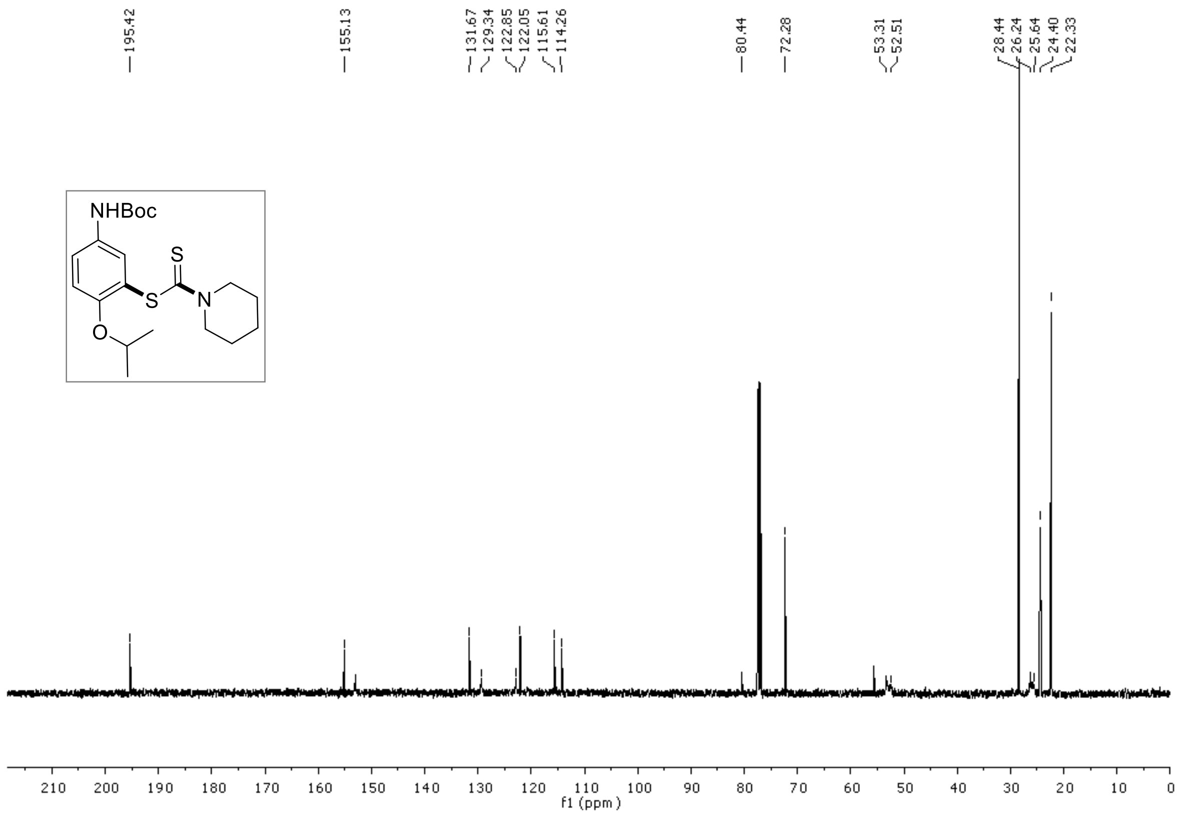

**$^{1}$H and $^{13}$C NMR of 37**

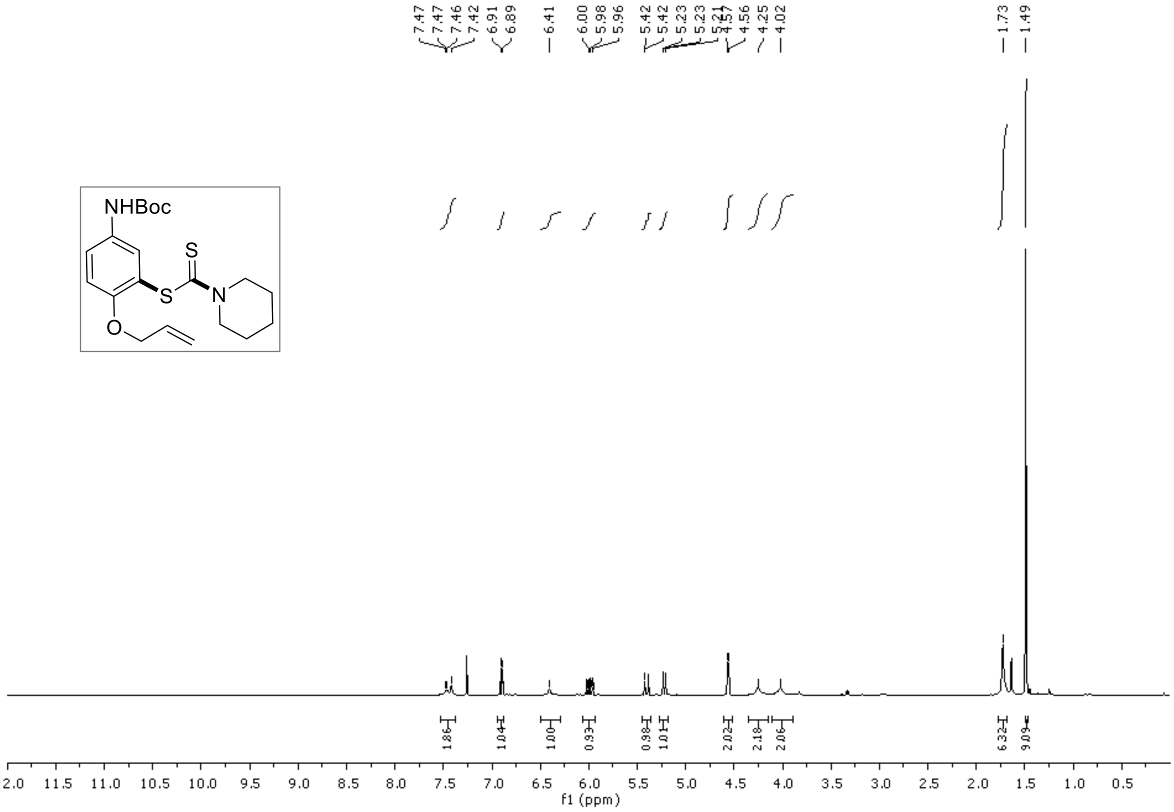


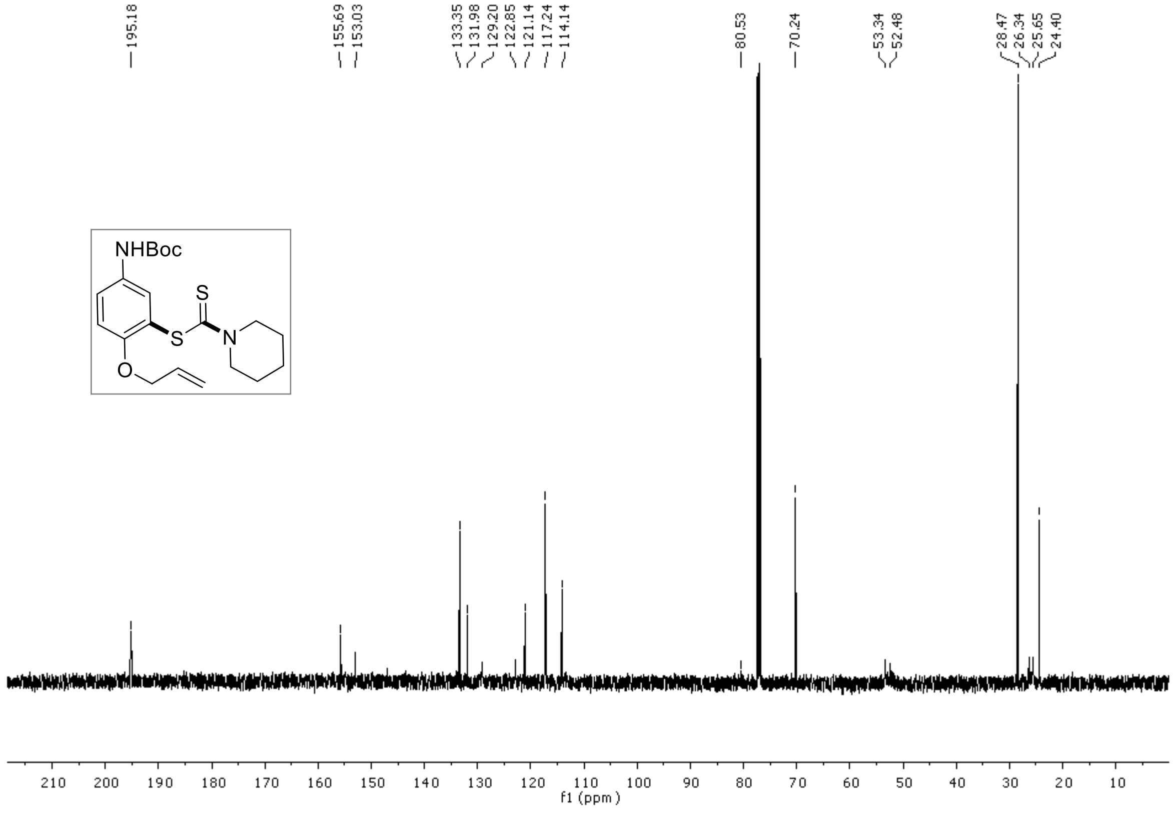

# $^{1}H$ and $^{13}C$ NMR of 38

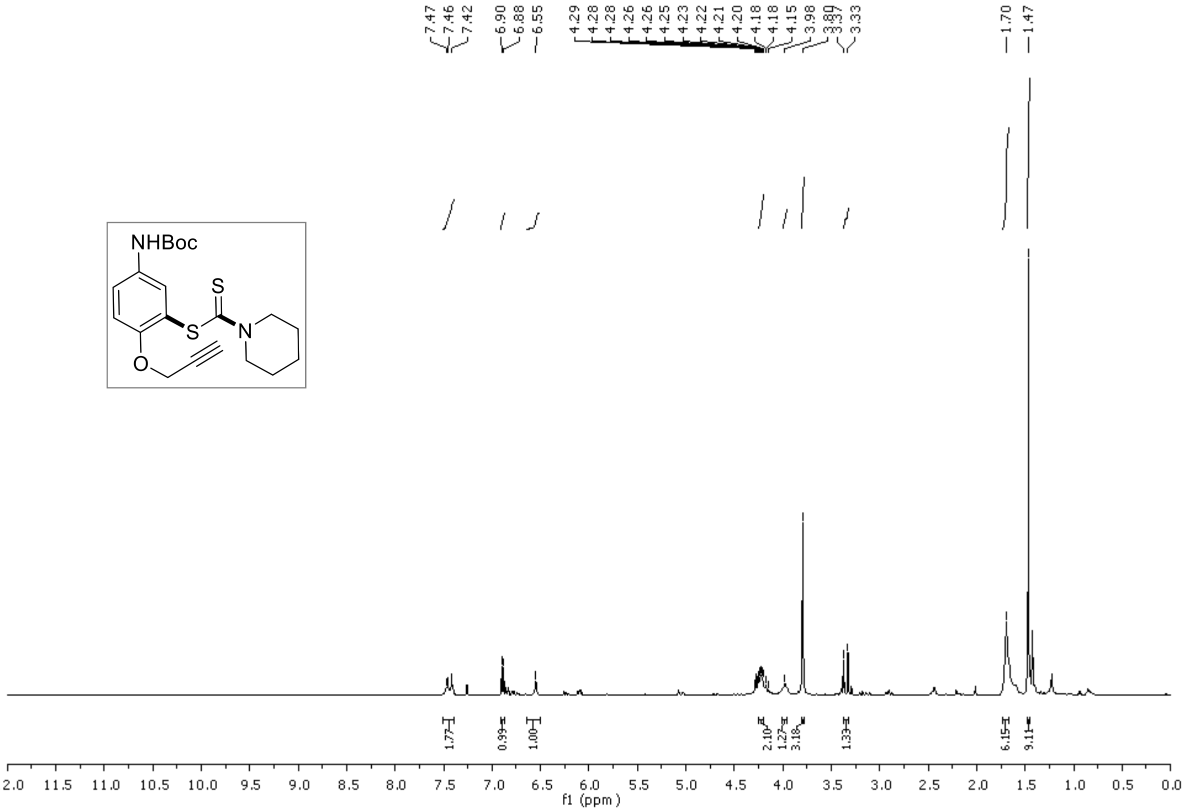


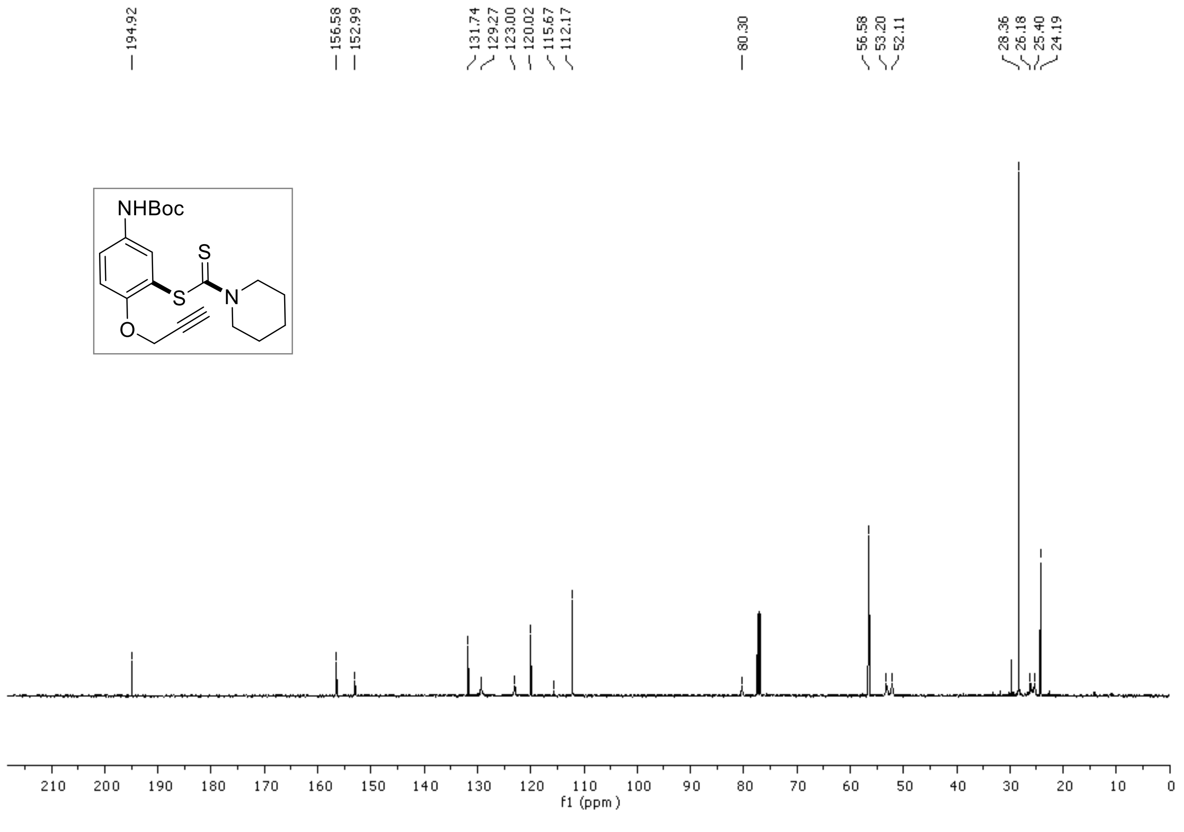

## $^{1}$H and $^{13}$C NMR of 39

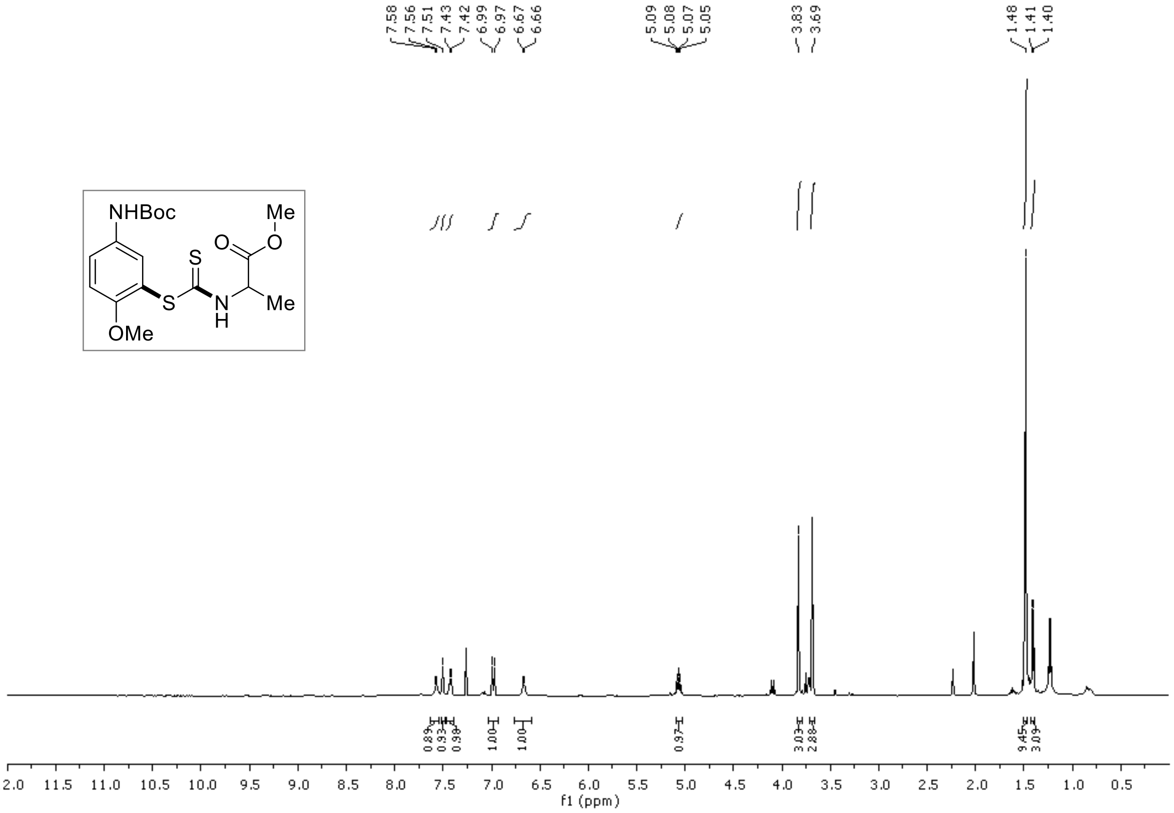


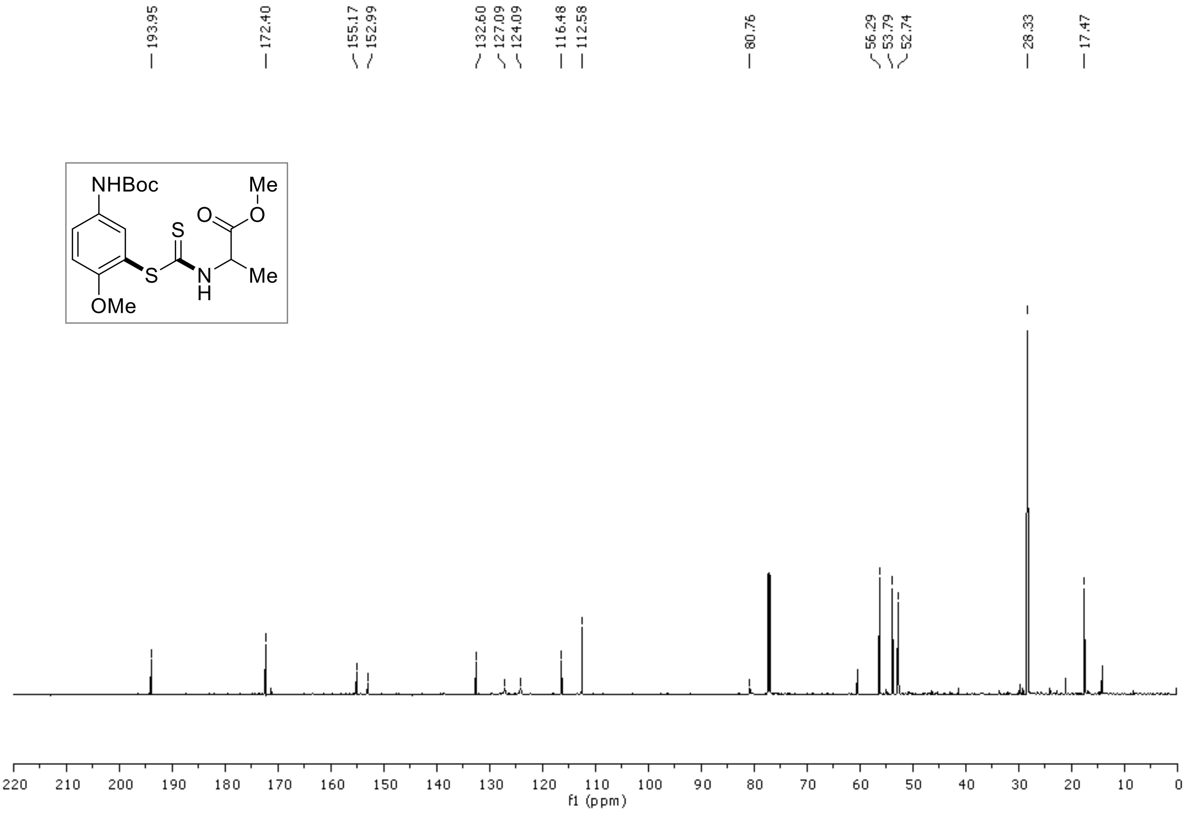

# $^{1}H$ and $^{13}C$ NMR of 40

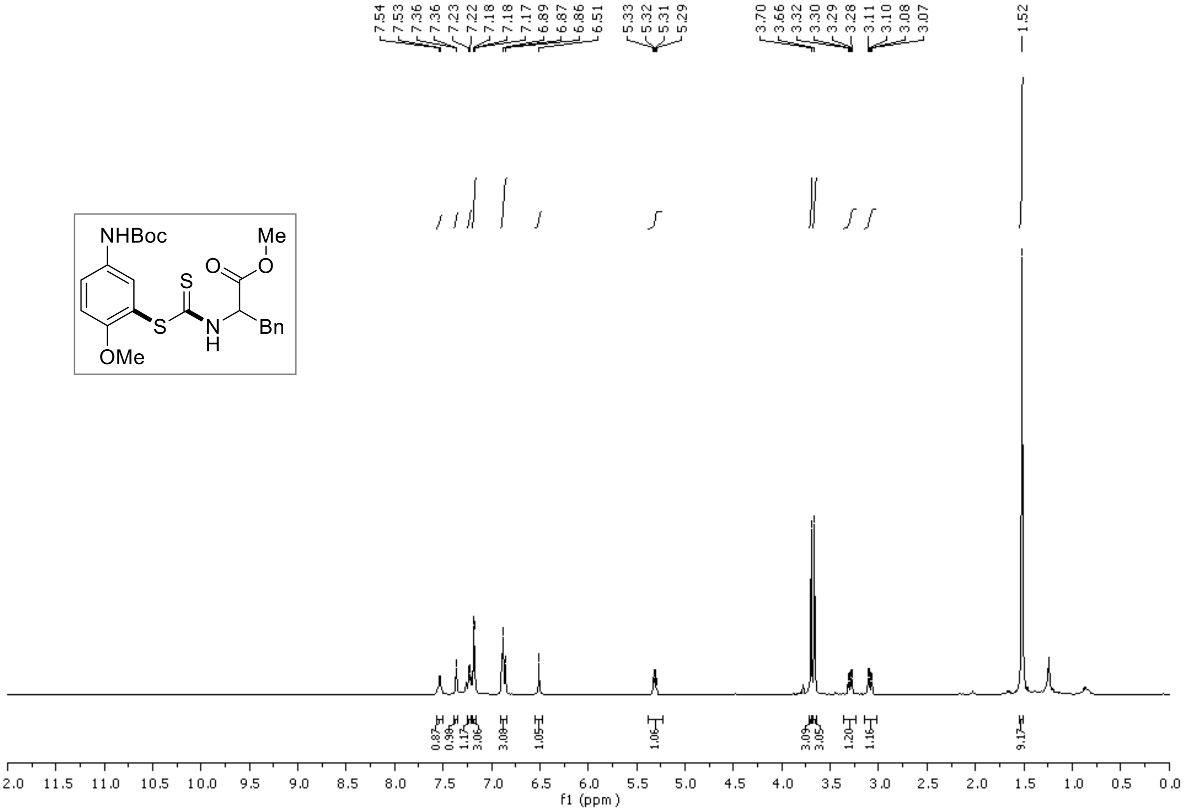


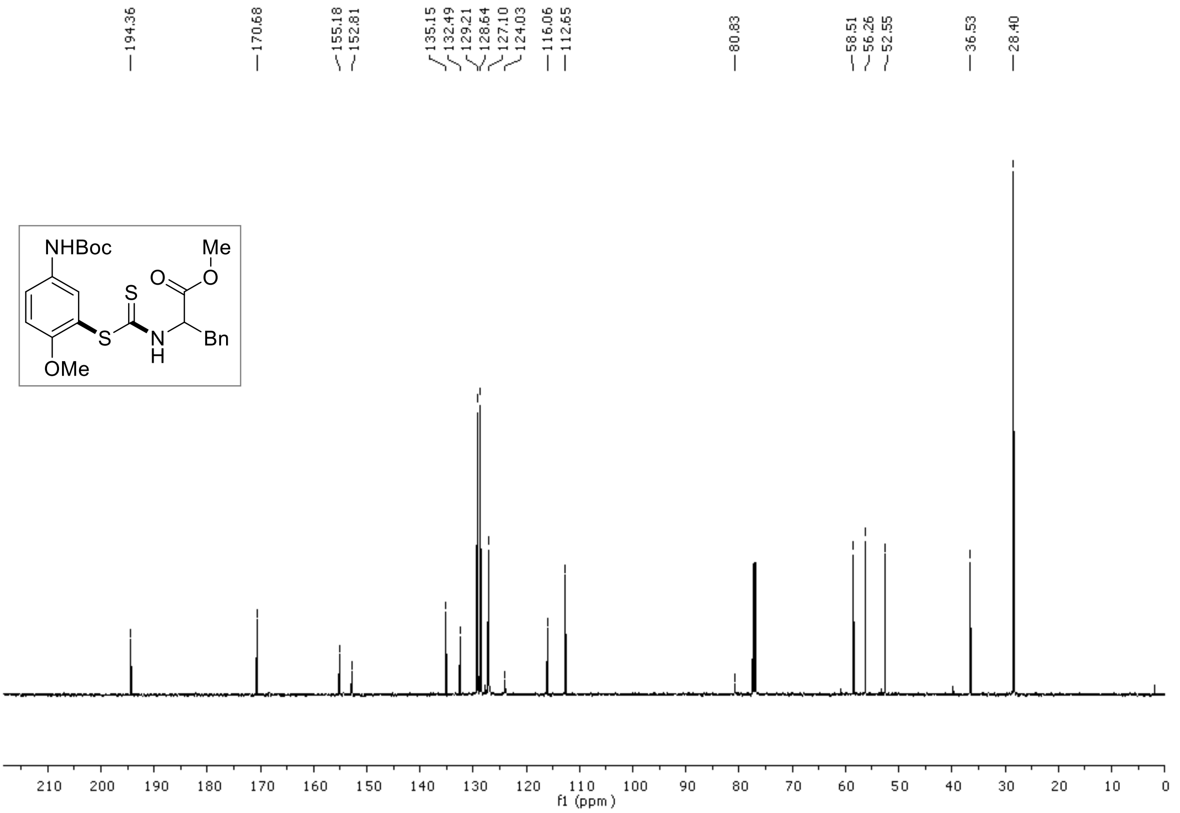

## $^{1}$H and $^{13}$C NMR of 41

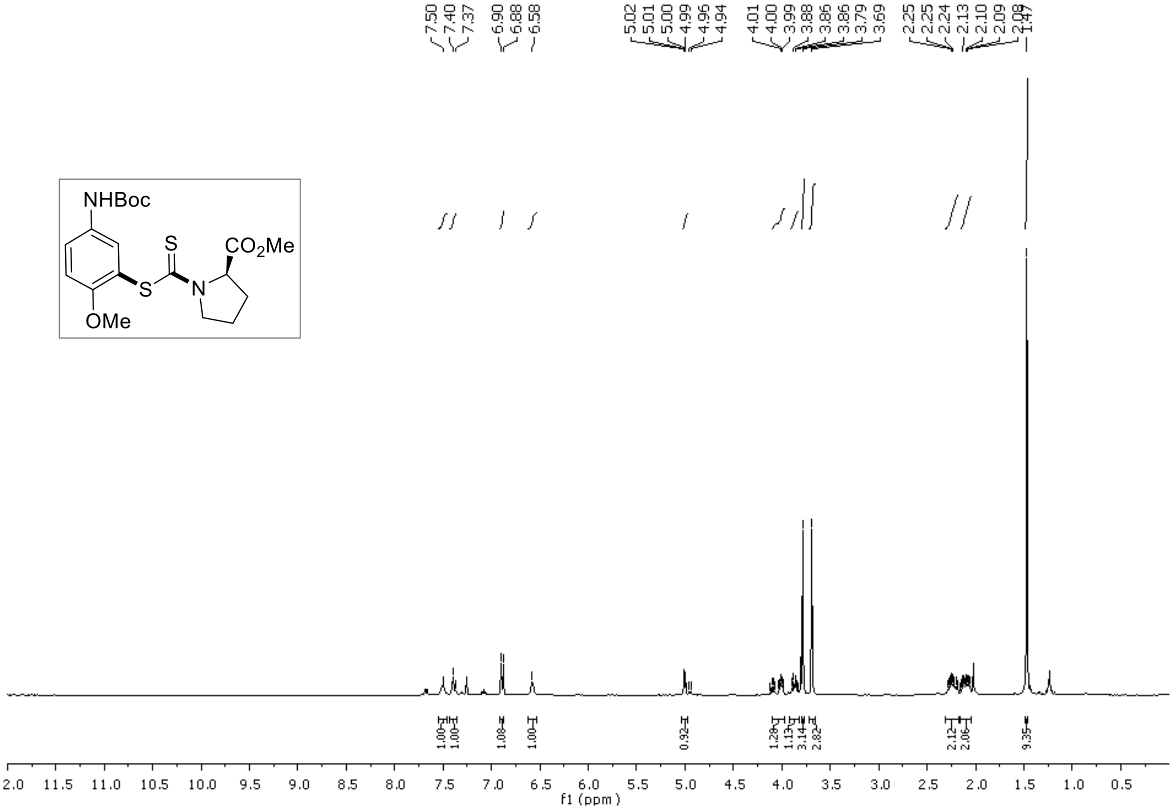


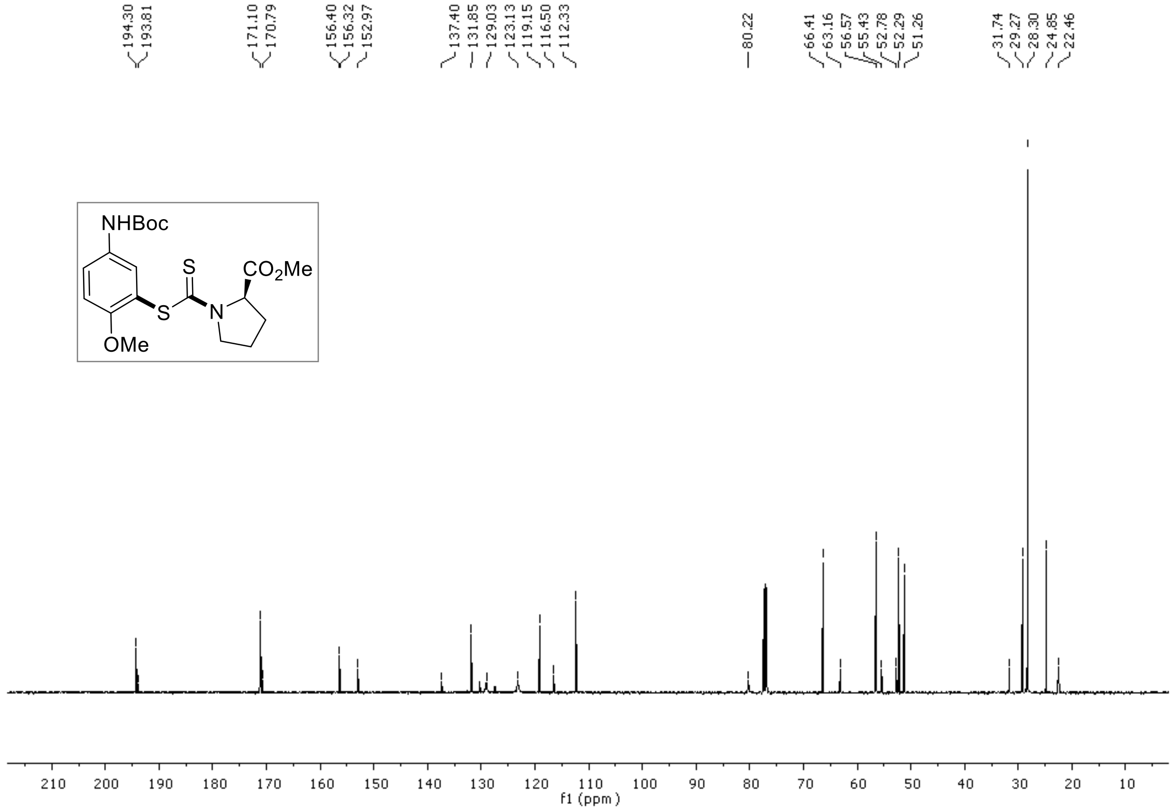

## $^{1}$H and $^{13}$C NMR of 42

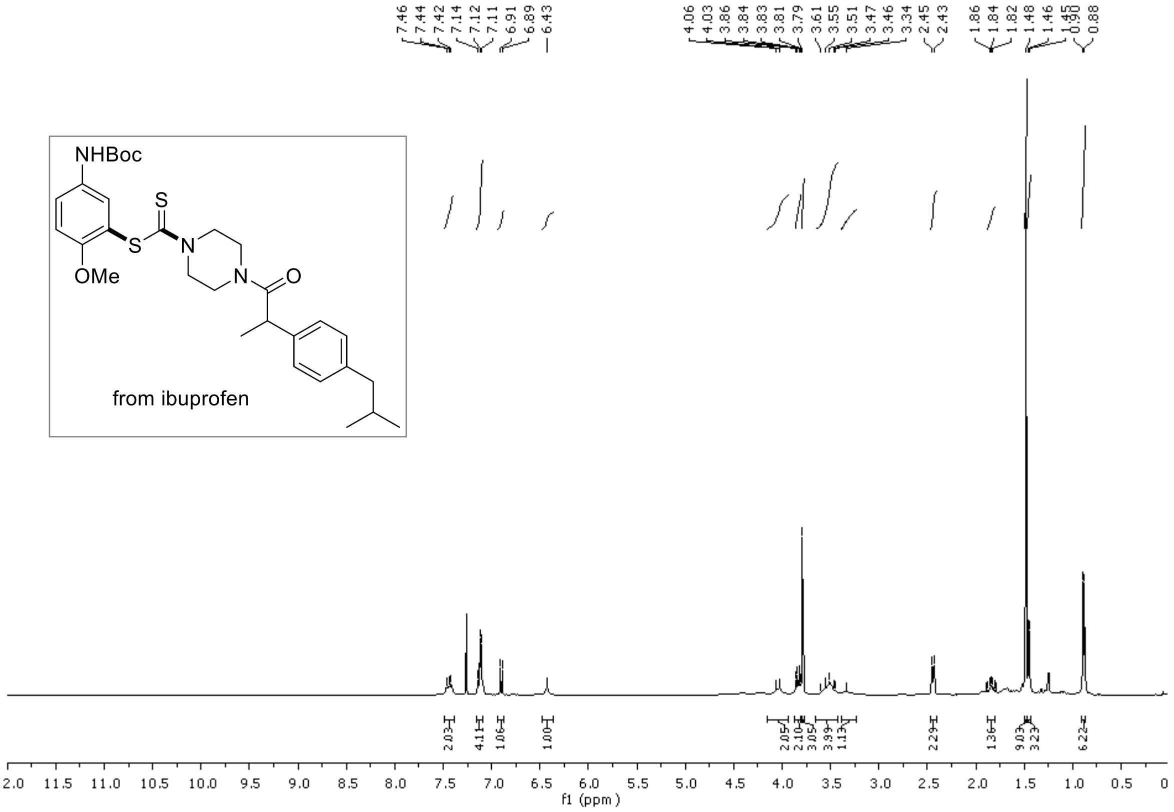


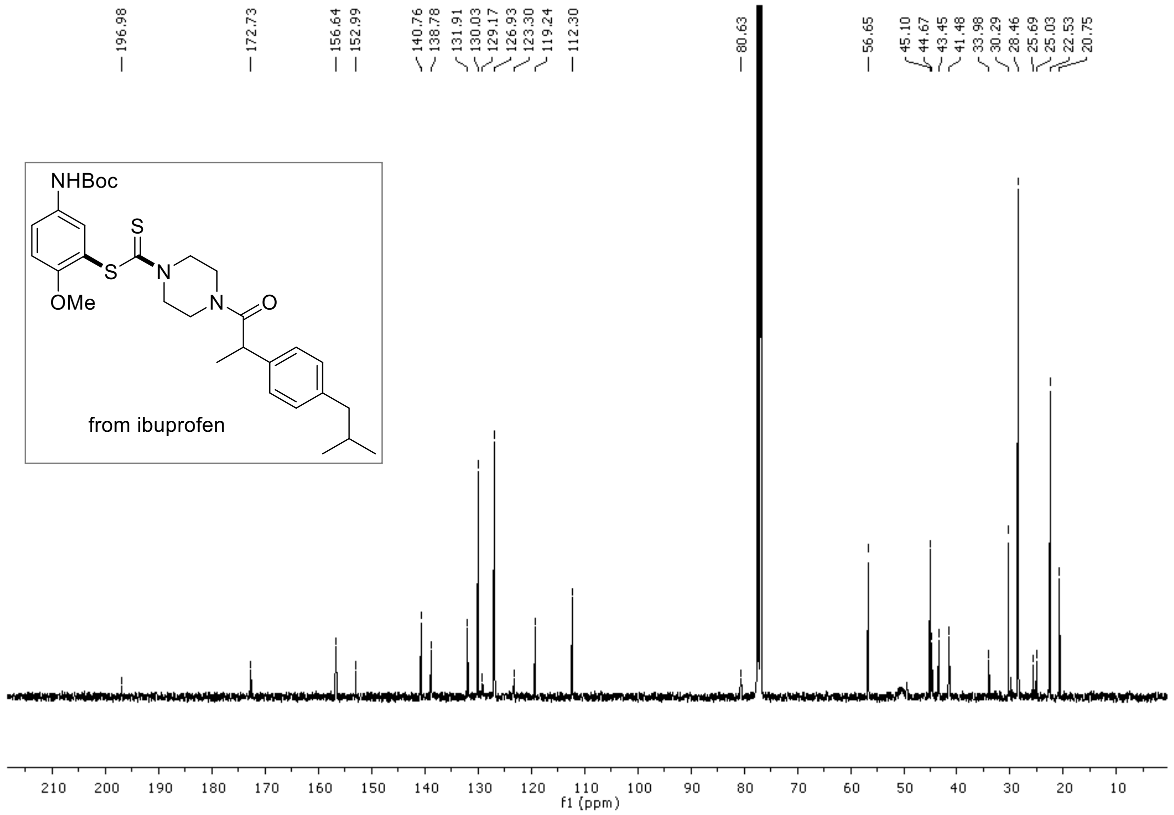

# $^{1}$H and $^{13}$C NMR of 43

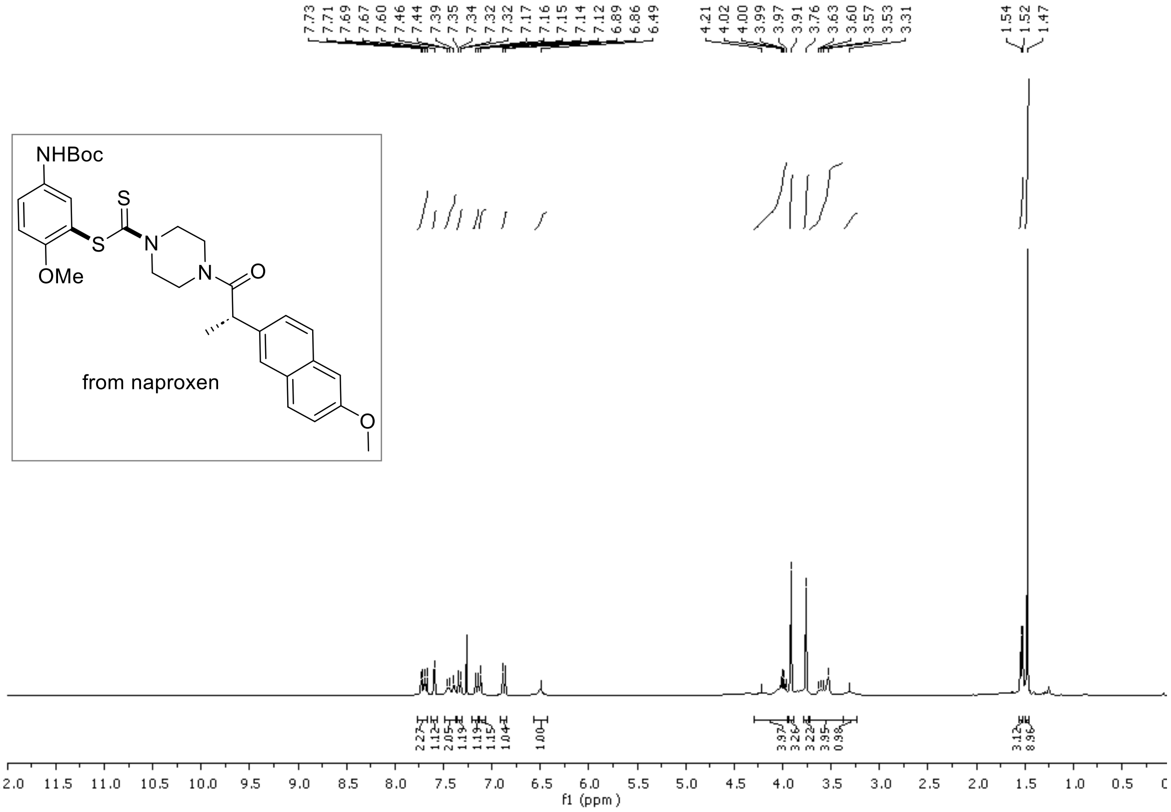


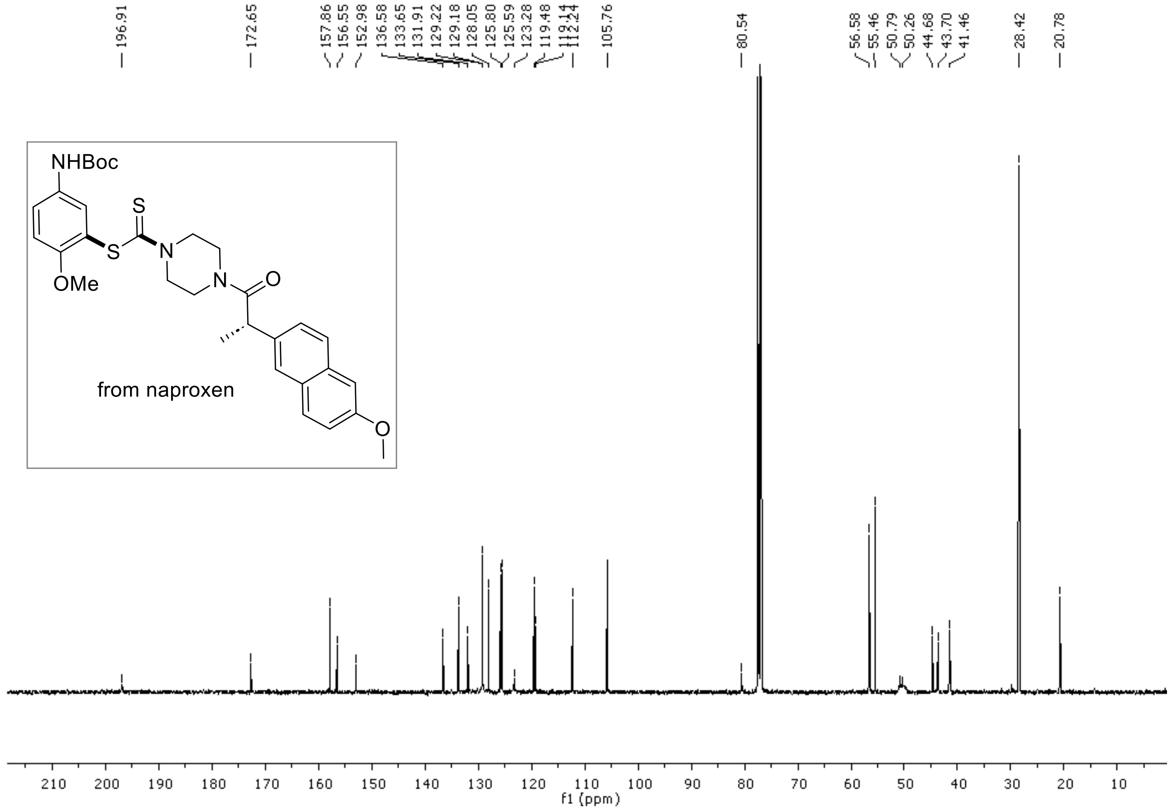

## $^{1}$H and $^{13}$C NMR of 44

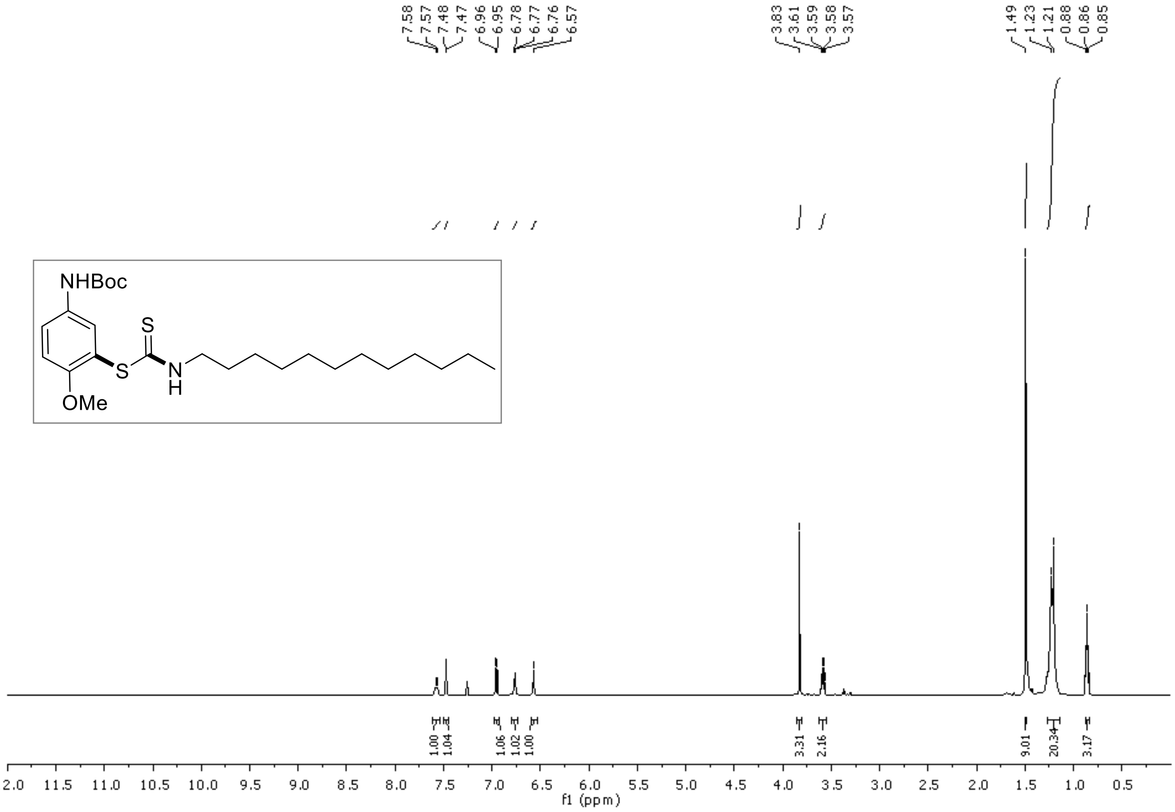

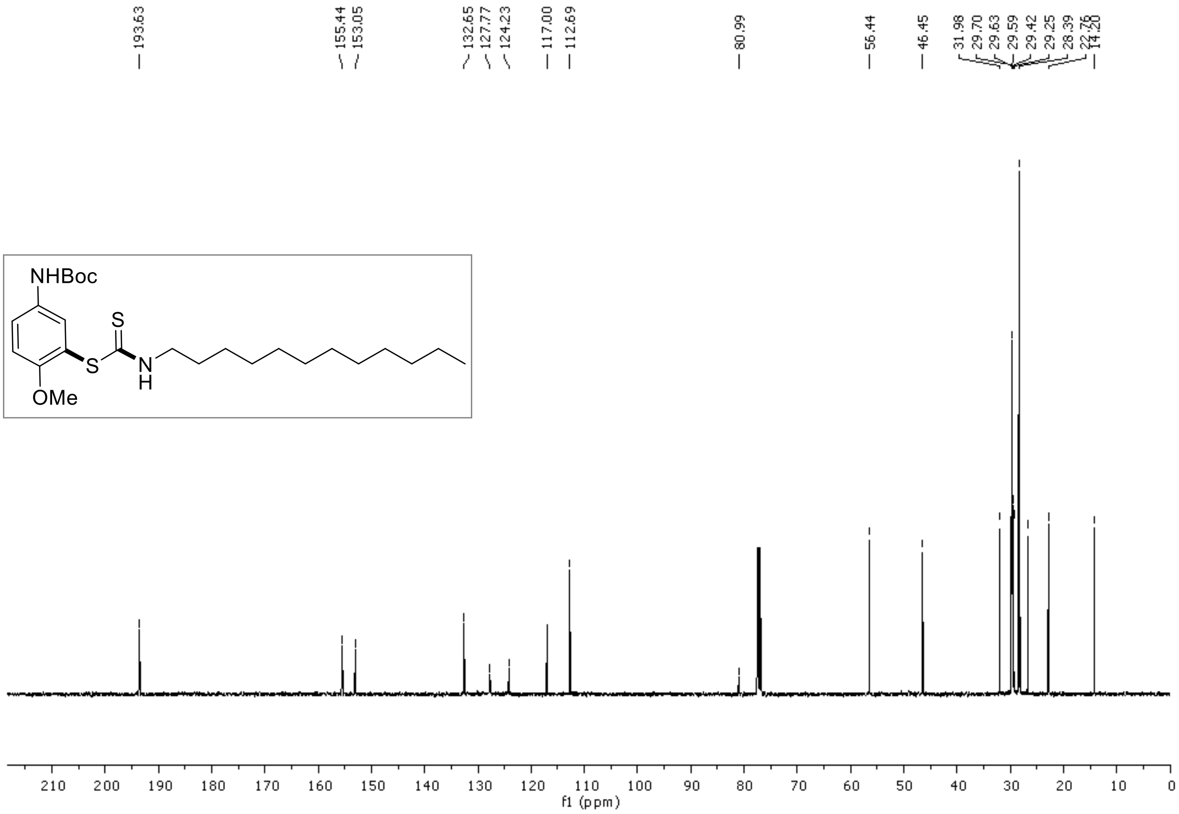

## $^{1}$H and $^{13}$C NMR of 45

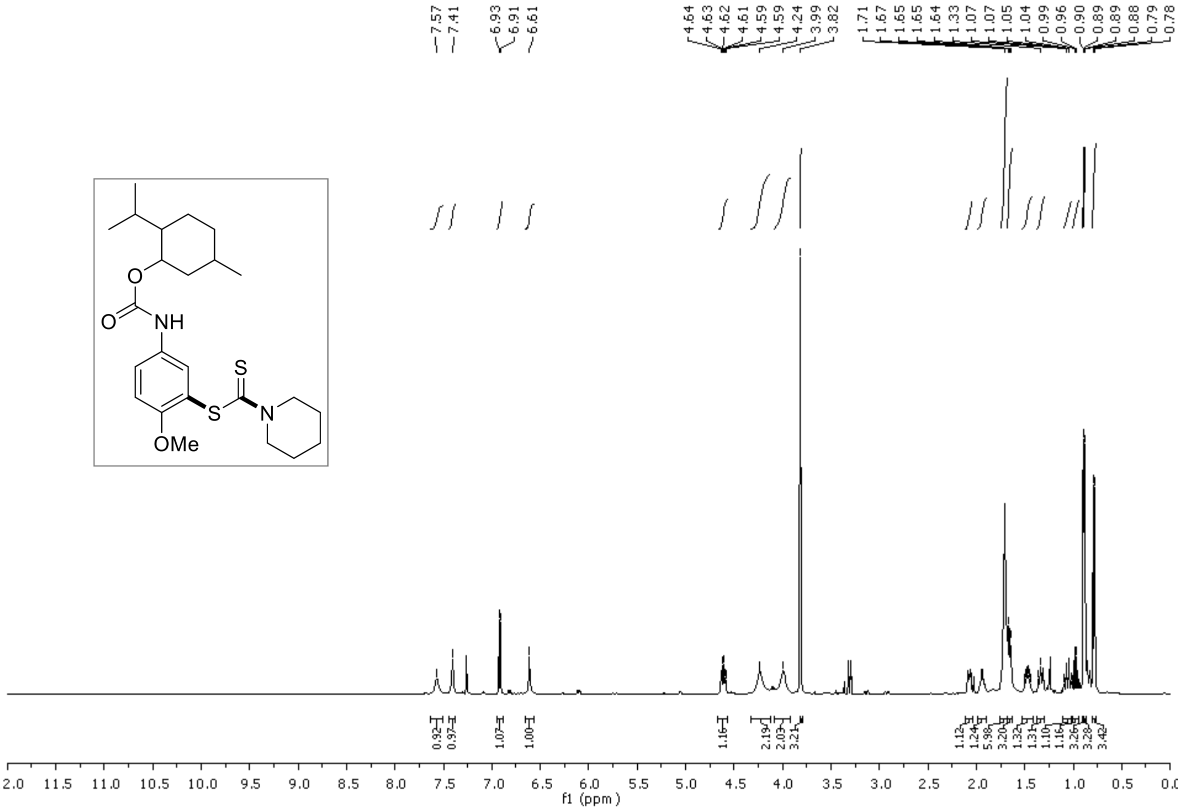


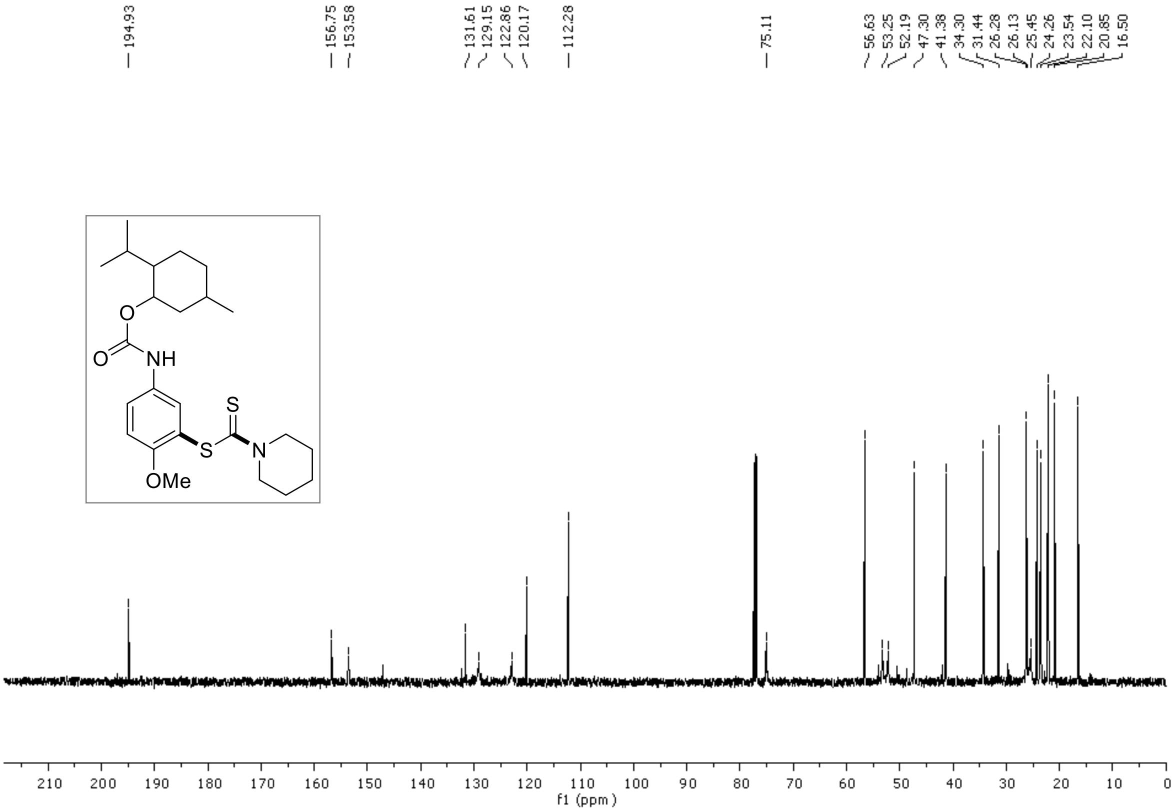

# $^{1}$H and $^{13}$C NMR of 46

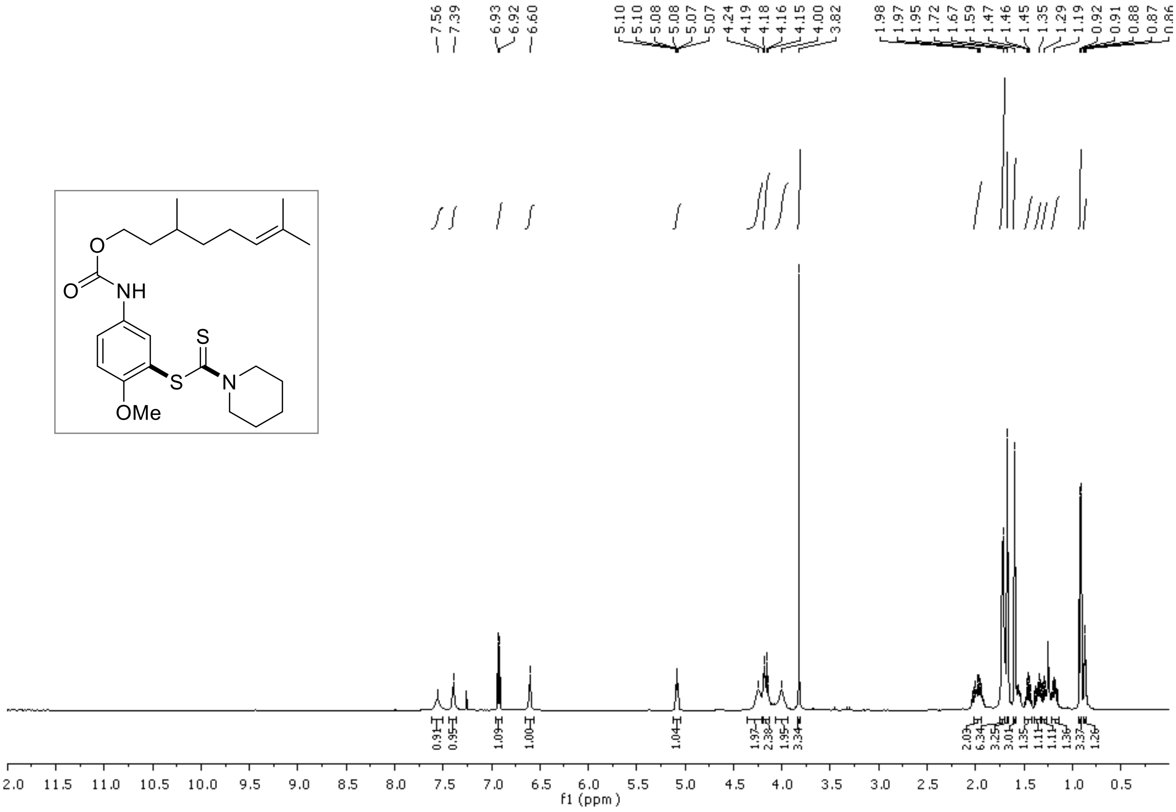


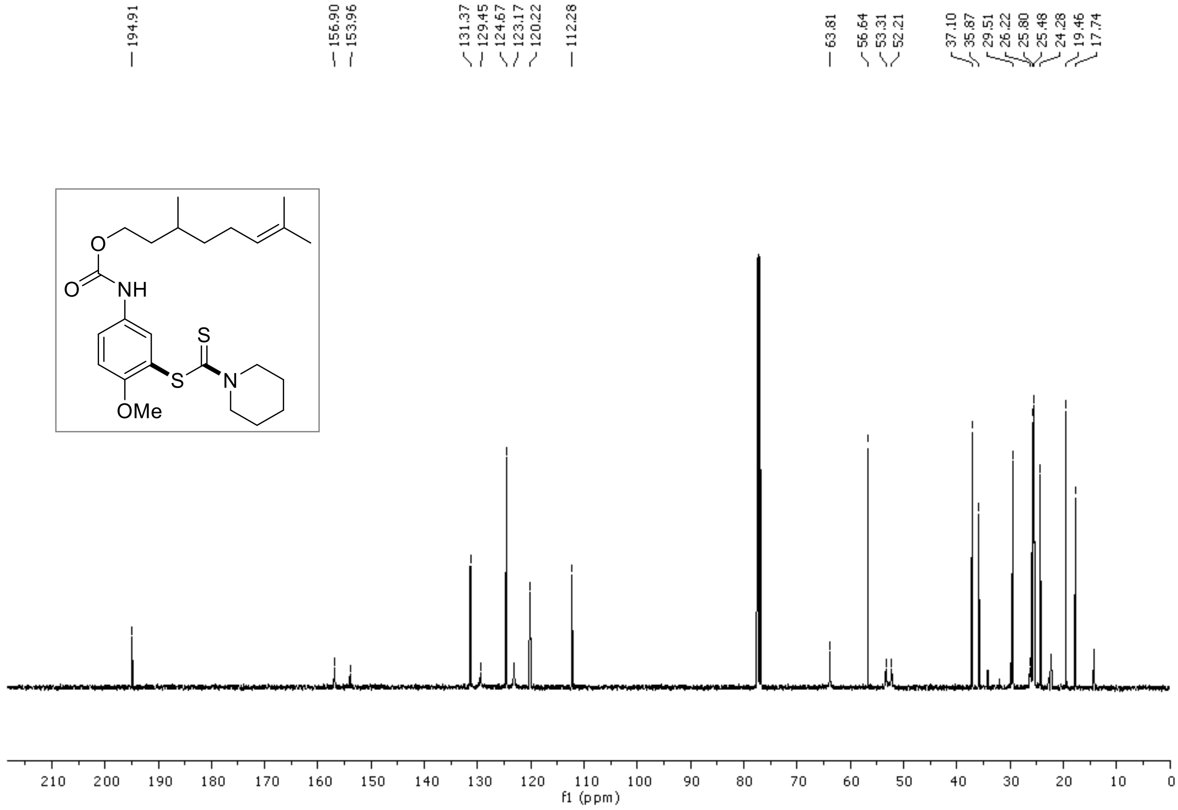

## $^{1}H$ and $^{13}C$ NMR of 47

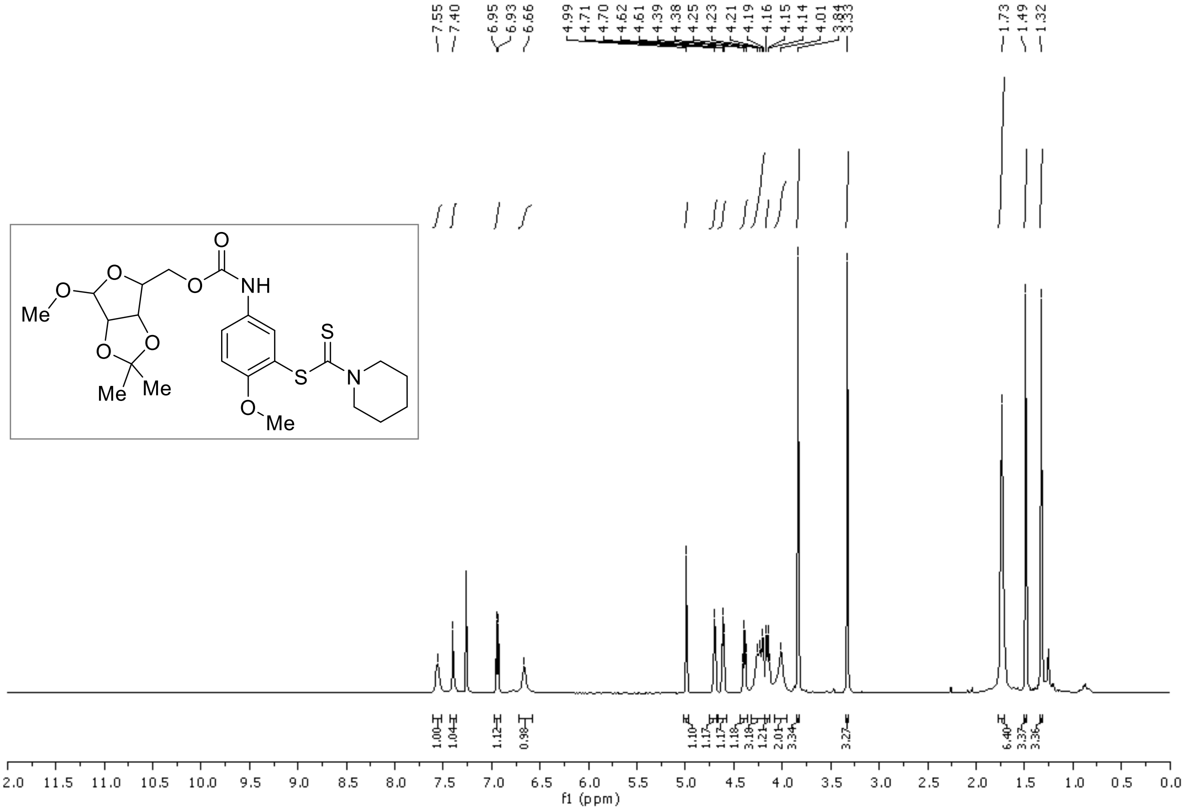


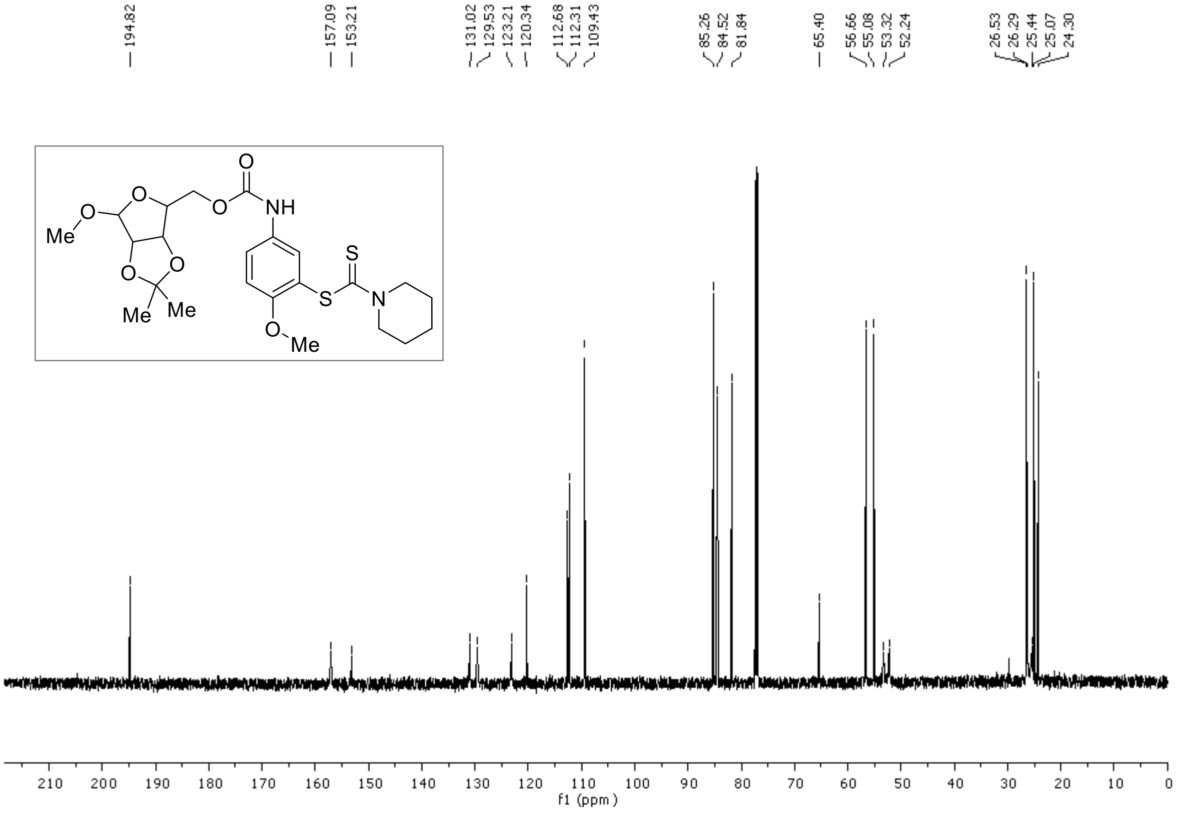

# $^{1}$H and $^{13}$C NMR of 48

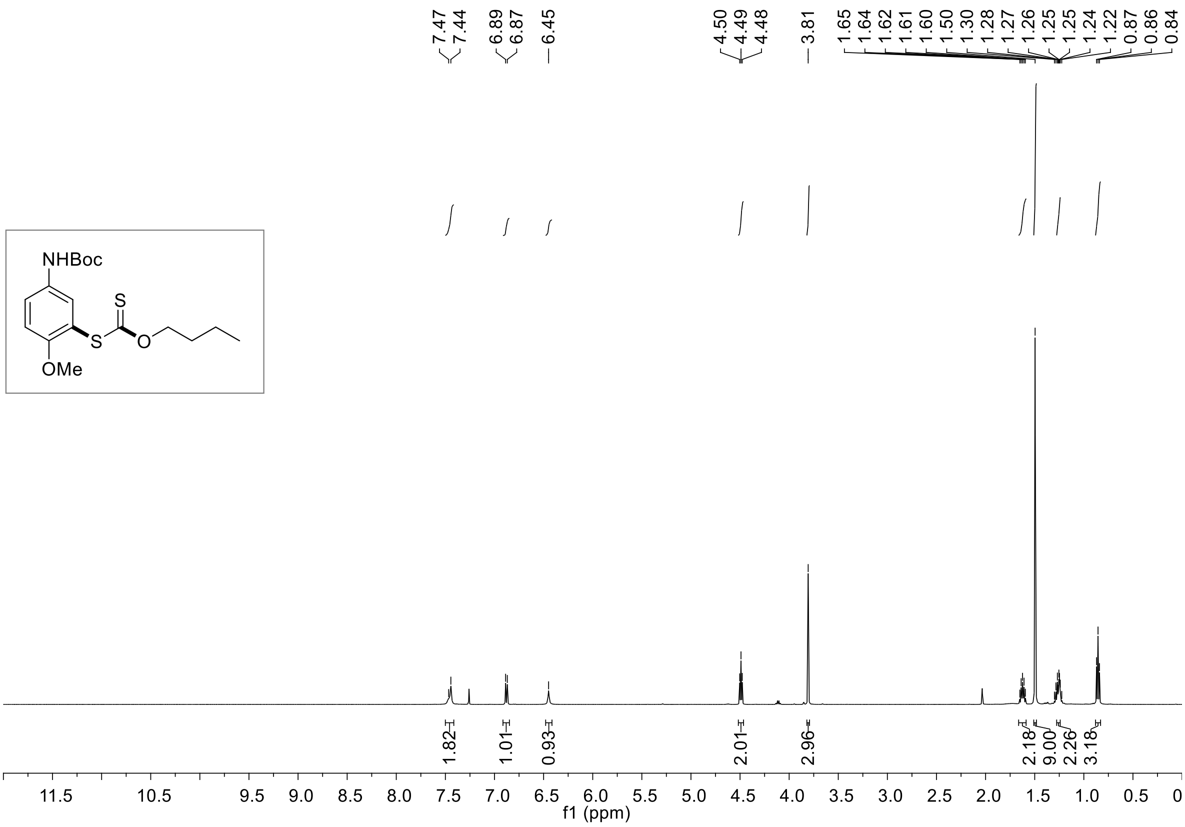


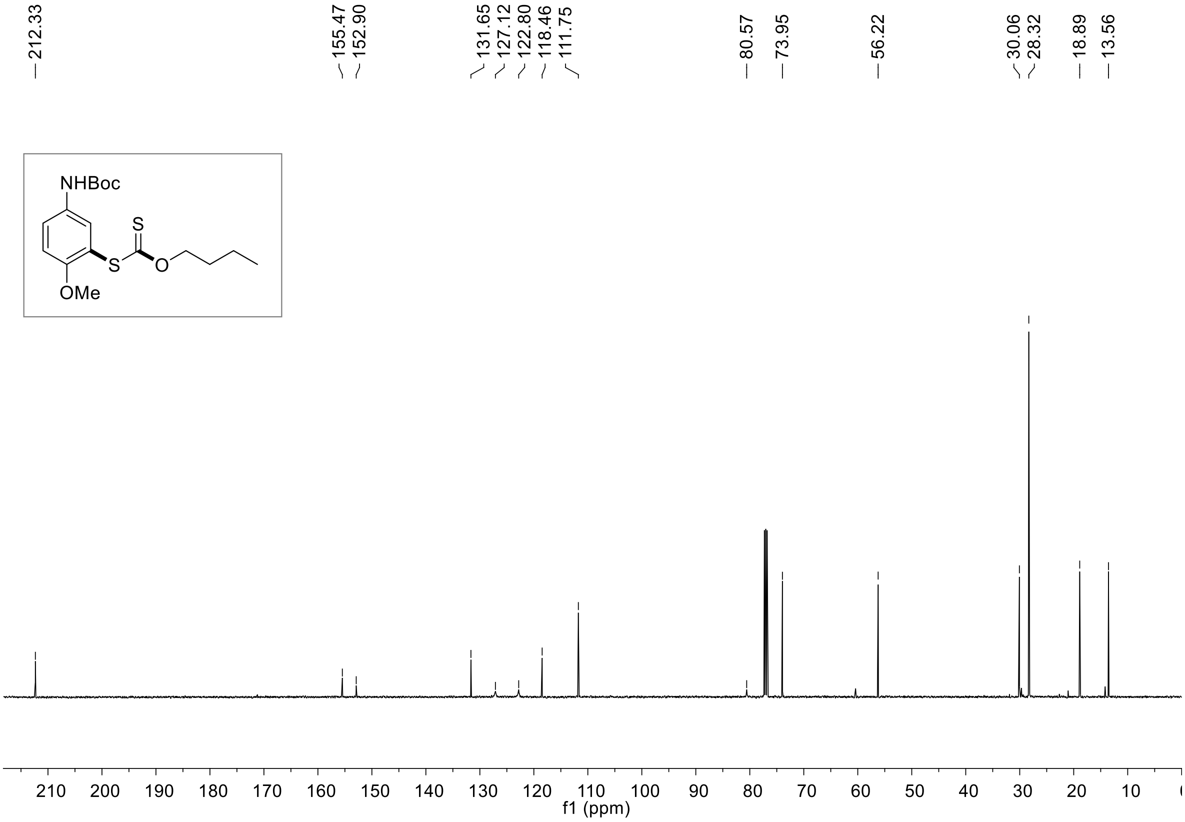

## $^{1}H$ and $^{13}C$ NMR of 50

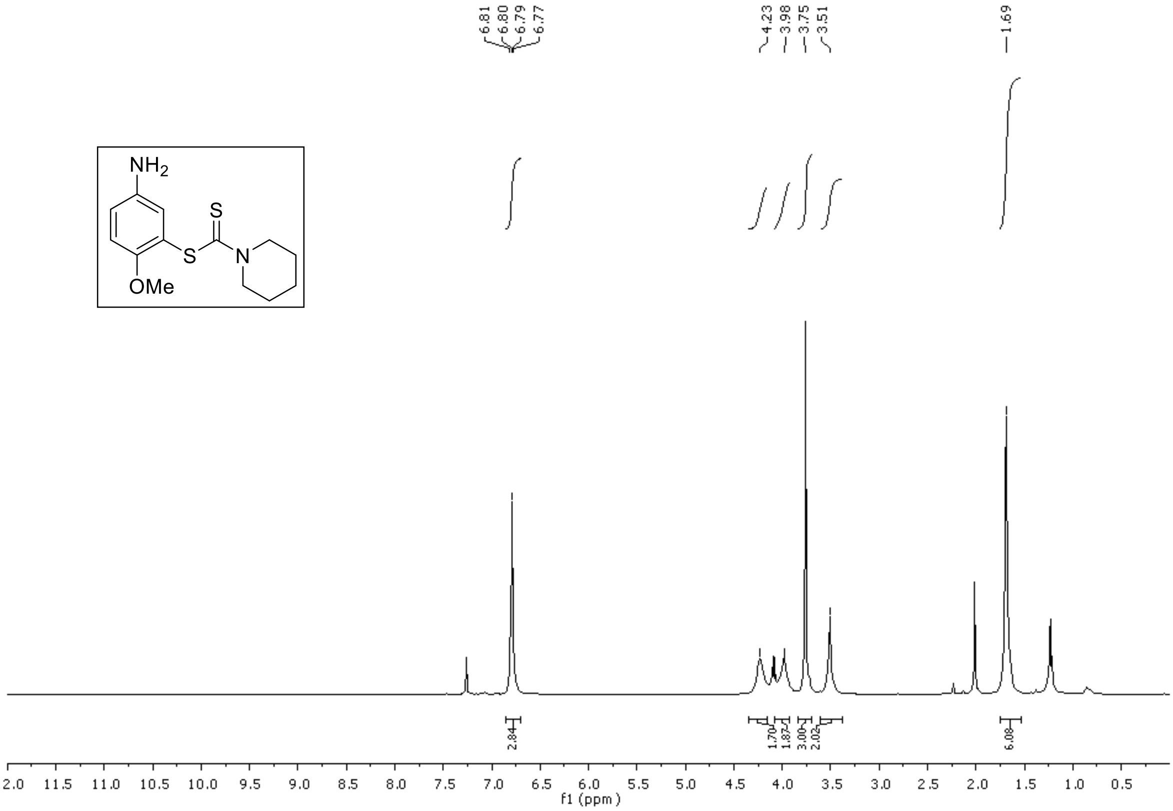


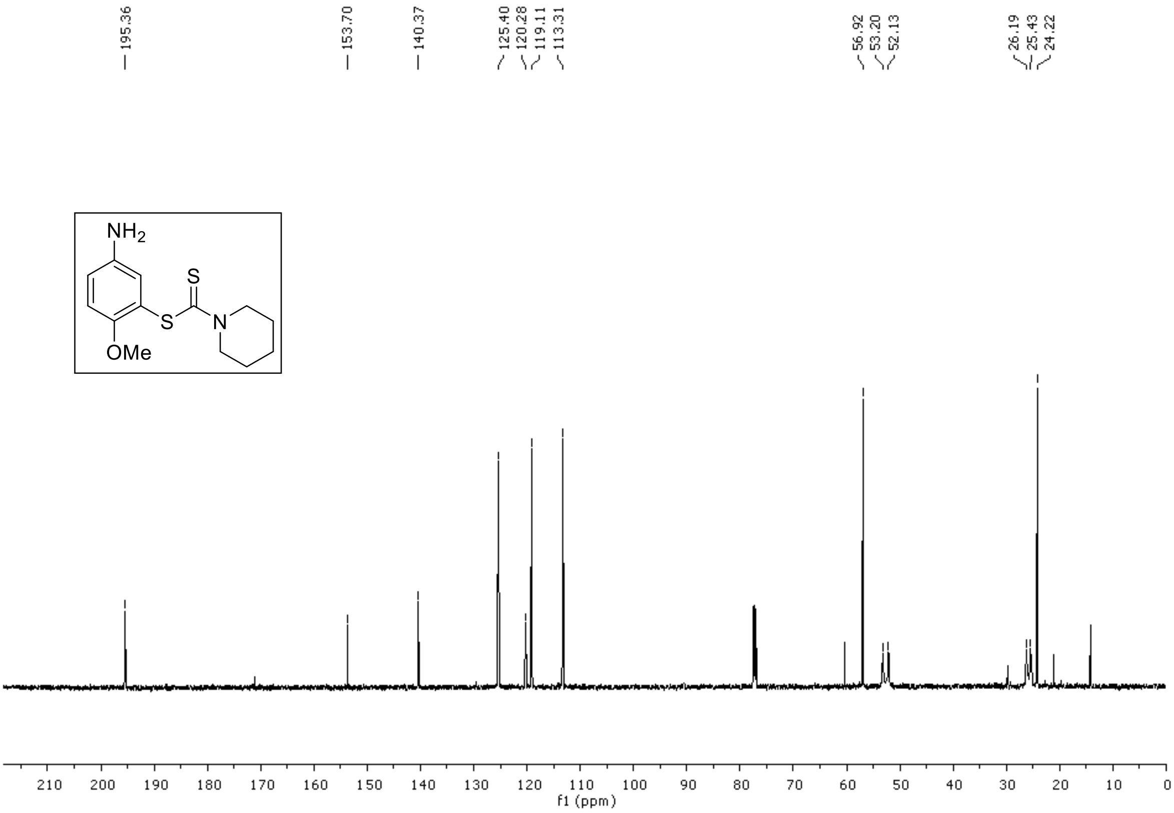

**$^1$H and $^{13}$C NMR of 51**

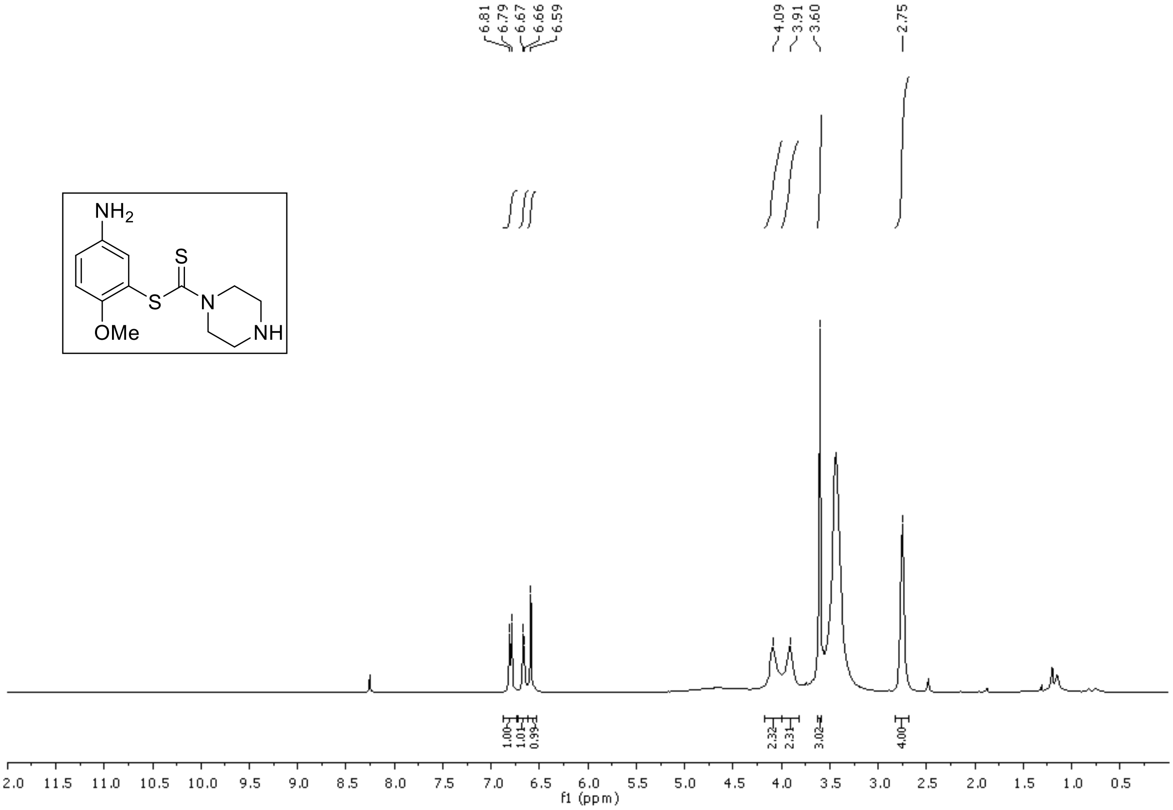


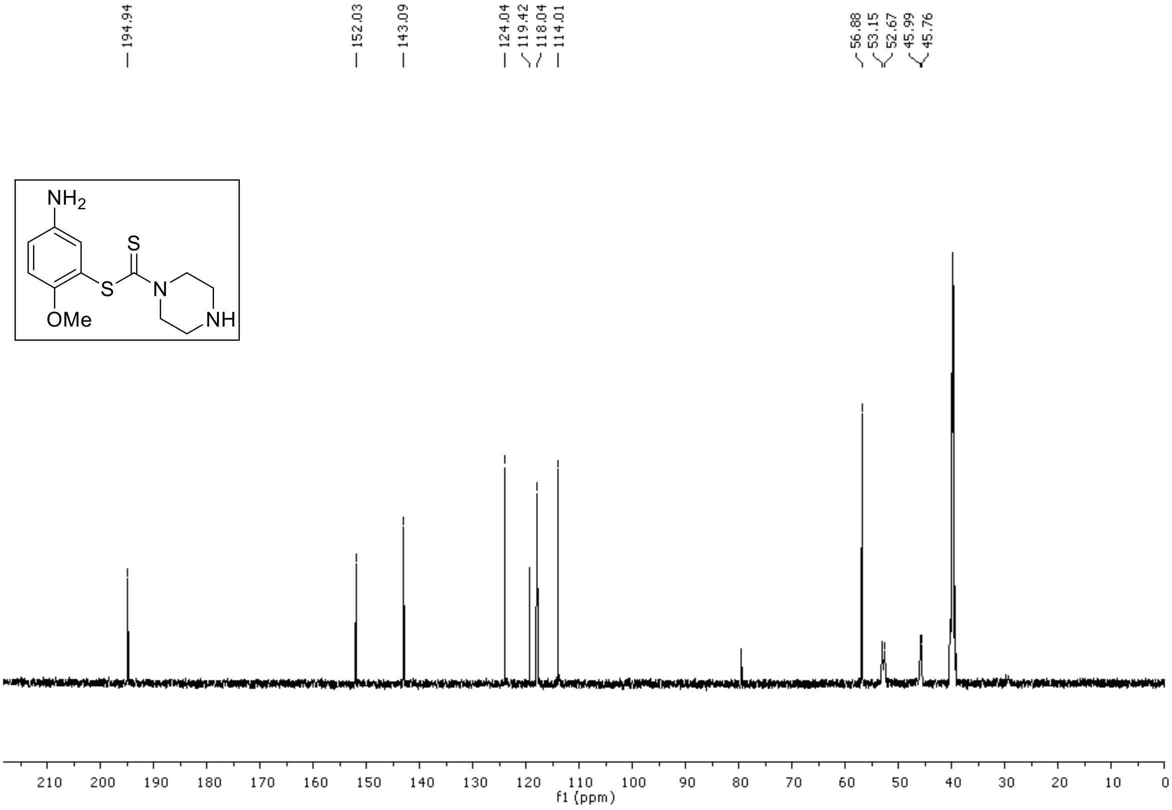

## $^{1}H$ and $^{13}C$ NMR of 52

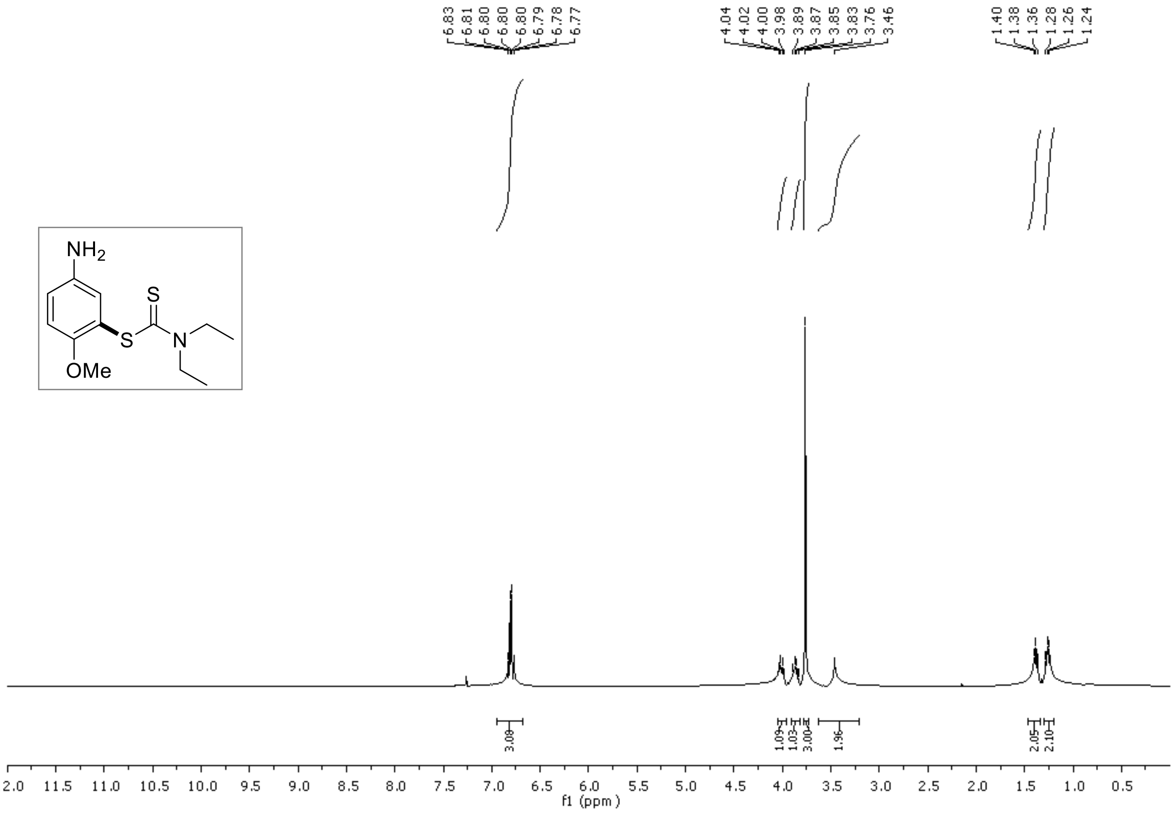


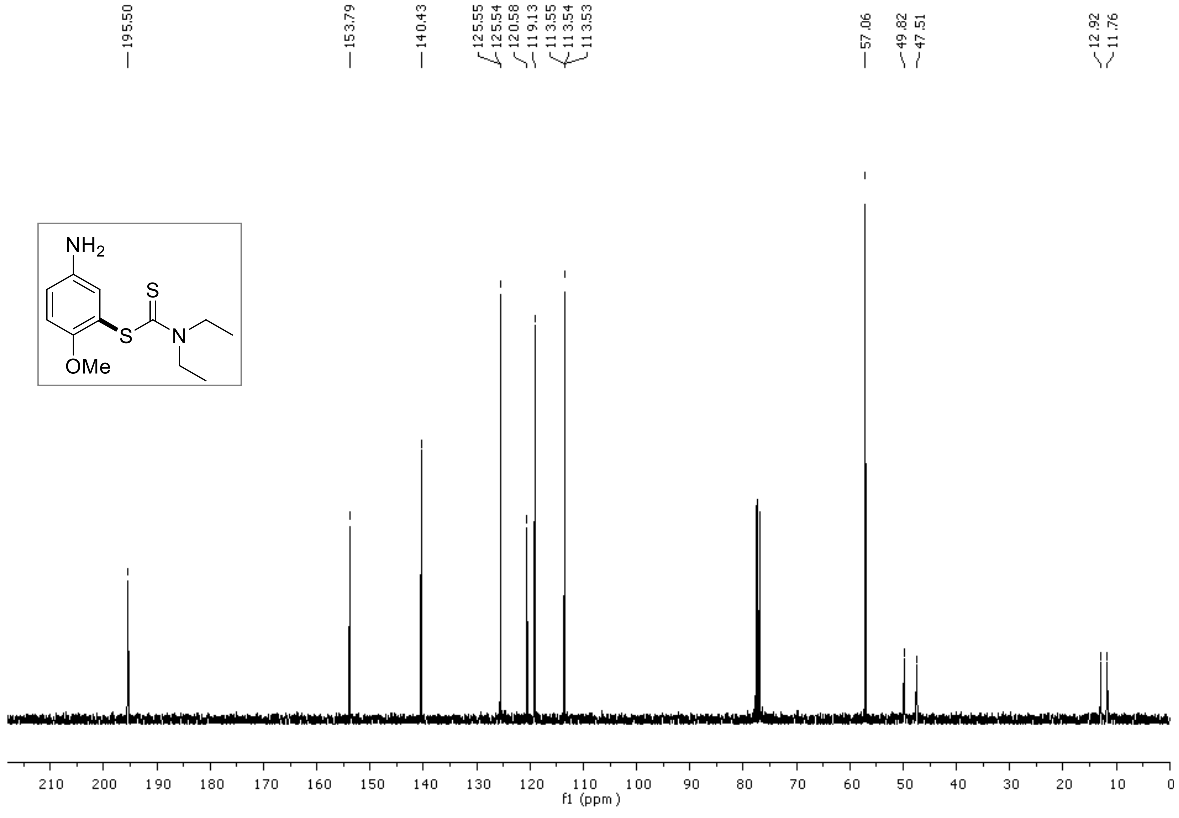

## $^{1}H$ and $^{13}C$ NMR of 53

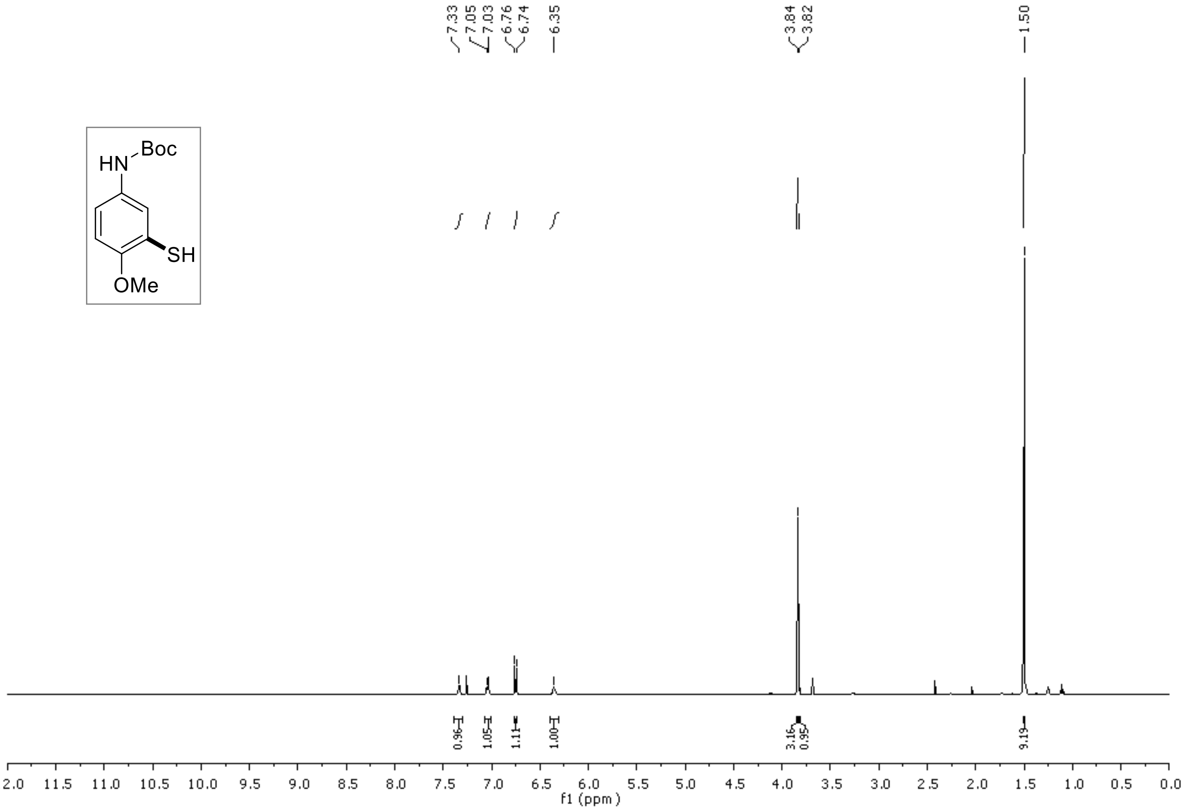


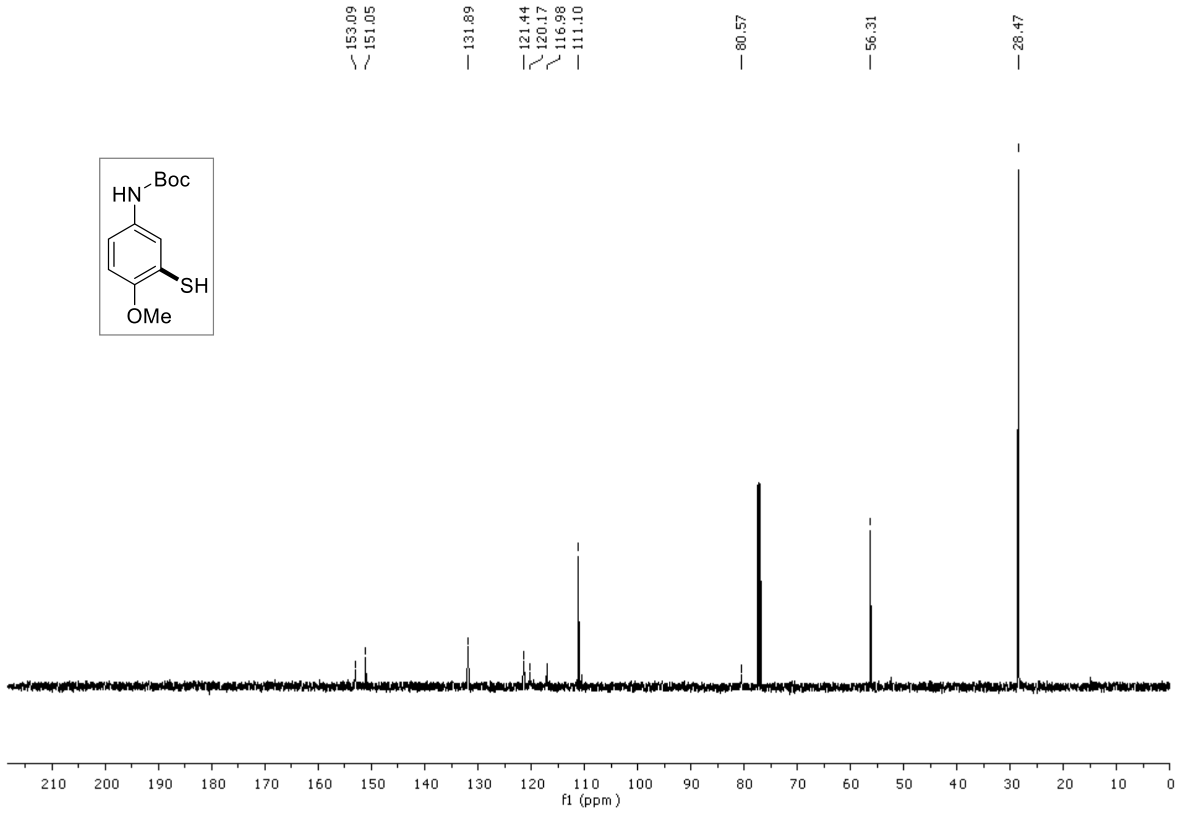

## $^{1}$H and $^{13}$C NMR of 54

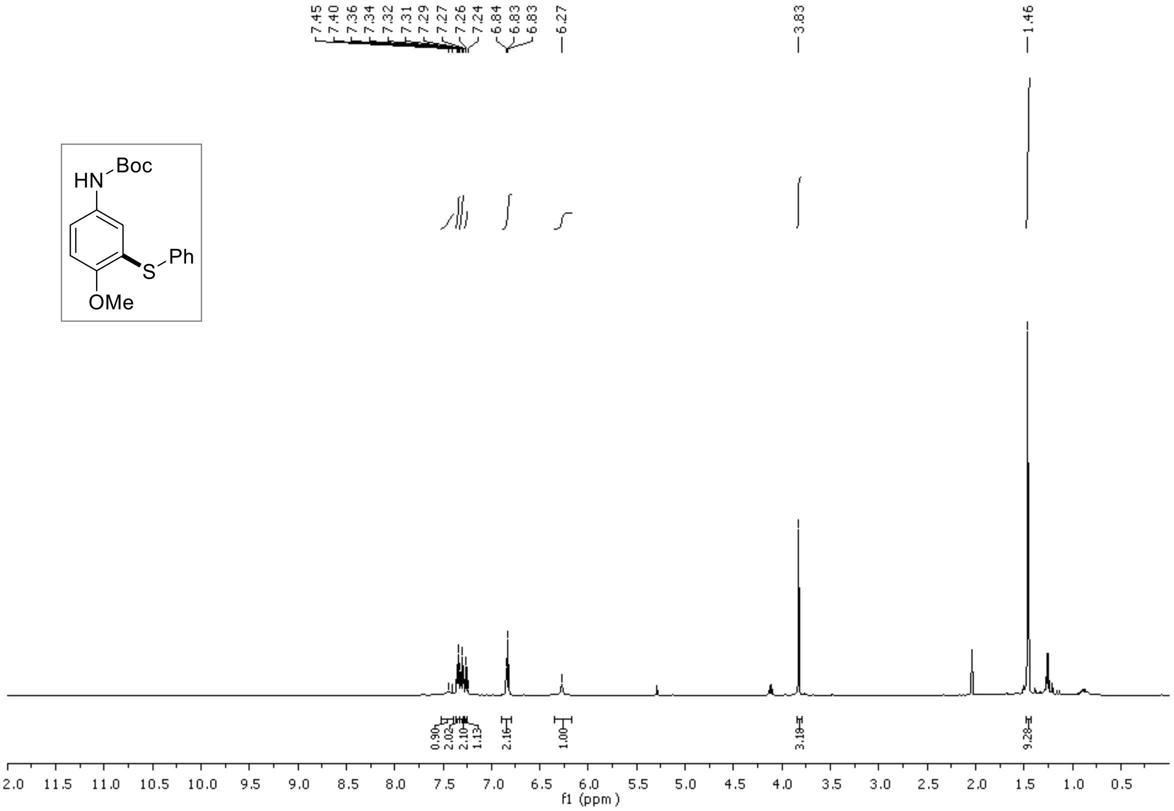


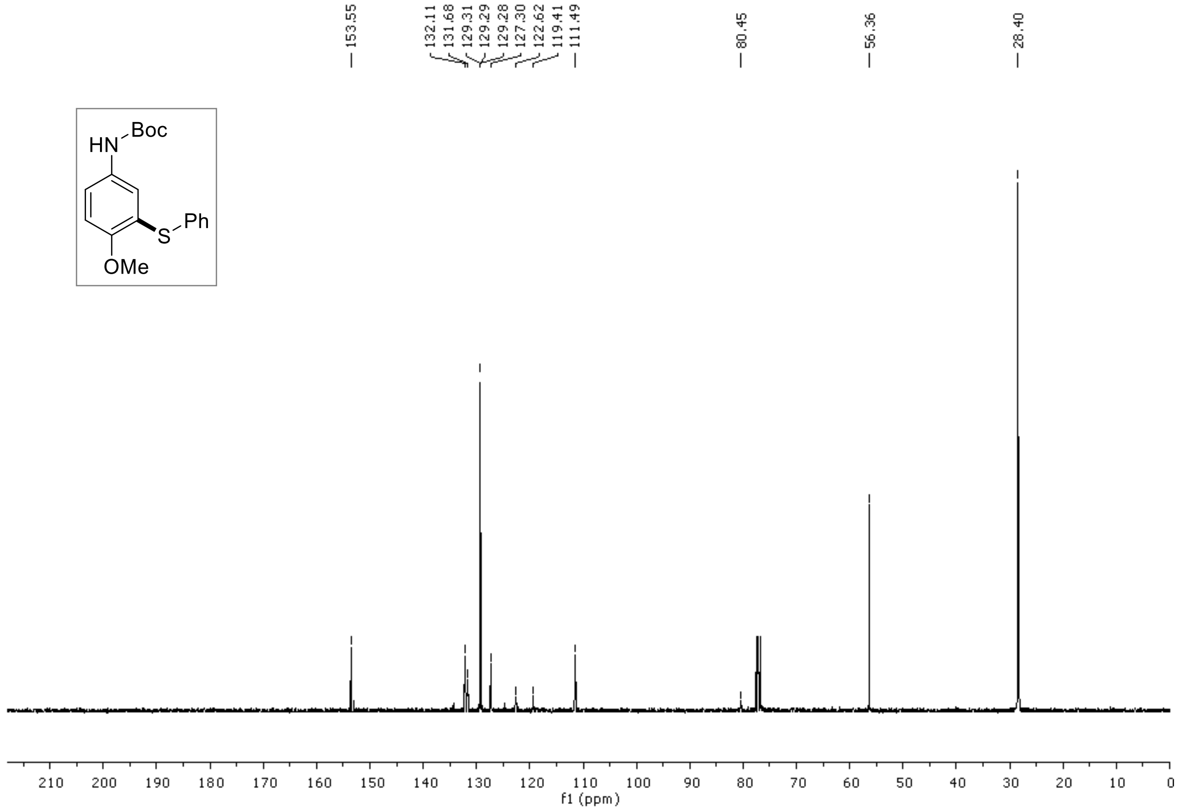

## $^{1}H$ and $^{13}C$ NMR of 55

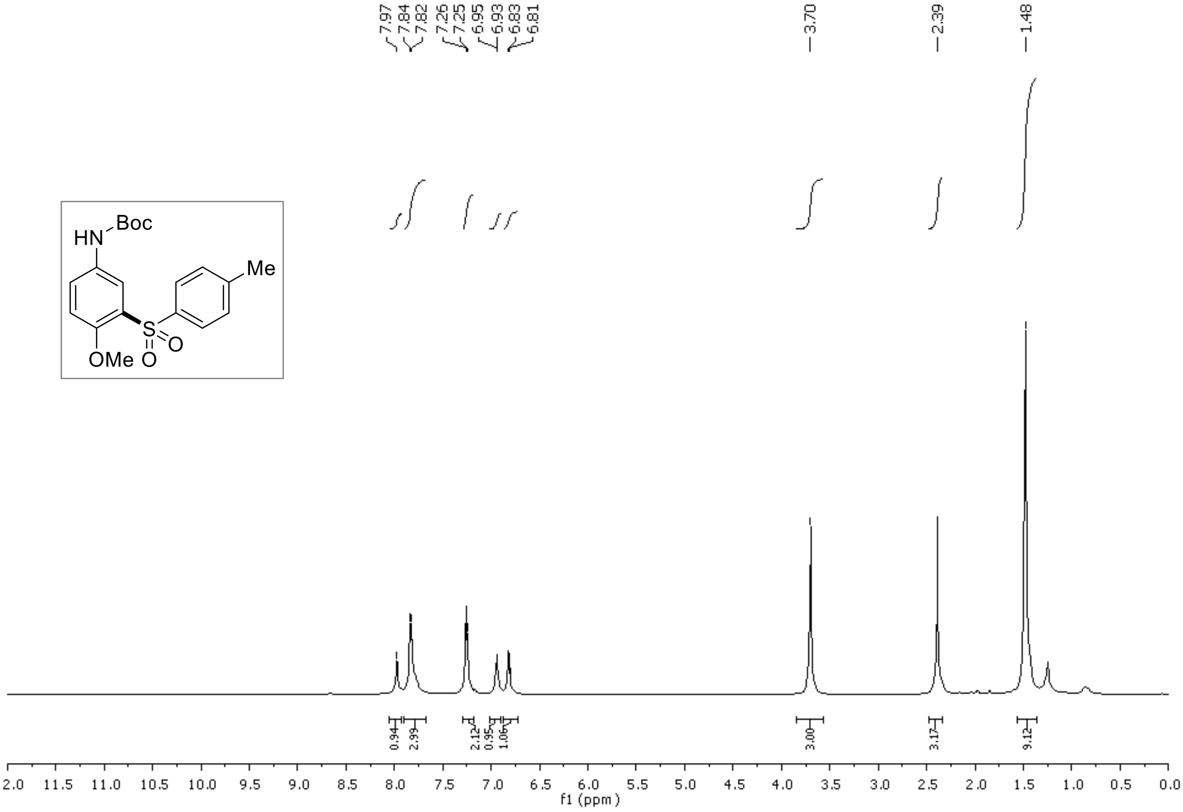


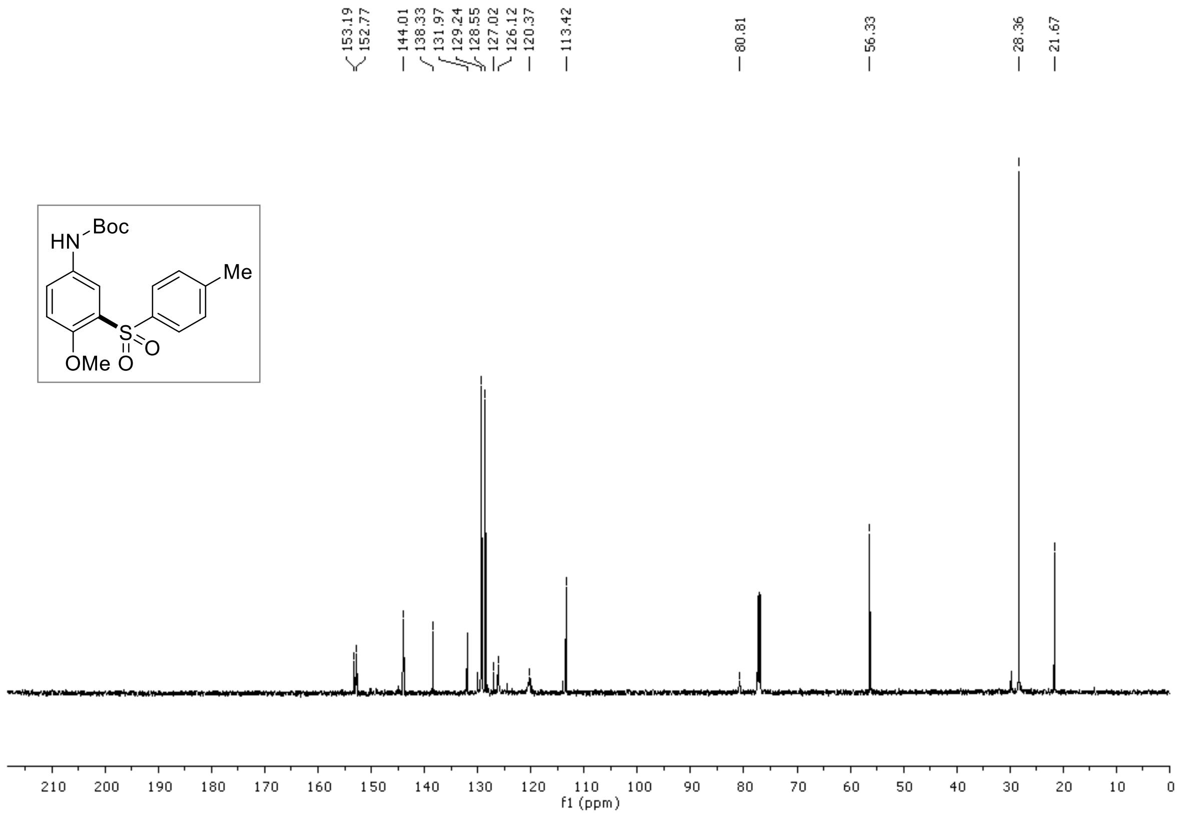

Supplement: Supplementary file 1 [file oc5c01231_si_001.pdf]
